# Global burden and trends of stroke attributable to kidney dysfunction from 1990 to 2021

**DOI:** 10.1093/ckj/sfaf160

**Published:** 2025-05-23

**Authors:** Zi-Han Yin, Qiong-Nan Bao, Jiao Chen, Fan-Rong Liang, Ling Zhao

**Affiliations:** School of Acu-Mox and Tuina, Chengdu University of Traditional Chinese Medicine, Chengdu, China; Acupuncture Clinical Research Center of Sichuan Province, Chengdu, China; School of Acu-Mox and Tuina, Chengdu University of Traditional Chinese Medicine, Chengdu, China; Acupuncture Clinical Research Center of Sichuan Province, Chengdu, China; School of Acu-Mox and Tuina, Chengdu University of Traditional Chinese Medicine, Chengdu, China; Acupuncture Clinical Research Center of Sichuan Province, Chengdu, China; School of Acu-Mox and Tuina, Chengdu University of Traditional Chinese Medicine, Chengdu, China; Acupuncture Clinical Research Center of Sichuan Province, Chengdu, China; School of Acu-Mox and Tuina, Chengdu University of Traditional Chinese Medicine, Chengdu, China; Acupuncture Clinical Research Center of Sichuan Province, Chengdu, China

**Keywords:** disability-adjusted life years, global burden of disease, kidney dysfunction, mortality, stroke and stroke subtypes

## Abstract

**Background:**

Accumulating evidence suggests that kidney dysfunction (KD) is a risk factor for stroke and stroke subtypes (SSS). However, comprehensive studies on the global burden of SSS attributable to KD are lacking. This study aimed to compare the long-term trends of KD-related SSS from 1990 to 2021 at the global, regional and national levels, and predict the disease burden until 2045.

**Methods:**

The dataset was collected from the 2021 Global Burden of Disease Study (GBD). The study estimated mortality and disability-adjusted life years (DALYs) counts, while also calculating estimated annual percentage changes (EAPCs) to evaluate long-term trends in age-standardized rates of mortality and DALYs. The analyses were stratified based on sex, 14 age categories, 5 socio-demographic index (SDI) quintiles, 21 GBD regions, and 204 nations and territories. Statistical analyses and visualizations were conducted using R version 4.4.2.

**Results:**

Between 1990 and 2021, KD-related stroke mortality rose by 40.4%, and DALYs were increased by 36.7%, with EAPCs of –1.8 and –1.7, respectively. KD-related ischaemic stroke mortality and DALYs grew by 45.9% and 47.4%, with EAPCs of –1.9 and –1.7. KD-related intracerebral haemorrhage mortality and DALYs increased by 35.3% and 28.7%, with EAPCs of –1.7 and –1.7. There was notable variation by sex and age. The major burden was located in the middle SDI region and East Asia (especially in China). Decomposition analyses revealed an increase burden in total KD-related SSS, with a positive contribution from population growth and aging. The burden of KD-related SSS has steadily risen and is expected to keep growing until 2045.

**Conclusion:**

Despite a slight decrease in long-term trends, this study highlights a significant rise in the global burden of KD-related SSS, with notable variations across SDI areas, GBD regions, countries, sexes and age groups. This increasing challenge necessitates specific therapies and public health initiatives for KD-related SSS.

KEY LEARNING POINTS **What was known:**

- Kidney dysfunction (KD) is a significant global health concern, closely linked to an increased risk of stroke and its subtypes (SSS).
- However, comprehensive studies on the global burden of SSS attributable to KD are lacking.

**This study adds:**

- This study is the first, most recent, and comprehensive assessment to investigate the global burden of KD-related SSS using the 2021 GBD study, revealing novel evidence-based insights.
- In addition, our findings highlight the distinct challenges faced by individuals with KD-related SSS, underscoring the critical need for KD interventions and therapies targeting the kidney–brain axis.

**Potential impact:**

- This research will form the basis for subsequent research and policymaking initiatives aimed at enhancing the understanding and management of KD-related SSS in this vulnerable demographic region.

## INTRODUCTION

Stroke is a cerebrovascular event that occurs when blood flow to the brain is disrupted, leading to neuronal death and impaired cerebral function [1]. Stroke has three main subtypes: ischaemic stroke (IS), intracerebral haemorrhage (ICH) and subarachnoid haemorrhage (SAH) [2]. According to the World Health Organization (WHO), stroke is the leading cause of disability worldwide and the second leading cause of death, responsible for approximately 10% of all deaths in 2022 [3]. Recent stroke surveillance data from China reported disability rates of 14.8% at 3 months and 14.0% at 12 months among survivors. This burden is expected to intensify as the population continues to age at an accelerating pace [4]. Overall, stroke imposes a substantial death and disability burden on both individuals and society.

Growing evidence highlights the important role of kidney dysfunction (KD) in stroke development [5]. The kidney and brain share physiological traits: both receive high blood flow, maintain constant perfusion despite blood pressure changes through autoregulation, and exhibit haemodynamic similarities in their vascular systems—these shared traits provide a physiological intricate linkage between KD and stroke [6]. Numerous cases have found KD to be an important risk factor for stroke and is associated with more severe stroke and poorer outcomes [7, 8]. A large case–control study involving 21 127 individuals across 27 countries identified a link between KD and an increased likelihood of stroke [6]. Mendelian randomization further revealed an independent causal relationship between impaired kidney function and elevated risk of stroke [2]. Therefore, understanding the current burden of KD-related stroke and forecasting its trends over time are essential for selecting prevention and treatment strategies.

Previous studies have generally focused on the global burden and risk factors of stroke or its subtypes [9–11]; however, comprehensive and systematic analyses of the global burden specifically associated with KD-related stroke are lacking. Additionally, evolving trends in this disease burden remain underexplored, hindering efficient prediction and resource planning for acknowledging and preventing stroke in clinical guidance. The Global Burden of Disease Study (GBD) is a collaborative and continuously updated research project led by the Institute for Health Metrics and Evaluation. It provides a comprehensive assessment of disease epidemiology, covering mortality and disability-adjusted life years (DALYs) for 369 conditions across 204 countries from 1990 to 2021, stratified by sex, age, sociological factors, and geographic region. The GBD datasets provide the opportunity to explore the trends in the burden of KD-related stroke and stroke subtypes (SSS).

In this study, we retrieved detailed, updated data from the GBD 2021 datasets to analyse the mortality and DALY trends of KD-related SSS from 1990 to 2021. The analysis was stratified by age, sex, year, social development index, global SSS, socio-demographic index (SDI) areas, GBD regions and nations/territories. This study aimed to examine the global burden and evolving trajectories of KD-related SSS. Additionally, we utilized time-series models to project the disease burden to 2045. These findings offer valuable insights into policymaking and initiatives aimed at controlling KD exposure and protecting high-risk individuals.

## MATERIALS AND METHODS

### Study population and data collection

KD-related SSS data were analysed from the GBD 2021 dataset, which offers the latest estimates for disorders and risk elements across 204 nations and territories organized into 21 areas [12–14]. This study employed the GBD 2021 classification system, which categorizes the world into 21 regions based on geographic proximity and epidemiological similarities [4, 15]. This categorization enabled a detailed perception of global variations in disease burden, facilitating the formulation of targeted public health policies and interventions. The geographical categorization system has consistently proven effective in previous iterations of the GBD research, facilitating the analysis and comparison of health metrics across areas with diverse geographical and epidemiological profiles [4, 15]. For this study, we selected data from adults with SSS attributable to KD, including mortality and DALY rates. These metrics are presented with their 95% uncertainty intervals (UI). To calculate DALYs, both the years lived with disability and the years of life lost were considered, offering a comprehensive view of the disorder burden. The age range spans from 25 to 94 years.

We contextualized our findings by using the SDI, a comprehensive measure that assesses local development using indicators like economic income, educational levels and fertility statistics [16]. In the GBD 2021 dataset, nations and territories are classified into five SDI strata spanning a five levels of development (high, high-middle, middle, low-middle and low). All utilized GBD data are available to the public on the Global Health Data Exchange (GHDx) platform (https://vizhub.healthdata.org/gbd-results/). For GBD studies, the Institutional Review Board of the University of Washington granted a waiver of informed consent. Our research was conducted according to Strengthening the Reporting of Cohort Studies in Surgery (STROCSS) guidelines [17] and the Guidelines for Accurate and Transparent Health Estimates Reporting (GATHER) [18].

The study is an integrated and anonymous analysis based on the open-source data from GBD database. Therefore, no additional informed consent was required.

### Definitions

The GBD 2021 classification system ranks stroke at the tertiary level within the larger framework of cardiovascular diseases (Level 2) and noncommunicable diseases (Level 1) [14]. For our analysis, we extracted the KD-related strokes specific to the KD-related IS and ICH data. According to the WHO, stroke is clinically defined as the abrupt onset of localized neurological deficits persisting for more than 24 h or leading to mortality. IS was defined as a vascular event that restricted cerebral blood flow and led to tissue infarction, including atherosclerotic and thromboembolic factors, excluding those that resulted in ICH. ICH is a type of stroke characterized by the localized accumulation of blood within the brain parenchyma, occurring independently of traumatic injury. Additionally, the GBD study [15, 19–21] classified KD based on an estimated glomerular filtration rate (eGFR) <60 mL/min/1.73 m^2^ or an albumin–creatinine ratio (ACR) of 30 mg/g or higher. The definition of KD in the GBD study differs from that outlined in the KDIGO 2012 Clinical Practice Guidelines [22]. Within the GBD framework, KD is defined with a focus on eGFR and ACR, with ACR data systematically incorporated from 2012 onward. In contrast, earlier years primarily relied on eGFR, which has been a standardized measure of kidney function since 1990.

### Statistical analysis

All statistical analyses were conducted using R software (v4.4.2), with statistical significance set at *P*-values <.05. Our findings clarify and interpret the statistical test results encompassing effect sizes, confidence intervals (CI), UI and *P*-values.

(i) Global, regional and national/territorial trends (1990–2021): this study aimed to measure the global trends in KD-related SSS from 1990 to 2021 for mortality and DALY rates. Age-standardized rates and estimated annual percentage change (EAPC) were used to investigate data trends across different time periods [23]. The age-standardized rate per 100 000 population is calculated using the following formula [24]:
  \begin{eqnarray*}Age - \textit{standardized}\ \textit{rates} = \frac{{\mathop \sum \nolimits_{i = 1}^A {a}_i{{\mathrm{\omega }}}_i}}{{\mathop \sum \nolimits_{i = 1}^A {{\mathrm{\omega }}}_i}} \times 100\ 000\end{eqnarray*}
  where *a*_*i*_ and ω_*i*_ denote age-specific rates and the number of people (or weight) in the same age subgroup of the chosen reference standard population (where *i* denotes the age class). EAPC represents the annual percentage change, and a linear regression model was used to calculate the 95% CI for EAPC. In addition, the subgroup analyses were conducted by age and sex.
(ii) Stratified trends and correlation analysis: the study further conducted a correlational trend analysis examining mortality and DALY rates in relation to the SDI, using age-standardized rates stratification (per 100 000 individuals). This phase involved identifying point estimates for 204 countries and territories. The fitting of linear model and Spearman's correlation was performed, and correlational graphs were drawn.
(iii) Contributions to disease burden: the decomposition approach [25, 26] was used to assess the contributions of population age structure, growth and epidemiological changes to the observed trends in KD-related SSS mortality and DALYs. This method facilitated understanding the associated contributions of these factors to the evolving burden of KD-related SSS and determining whether they have led to an increase or decrease in this burden.
(iv) Forecasting future disease burden: the Bayesian age–period–cohort (BAPC) model [11, 27] has been widely used to estimate marginal posterior distributions, particularly in forecasting future disease burden or health outcomes across populations or time periods. We utilized the integrated nested Laplace approximations to bypass the challenges frequently encountered with Markov chain Monte Carlo methods in Bayesian analysis. Furthermore, a sensitivity analysis using nordpred model was conducted to evaluate the robustness of the BAPC model forecasts under varying prior distributions and model specification [28, 29].

## RESULTS

### Global level trends

**Figure 1:**
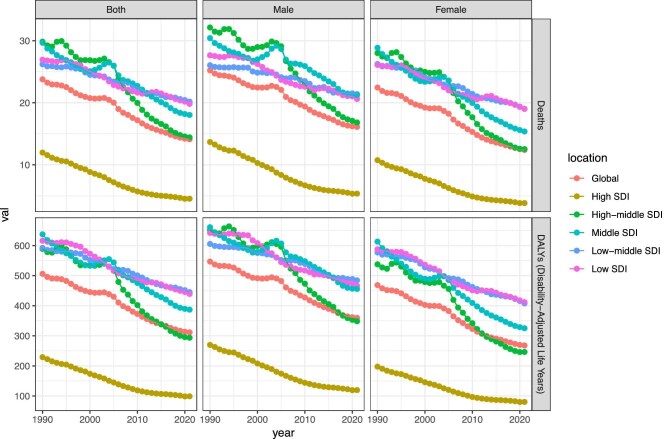
Trends in KD-related stroke mortality and DALYs from 1990 to 2021.

For KD-related stroke, the mortality cases has risen 40.4% from 465 801.2 (95% UI 310 730.9 to 637 231.3) in 1990 to 654 183.6 (95% UI 427 040.4 to 909 865.0) in 2021, with an EAPC of –1.8 (95% CI –1.9 to –1.7); the number of DALYs has also risen 36.7% from 10 843 730.1 (95% UI 7 638 187.5 to 14 470 967.6) in 1990 to 14 820 610.7 (95% UI 10 194 050.9 to 20 051 858.6) in 2021, with an EAPC of –1.7 (95% CI –1.8 to –1.6) (Table 1, Fig. 1).

**Table 1:** Global and regional trends in stroke attributable to KD burden: mortality and DALYs (1990–2021).

	1990		2021		
Location	Number	ASR, per 100 000 persons	Number	ASR, per 100 000 persons	EAPC (95% CI)
Deaths					
Global	465 801.2 (310 730.9, 637 231.3)	23.8 (15.4, 33.0)	654 183.6 (427 040.4, 909 865.0)	14.1 (9.1, 19.7)	–1.8 (–1.9, –1.7)
High SDI	74 166.7 (44 642.3, 106 675.2)	12.0 (7.2, 17.3)	60 753.2 (34 866.3, 89 347.0)	4.5 (2.7, 6.6)	–3.4 (–3.5, –3.3)
High-middle SDI	143 661.1 (94 094.8, 199 432.0)	29.8 (18.9, 42.0)	156 274.9 (97 856.4, 223 239.3)	14.4 (9.0, 20.7)	–2.6 (–2.9, –2.4)
Middle SDI	142 001.3 (97 077.5, 194 869.0)	29.7 (19.4, 41.7)	245 915.3 (161 636.5, 344 638.1)	18.0 (11.6, 25.6)	–1.5 (–1.7, –1.4)
Low-middle SDI	76 489.3 (52 949.1, 102 185.9)	26.2 (17.4, 35.7)	143 513.5 (96 648.2, 194 522.7)	20.2 (13.2, 27.8)	–0.9 (–0.9, –0.9)
Low SDI	28 922.7 (20 008.5, 38 848.9)	26.9 (17.9, 37.0)	47 132.7 (31 535.4, 65 010.8)	19.8 (12.8, 27.8)	–1.1 (–1.1, –1.0)
High-income Asia Pacific	16 928.3 (9940.7, 24 568.4)	16.8 (9.5, 24.7)	14 132.8 (7384.1, 21 788.9)	4.3 (2.4, 6.5)	–4.6 (–4.8, –4.4)
High-income North America	14 336.2 (8412.6, 20 764.9)	7.0 (4.1, 10.1)	18 218.7 (10 685.4, 26 654.3)	4.6 (2.7, 6.7)	–1.8 (–2.0, –1.5)
Western Europe	40 491.4 (23 456.4, 59 128.4)	11.9 (6.9, 17.4)	22 724.9 (12 568.5, 34 084.1)	3.4 (2.0, 5.0)	–4.2 (–4.4, –4.1)
Australasia	1115.5 (633.9, 1635.9)	8.9 (5.0, 13.2)	989.3 (527.7, 1501.9)	2.8 (1.5, 4.3)	–3.9 (–4.0, –3.8)
Andean Latin America	905.4 (584.6, 1275.4)	8.7 (5.5, 12.3)	1352.3 (830.3, 1977.9)	4.3 (2.6, 6.3)	–2.5 (–2.7, –2.3)
Tropical Latin America	10 105.7 (7057.4, 13 398.2)	23.0 (15.4, 31.1)	11 097.6 (7465.4, 15 020.1)	8.1 (5.4, 11.0)	–3.3 (–3.4, –3.2)
Central Latin America	5100.0 (3430.8, 6930.7)	12.9 (8.4, 17.8)	8674.2 (5695.9, 11 862.7)	6.6 (4.3, 9.1)	–2.4 (–2.6, –2.3)
Southern Latin America	2974.5 (1853.5, 4261.7)	12.3 (7.5, 17.8)	2298.8 (1392.5, 3358.3)	4.6 (2.8, 6.7)	–3.0 (–3.1, –2.9)
Caribbean	2063.9 (1377.5, 2826.5)	15.2 (10.0, 20.9)	3074.2 (1973.3, 4369.0)	10.4 (6.7, 14.8)	–1.2 (–1.3, –1.1)
Central Europe	21 002.9 (13 266.1, 29 406.3)	28.1 (17.3, 39.8)	15 929.3 (9453.3, 23 008.7)	11.9 (7.2, 17.2)	–3.2 (–3.4, –3.0)
Eastern Europe	53 035.0 (34 237.1, 73 724.0)	37.8 (23.9, 53.1)	41 690.8 (26 050.6, 58 786.7)	21.0 (13.2, 29.5)	–2.7 (–3.2, –2.2)
Central Asia	8222.9 (5679.1, 10 988.5)	34.7 (23.5, 46.8)	10 108.7 (6852.2, 13 679.7)	26.5 (17.4, 36.4)	–1.3 (–1.6, –1.0)
North Africa and Middle East	22 923.7 (15 143.4, 32 129.8)	29.7 (18.8, 42.6)	37 178.2 (23 400.5, 52 432.4)	17.8 (10.8, 25.5)	–1.7 (–1.8, –1.6)
South Asia	55 335.6 (37 853.4, 74 755.0)	20.5 (13.4, 28.3)	112 718.9 (75 314.5, 155 447.1)	15.5 (10.0, 21.6)	–1.0 (–1.1, –0.9)
Southeast Asia	49 978.2 (34 693.3, 67 101.7)	40.4 (27.3, 55.1)	106 464.4 (71 928.0, 144 696.6)	32.6 (21.4, 44.9)	–0.7 (–0.8, –0.5)
East Asia	131 011.3 (86 727.1, 186 174.5)	34.2 (21.3, 50.0)	196 467.6 (122 181.3, 287 418.9)	17.6 (10.7, 25.9)	–1.9 (–2.2, –1.6)
Oceania	453.6 (300.3, 644.4)	33.5 (21.3, 48.3)	943.9 (620.4, 1359.1)	27.2 (17.3, 39.5)	–0.7 (–0.7, –0.7)
Western Sub-Saharan Africa	14 379.6 (9835.7, 19 363.2)	34.3 (22.7, 47.1)	22 462.7 (15 105.2, 30 938.5)	25.2 (16.4, 35.1)	–1.1 (–1.1, –1.0)
Eastern Sub-Saharan Africa	8920.4 (5903.0, 12 461.3)	24.7 (15.8, 35.3)	13 629.5 (8697.6, 19 504.0)	17.3 (10.6, 25.2)	–1.3 (–1.4, –1.2)
Central Sub-Saharan Africa	3701.6 (2398.2, 5199.7)	36.9 (22.8, 52.9)	7203.9 (4439.4, 10 651.1)	30.7 (18.1, 46.4)	–0.8 (–0.8, –0.7)
Southern Sub-Saharan Africa	2815.7 (1896.3, 3893.3)	21.2 (13.9, 29.7)	6823.1 (4738.8, 9189.1)	25.1 (16.8, 34.4)	0.6 (0.2, 1.1)
DALYs					
Global	10 843 730.1 (7 638 187.5, 14 470 967.6)	506.2 (349.7, 681.9)	14 820 610.7 (10 194 050.9, 20 051 858.6)	311.7 (213.0, 423.3)	–1.7 (–1.8, –1.6)
High SDI	1 414 228.6 (923 328.7, 1 958 743.8)	229.2 (150.1, 317.2)	1 177 818.2 (746 120.0, 1 668 188.7)	98.9 (64.5, 138.3)	–3.0 (–3.1, –2.8)
High-middle SDI	3 112 699.8 (2 155 593.9, 4 219 088.5)	589.2 (399.6, 806.4)	3 200 182.0 (2 140 116.7, 4 443 800.9)	293.7 (195.6, 408.8)	–2.5 (–2.8, –2.3)
Middle SDI	3 544 761.7 (2 528 441.6, 4 750 363.7)	637.5 (441.8, 867.9)	5 695 536.9 (3 925 755.8, 7 758 764.6)	387.1 (262.4, 532.3)	–1.5 (–1.6, –1.4)
Low-middle SDI	1 990 307.1 (1 425 036.9, 2 616 541.9)	592.4 (414.5, 787.6)	3 513 941.4 (2 462 730.8, 4 681 524.0)	445.4 (306.6, 598.5)	–1.0 (–1.0, –0.9)
Low SDI	769 661.0 (547 163.7, 1 018 432.9)	616.3 (428.3, 826.2)	1 220 212.3 (843 908.3, 1 658 461.8)	439.2 (297.3, 602.8)	–1.2 (–1.3, –1.2)
High-income Asia Pacific	355 359.7 (229 416.5, 498 471.8)	329.5 (208.2, 467.1)	269 212.7 (159 436.4, 398 493.6)	100.9 (63.6, 145.6)	–4.0 (–4.2, –3.9)
High-income North America	277 847.2 (177 609.2, 388 142.6)	139.0 (89.6, 193.4)	368 574.0 (237 612.1, 516 669.7)	99.7 (65.2, 139.0)	–1.4 (–1.6, –1.2)
Western Europe	672 103.1 (422 339.1, 948 835.8)	200.7 (127.5, 282.0)	368 212.4 (223 101.4, 533 918.3)	62.8 (39.9, 89.2)	–3.9 (–4.1, –3.8)
Australasia	19 520.5 (12 065.5, 27 705.0)	150.3 (91.5, 214.8)	16 683.8 (9820.5, 24 477.3)	51.6 (31.1, 75.1)	–3.7 (–3.8, –3.5)
Andean Latin America	22 172.9 (15 017.7, 30 582.8)	193.5 (129.5, 268.2)	30 421.7 (19 849.2, 43 246.0)	93.4 (60.6, 133.1)	–2.6 (–2.8, –2.4)
Tropical Latin America	244 124.3 (179 433.7, 315 273.1)	489.2 (350.6, 640.2)	244 118.7 (175 613.1, 318 925.9)	172.7 (123.1, 226.8)	–3.5 (–3.5, –3.4)
Central Latin America	117 383.7 (83 485.7, 155 236.9)	263.7 (184.0, 351.8)	193 576.1 (134 729.7, 257 718.0)	141.5 (97.5, 189.3)	–2.4 (–2.5, –2.2)
Southern Latin America	65 475.3 (43 699.0, 90 997.9)	259.6 (171.1, 363.0)	47 807.6 (31 068.5, 67 738.6)	98.4 (64.4, 139.0)	–3.0 (–3.1, –2.9)
Caribbean	47 486.0 (33 110.6, 63 486.8)	331.9 (230.0, 445.2)	69 016.5 (46 569.2, 96 170.1)	233.3 (157.2, 325.5)	–1.1 (–1.2, –1.0)
Central Europe	415 659.8 (282 512.7, 563 541.0)	521.2 (347.9, 712.7)	282 363.6 (181 682.2, 393 990.3)	222.0 (144.9, 307.9)	–3.3 (–3.5, –3.1)
Eastern Europe	1 084 380.2 (740 743.4, 1 467 343.4)	723.9 (488.0, 985.7)	823 587.6 (550 659.6, 1130 364.0)	425.5 (285.5, 583.4)	–2.5 (–3.0, –2.0)
Central Asia	199 361.1 (146 733.1, 256 850.9)	782.6 (568.1, 1015.7)	245 113.0 (177 134.7, 320 473.7)	566.3 (398.2, 750.9)	–1.5 (–1.9, –1.2)
North Africa and Middle East	566 459.6 (390 385.8, 773 057.2)	626.4 (419.4, 868.6)	886 903.8 (590 669.2, 121 5281.1)	365.4 (236.3, 506.8)	–1.8 (–1.9, –1.8)
South Asia	1 460 385.1 (1 034 766.2, 1 945 452.8)	462.7 (319.2, 623.8)	2 722 640.5 (1 898 204.0, 3 668 177.8)	337.0 (230.4, 458.1)	–1.1 (–1.2, –1.1)
Southeast Asia	1 338 042.8 (954 159.8, 1 771 235.6)	938.6 (658.6, 1252.4)	2 727 644.8 (1 901 958.5, 3 652 656.8)	745.6 (511.2, 1006.7)	–0.7 (–0.8, –0.6)
East Asia	3 166 460.4 (2 192 325.4, 4 392 279.3)	697.1 (464.1, 985.8)	4 198 687.3 (2 756 171.6, 5 984 316.6)	352.8 (227.9, 506.8)	–1.9 (–2.2, –1.7)
Oceania	12 916.7 (8691.0, 18 286.0)	779.3 (513.4, 1108.0)	26 427.1 (17 753.3, 37 769.3)	625.9 (413.1, 896.2)	–0.7 (–0.8, –0.7)
Western Sub-Saharan Africa	361 446.5 (255 158.9, 477 141.0)	756.7 (523.4, 1009.8)	577 648.5 (402 379.1, 782 336.0)	543.6 (370.9, 741.8)	–1.1 (–1.2, –1.1)
Eastern Sub-Saharan Africa	242 769.8 (164 618.6, 334 659.1)	578.4 (384.2, 806.2)	363 960.3 (239 740.4, 512 623.4)	389.9 (251.5, 554.5)	–1.4 (–1.5, –1.4)
Central Sub-Saharan Africa	99 936.4 (66 555.3, 139 210.3)	825.6 (535.3, 1161.4)	190 717.4 (122 199.7, 276 515.4)	662.4 (412.6, 971.4)	–0.9 (–1.0, –0.8)
Southern Sub-Saharan Africa	74 439.2 (52 597.4, 100 022.5)	493.3 (342.3, 668.8)	167 293.2 (121 469.4, 220 217.9)	539.2 (382.3, 718.1)	0.4 (–0.1, 0.9)

ASR, age-standardized rates.

For KD-related IS, the mortality cases has risen 45.9% from 225 704.3 (95% UI 43 330.6 to 317 278.7) in 1990 to 329 237.4 (95% UI 205 507.5 to 467 377.9) in 2021, with an EAPC of –1.9 (95% CI –2.0 to –1.8); the number of DALYs has also risen 47.4% from 4 619 125.3 (95% UI 3 138 719.8 to 6 312 514.5) in 1990 to 6 808 070.0 (95% UI 4 519 000.4 to 9 385 703.3) in 2021, with an EAPC of –1.7 (95% CI –1.7 to –1.6) (Supplementary data, Table S1, Fig. S1).

For KD-related ICH, the mortality cases has risen 35.3% from 240 096.8 (95% UI 166 883.0 to 322 027.6) in 1990 to 324 946.2 (95% UI 220 387.6 to 443 171.6) in 2021, with an EAPC of –1.7 (95% CI –1.8 to –1.5); the number of DALYs has also risen 28.7% from 6 224 604.8 (95% UI 4 483 293.4 to 8 204 935.0) in 1990 to 8 012 540.7 (95% UI 5 656 124.7 to 1 0694 840.6) in 2021, with an EAPC of –1.7 (95% CI –1.8 to –1.6) (Supplementary data, Table S2, Fig. S2).

### SDI regional-level trends

For KD-related stroke from 1990 to 2021, the middle SDI area was the epicentre of the burden in 2021, recording the highest absolute cases in mortality (245 915.3; 95% UI 161 636.5 to 344 638.1) and DALYs (5 695 536.7; 95% UI 3 925 755.8 to 7 758 764.6). Compared with 1990, when mortality was 142 001.3 (95% UI 97 077.5 to 194 869.0) and DALYs were 3 544 761.7 (95% UI 2 528 441.6 to 4 750 363.7), both metrics have shown a significant increase over the past three decades. However, the low-middle SDI region showed the lowest decrease in EAPCs of mortality (–0.9; 95% CI –0.9 to –0.9) and DALYs (–1.0; 95% CI –1.0 to –0.9) (Table 1, Fig. 1).

For KD-related IS, the middle SDI area was the epicentre of the burden in 2021, recording the highest absolute cases in mortality (110 754.8; 95% UI 70 725.3 to 157 781.3) and DALYs (2 395 986.7; 95% UI 1 609 493.4 to 3 313 227.7). Compared with 1990, when mortality was 51 340.4 (95% UI 34 056.8 to 71 420.0) and DALYs were 1 178 095.3 (95% UI 824 574.4 to 1 602 997.7), both metrics have shown a significant increase over the past three decades. In contrast, the low SDI region experienced lowest downward trend with EAPCs of mortality (–0.5; 95% CI –0.5 to –0.5) and DALYs (–0.6; 95% CI –0.7 to –0.6) (Supplementary data, Table S1, Fig. S1).

For KD-related ICH, the middle SDI area was the epicentre of the burden in 2021, recording the highest absolute cases in mortality (135 160.7; 95% UI 90 549.5 to 187 320.3) and DALYs (3 299 550.2; 95% UI 2 310 202.4 to 4 462 477.8). Compared with 1990, when mortality was 90 660.9 (95% UI 62 840.2 to 123 906.3) and DALYs were 2 366 666.4 (95% UI 1 697 290.6 to 3 171 630.5), both metrics have shown a significant increase over the past three decades. The low-middle SDI region showed the lowest decrease with EAPCs of mortality (–1.3; 95% CI –1.3 to –1.2) and DALYs (–1.3; 95% CI –1.3 to –1.2) (Supplementary data, Table S2, Fig. S2).

### GBD regional-level trends

In KD-related stroke, from 1990 to 2021, East Asia was the epicentre of the burden in 2021, recording the highest absolute cases in mortality (196 467.6; 95% UI 122 181.3 to 287 418.9) and DALYs (4 198 687.3; 95% UI 2 756 171.6 to 5 984 316.6). Compared with 1990, when mortality was 131 011.3 (95% UI 86 727.1 to 186 174.5) and DALYs were 3 166 460.4 (95% UI 2 192 325.4 to 4 392 279.3), both metrics have shown a significant increase over the past three decades. Among the 21 areas analysed, 17 exhibited an increase in absolute mortality and DALYs over time. However, certain high or middle SDI areas, including Eastern Europe, high-income Asia Pacific, Western Europe and Southern Latin America, showed decreases. Despite this trend, Southern sub-Saharan Africa experienced the highest increase with EAPCs of mortality (0.6; 95% CI 0.2 to 1.1) and DALYs (0.4; 95% CI –0.1 to 0.9) (Table 1).

In KD-related IS, from 1990 to 2021, East Asia was the epicentre of the burden in 2021, recording the highest absolute cases in mortality (90 406.6; 95% UI 54 362.4 to 134 114.1) and DALYs (1 906 611.5; 95% UI 1 226 043.8 to 2 751 838.6). Compared with 1990, when mortality was 41 713.1 (95% UI 26 798.5 to 59 834.7) and DALYs were 978 804.1 (95% UI 662 920.5 to 1 371 681.2), both metrics have shown a significant increase over the past three decades. Among the 21 areas analysed, 16 exhibited an increase in absolute mortality and DALYs over time. However, certain high or middle SDI areas, including Australasia, Central Europe, Eastern Europe, Western Europe and Southern Latin America, showed decreases. Despite this trend, Southern sub-Saharan Africa experienced the highest increase with EAPCs of mortality (1.0; 95% CI 0.5 to 1.5) and DALYs (0.7; 95% CI 0.3 to 1.1) (Supplementary data, Table S1).

For KD-related ICH from 1990 to 2021, East Asia was the epicentre of the burden in 2021, recording the highest absolute cases of mortality (106 061.0; 95% UI 66 871.6 to 154 721.8), and DALYs (2 292 075.9; 95% UI 1 516 157.3 to 3 264 405.7). Compared with 1990, when mortality was 89 298.2 (95% UI 59 685.9 to 126 544.1) and DALYs were 2 187 656.4 (95% UI 1 519 615.5 to 3 036 006.5), both metrics have shown a significant increase over the past three decades. Among the 21 areas analysed, 15 exhibited an increase in absolute mortality and DALYs over time. However, certain high or middle SDI areas, including Central Europe, Eastern Europe, high-income Asia Pacific, Tropical Latin America, Western Europe and Southern Latin America, showed a decrease. Southern sub-Saharan Africa recorded the highest increase with EAPCs of mortality (0.3; 95% CI –0.2 to 0.8) and DALYs (0.2; 95% CI –0.3 to 0.7) (Supplementary data, Table S2).

Supplementary data, Figs S2–S8 demonstrate positive correlations between age-standardized mortality rates, age-standardized DALY rates and the SDI for ICH. No correlations were observed between age-standardized mortality rates, age-standardized DALY rates and the SDI in stroke and IS.

### National/territorial level trends

**Figure 2:**
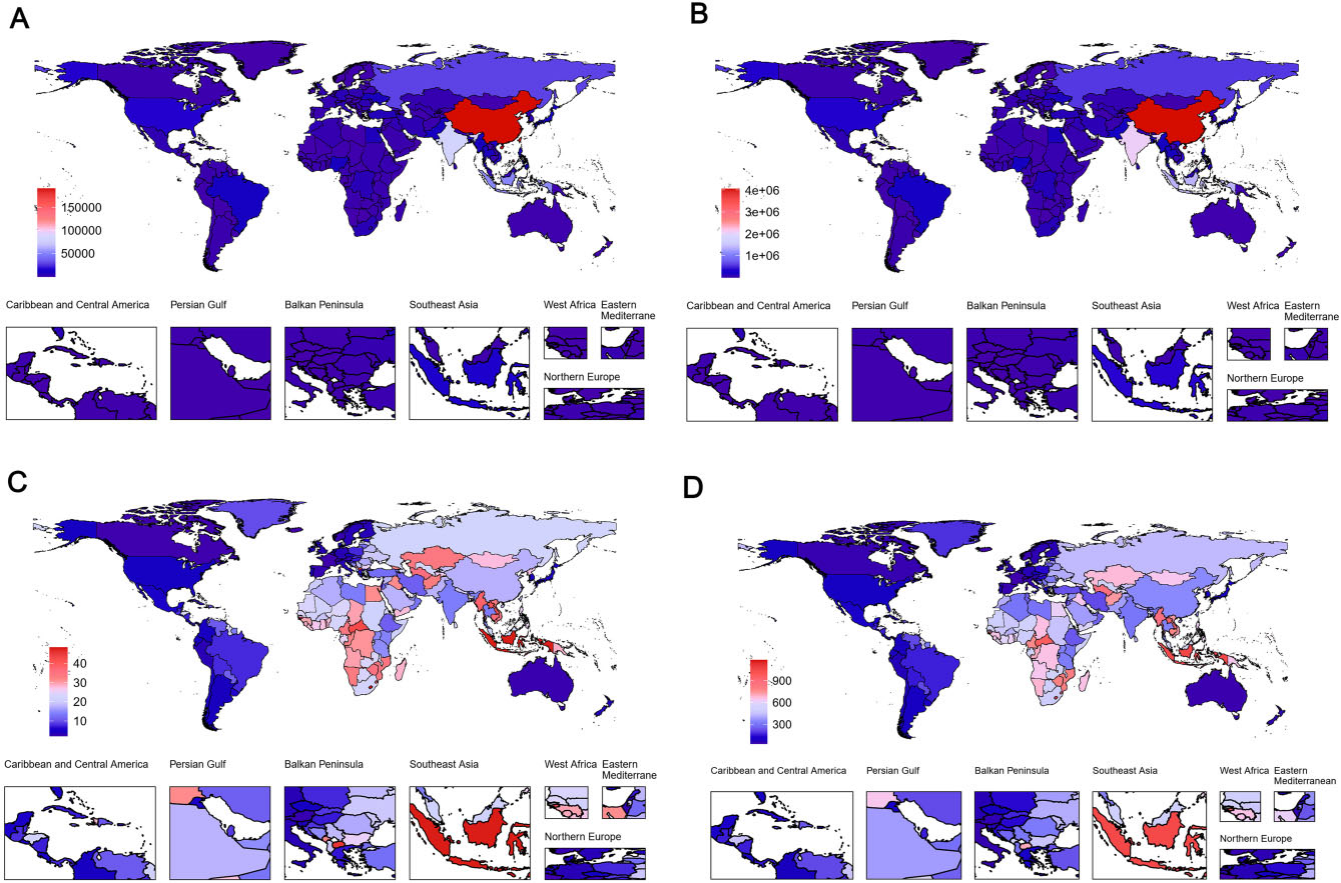
(**A**) The global disease burden of KD-related stroke mortality number in 204 countries and territories; (**B**) the global disease burden of KD-related stroke DALYs number in 204 countries and territories; (**C**) the global disease burden of KD-related stroke mortality rate in 204 countries and territories; (**D**) the global disease burden of KD-related stroke DALYs rate in 204 countries and territories.

In KD-related stroke, from 1990 to 2021, China was the epicentre of the burden in 2021, with the highest absolute cases in mortality (88 360.0; 95% UI 53 027.6 to 131 237.3) and DALYs (1 856 478.9; 95% UI 1 189 805.2 to 2 684 735.8). Compared with 1990, when mortality was 40 386.7 (95% UI 25 903.1 to 58 046.0) and DALYs were 946 137.4 (95% UI 639 249.4 to 1 328 441.3), both metrics have shown a significant increase over the past three decades. Conversely, Tokelau reported the lowest absolute cases in mortality (0.1; 95% UI 0.0 to 0.1) and DALYs (1.3; 95% UI 0.8 to 1.9). Compared with 1990, when mortality was 0.1 (95% UI 0.0 to 0.1) and DALYs were 1.5 (95% UI 0.9 to 2.2), both metrics have shown a significant decrease over the past three decades. Lesotho experienced the highest increase with EAPCs of mortality (2.6; 95% CI 2.1 to 3.1) and DALYs (2.5; 95% CI 2.1 to 2.9). In contrast, Estonia reported the highest decrease with EAPCs of mortality (–7.0; 95% UI –7.6 to –6.4), whereas the Republic of Korea reported the highest decrease with EAPCs of DALYs (–7.7; 95% UI –8.0 to –7.4) (Fig. 2, and Supplementary data, Table S3, Figs S9–S10).

In KD-related IS, from 1990 to 2021, China was the epicentre of the burden in 2021, recording the highest absolute cases in mortality (102 353.6; 95% UI 64 131.5 to 149 801.2) and DALYs (2 200 339.0; 95% UI 1 445 244.0 to 3 147 436.7). Compared with 1990, when mortality was 86 599.4 (95% UI 57 748.0 to 123 205.4) and DALYs were 21 182 42.3 (95% UI 1 467 152.6 to 2 953 532.4), both metrics have shown a significant increase over the past three decades. Tokelau reported the lowest absolute cases in mortality (0.1; 95% UI 0.1 to 0.2) and DALYs (2.9; 95% UI 1.8 to 4.3). Compared with 1990, when mortality was 0.2 (95% UI 0.1 to 0.3) and DALYs were 4.4 (95% UI 2.8 to 6.6), both metrics have shown a significant decrease over the past three decades. Lesotho experienced the highest increase with EAPCs of mortality (2.1; 95% CI 1.6 to 2.6) and DALYs (2.3; 95% CI 1.8 to 2.9). Conversely, Singapore reported the highest decrease with EAPCs of mortality (–5.5; 95% UI –5.7 to –5.3) and Portugal reported the highest decrease with EAPCs of DALYs (–5.5; 95% UI –5.6 to –5.3) (Supplementary data, Table S1, Figs S11–S13).

In KD-related ICH, from 1990 to 2021, China was the epicentre of the burden in 2021, recording the highest absolute cases of mortality (190 713.6; 95% UI 118 059.8 to 279 641.1) and DALYs (4 056 817.9; 95% UI 2 650 272.2 to 5 790 180.8). Compared with 1990, when mortality was 126 986.1 (95% UI 83 774.2 to 181 101.8) and DALYs were 3 064 379.6 (95% UI 2 114 240.5 to 4 267 935.1), both metrics have shown a significant increase over the past three decades. In contrast, Tokelau reported the lowest absolute cases in mortality (0.2; 95% UI 0.1 to 0.3) and DALYs (4.2; 95% UI 2.7 to 6.2). Compared with 1990, when mortality was 0.3 (95% UI 0.2 to 0.4) and DALYs were 5.9 (95% UI 3.8 to 8.7), both metrics have shown a significant decrease over the past three decades. Zimbabwe experienced the highest increase with EAPCs of mortality (2.0; 95% CI 1.4 to 2.6) and DALYs (2.1; 95% CI 1.5 to 2.8). The Republic of Korea reported the highest decrease with EAPCs of mortality (–6.7; 95% UI –6.9 to –6.4) and DALYs (–6.6; 95% UI –6.8 to –6.3) (Supplementary data, Table S2, Figs S14–S16).

Supplementary data, Figs S17–S22 demonstrate negative correlations between age-standardized mortality rates, age-standardized DALY rates, and the SDI in the SSS.

### Gender and age patterns

From 1990 to 2021, the global KD-related SSS highlighted distinct disparities in age- and sex-related patterns. It revealed that the mortality and DALY rates were increased with age in stroke and IS. Mortality and DALY rates associated with KD-related stroke and IS showed a progressive increase with age, reaching their peak in individuals aged 90–94 years. Similarly, rates associated with KD-related ICH attained a zenith in individuals aged 90–94 years. Furthermore, a notable disparity was observed in the burden between males and females, with males experiencing a significantly greater burden in age-standardized rates of mortality and DALYs (Supplementary data, Table S4–S6, Figs S23–S34).

### Decomposition analysis from 1990 to 2021 according to SDI and 21 GBD regions

**Figure 3:**
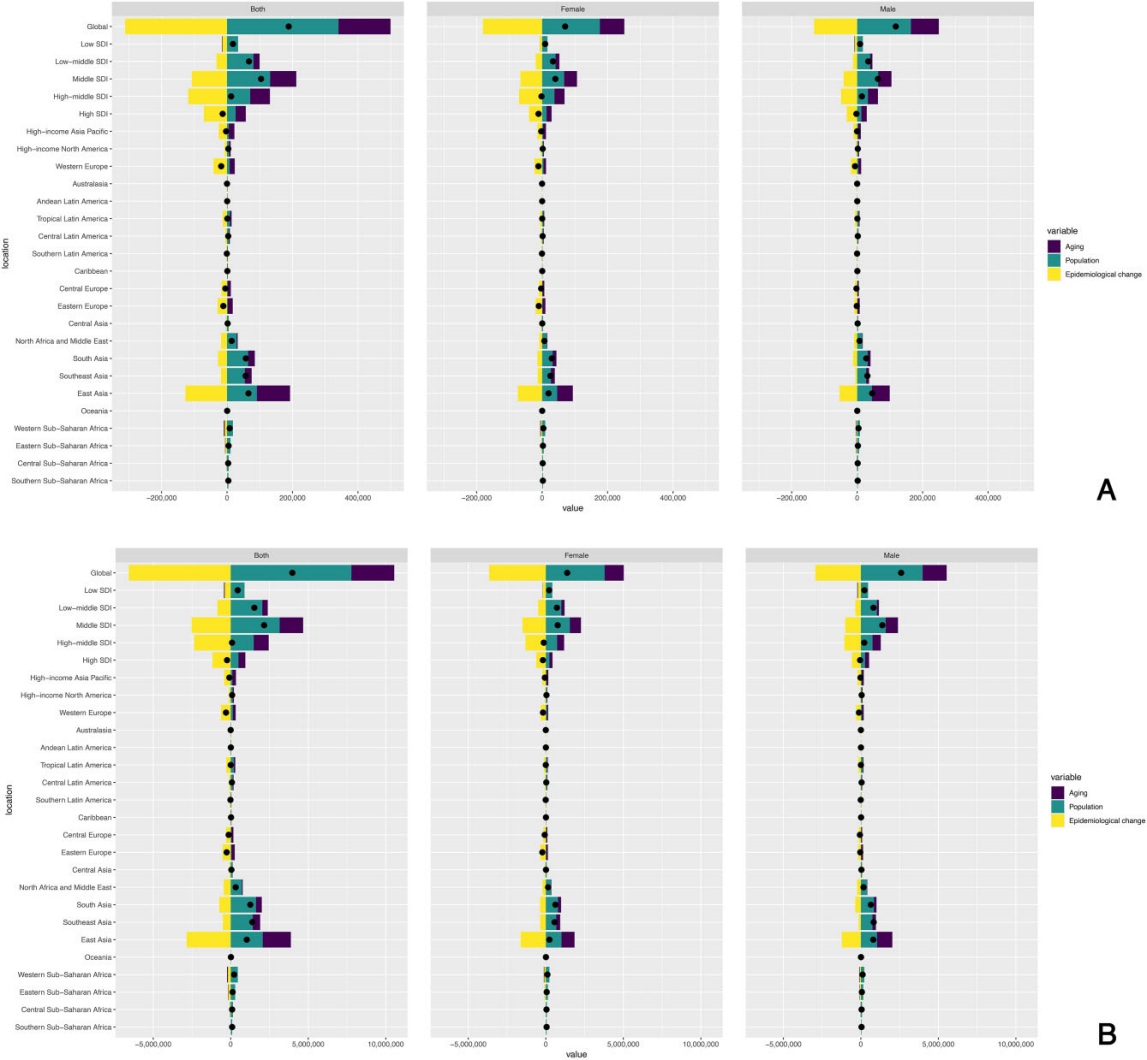
(**A**) Decomposition analysis of KD-related stroke burden change in mortality by SDI and 21 GBD region, 1990 to 2021. Black dots represent the total change contributed by all three components. A positive value for each component indicates a corresponding positive contribution in mortality, and a negative value indicates a corresponding negative contribution in mortality. (**B**) Decomposition analysis of KD-related stroke burden change in DALYs by SDI and 21 GBD region, 1990 to 2021. Black dots represent the total change contributed by all three components. A positive value for each component indicates a corresponding positive contribution in DALYs, and a negative value indicates a corresponding negative contribution in DALYs.

Globally, total KD-related mortality and DALYs burden increased from 1990 to 2021, with population growth and aging contributing positively and epidemiological changes contributing negatively. Excluding high SDI and high-middle SDI areas, the other three SDI areas showed an increase in total KD-related SSS-related mortality and DALYs from 1990 to 2021, largely attributed to population growth. Furthermore, in the middle SDI region, both sexes recorded the greatest increases in mortality and DALYs, driven mainly by population growth, followed by aging. Among the GBD areas, East Asia, Southeast Asia and South Asia showed increased total KD-related, SSS-related mortality and DALYs from 1990 to 2021, primarily attributed to population growth. In East Asia, both sexes recorded the greatest increases in mortality and DALYs, driven primarily by population growth and aging (Fig. 3, and Supplementary data, Figs S35–S36).

### Future burden

**Figure 4:**
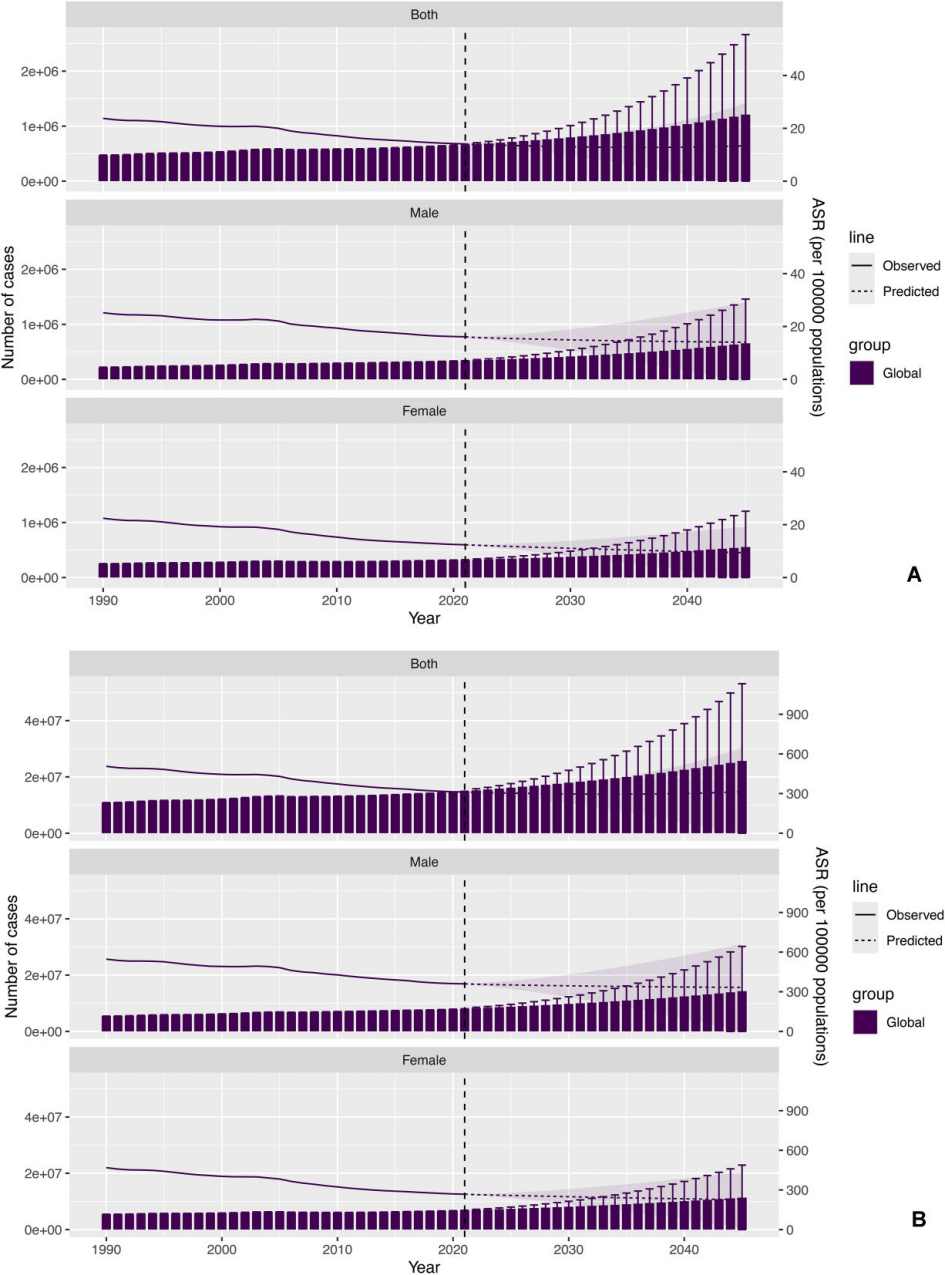
(**A**) Future forecasts of GBD in KD-related stroke mortality using BAPC model; (**B**) future forecasts of GBD in KD-related stroke disability-adjusted life years using BAPC model. ASR, age-standardized rates. The line graphs show the change of ASR, and the bar graphs show the change of burden.

Figure 4, and Supplementary data, Tables S7–S9, Figs S37–S38 show the trends in KD-related SSS from 1990 to 2045 using using BAPC model. The graphs illuminate a persistent upward trend in the count of individuals impacted by KD-associated SSS worldwide, with age-standardized rates showing a downward trend in mortality and DALYs. For KD-related stroke, the expected number of mortality cases (1 210 748.0; 95% UI 0.0 to 2 665 434.2) and DALYs (2 5678 609.5; 95% UI 0.0 to 53 108 368.9) is projected to rise globally by 2045, whereas the age-standardized rates in mortality, and DALYs are projected to decline. For KD-related IS, the expected number of mortality cases (721 094.6; 95% UI 0.0 to 1 780 305.3) and DALYs (13 954 991.2; 95% UI 0.0 to 31 252 261.0) is expected to rise globally by 2045. However, the age-standardized rates in mortality and DALYs are expected to decline. For KD-related ICH, the expected number of mortality cases (560 564.2; 95% UI 0.0 to 1 249 349.6) and DALYs (12 972 006.5; 95% UI 0.0 to 27 851 633.4) is projected to increase globally by 2045, whereas the age-standardized rates in mortality, and DALYs are projected to show a downward trend. Males consistently demonstrated higher rates than females. Sensitivity analyses using the nordpred model confirmed the robustness of the results of BAPC model (Supplementary data, Table S10–S12, Figs S39–S41).

## DISCUSSION

KD, an important worldwide public health issue, carries a substantial risk of stroke morbidity and mortality. Studies have demonstrated an independent graded association between renal function and stroke events [30, 31]. However, no systematic analyses have explored whether KD affects the disease burden of SSS. Based on the GBD 2021 dataset, this study offers novel epidemiological insights into the burden on individuals with KD-related SSS. We applied sophisticated statistical methodologies, including the EAPC and decomposition methods. Additionally, our study utilizes the BAPC method to predict future trends, providing GBD evidence-based insights for public health programs and resource allocation. Thus, using above methods, the study confirmed that first, global mortality and DALYs burden associated with KD-related SSS significantly increased; in addition, the highest mortality and age-standardized DALY rates observed in older age groups. In addition, the study found several unexpected findings that provide new insights into the global burden of KD-related stroke: first, KD-related SSS burden disproportionately affected males more than females; second, the upward trend in total KD-related SSS mortality and DALYs was primarily driven by population growth and aging, while epidemiological changes contributed negatively to this trend; in addition, a positive association was observed between the SDI and the burden of ICH, with higher SDI regions exhibiting reduced mortality rates and DALYs rates from KD-related ICH; finally, the burden of KD-related SSS is projected to gradually increase until 2045, particularly among male individuals.

Between 1990 and 2021, global mortality burden associated with KD-related SSS significantly increased. By 2021, these increasing rates were 40.4%, 45.9% and 35.3%, respectively, compared with 1990 levels. Similarly, the global DALYs associated with stroke, IS and KD-related ICH were increased by 36.7%, 47.4% and 28.7%, respectively, in 2021 compared with the figures recorded in 1990. Specific regions disproportionately shoulder the burden, with the middle SDI area and East Asia (GBD region) experiencing a huge burden on KD-related SSS mortality and DALYs. Additionally, our study results revealed wide variations between countries/territories, with China emerging as the epicentre of burden in KD-related SSS mortality and DALYs. These areas experience major transformations in living circumstances, work settings, social frameworks, population growth and aging, which contribute to altered lifestyles and population structures [32]. In addition, these regional disparities in disease burden and trends are likely attributable to variations in multiple factors, including socioeconomic status, healthcare infrastructure, environmental exposures, lifestyle patterns, genetic predispositions, biological differences, access to quality kidney dysfunction services and healthcare-seeking behaviours [33–36]. Such changes may represent major risk factors for KD-related SSS.

Nevertheless, the EAPC of KD-related SSS mortality and DALYs showed a decreasing trend between 1990 and 2021. By 2021, the EAPCs of mortality declined to –1.8, –1.9 and –1.7 for stroke, IS, and ICH, respectively, in comparison with the levels recorded in 1990. Similarly, the global DALYs associated with stroke, IS and KD-related ICH significantly decreased in 2021. By 2021, the EAPCs of mortality declined to –1.7, –1.7 and –1.7, respectively, in comparison with the levels recorded in 1990. Up-to-now, this represents a further understanding of the pathogenesis, risk factors and biomarkers of KD-related SSS. However, specific regions changed disproportionately. The low-middle SDI regions experienced the smallest decreases in trends related to KD-associated SSS mortality and DALYs, whereas Southern sub-Saharan Africa (GBD region) demonstrated the highest increase. Furthermore, our findings revealed huge variations between countries/territories. Lesotho reported the highest increase in EAPCs of mortality and DALYs in KD-related stroke and IS, whereas Zimbabwe recorded the highest increase in EAPCs of mortality and DALYs in KD-related ICH. Owing to its complexity and high cost, KD care is closely associated with the economic development and healthcare resources of each nation. In Southern sub-Saharan Africa, such as Lesotho and Zimbabwe, the lowest decrease in KD-related SSS burden might be associated with the sluggish rate of expansion in conjunction with economic progress [32, 37].

Our findings demonstrate a complex link between KD-related SSS burden and SDI. The inconsistency in the KD-related SSS burden among SDI areas and nations stresses the relevance of personalized therapies and public health tactics. According to the study, there is a positive association between SDI and ICH burden, with areas of higher SDI showing reduced mortality and DALYs from KD-related ICH. This correlation can be traced back to socioeconomic factors, which is related to the large population base, insufficient medical resources, uneven distribution of medical resources, and relatively backward medical technology. This change may lead to increased metabolic risk, behavioural risk, environmental and air pollution, and exposure to environmental pollutants, which are risk factors for ICH [32]. Additionally, KD-related SSS disproportionately affects males more than females across various population groups and regions; this pattern is expected to continue until 2045. This finding is consistent with previous research [32, 37–39], indicating that hormonal factors, particularly male hormone levels, may significantly contribute to the increased mortality and DALYs associated with KD-related SSS in males. Male hormones exacerbate KD progression through increased oxidative stress, the promotion of fibrosis and the activation of the renin–angiotensin–aldosterone system [40, 41]. The disparities in how males and females access healthcare and seek treatment may contribute to the differences observed in the KD-related SSS burden. Women are more likely to seek care earlier than men, which could lead to premature prevention. Moreover, women usually adopt healthier lifestyles than men [42]. Our study also showed that the burden of KD-related SSS increases with age, with the highest mortality and age-standardized DALY rates observed in older age groups. Our finding aligns with the well-established association between aging and stroke risk [32, 43, 44]. The heightened burden observed in the older population may be attributed to the cumulative effects of the KD over time combined with age-related changes in arterial stiffness and endothelial dysfunction [45].

We utilized decomposition analysis to effectively differentiate the effects of population growth, aging and epidemiological changes. The analysis revealed an upward trend in total KD-related SSS mortality and DALYs from 1990 to 2021. This upward trend was primarily attributed to population growth and aging, whereas epidemiological changes contributed negatively. Notably, East Asia, Southeast Asia and South Asia reported an increased burden from 1990 to 2021, which is attributed to population growth and aging. Population growth and aging have led to more severe outcomes owing to the high social and economic costs associated with the disease. This massive burden on the public health system may persist as aging progresses [46, 47]. Therefore, regularly monitoring the KD of the aging population and controlling population growth is recommended. Notably, in the high and high-middle SDI areas, the total burden reduced from 1990 to 2021. This decline was mainly caused by changes in epidemiological patterns. This discovery requires more research because it goes against the general trend of a rising burden of KD-related SSS. The reduction in high and high-middle SDI regions can be linked to swift economic growth and improvements in medical care frameworks, leading to improved access to healthcare, increased medical awareness, and better diagnostic capabilities [32]. Further studies are needed to explore the underlying mechanisms causing these regional declines and to assess whether the strategies used in these areas can be implemented in other locations to lessen the worldwide impact on KD-related SSS patients.

The BAPC analysis offered new perspectives on the time-related patterns of KD-related SSS burden. Figure 4, and Figs S37-38 illustrates a gradual global increase in the count of individuals experiencing KD-related SSS through 2045. Multifaceted strategies are required to address the increasing burden of KD-related SSS in adults. Public health initiatives have focused on increasing awareness of KD-related SSS, and management techniques can assist individuals to understand and effectively manage their condition [7, 48]. These initiatives should target healthcare providers and policymakers as well as the general public to ensure a comprehensive and coordinated approach. It is essential for medical care frameworks to emphasize training professionals in the precise diagnosis and treatment of KD-related SSS, especially in regions where the condition is underrecognized [49]. Furthermore, providing telemedicine services, regular KD tests, and standardized treatment can prevent SSS and reduce mortality and DALY rates, especially in people at high risk and in the early stages of KD [50, 51], especially in lacking adequate medical service areas. Furthermore, studies should aim to identify novel therapeutic treatments for KD and create more impactful and available ways for KD-related SSS. Advances in individualized medicine and biomarker utilization can enhance KD-related SSS treatment plans and improve patient outcomes.

To our knowledge, this study is the first, most recent, and comprehensive assessment to investigate the global burden of KD-related SSS using the 2021 GBD study, revealing novel evidence-based insights. In addition, our findings highlight the distinct challenges faced by individuals with KD-related SSS, underscoring the critical need for KD interventions and therapies targeting the kidney–brain axis. Such treatments play a key role in mitigating the prolonged impact of this life-threatening issue and enhancing the well-being of this vulnerable demographic. To address data quality concerns, we used uncertainty quantification (95% UIs) and sensitivity analyses, ensuring reliable estimates while minimizing data quality impacts. Moreover, the disparities in KD-related SSS mortality and DALYs across various SDI and GBD regions, countries/territories, age cohorts and sexes indicate the need for customized strategies to effectively successfully manage the distinct issues and demands of every demographic category. Additionally, limited data availability poses challenges in assessing the impact of key factors on the burden of KD-related SSS and capturing trends over time. Future research should incorporate additional variables (e.g. genetic markers, environmental factors), conduct longitudinal analyses, and explore advanced statistical techniques such as machine learning algorithms or causal inference methods to uncover complex interactions and better understand temporal dynamics of KD-related SSS burden and trends [52, 53]. This study forms the basis for subsequent research and policymaking initiatives aimed at enhancing the understanding and management of KD-related SSS in this vulnerable demographic region.

However, this study has some limitations. First, variations in data quality and missing data across regions and countries may have affected the accuracy of the estimates. In certain low and middle-income nations/territories, the lack of reliable epidemiological data and underreporting of SSS cases may cause an underestimation of the true burden of the disorder. Thus, we preformed the uncertainty quantification using 95% UIs to transparently communicate estimate precision and conducted sensitivity analyses to test the robustness of our results. Second, due to the data limitations of the GBD database, the study only covered data on people aged 25–94 years with stroke and subtypes (IS and ICH) attributable to KD, excluding those aged <25 years with stroke and subtype (IS and ICH) data and SAH data. Third, this research depended exclusively on the GBD database, excluding other potential datasets, such as the WHO's Global Health Estimates, United Nations Population Division, World Bank Health Nutrition and Population Data. Fourth, kidney dysfunction was identified as the eighth risk factor to stroke, since this study only explored kidney dysfunction as a risk factor for stroke mortality and disability, it did not assess its relative contribution compared with other risk factors [54]. Finally, the GBD methodology relies on a range of assumptions and modelling approaches, potentially introducing several uncertainties into the estimates. Despite using robust statistical approaches to address these uncertainties, the findings are the most reliable estimates based on currently accessible evidence.

## CONCLUSIONS

This study provides a comprehensive analysis of the global patterns of mortality and DALYs of KD-related SSS from 1990 to 2021. Although age-standardized rates have decreased, the absolute burden remains substantial, with notable differences across the globe, including among the 5 SDI quintiles, 21 GBD regions, 204 nations and territories, and age groups and sexes. These findings emphasize the importance of developing tailored prevention and targeted treatment strategies that specifically address KD, particularly in areas and populations disproportionately affected by KD-related SSS.

## Supplementary Material

sfaf160_Supplemental_Files

## Data Availability

Data will be made available on request.
