# Supplementary material for: Global burden and trends of stroke attributable to kidney dysfunction from 1990 to 2021
Source: Clin Kidney J. 2025 May 23;18(9):sfaf160. doi: 10.1093/ckj/sfaf160 (PMC12399972; doi:10.1093/ckj/sfaf160)
Supplement: sfaf160_Supplemental_Files [file sfaf160_supplemental_files.zip › Figures S1-41.pdf]

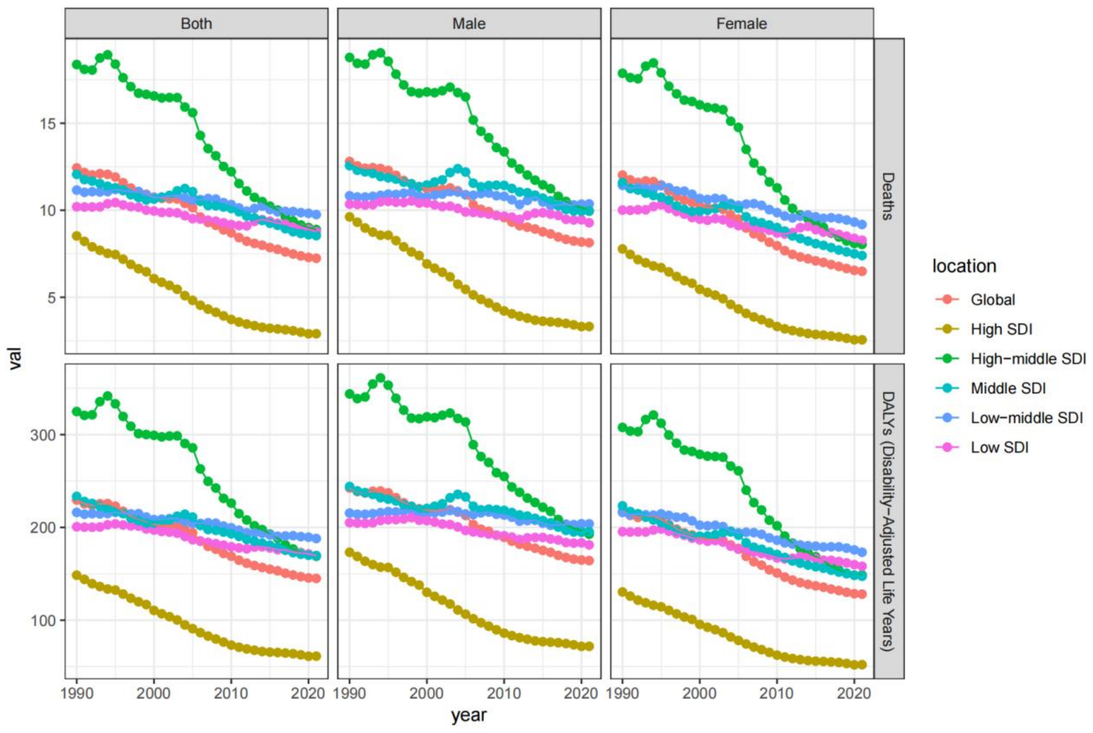

Fig. S1 Trends in kidney dysfunction related ischemic stroke mortality and disability-adjusted life-years from 1990 to 2021

Note: SDI: socio-demographic index.

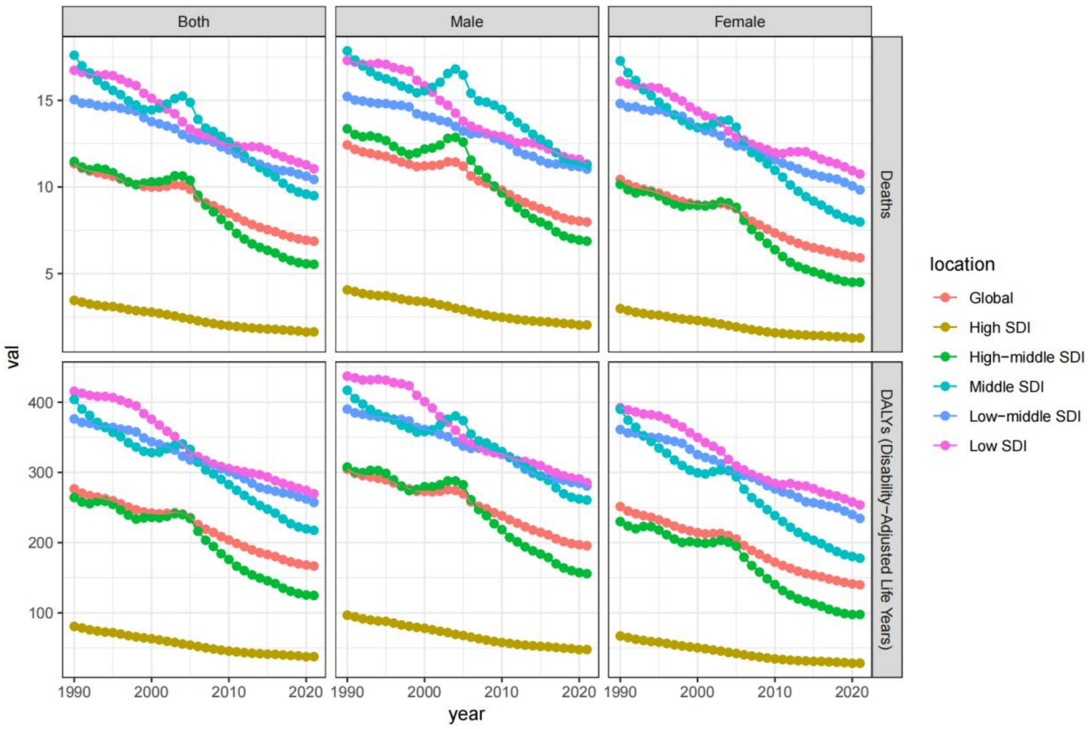

Fig. S2 Trends in kidney dysfunction related intracerebral hemorrhage mortality and disability-adjusted life-years from 1990 to 2021

Note: SDI: socio-demographic index.

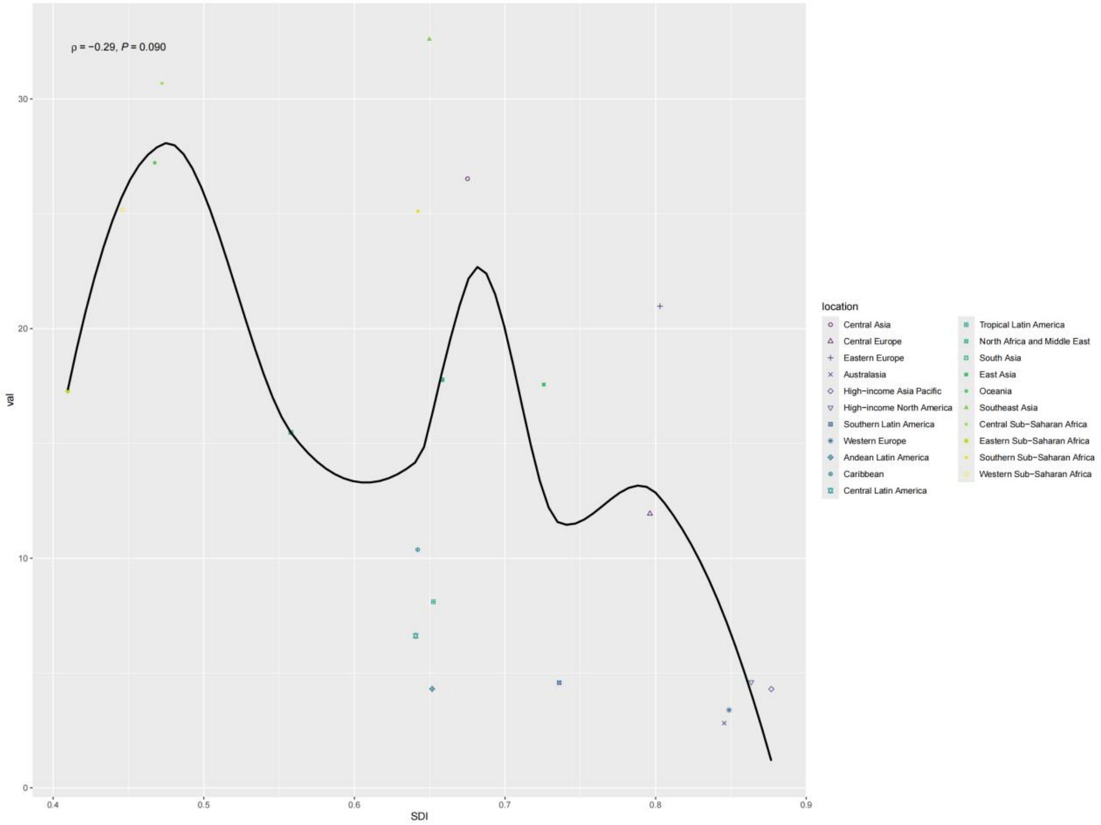

Fig. S3 The associations between age-standardized kidney dysfunction related stroke mortality rate and the sociodemographic index across 21 GBD regions.

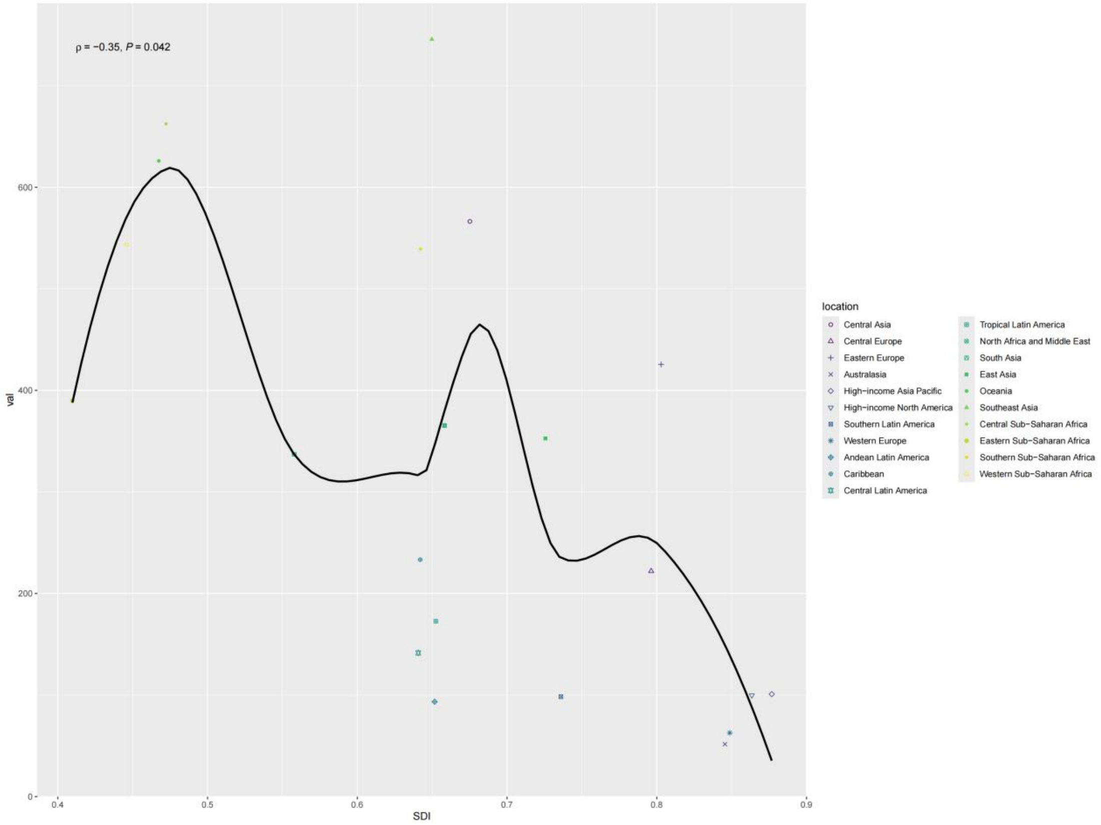

Fig. S4 The associations between age-standardized kidney dysfunction related stroke disability-adjusted life years rate and the sociodemographic index across 21 GBD regions.

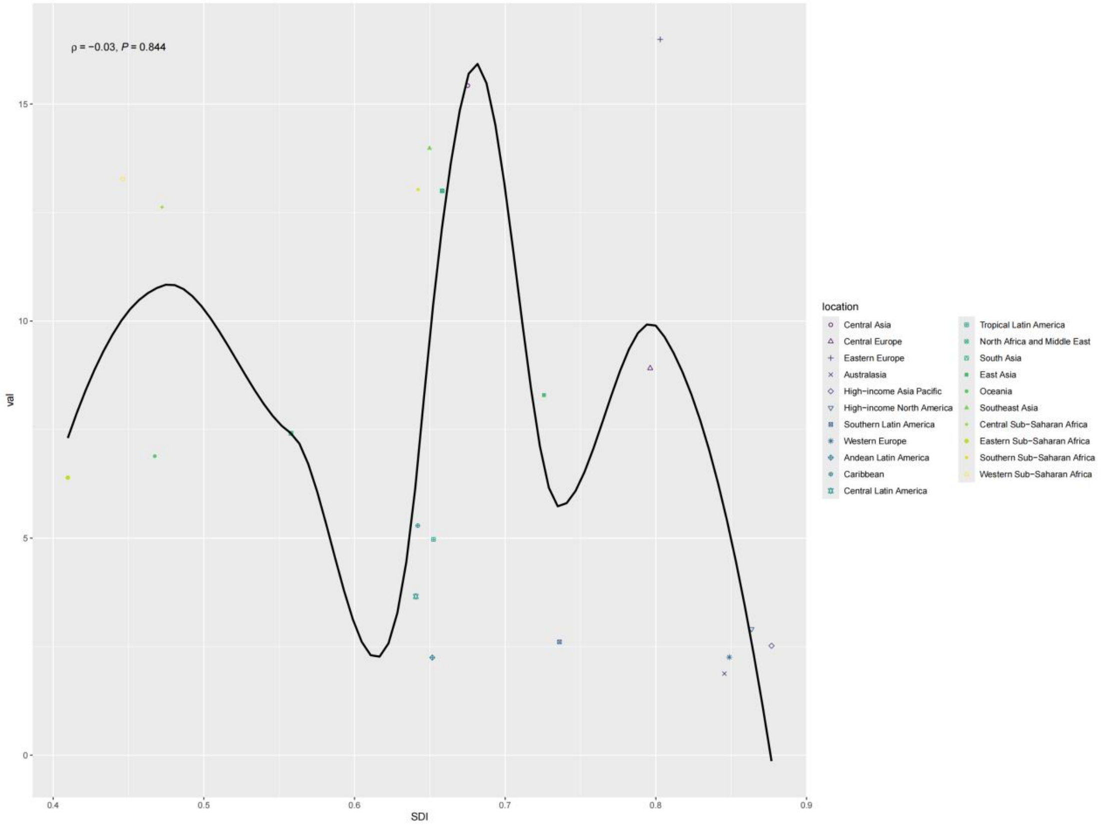

Fig. S5 The associations between age-standardized kidney dysfunction related ischemic stroke mortality rate and the sociodemographic index across 21 GBD regions.

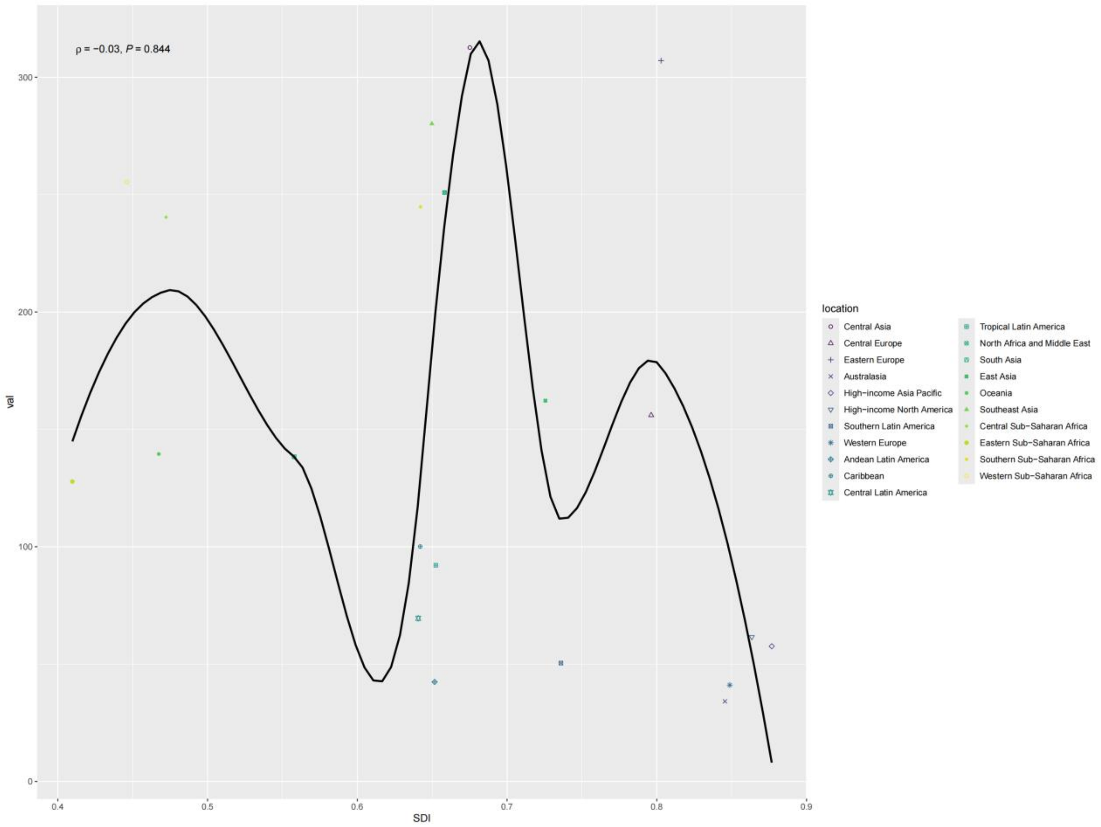

Fig. S6 The associations between age-standardized kidney dysfunction related ischemic stroke disability-adjusted life years rate and the sociodemographic index across 21 GBD regions.

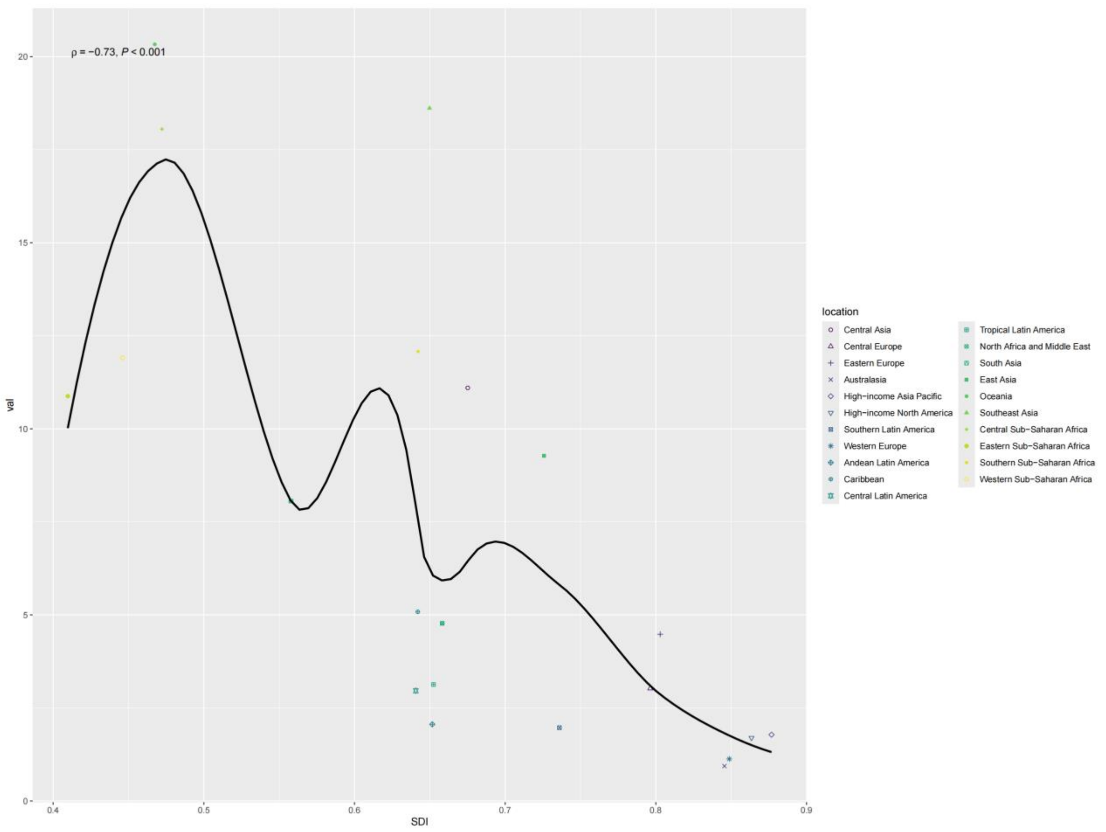

Fig. S7 The associations between age-standardized kidney dysfunction related intracerebral hemorrhage mortality rate and the sociodemographic index across 21 GBD regions.

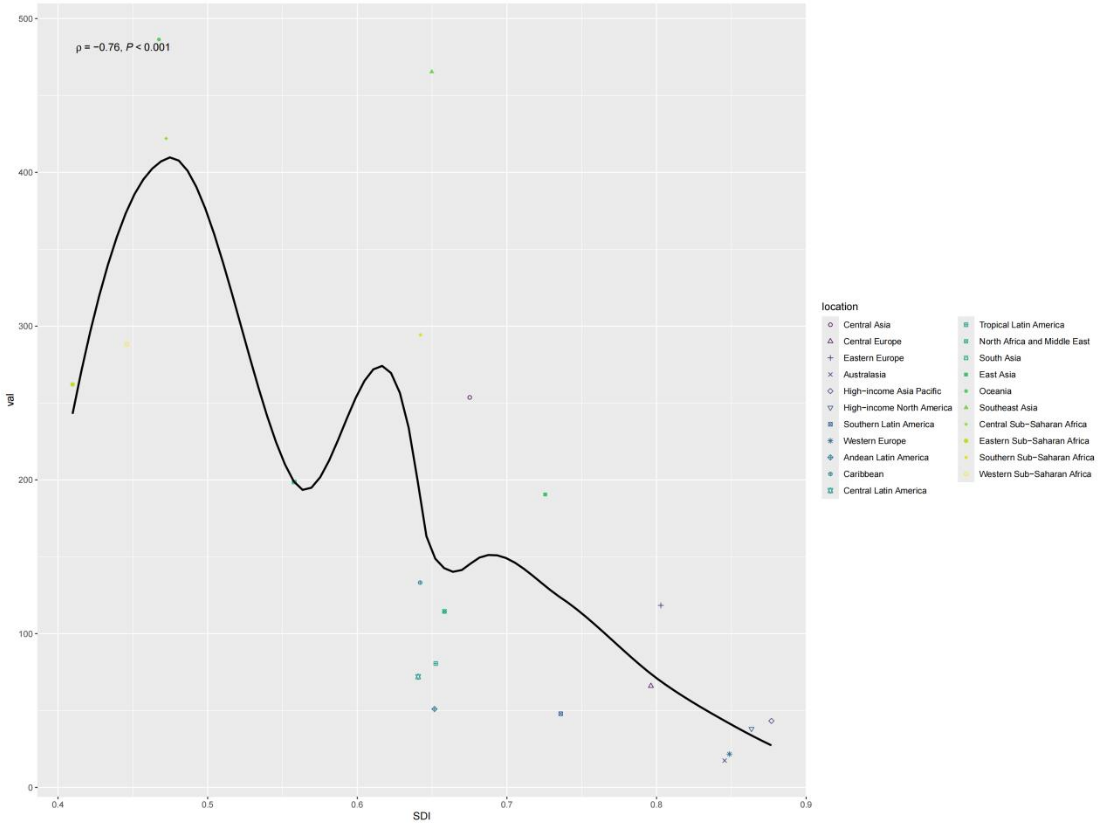

Fig. S8 The associations between age-standardized kidney dysfunction related intracerebral hemorrhage disability-adjusted life years rate and the sociodemographic index across 21 GBD regions.

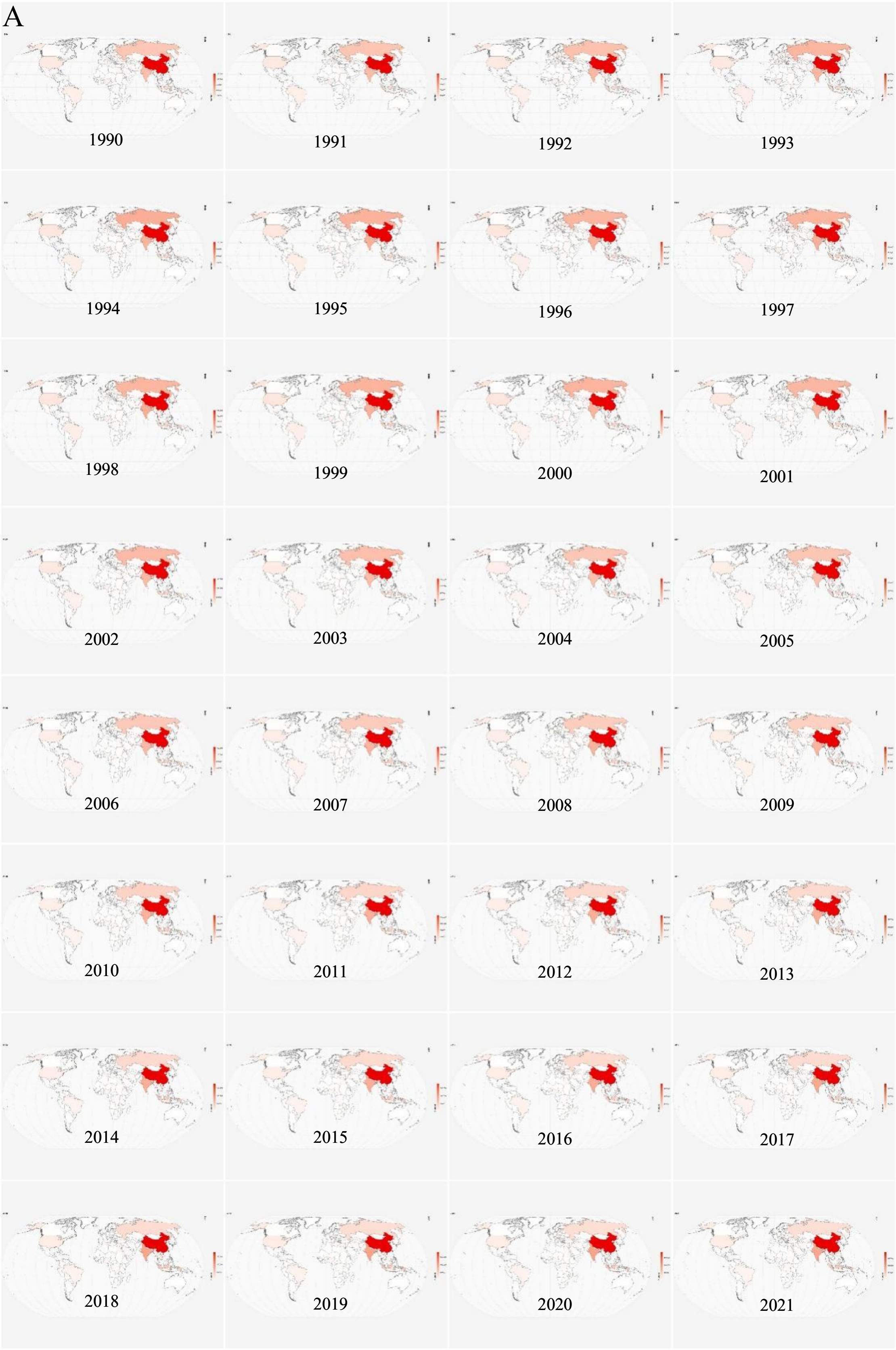
A
1990
1991
1992
1993
1994
1995
1996
1997
1998
1999
2000
2001
2002
2003
2004
2005
2006
2007
2008
2009
2010
2011
2012
2013
2014
2015
2016
2017
2018
2019
2020
2021

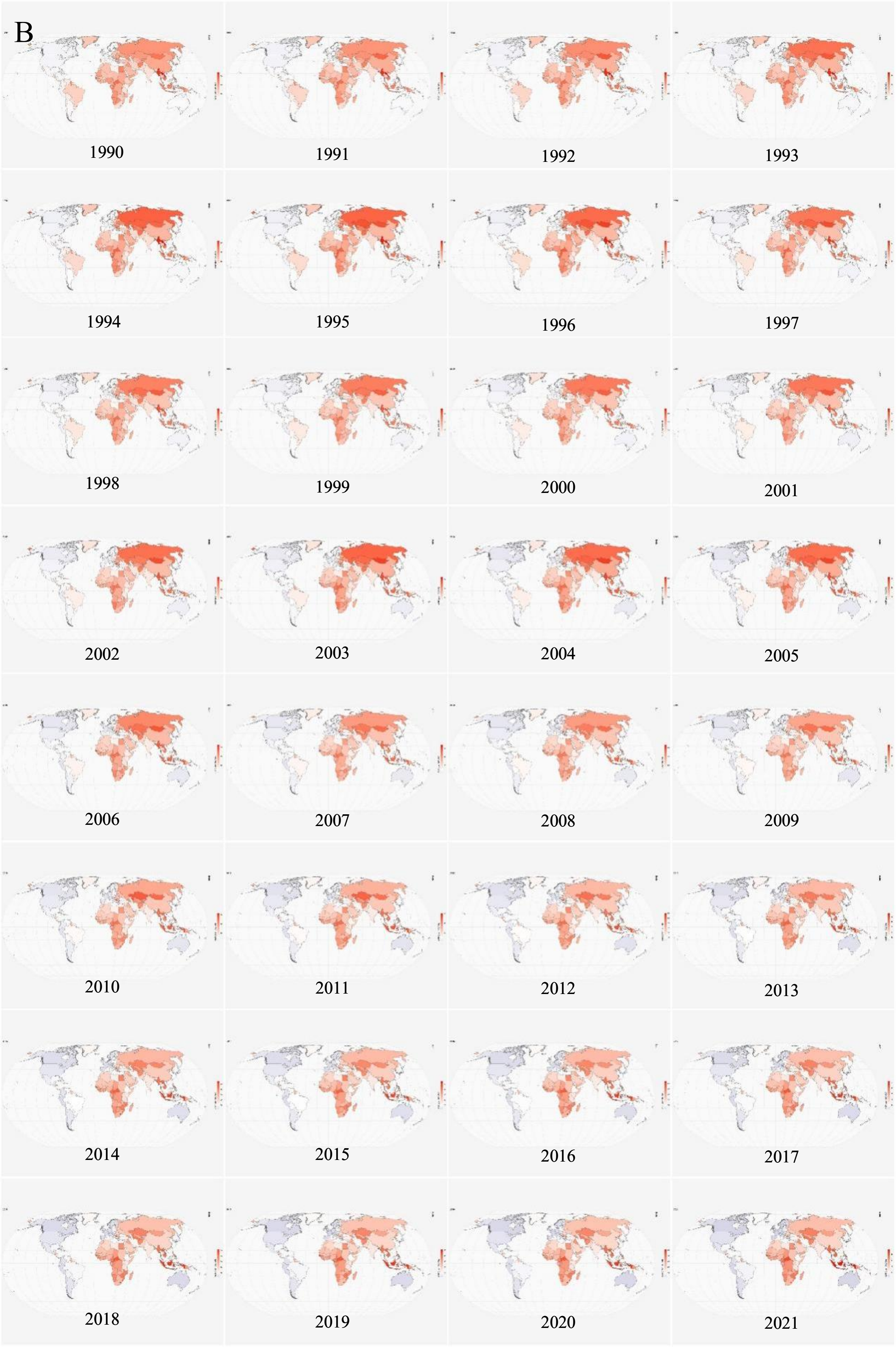

Fig. S9 A The global disease burden of kidney dysfunction related stroke mortality case in 204 countries and territories from 1990 to 2021; B The global disease burden of kidney dysfunction related stroke mortality rate in 204 countries and territories from 1990 to 2021.

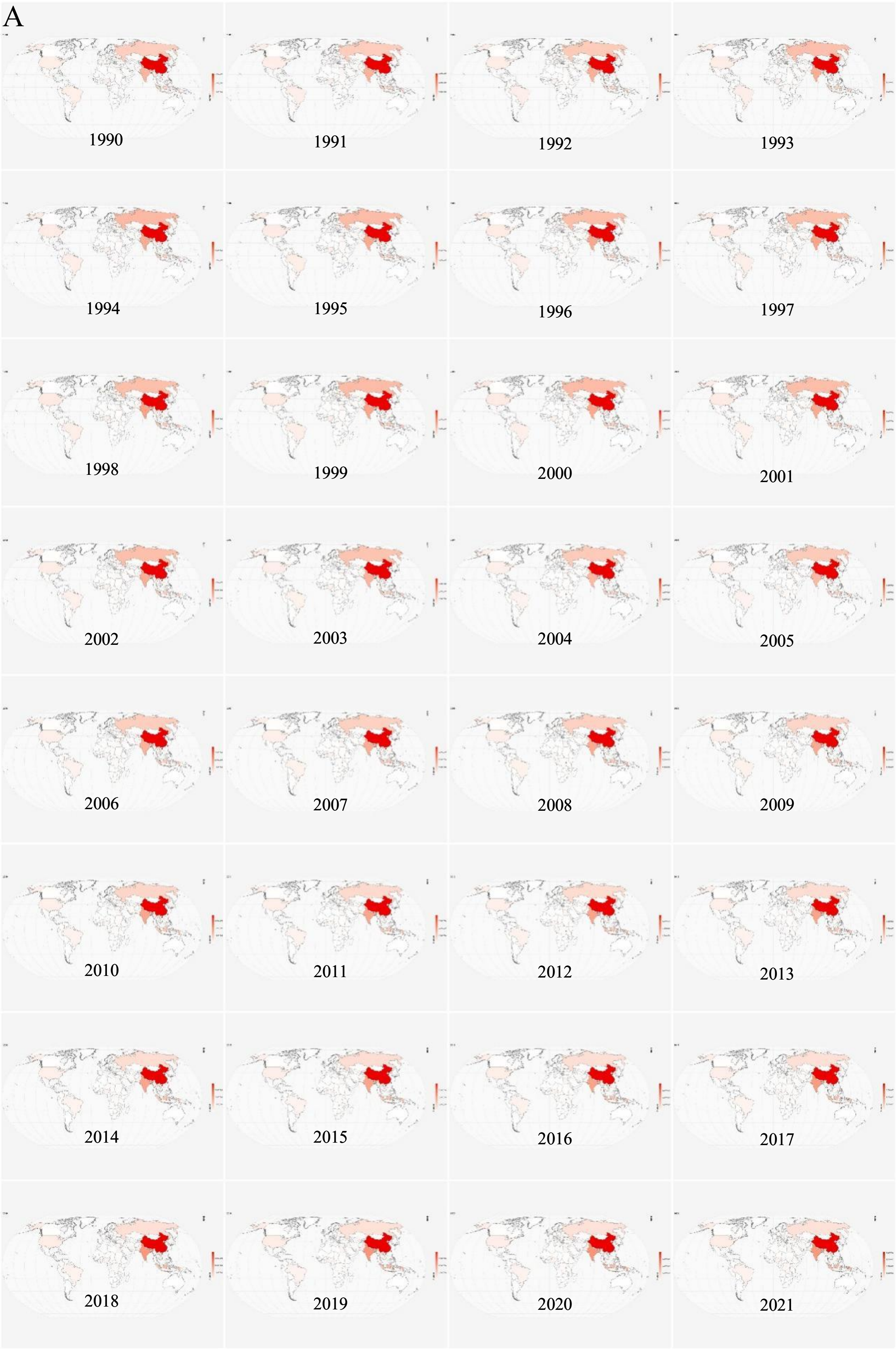
A
1990
1991
1992
1993
1994
1995
1996
1997
1998
1999
2000
2001
2002
2003
2004
2005
2006
2007
2008
2009
2010
2011
2012
2013
2014
2015
2016
2017
2018
2019
2020
2021

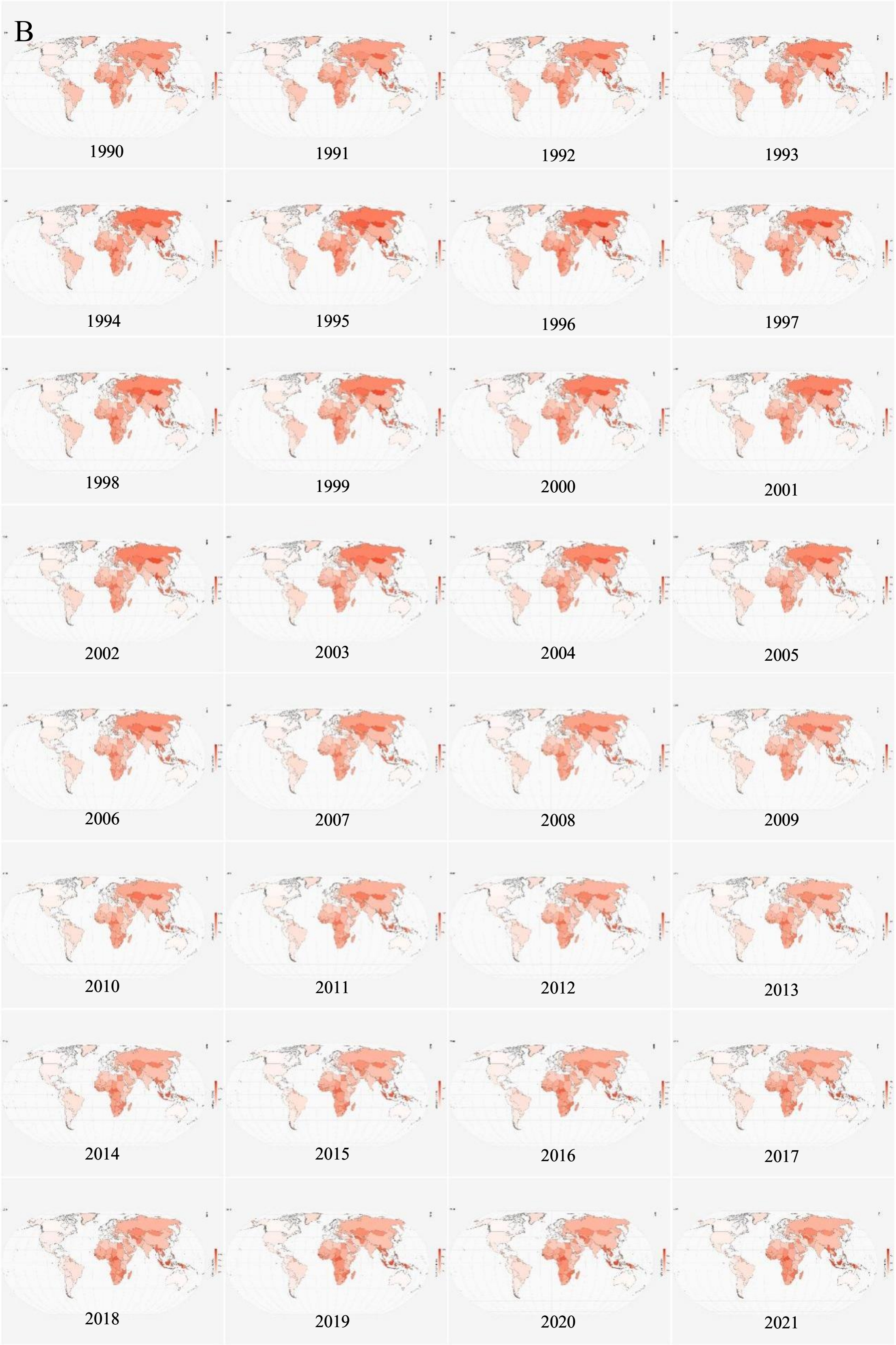

Fig. S10 A The global disease burden of kidney dysfunction related stroke disability-adjusted life years in 204 countries and territories from 1990 to 2021; B The global disease burden of kidney dysfunction related stroke disability-adjusted life years rate in 204 countries and territories from 1990 to 2021.

A

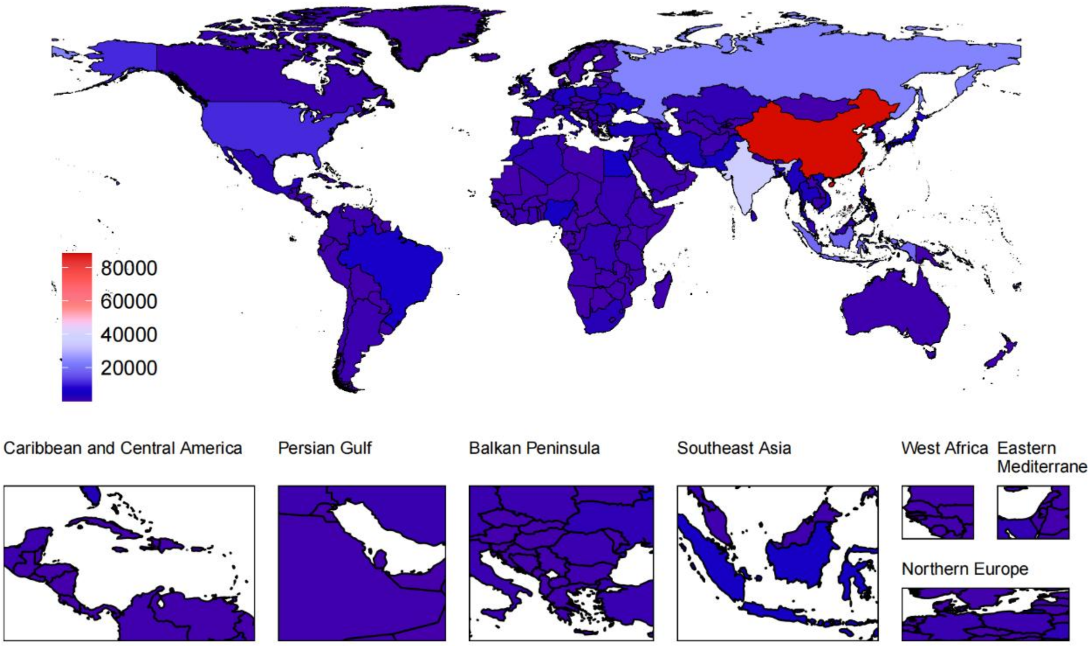
80000
60000
40000
20000
Caribbean and Central America
Persian Gulf
Balkan Peninsula
Southeast Asia
West Africa
Eastern Mediterrane
Northern Europe

B

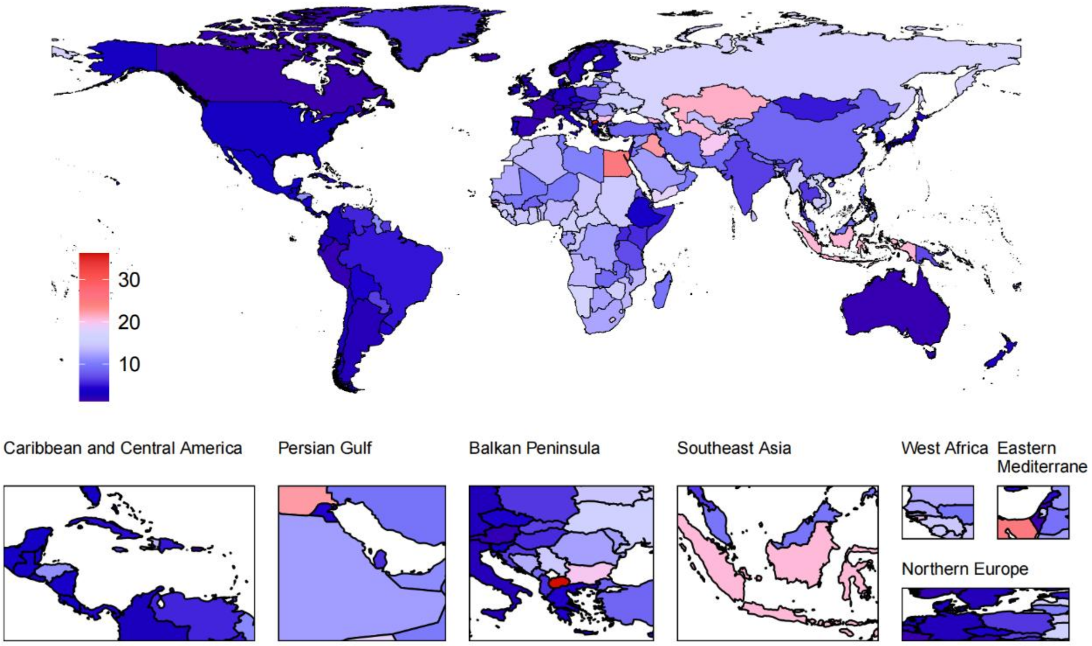
30
20
10
Caribbean and Central America
Persian Gulf
Balkan Peninsula
Southeast Asia
West Africa
Eastern Mediterrane
Northern Europe

C

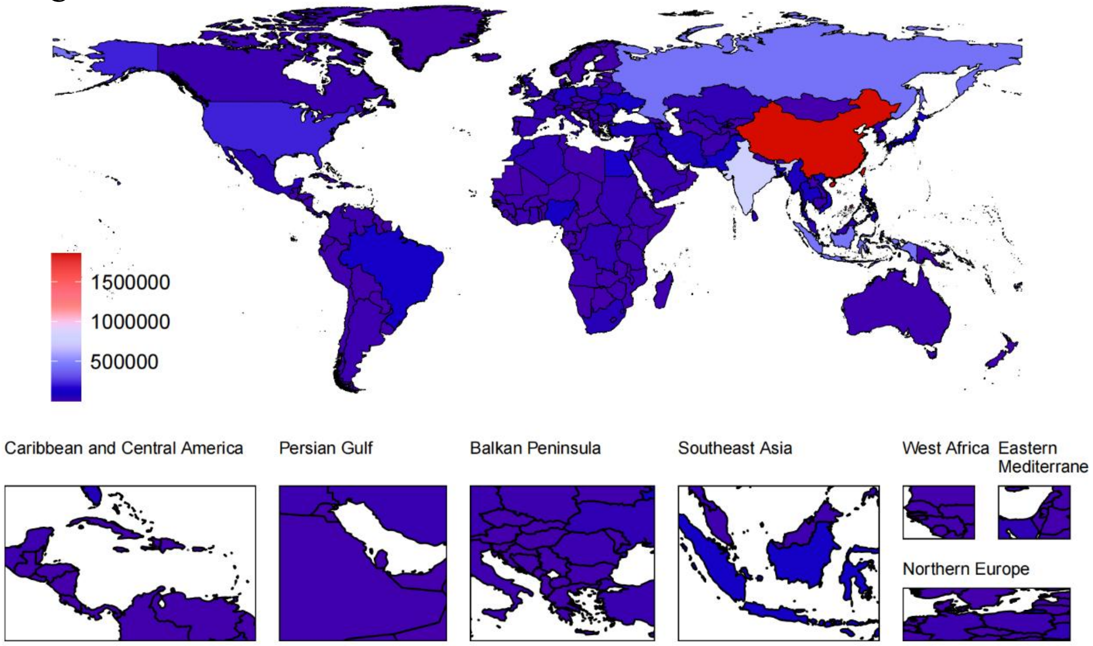

D

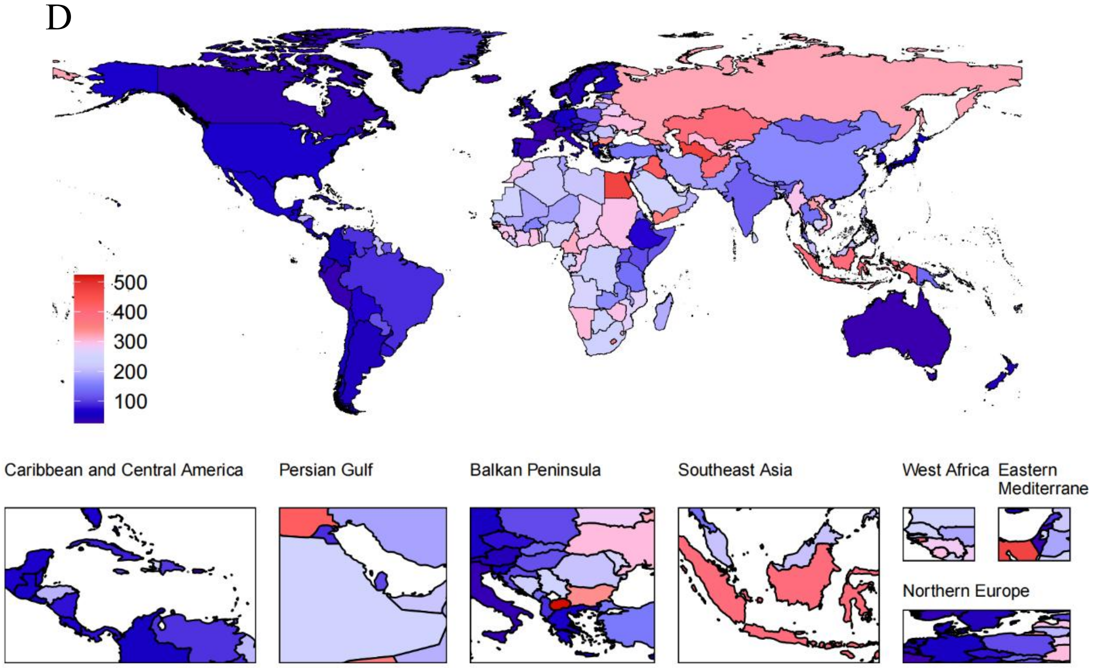

Fig. S11 A The global disease burden of kidney dysfunction related ischemic stroke mortality number in 204 countries and territories; B The global disease burden of kidney dysfunction related ischemic stroke mortality rate in 204 countries and territories; C The global disease burden of kidney dysfunction related ischemic stroke disability-adjusted life years number in 204 countries and territories; D The global disease burden of kidney dysfunction related ischemic stroke disability-adjusted life years rate in 204 countries and territories.

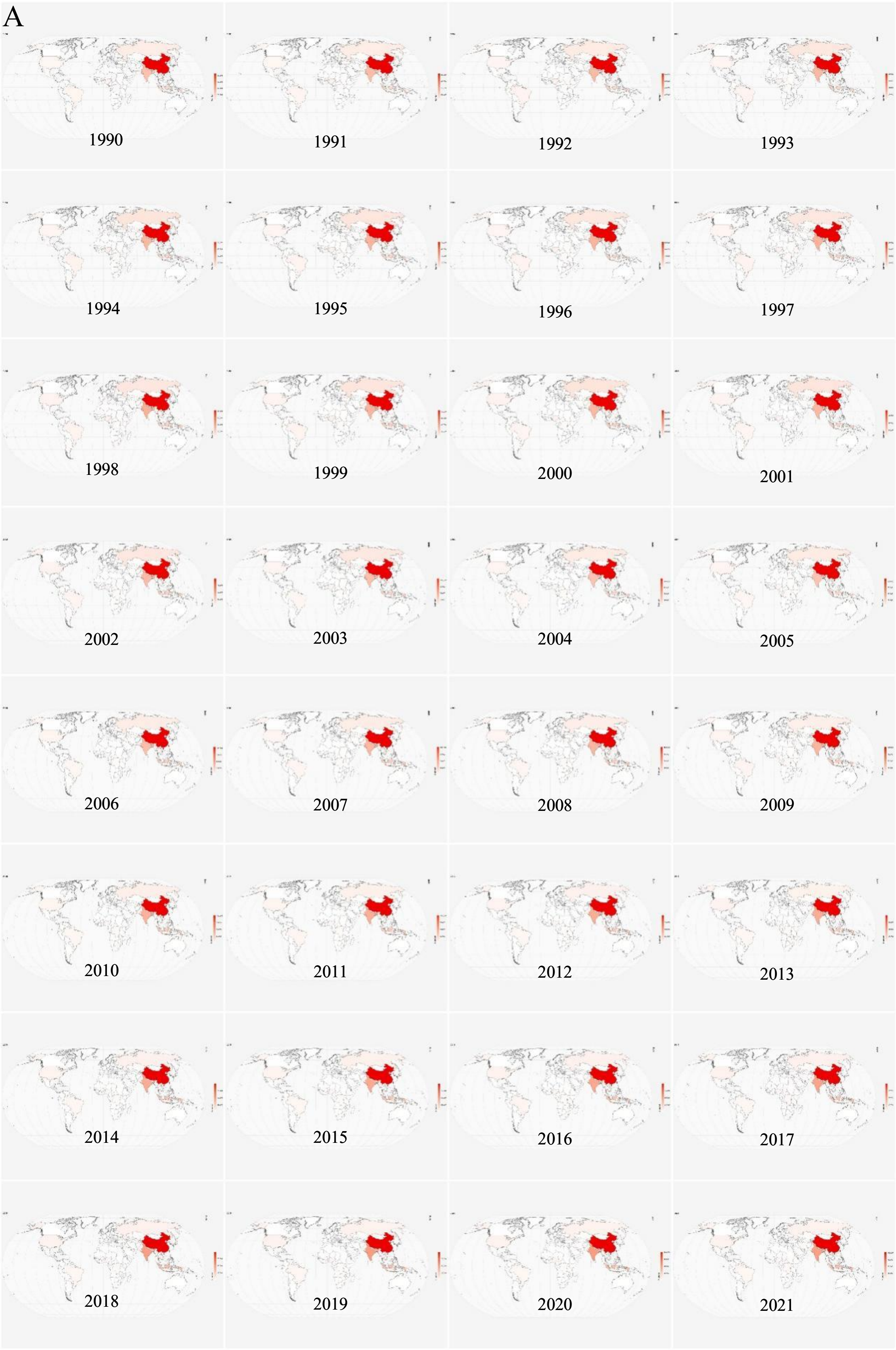
A
1990
1991
1992
1993
1994
1995
1996
1997
1998
1999
2000
2001
2002
2003
2004
2005
2006
2007
2008
2009
2010
2011
2012
2013
2014
2015
2016
2017
2018
2019
2020
2021

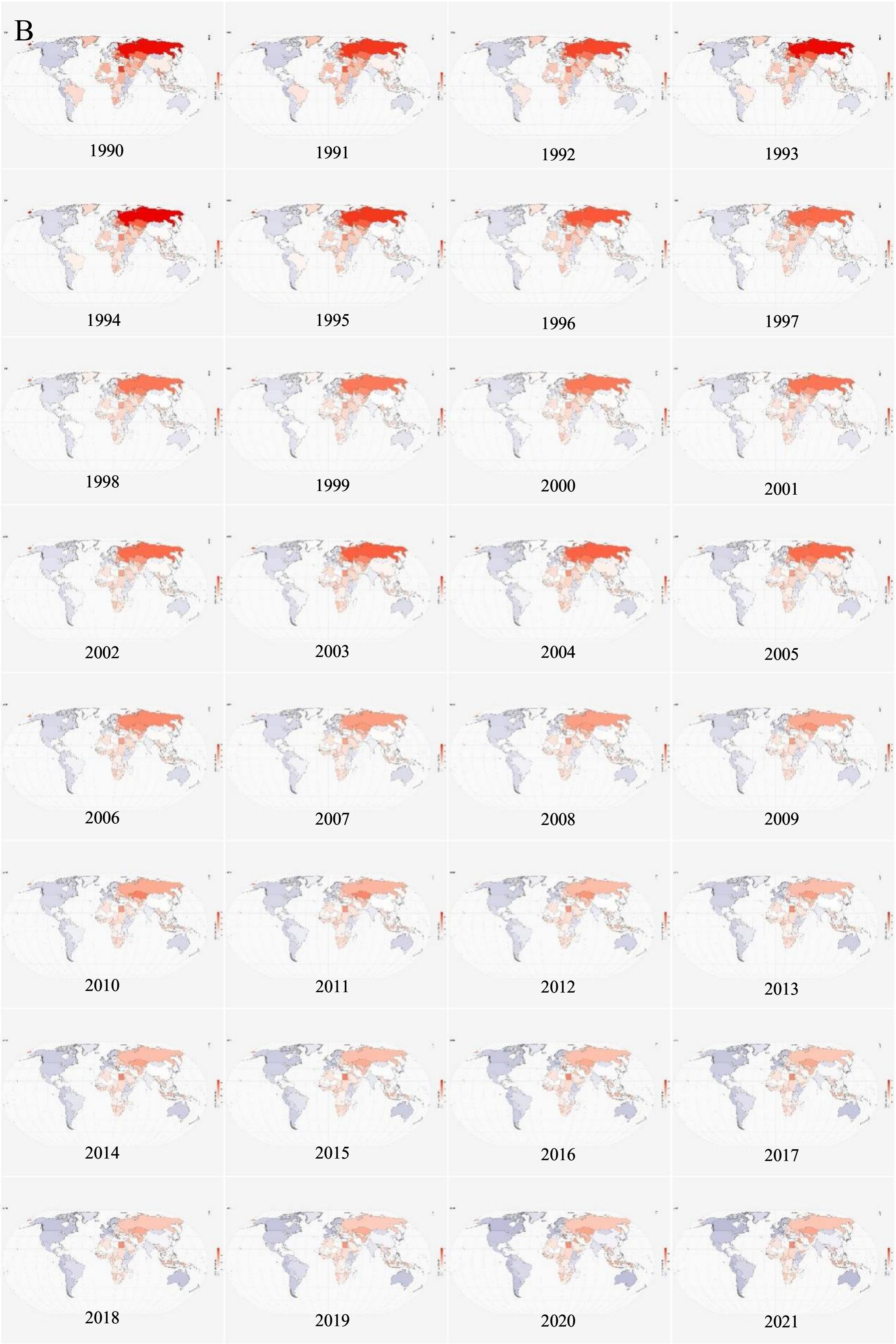

Fig. S12 A The global disease burden of kidney dysfunction related ischemic stroke mortality case in 204 countries and territories from 1990 to 2021; B The global disease burden of kidney dysfunction related ischemic stroke mortality rate in 204 countries and territories from 1990 to 2021.

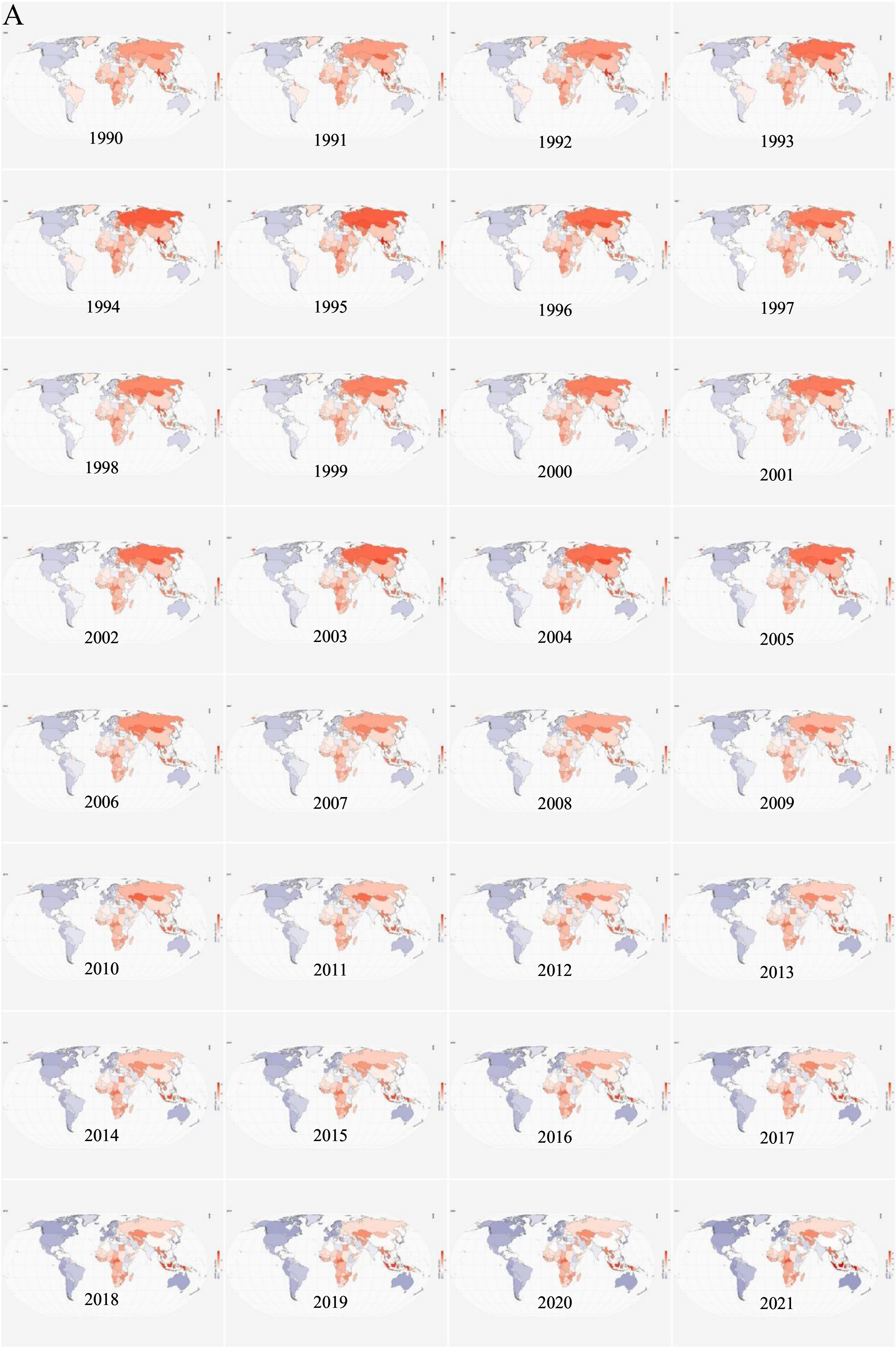
A
1990
1991
1992
1993
1994
1995
1996
1997
1998
1999
2000
2001
2002
2003
2004
2005
2006
2007
2008
2009
2010
2011
2012
2013
2014
2015
2016
2017
2018
2019
2020
2021

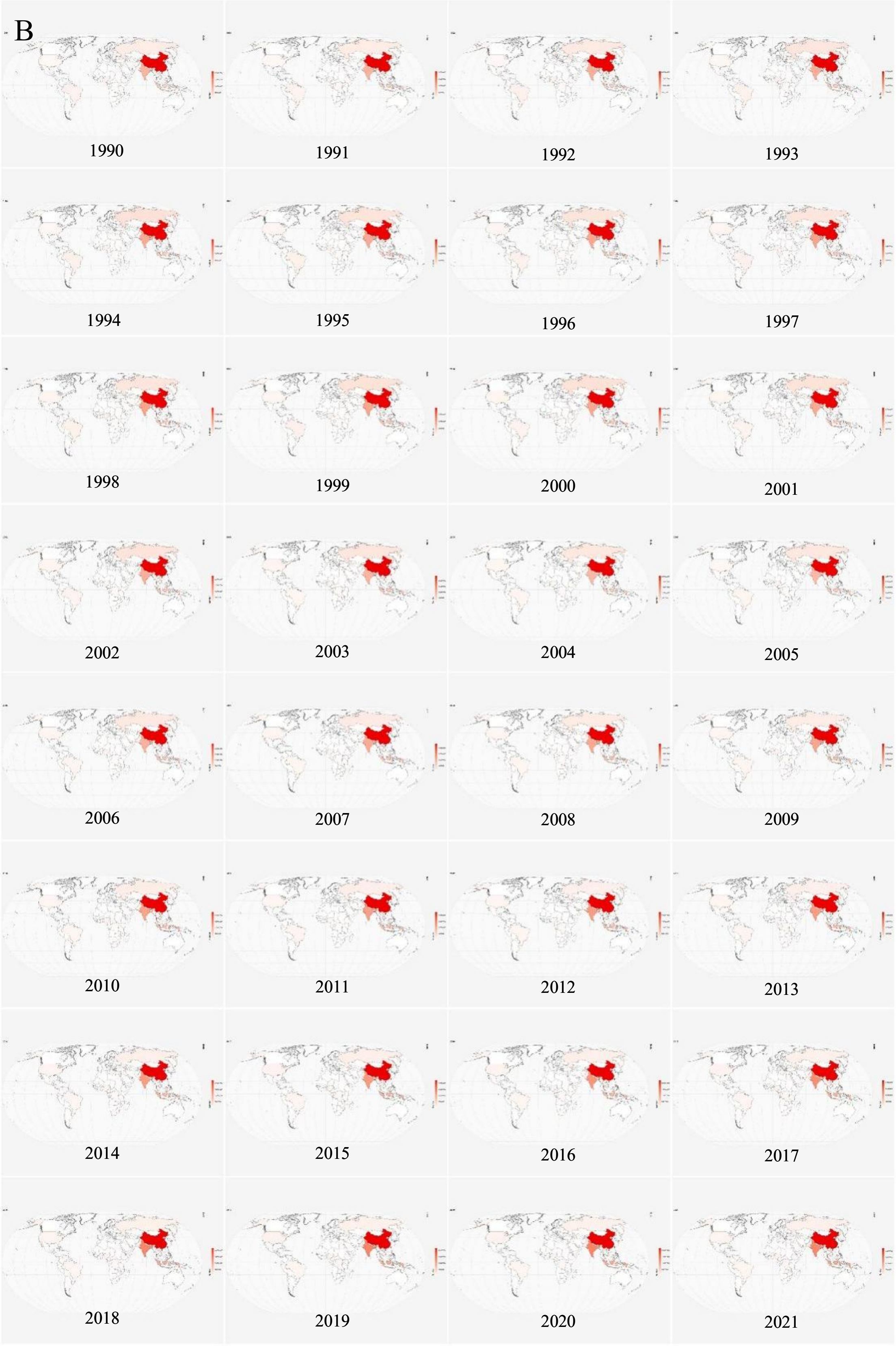

Fig. S13 A The global disease burden of kidney dysfunction related ischemic stroke disability-adjusted life years in 204 countries and territories from 1990 to 2021; B The global disease burden of kidney dysfunction related ischemic stroke disability-adjusted life years rate in 204 countries and territories from 1990 to 2021.

A

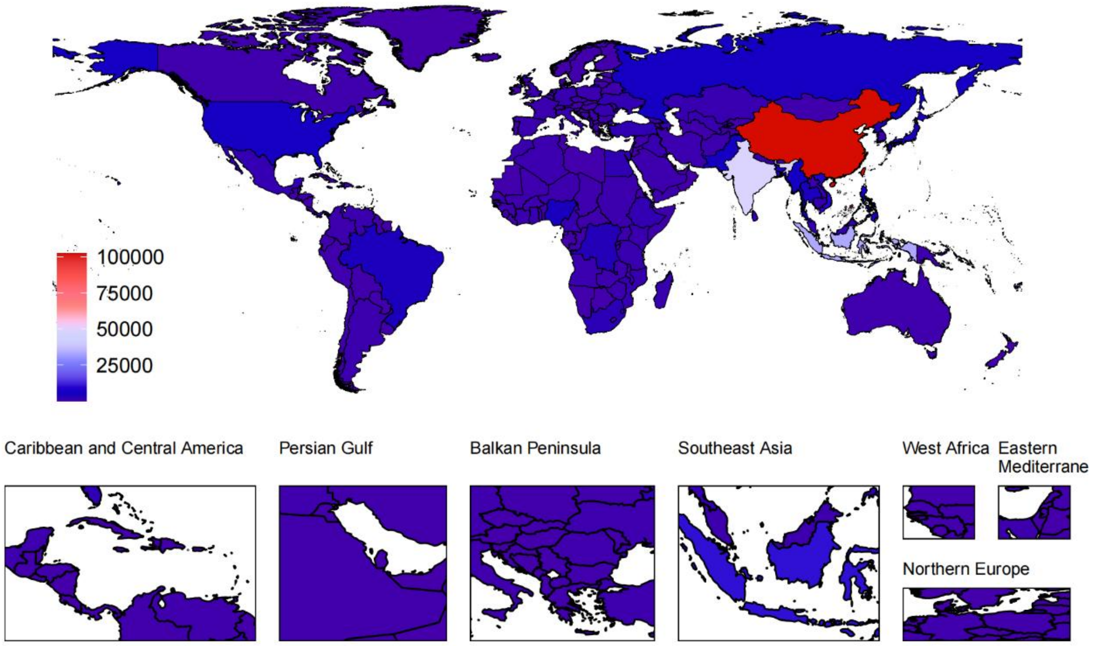
100000
75000
50000
25000
Caribbean and Central America
Persian Gulf
Balkan Peninsula
Southeast Asia
West Africa
Eastern
Mediterrane
Northern Europe

B

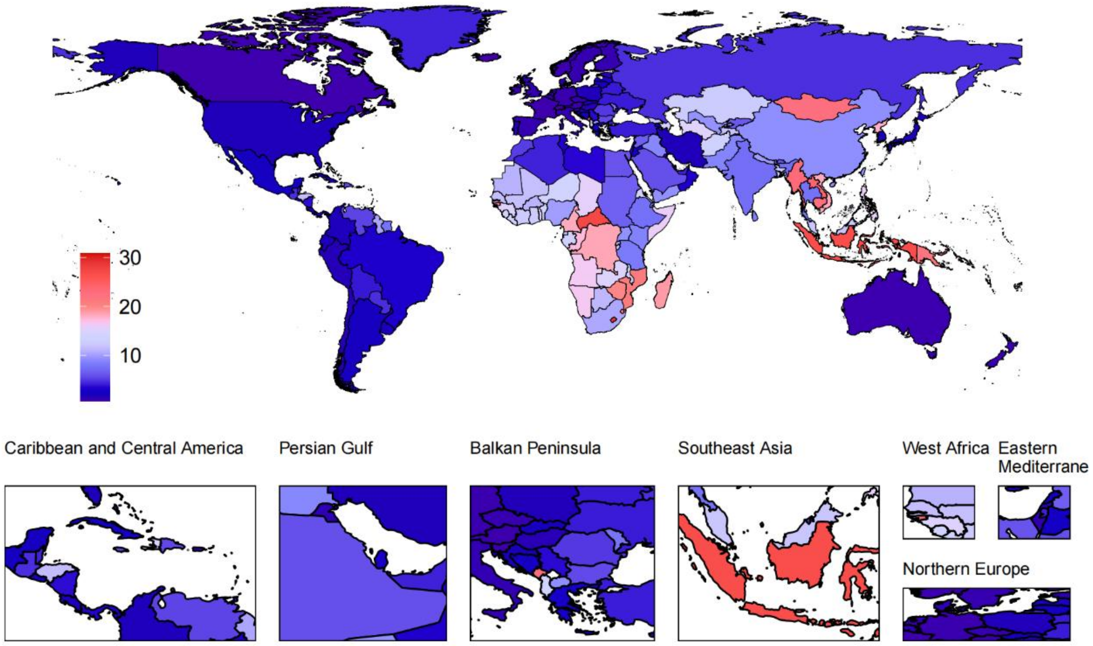
30
20
10
Caribbean and Central America
Persian Gulf
Balkan Peninsula
Southeast Asia
West Africa
Eastern
Mediterrane
Northern Europe

C

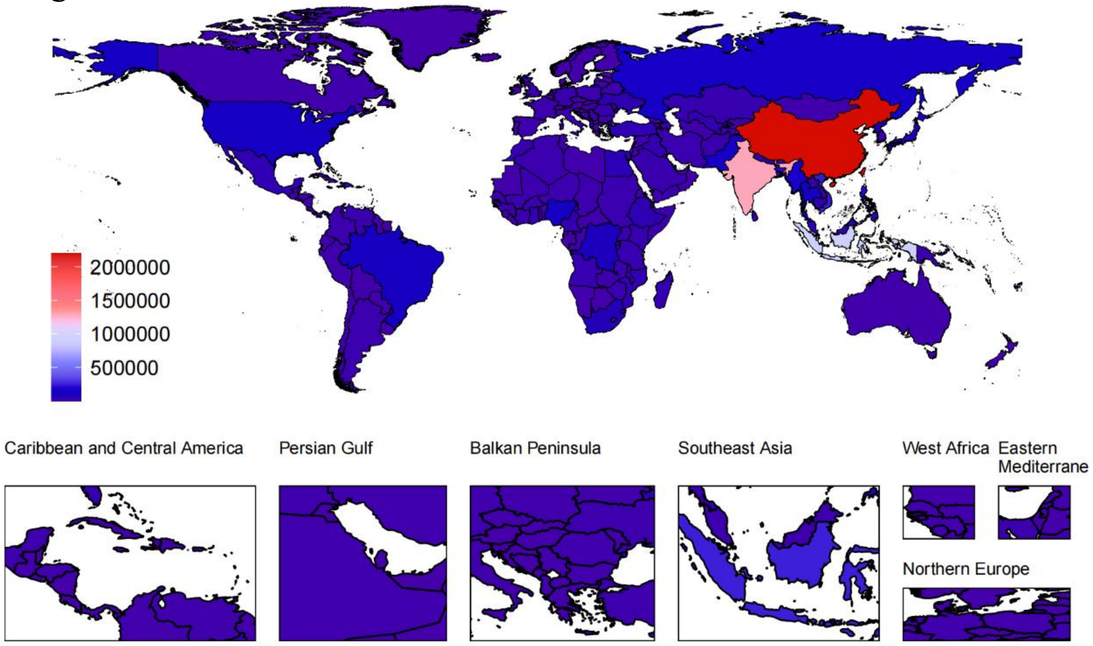

D

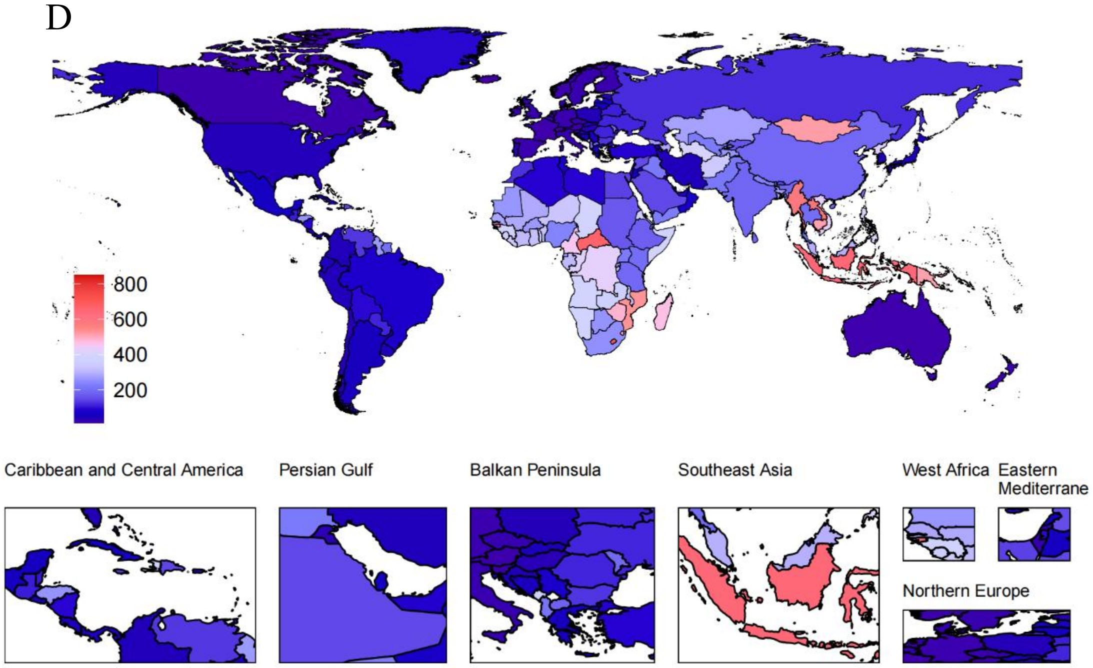

Fig. S14 A The global disease burden of kidney dysfunction related intracerebral hemorrhage mortality number in 204 countries and territories; B The global disease burden of kidney dysfunction related intracerebral hemorrhage mortality rate in 204 countries and territories; C The global disease burden of kidney dysfunction related intracerebral hemorrhage disability-adjusted life years number in 204 countries and territories; D The global disease burden of kidney dysfunction related intracerebral hemorrhage disability-adjusted life years rate in 204 countries and territories

A

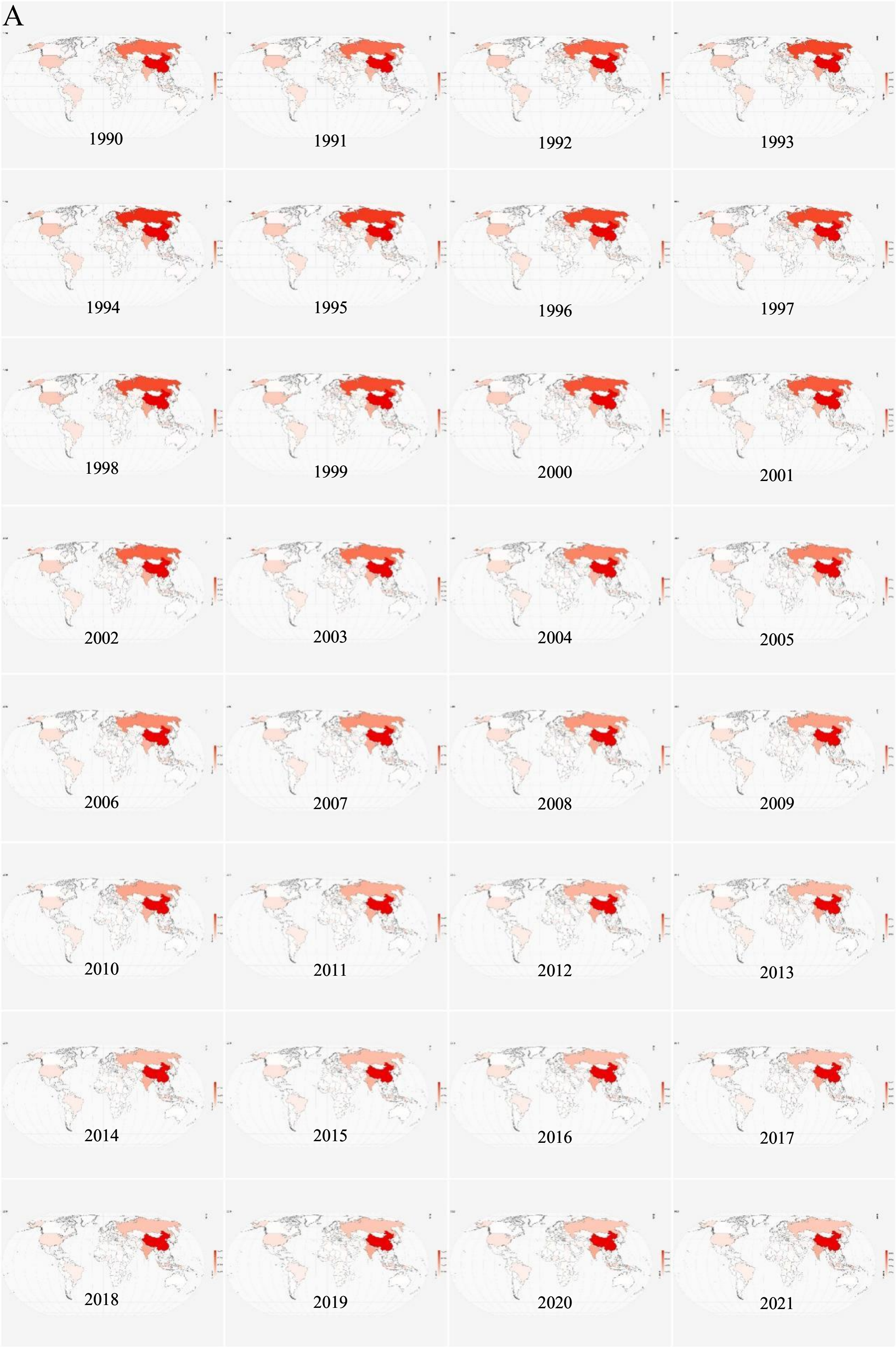
1990
1991
1992
1993
1994
1995
1996
1997
1998
1999
2000
2001
2002
2003
2004
2005
2006
2007
2008
2009
2010
2011
2012
2013
2014
2015
2016
2017
2018
2019
2020
2021

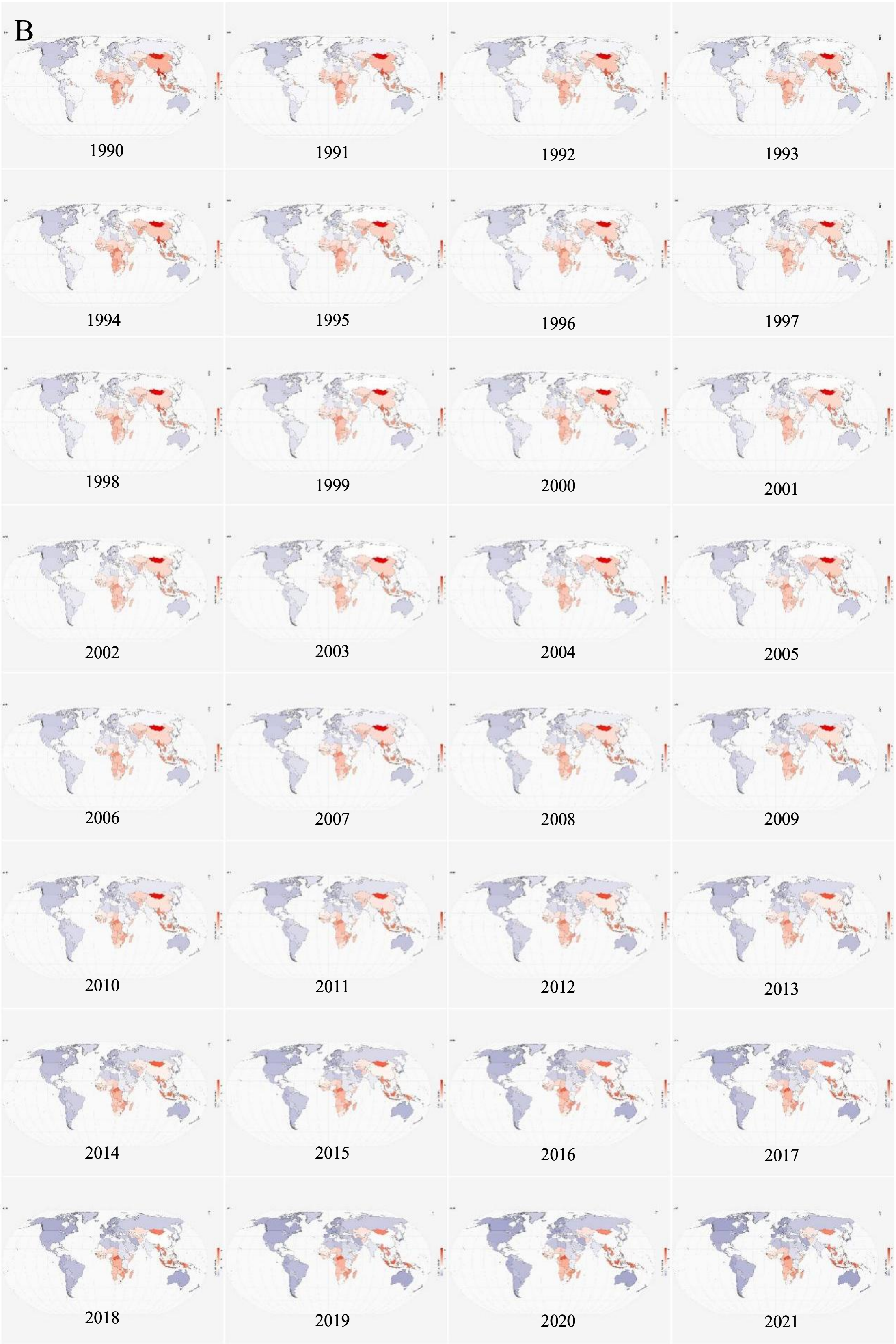

Fig. S15 A The global disease burden of kidney dysfunction related intracerebral hemorrhage mortality case in 204 countries and territories from 1990 to 2021; B The global disease burden of kidney dysfunction related intracerebral hemorrhage mortality rate in 204 countries and territories from 1990 to 2021.

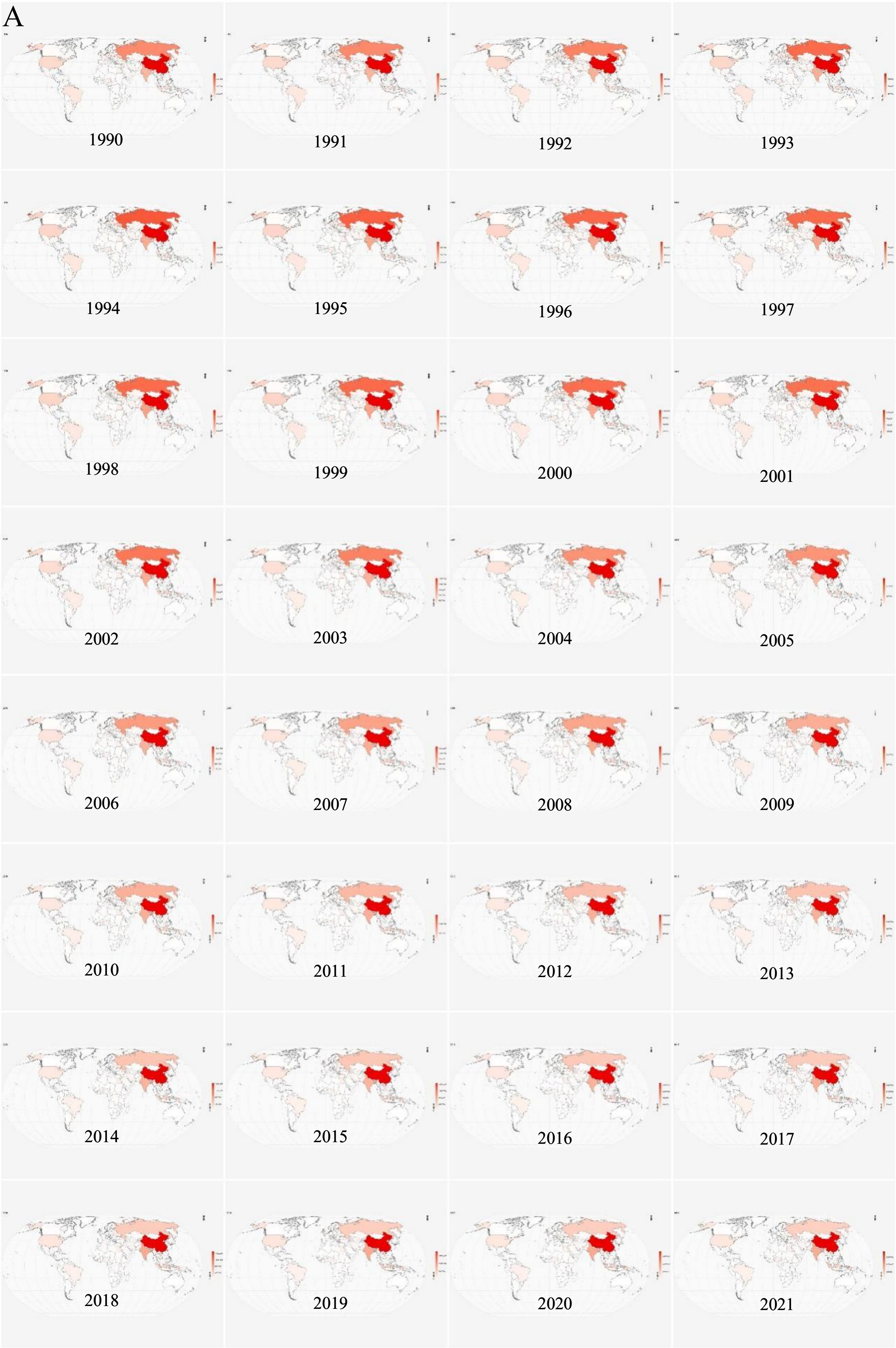
A
1990
1991
1992
1993
1994
1995
1996
1997
1998
1999
2000
2001
2002
2003
2004
2005
2006
2007
2008
2009
2010
2011
2012
2013
2014
2015
2016
2017
2018
2019
2020
2021

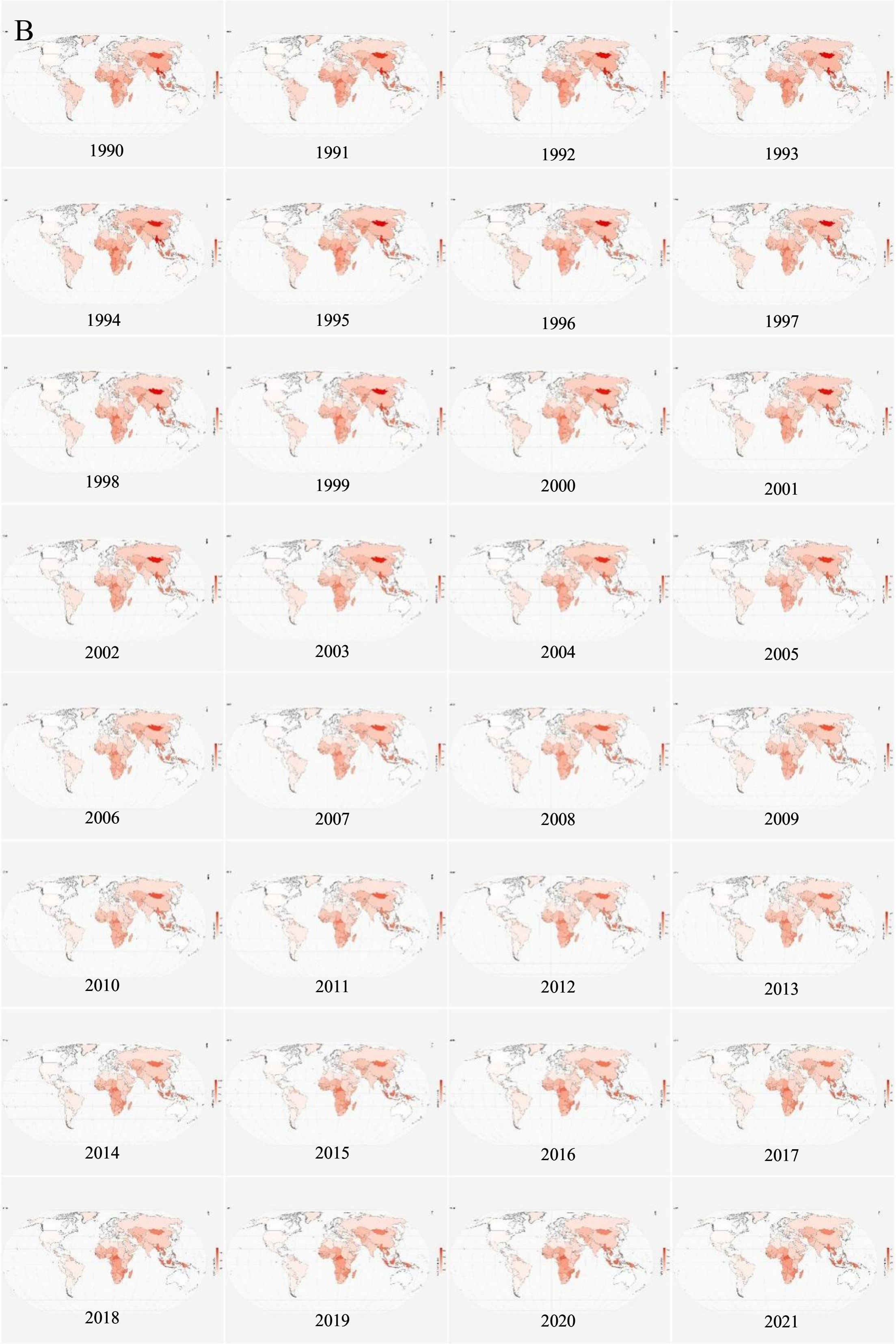

Fig. S16 A The global disease burden of kidney dysfunction related intracerebral hemorrhage disability-adjusted life years in 204 countries and territories from 1990 to 2021; B The global disease burden of kidney dysfunction related intracerebral hemorrhage disability-adjusted life years rate in 204 countries and territories from 1990 to 2021.

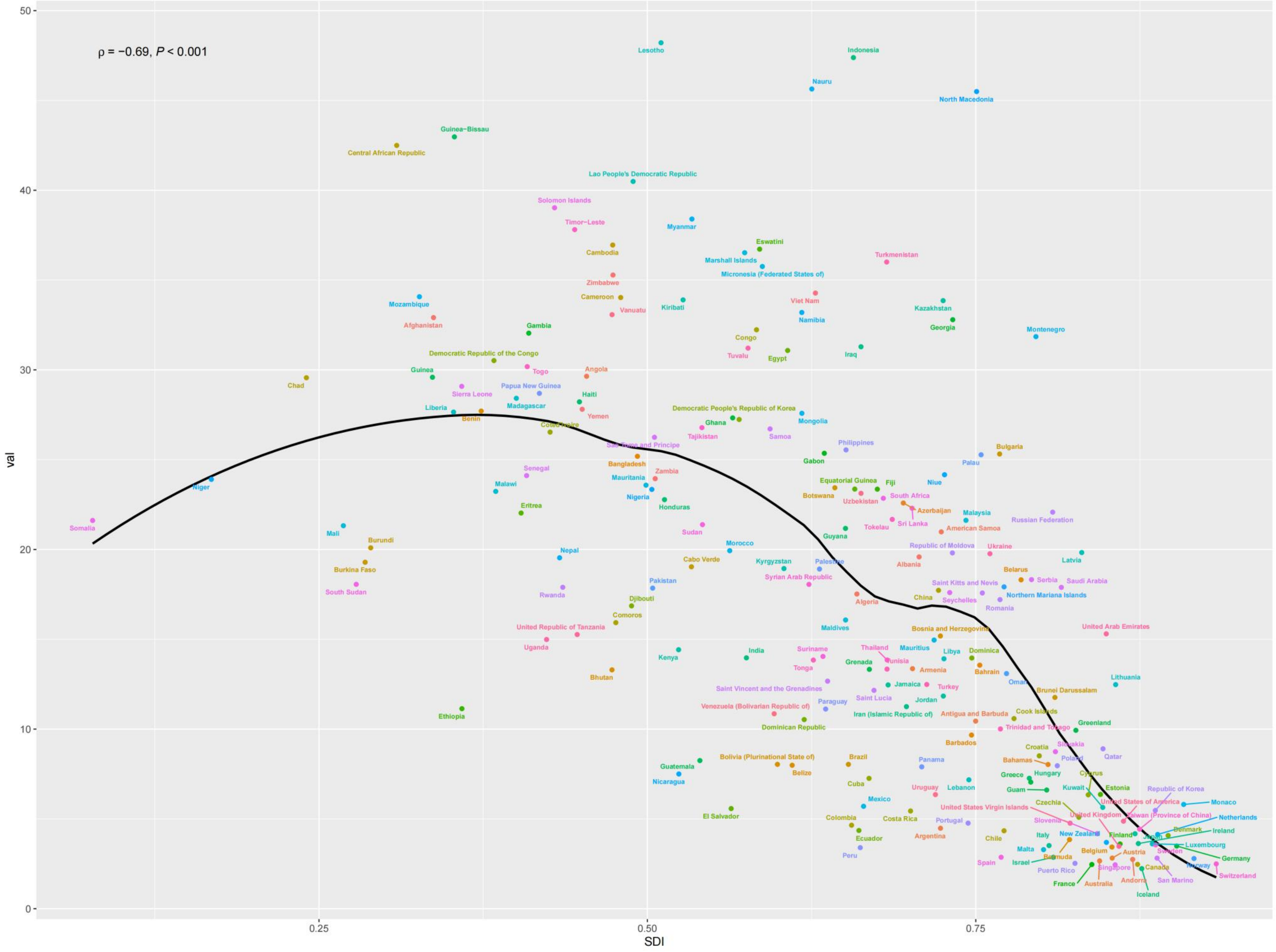

Fig. S17 The associations between age-standardized kidney dysfunction related stroke mortality rate and the sociodemographic index across 204 nations and territories.

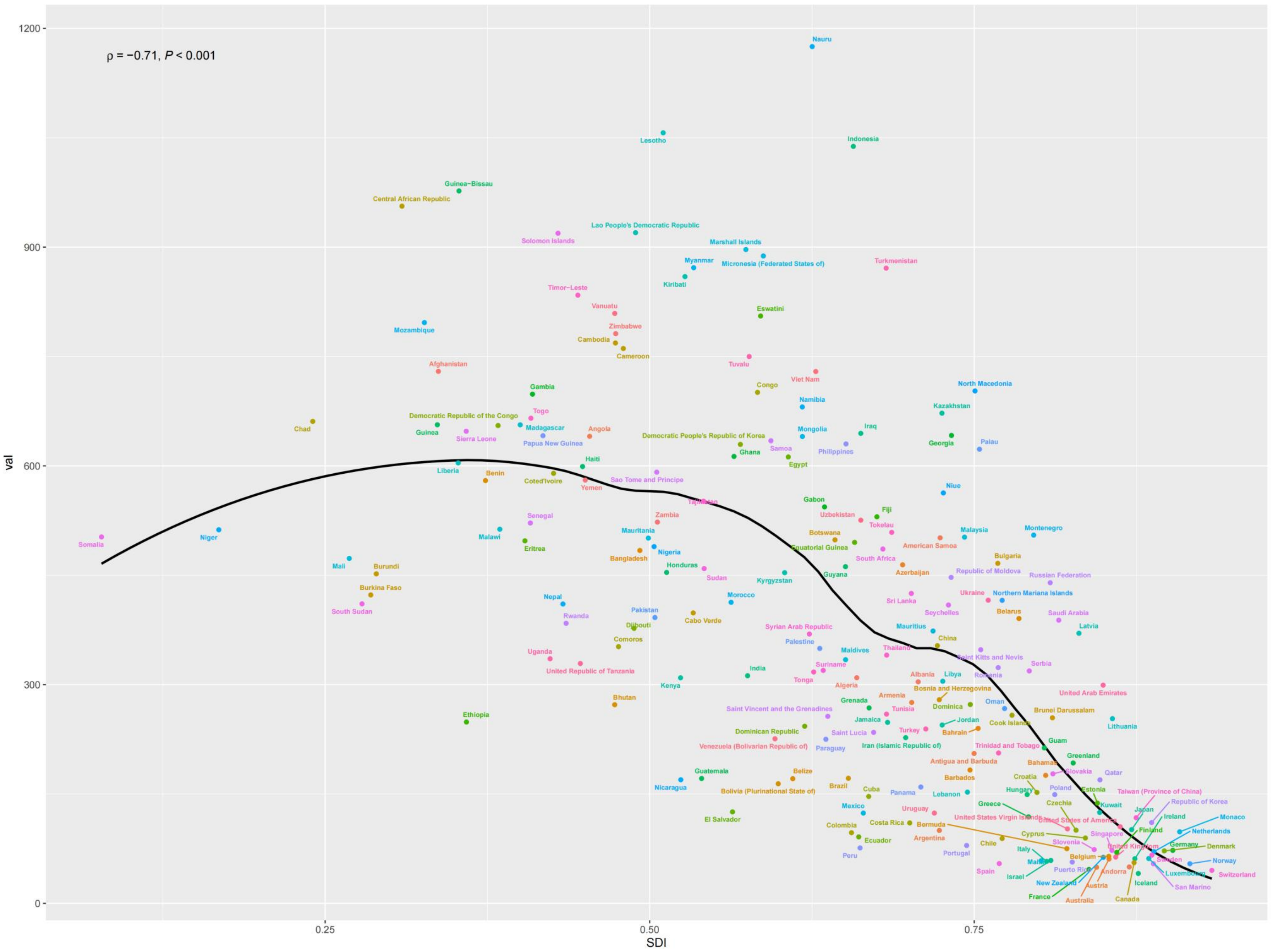

Fig. S18 The associations between age-standardized kidney dysfunction related stroke disability-adjusted life years rate and the sociodemographic index across 204 nations and territories.

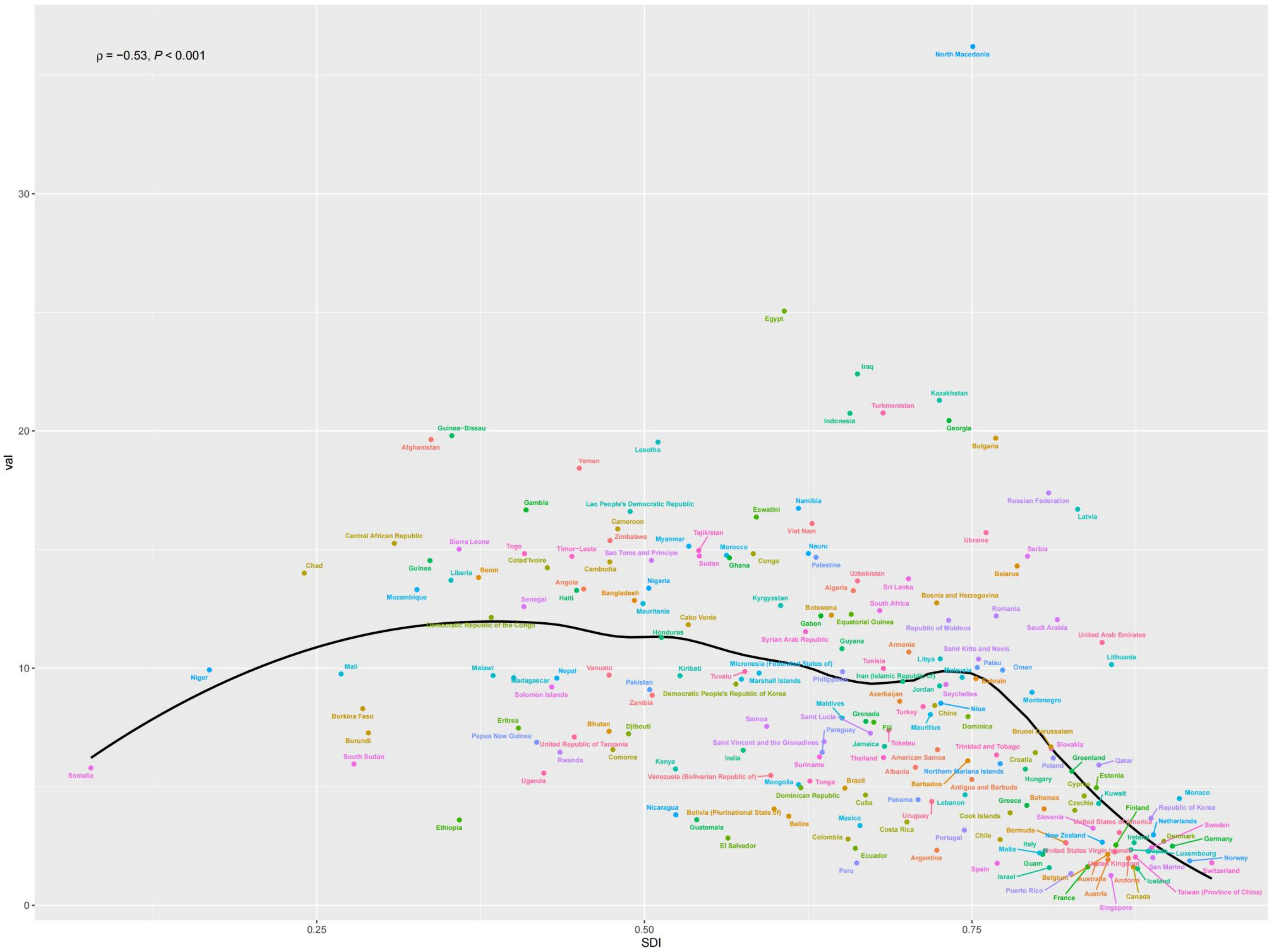

Fig. S19 The associations between age-standardized kidney dysfunction related ischemic stroke mortality rate and the sociodemographic index across 204 nations and territories.

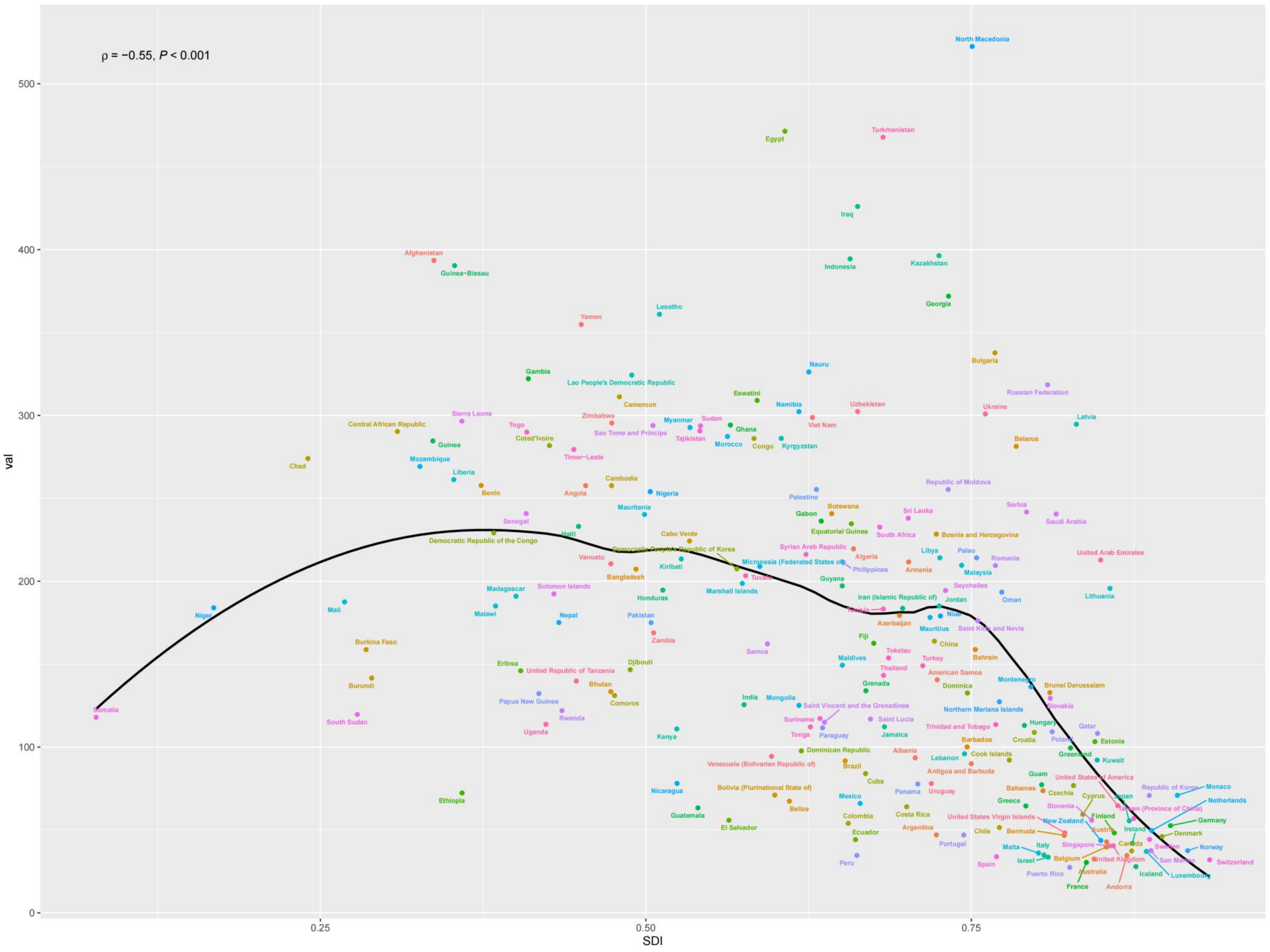

Fig. S20 The associations between age-standardized kidney dysfunction related ischemic stroke mortality rate and the sociodemographic index across 204 nations and territories.

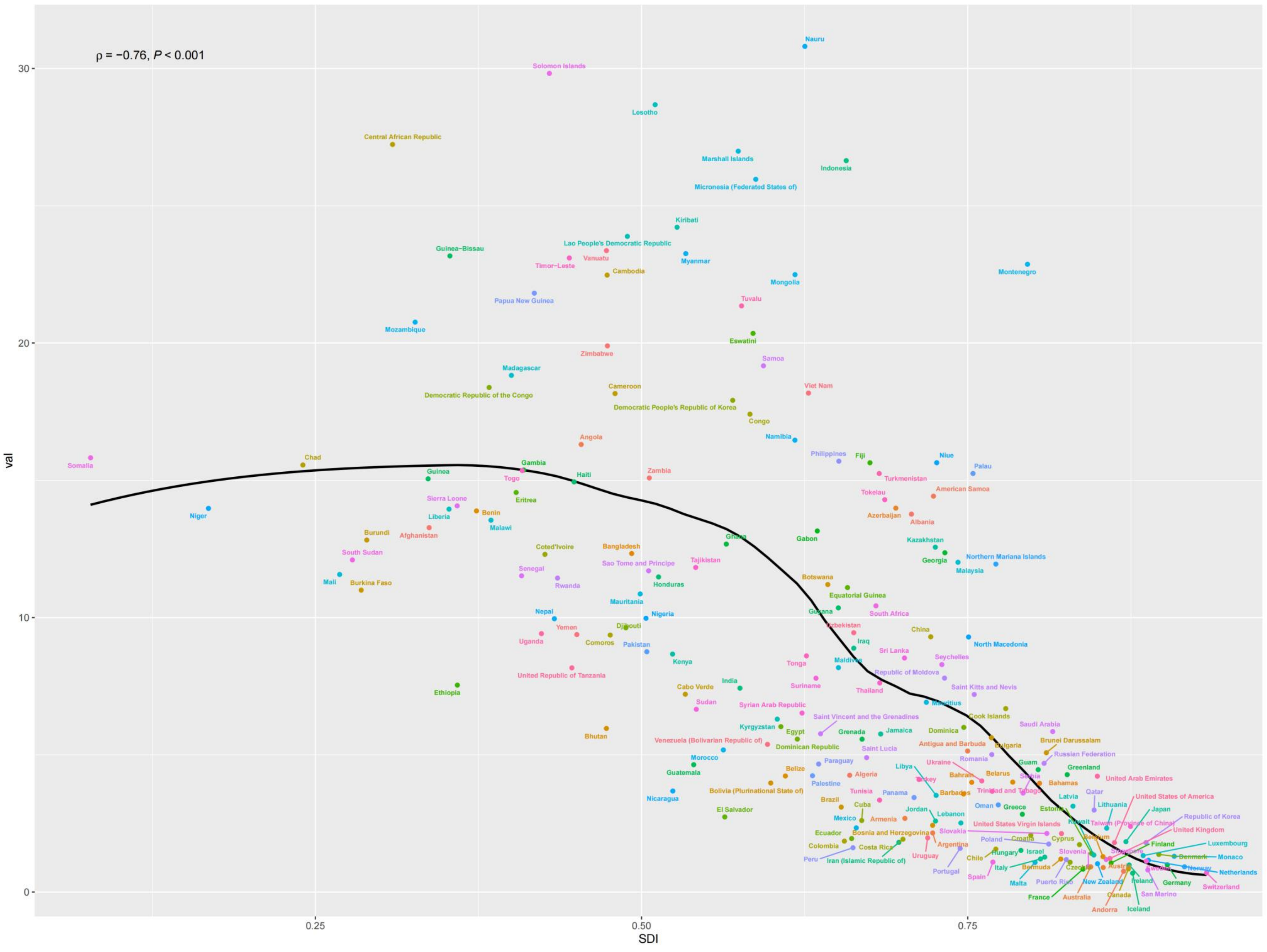

Fig. S21 The associations between age-standardized kidney dysfunction related intracerebral hemorrhage mortality rate and the sociodemographic index across 204 nations and territories.

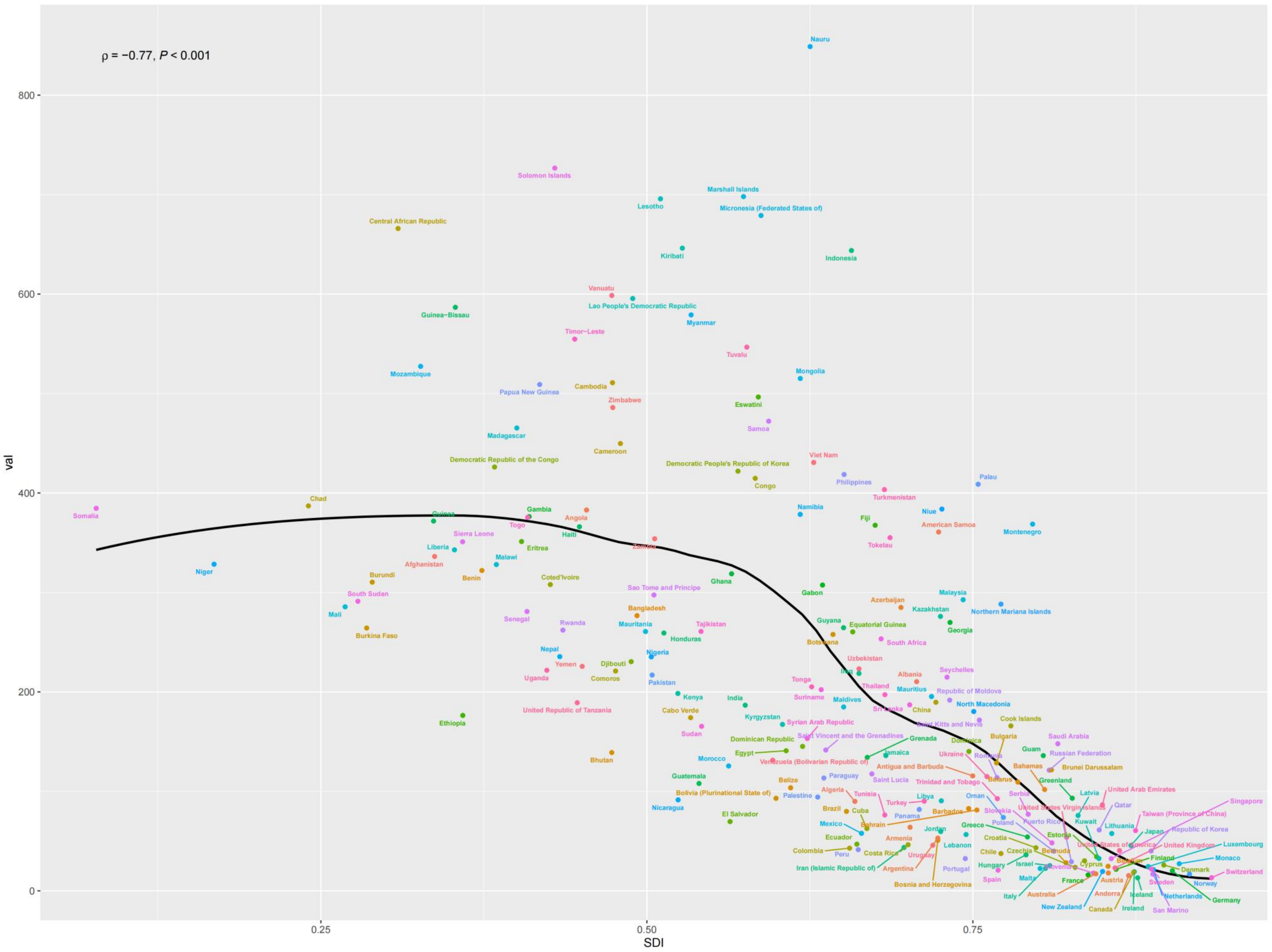

Fig. S22 The associations between age-standardized kidney dysfunction related intracerebral hemorrhage disability-adjusted life years rate and the sociodemographic index across 204 nations and territories.

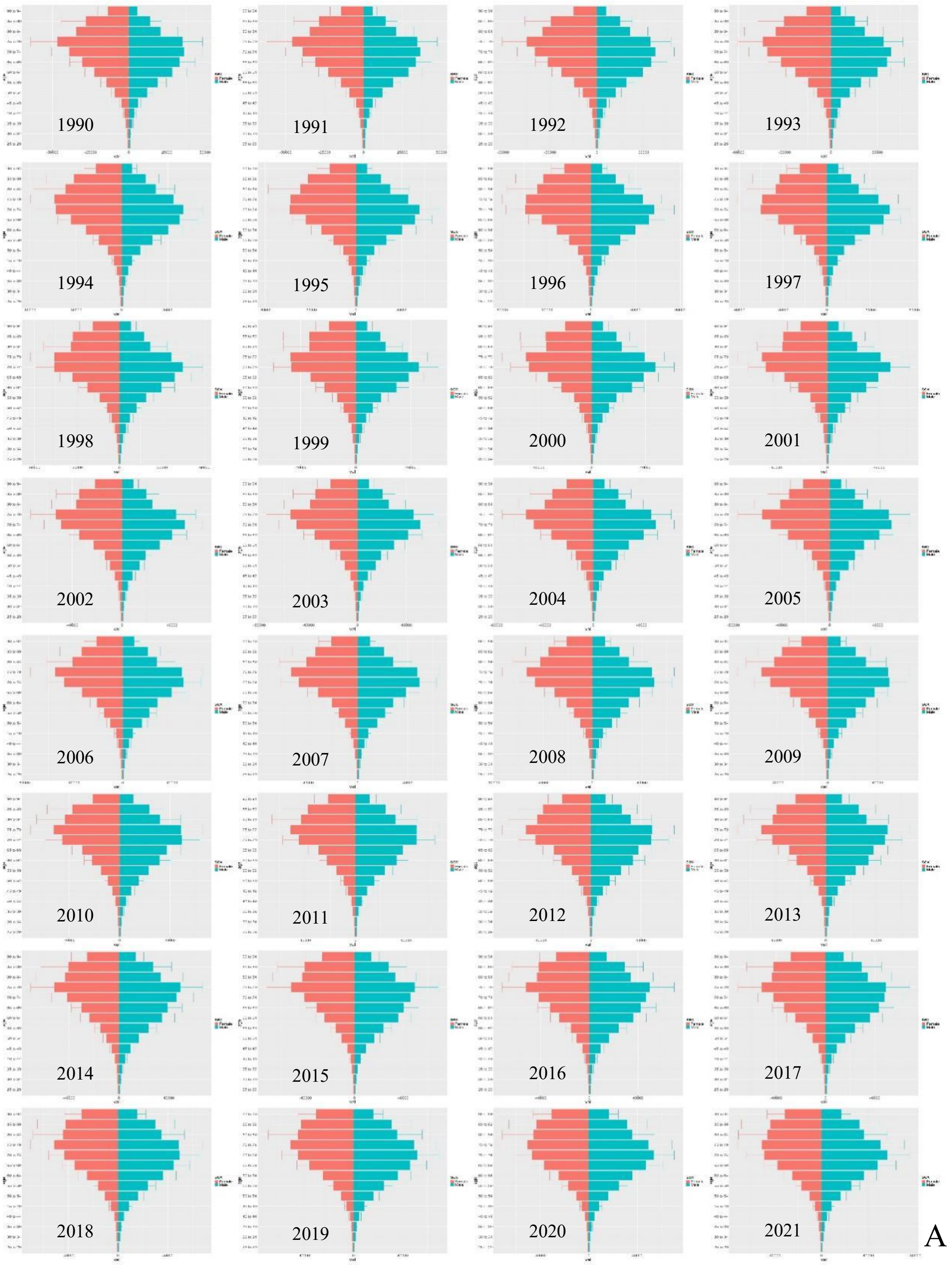
1990
1991
1992
1993
1994
1995
1996
1997
1998
1999
2000
2001
2002
2003
2004
2005
2006
2007
2008
2009
2010
2011
2012
2013
2014
2015
2016
2017
2018
2019
2020
2021
A

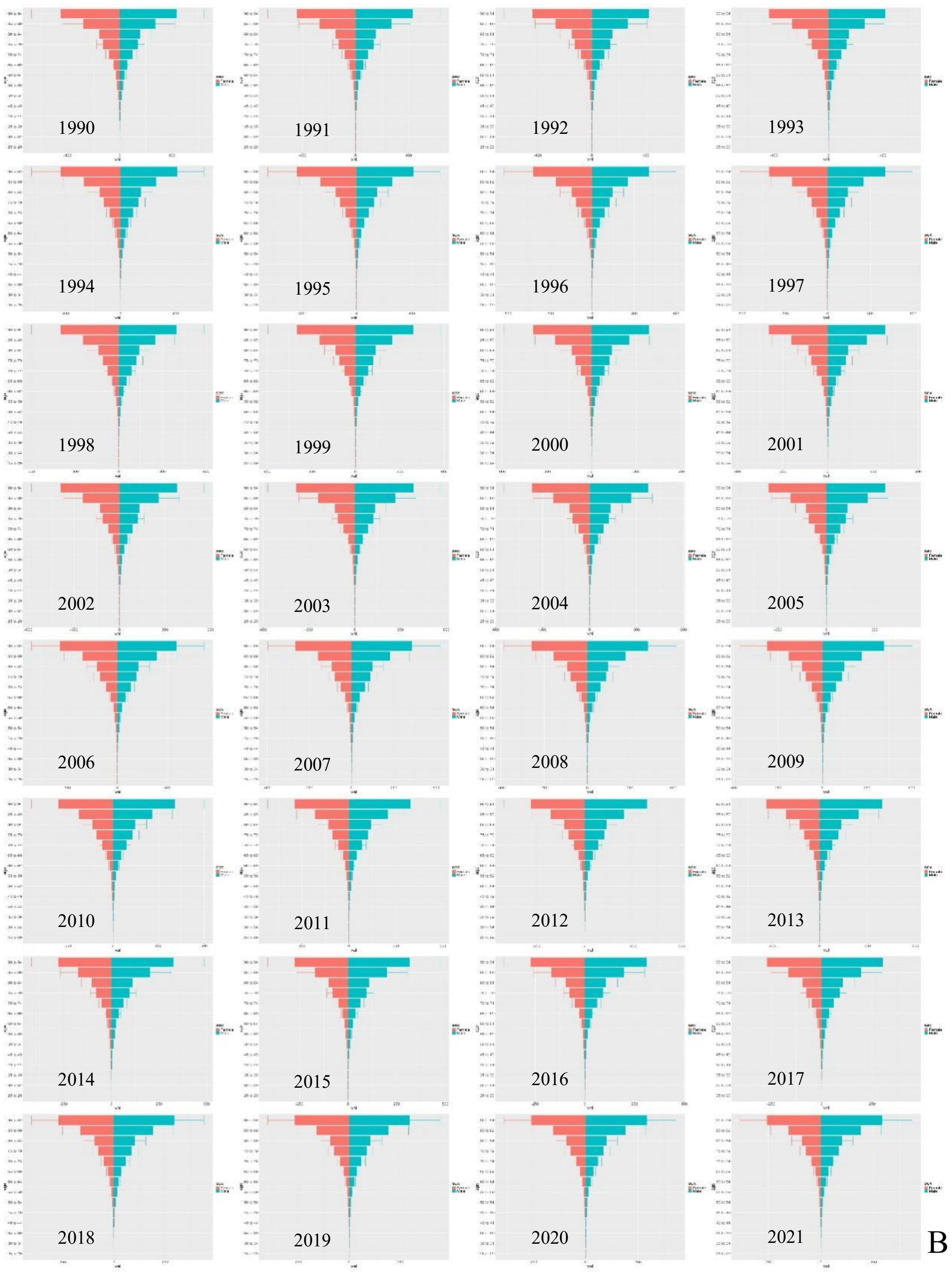

Fig. S23A The global disease burden of kidney dysfunction related stroke mortality cases in different ages from 1990 to 2021; B The global disease burden of kidney dysfunction related stroke mortality rate in different ages from 1990 to 2021. Notes: red for female, green for male; the ordinate from bottom to top is "25 to 29", "30 to 34", "35 to 39", "40 to 44", "45 to 49", "50 to 54", "55 to 59", "60 to 64", "65 to 69", "70 to 74", "75 to 79", "80 to 84", "85 to 89", "90 to 94".

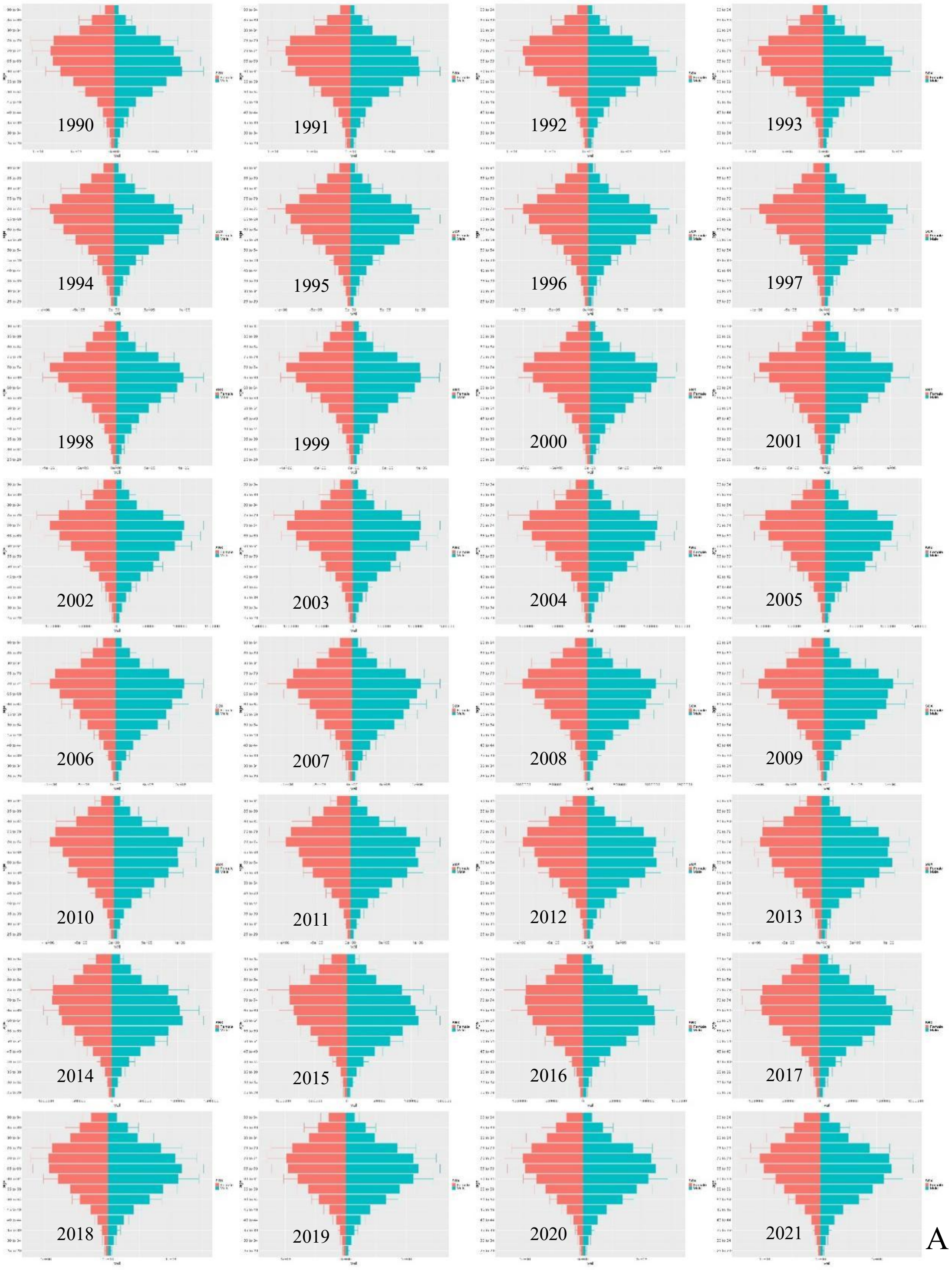
1990
1991
1992
1993
1994
1995
1996
1997
1998
1999
2000
2001
2002
2003
2004
2005
2006
2007
2008
2009
2010
2011
2012
2013
2014
2015
2016
2017
2018
2019
2020
2021
A

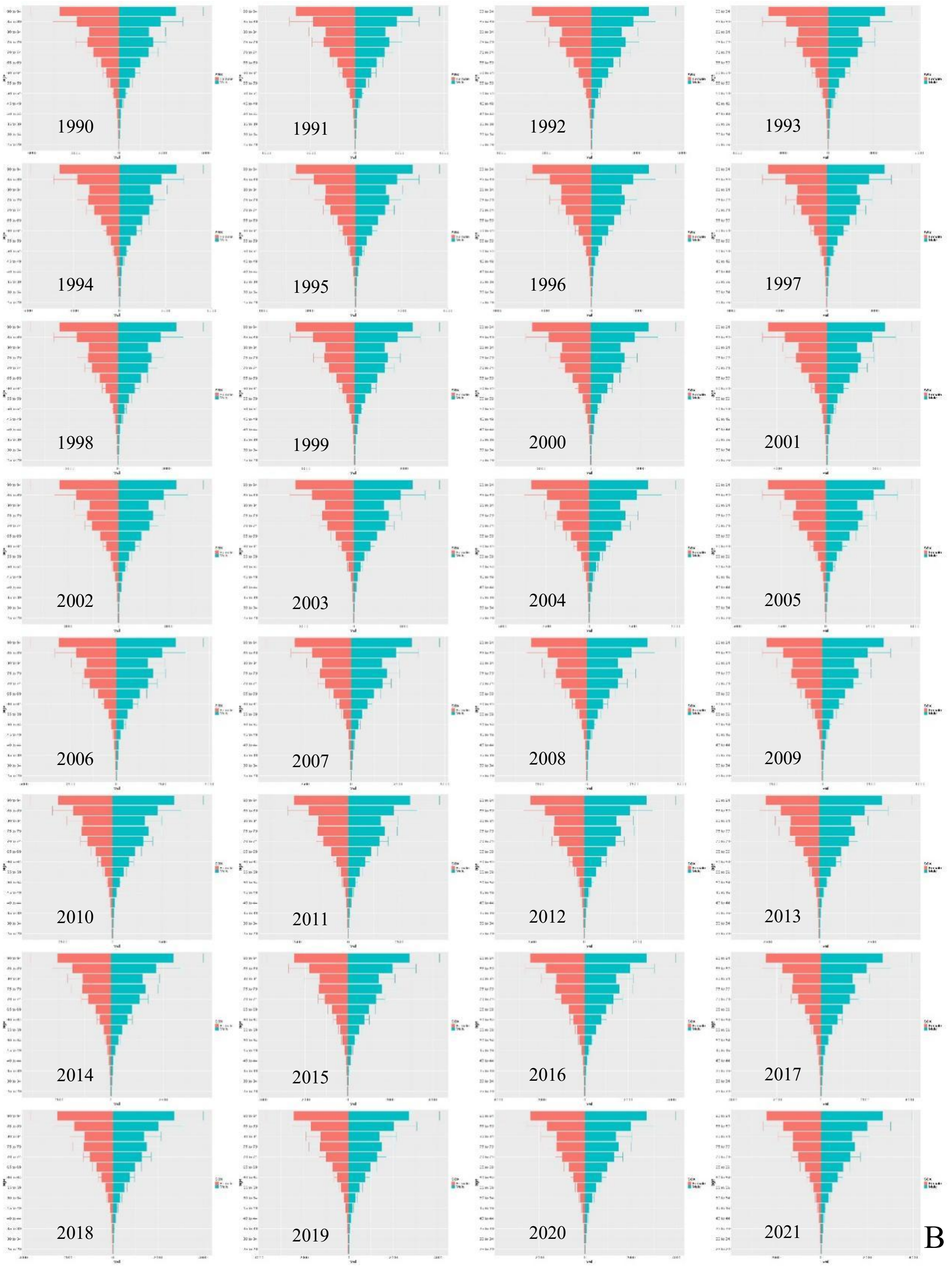

Fig. S24A The global disease burden of kidney dysfunction related stroke disability-adjusted life years in different ages from 1990 to 2021; B The global disease burden of kidney dysfunction related stroke disability-adjusted life years rate in different ages from 1990 to 2021. Notes: red for female, green for male; the ordinate from bottom to top is "25 to 29", "30 to 34", "35 to 39", "40 to 44", "45 to 49", "50 to 54", "55 to 59", "60 to 64", "65 to 69", "70 to 74", "75 to 79", "80 to 84", "85 to 89", "90 to 94".

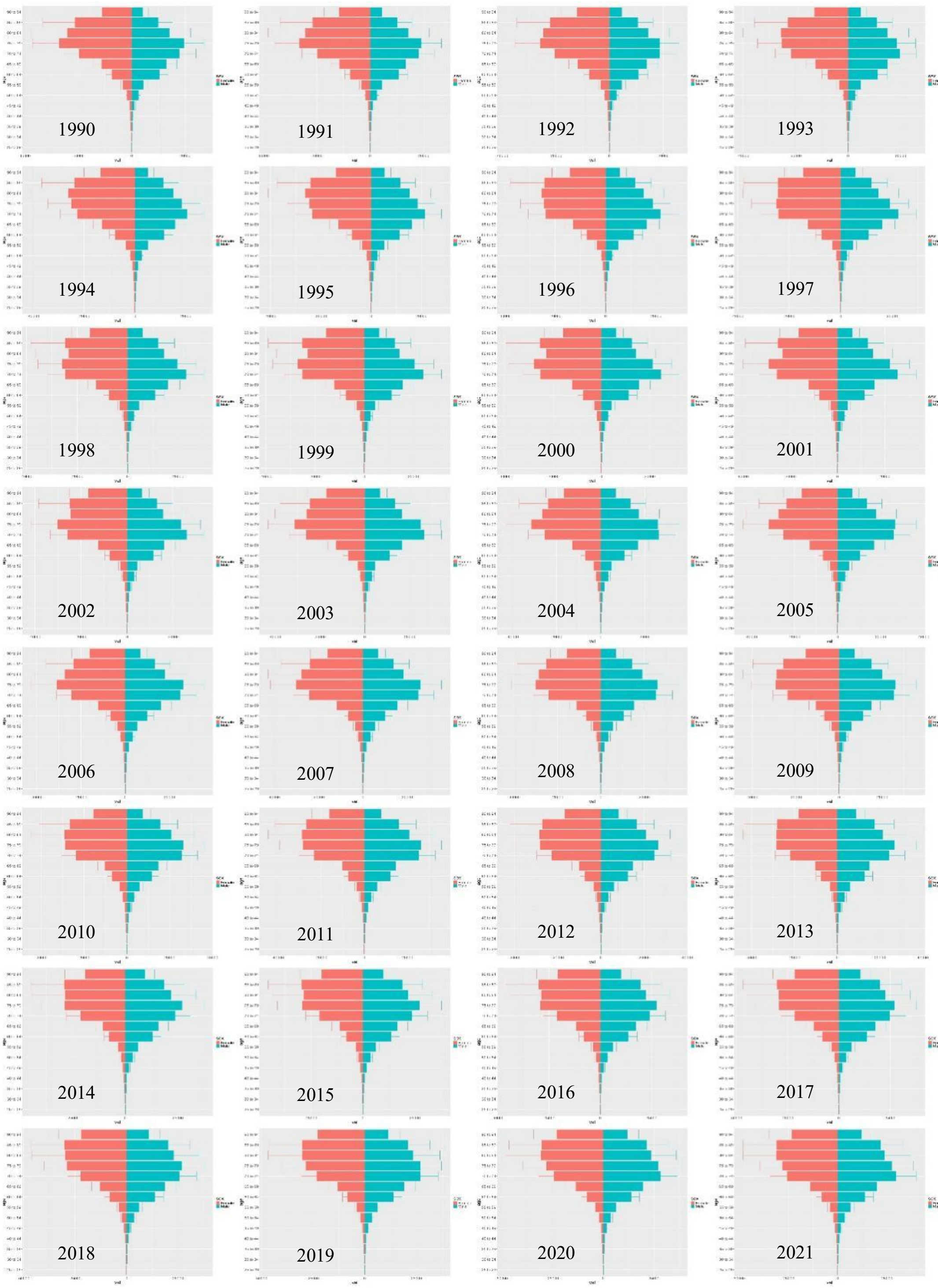
1990
1991
1992
1993
1994
1995
1996
1997
1998
1999
2000
2001
2002
2003
2004
2005
2006
2007
2008
2009
2010
2011
2012
2013
2014
2015
2016
2017
2018
2019
2020
2021

A

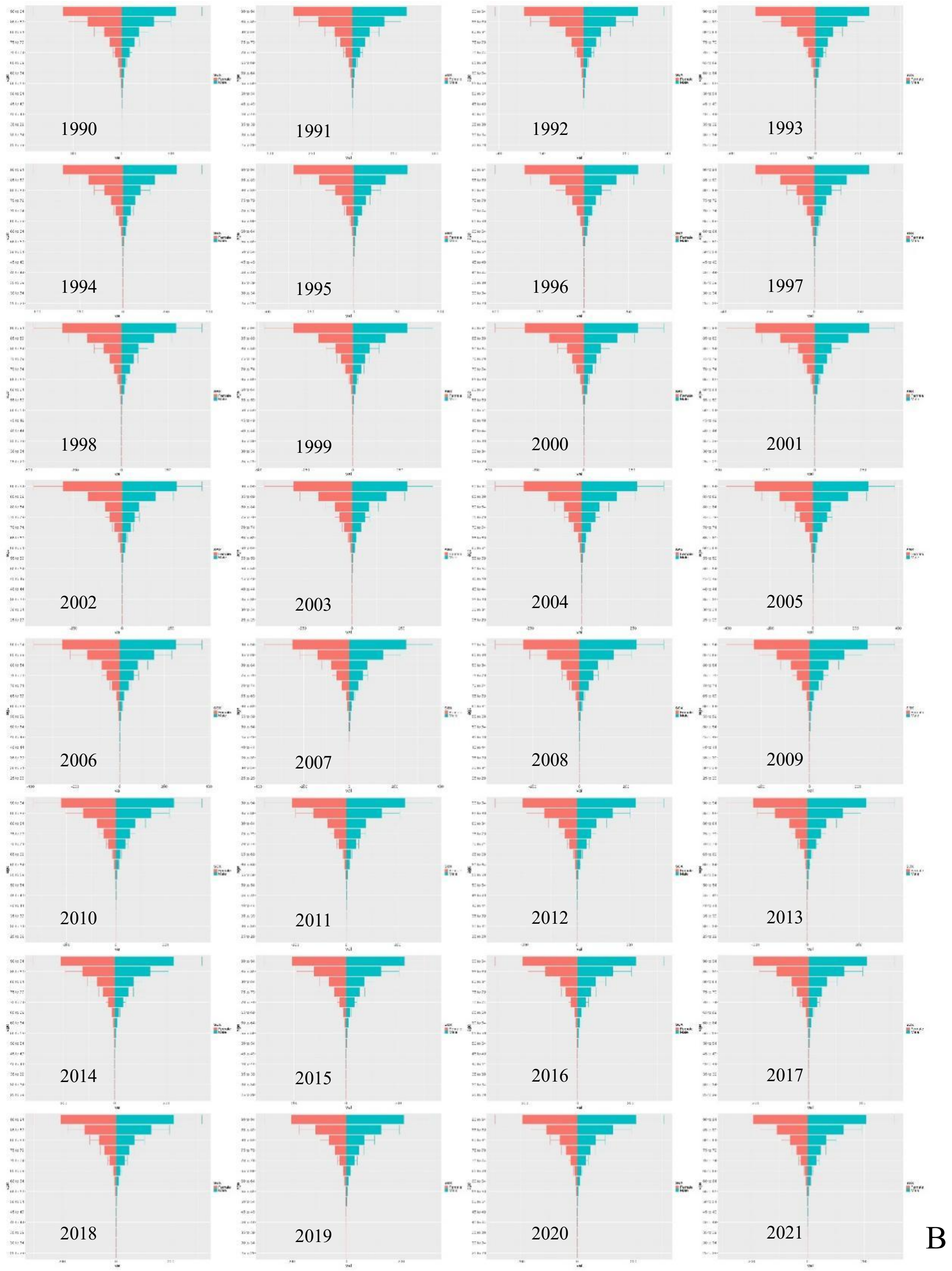

Fig. S25A The global disease burden of kidney dysfunction related ischemic stroke mortality cases in different ages from 1990 to 2021; B The global disease burden of kidney dysfunction related ischemic stroke mortality rate in different ages from 1990 to 2021. Notes: red for female, green for male; the ordinate from bottom to top is "25 to 29", "30 to 34", "35 to 39", "40 to 44", "45 to 49", "50 to 54", "55 to 59", "60 to 64", "65 to 69", "70 to 74", "75 to 79", "80 to 84", "85 to 89", "90 to 94".

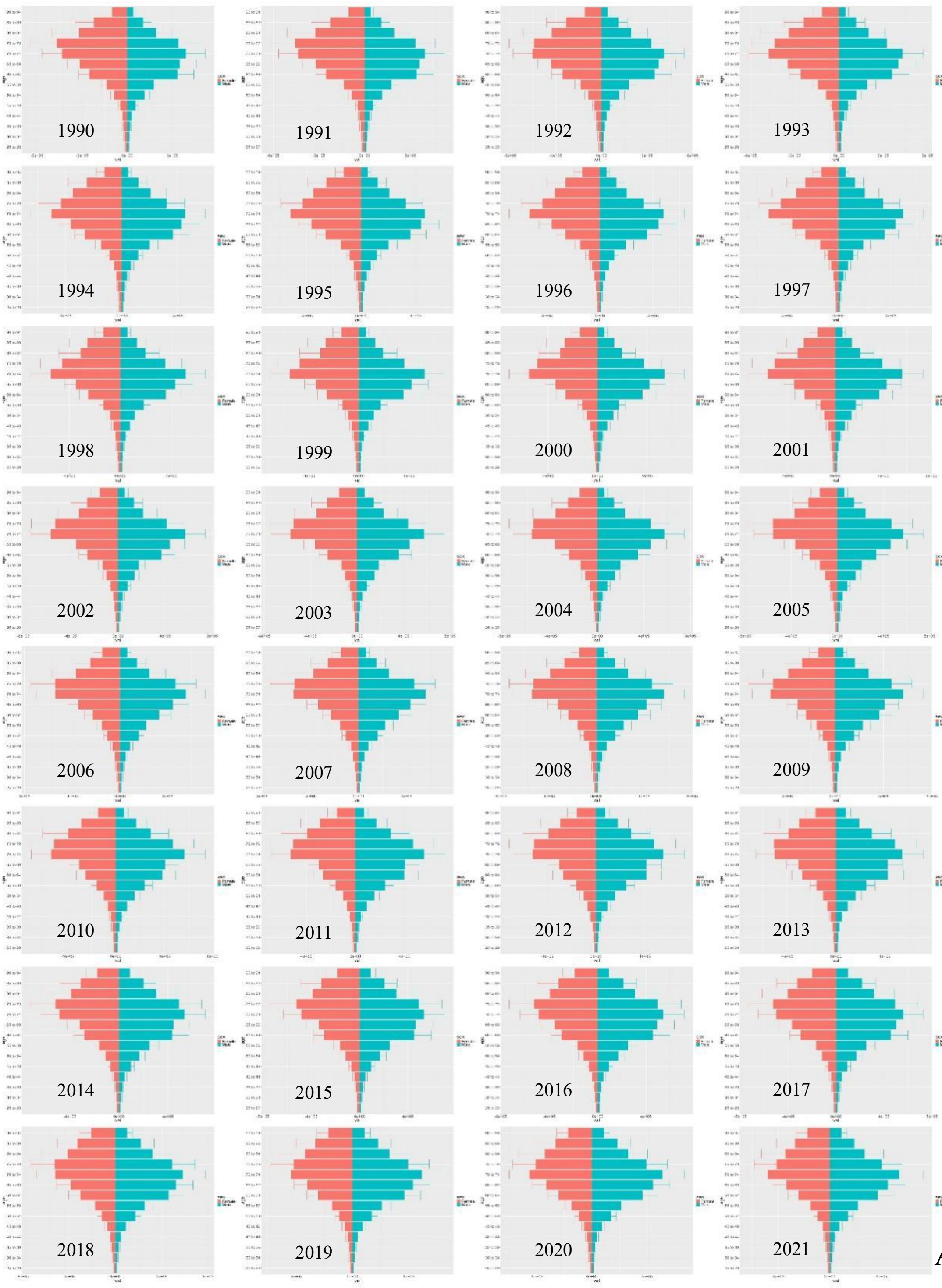
1990
1991
1992
1993
1994
1995
1996
1997
1998
1999
2000
2001
2002
2003
2004
2005
2006
2007
2008
2009
2010
2011
2012
2013
2014
2015
2016
2017
2018
2019
2020
2021

A

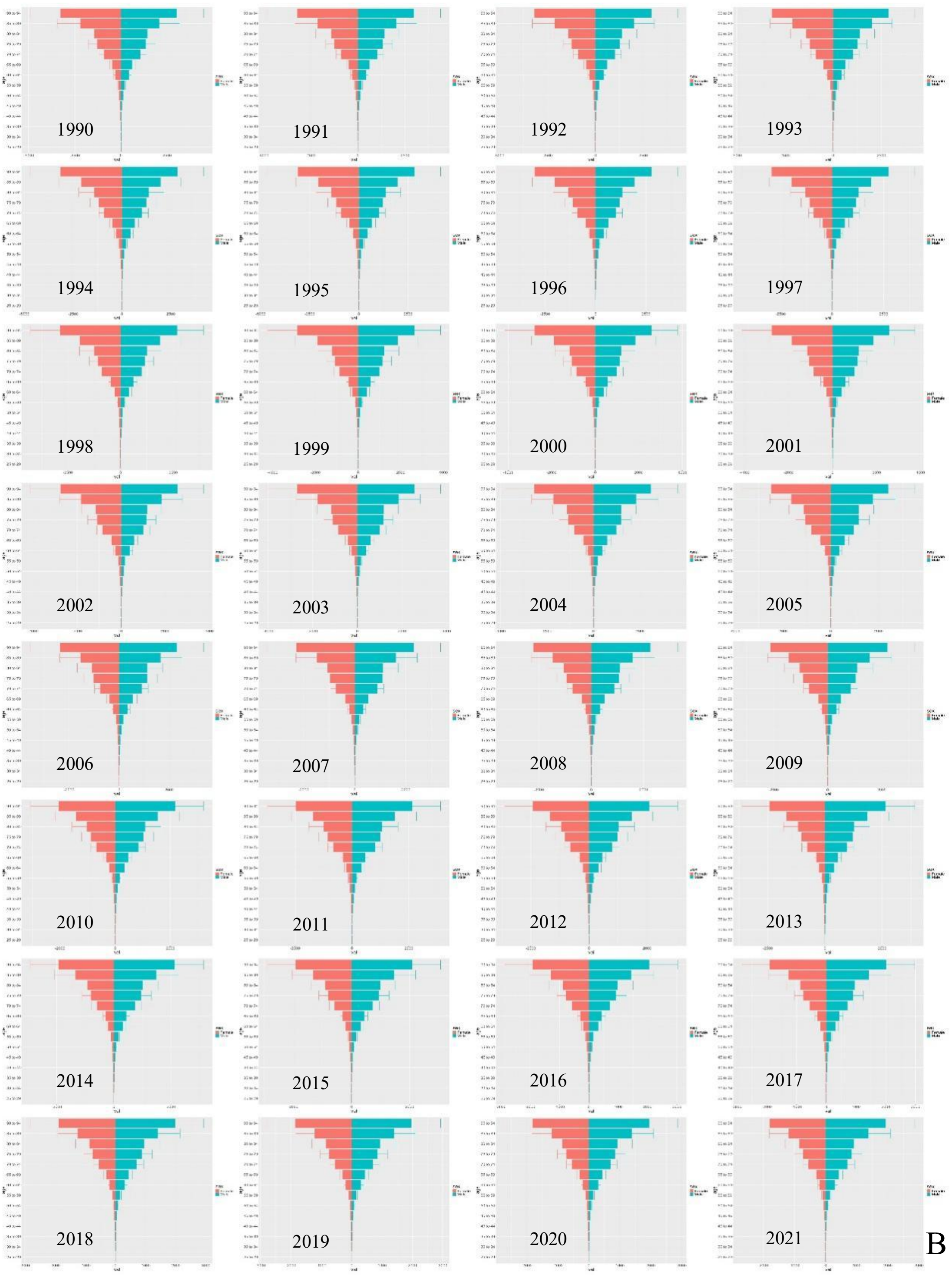

Fig. S26A The global disease burden of kidney dysfunction related ischemic stroke disability-adjusted life years in different ages from 1990 to 2021; B The global disease burden of kidney dysfunction related ischemic stroke disability-adjusted life years rate in different ages from 1990 to 2021. Notes: red for female, green for male; the ordinate from bottom to top is "25 to 29", "30 to 34", "35 to 39", "40 to 44", "45 to 49", "50 to 54", "55 to 59", "60 to 64", "65 to 69", "70 to 74", "75 to 79", "80 to 84", "85 to 89", "90 to 94".

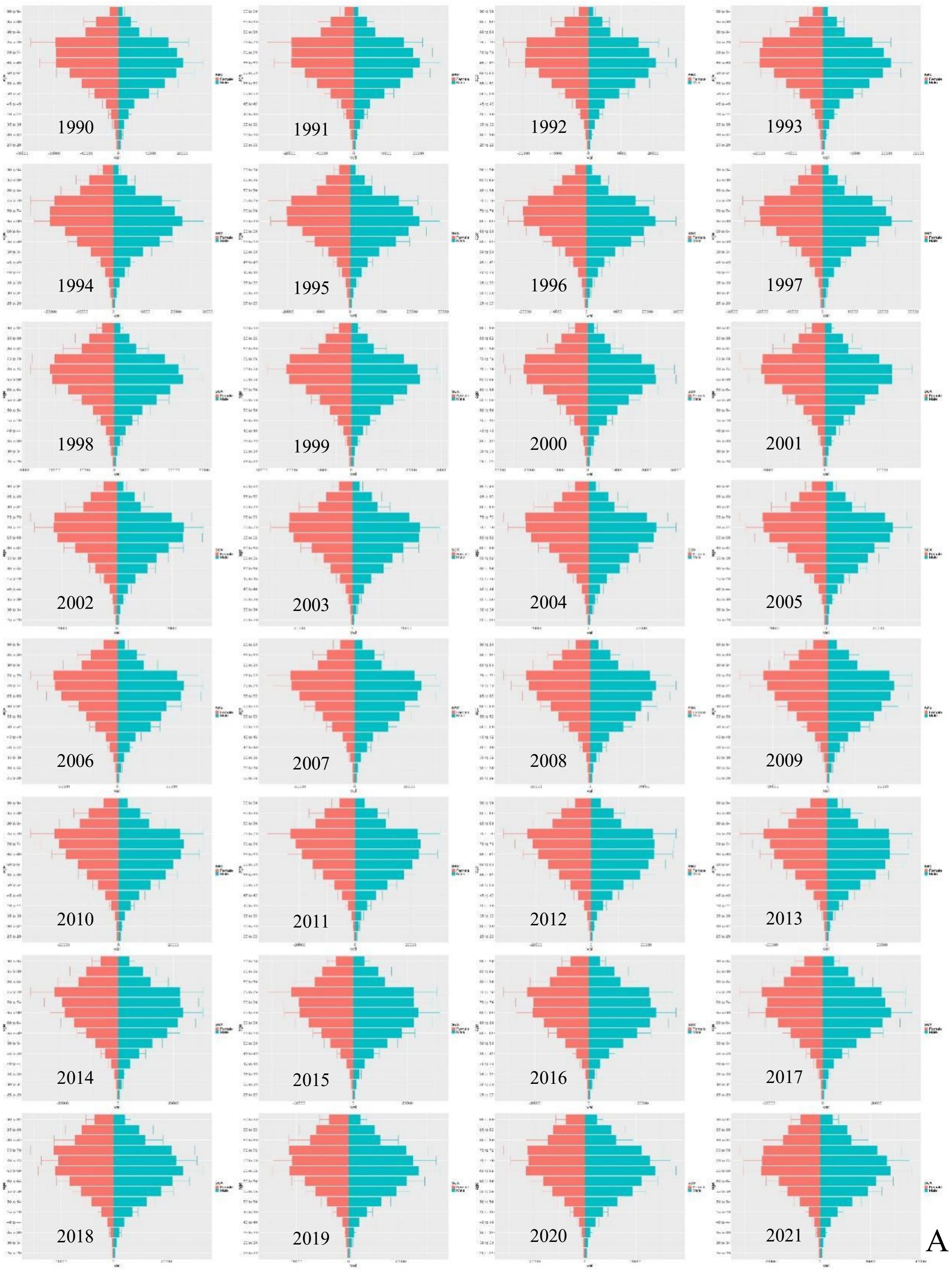
1990
1991
1992
1993
1994
1995
1996
1997
1998
1999
2000
2001
2002
2003
2004
2005
2006
2007
2008
2009
2010
2011
2012
2013
2014
2015
2016
2017
2018
2019
2020
2021
A

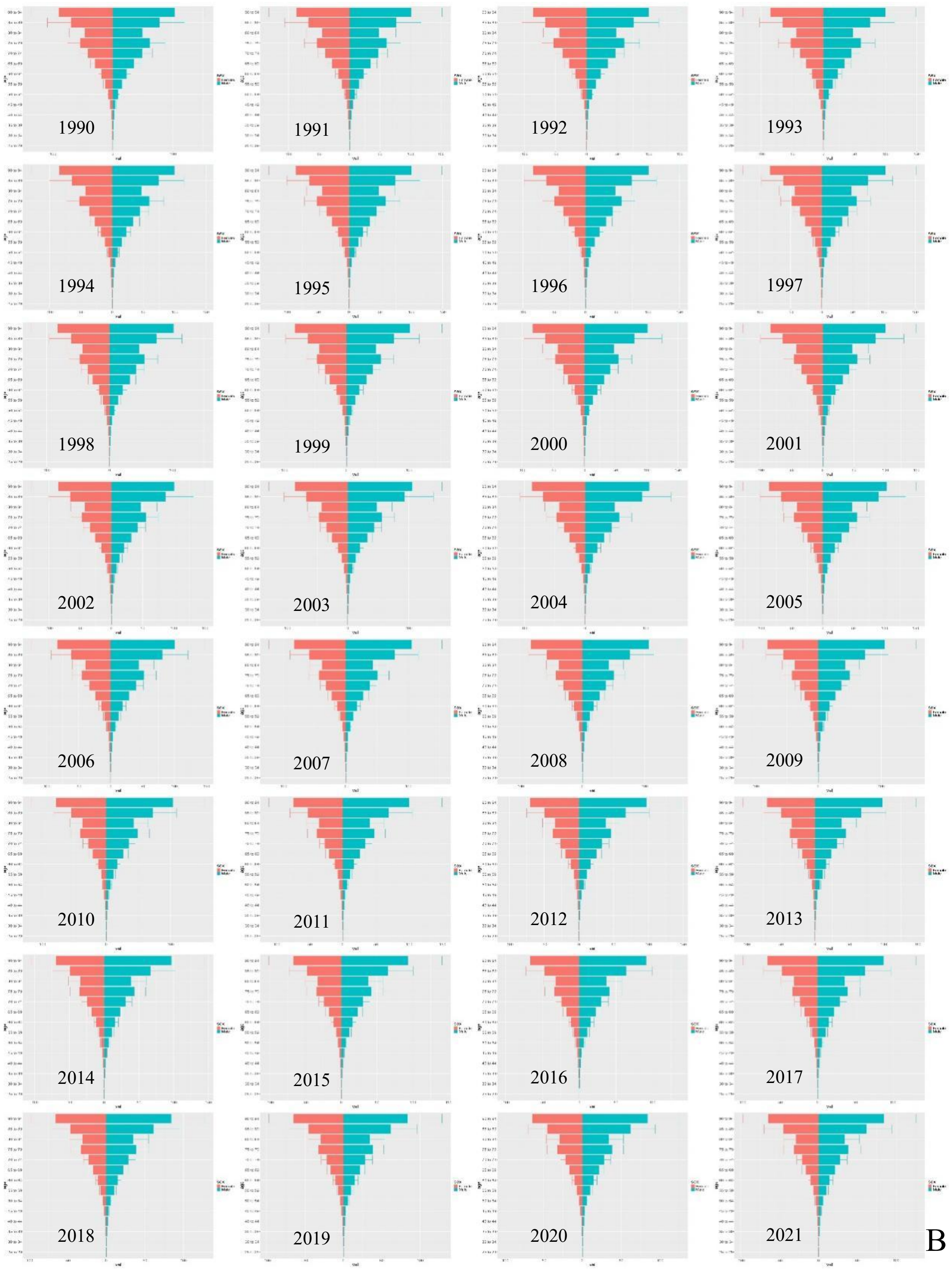

Fig. S27A The global disease burden of kidney dysfunction related intracerebral hemorrhage mortality cases in different ages from 1990 to 2021; B The global disease burden of kidney dysfunction related intracerebral hemorrhage mortality rate in different ages from 1990 to 2021. Notes: red for female, green for male; the ordinate from bottom to top is "25 to 29", "30 to 34", "35 to 39", "40 to 44", "45 to 49", "50 to 54", "55 to 59", "60 to 64", "65 to 69", "70 to 74", "75 to 79", "80 to 84", "85 to 89", "90 to 94".

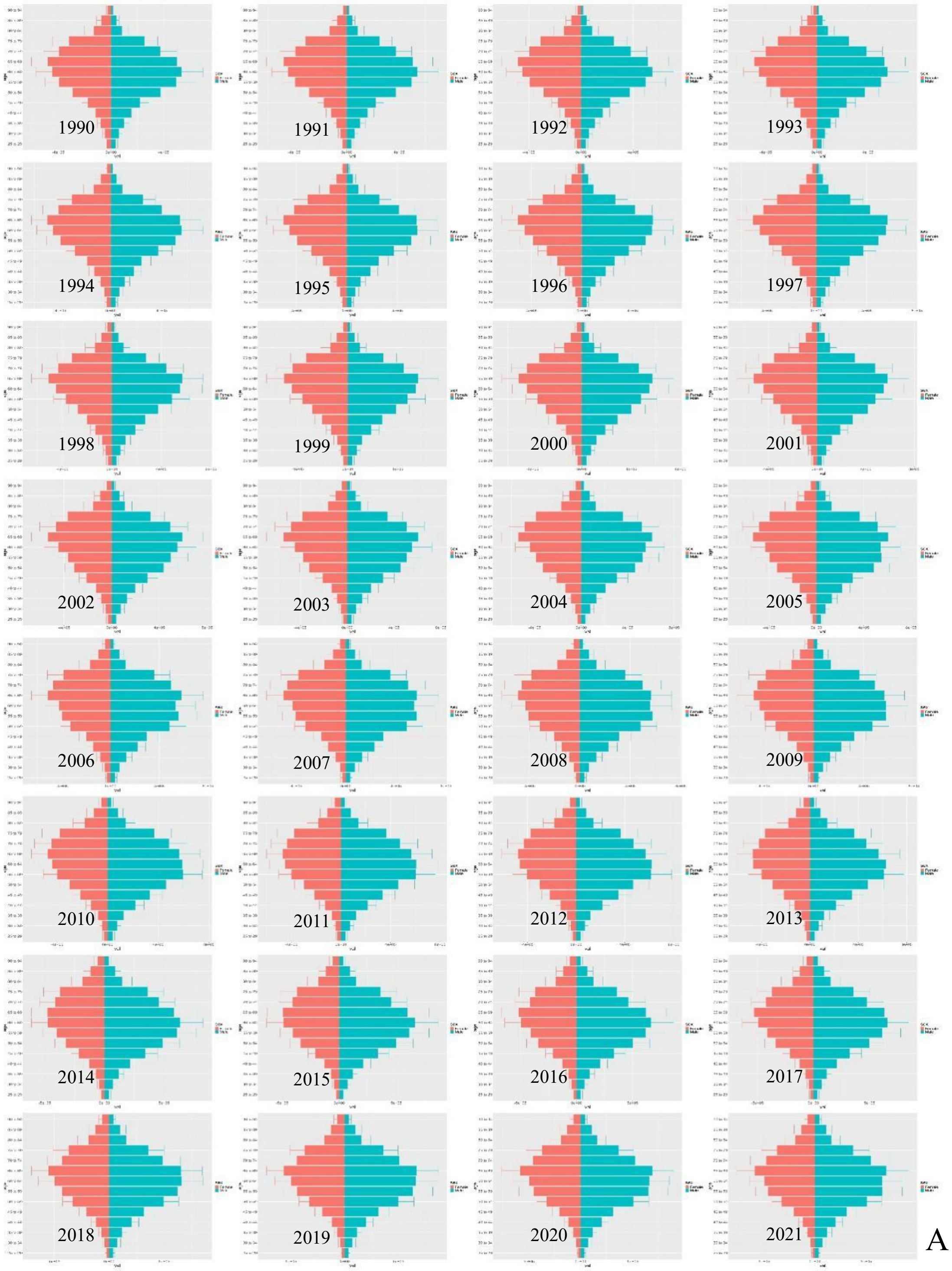
1990
1991
1992
1993
1994
1995
1996
1997
1998
1999
2000
2001
2002
2003
2004
2005
2006
2007
2008
2009
2010
2011
2012
2013
2014
2015
2016
2017
2018
2019
2020
2021
A

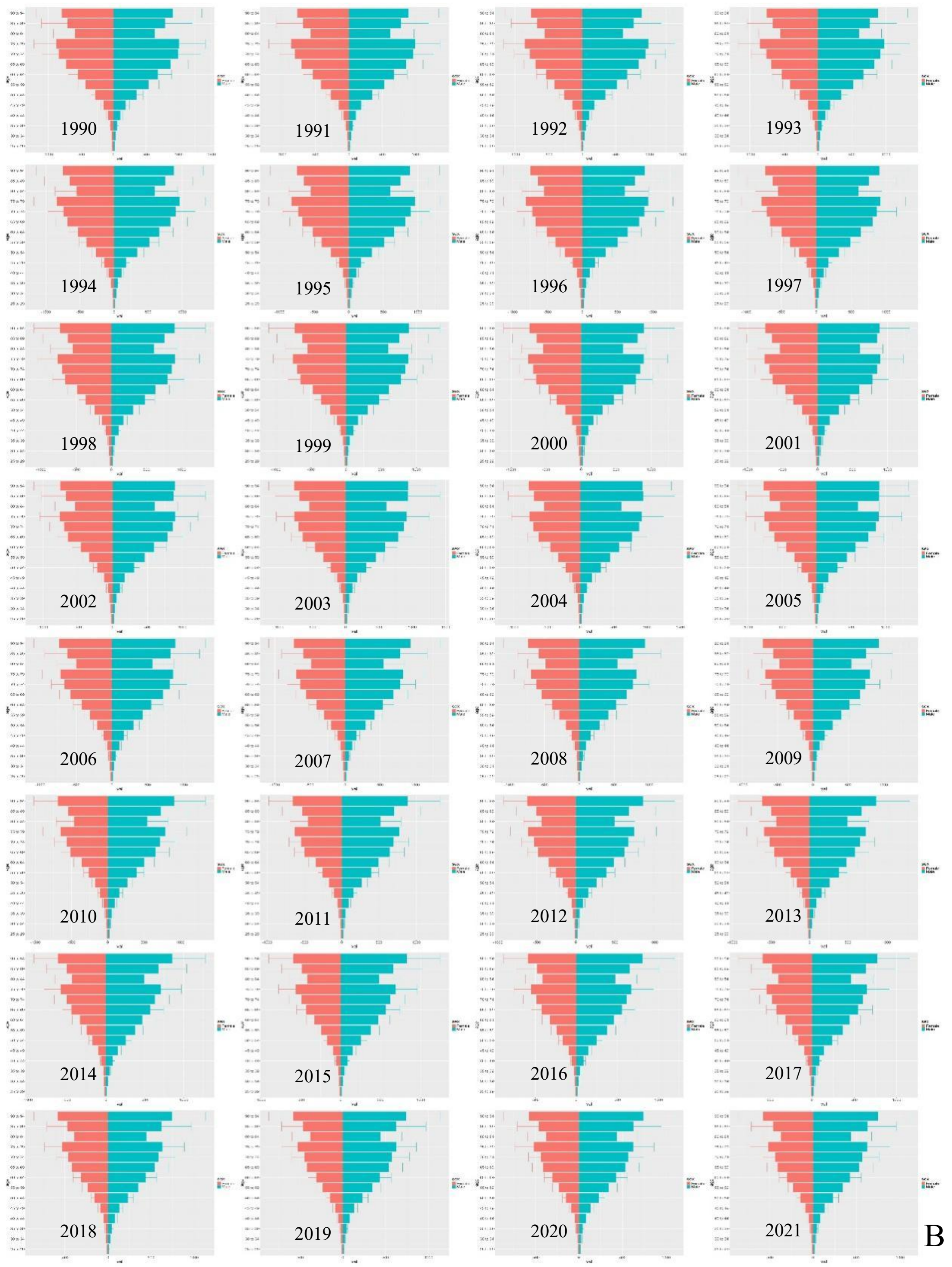

Fig. S28A The global disease burden of kidney dysfunction related intracerebral hemorrhage disability-adjusted life years in different ages from 1990 to 2021; B The global disease burden of kidney dysfunction related intracerebral hemorrhage disability-adjusted life years rate in different ages from 1990 to 2021. Notes: red for female, green for male; the ordinate from bottom to top is "25 to 29", "30 to 34", "35 to 39", "40 to 44", "45 to 49", "50 to 54", "55 to 59", "60 to 64", "65 to 69", "70 to 74", "75 to 79", "80 to 84", "85 to 89", "90 to 94".

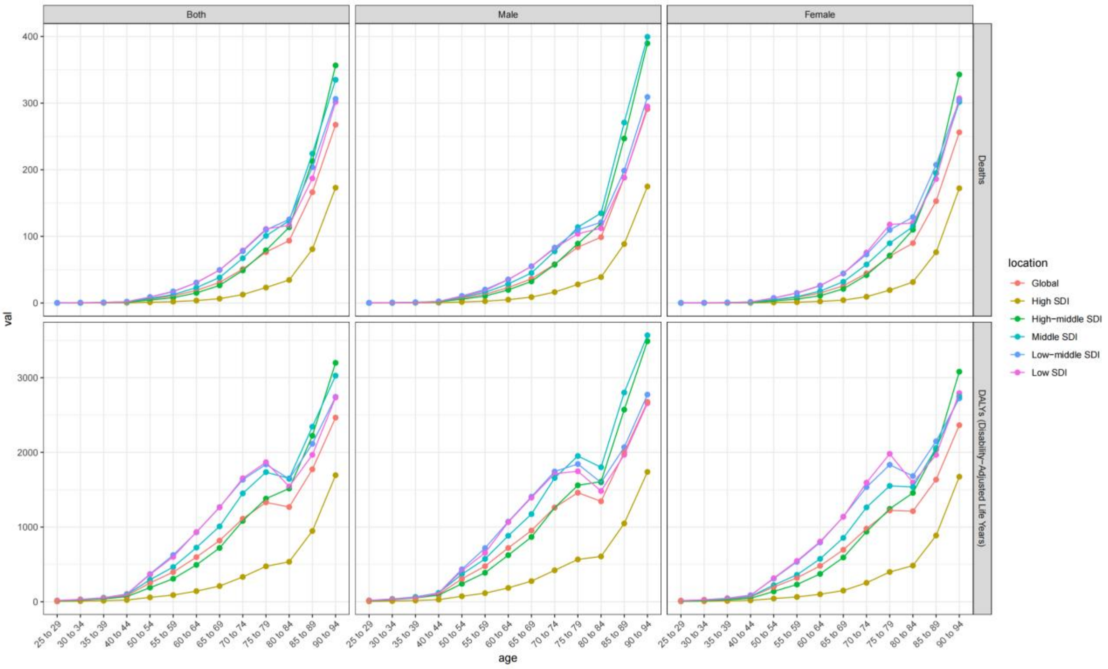

Fig. S29 The global disease burden of kidney dysfunction related stroke mortality and disability-adjusted life years rates in different ages.

Note: SDI: socio-demographic index.

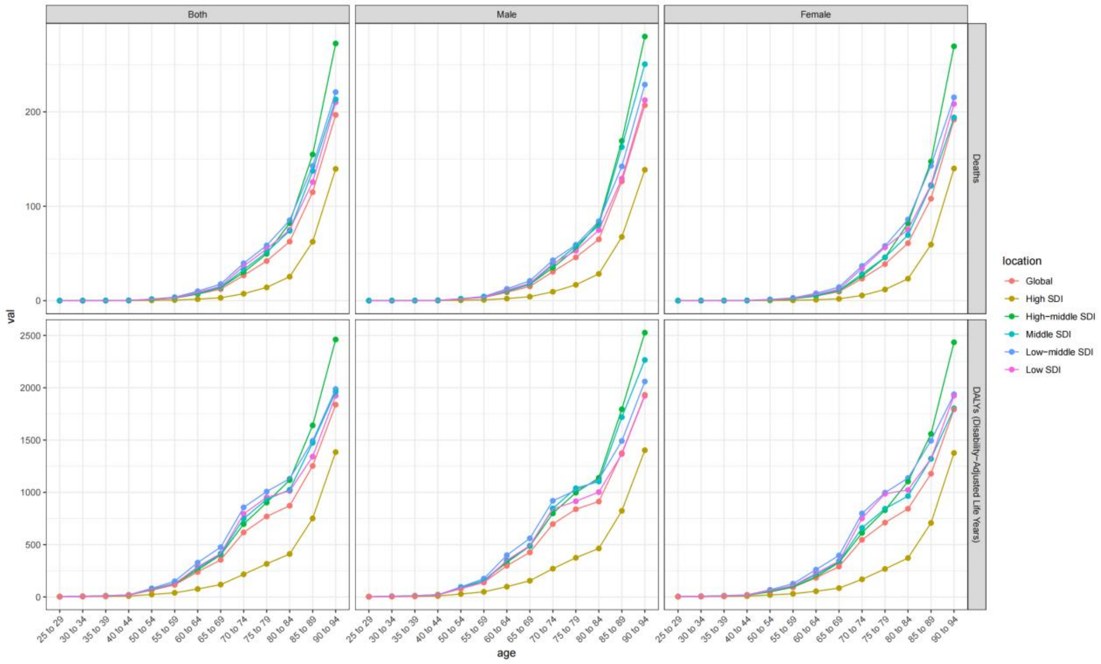

Fig. S30 The global disease burden of kidney dysfunction related ischemic stroke mortality and disability-adjusted life years rates in different ages.

Note: SDI: socio-demographic index.

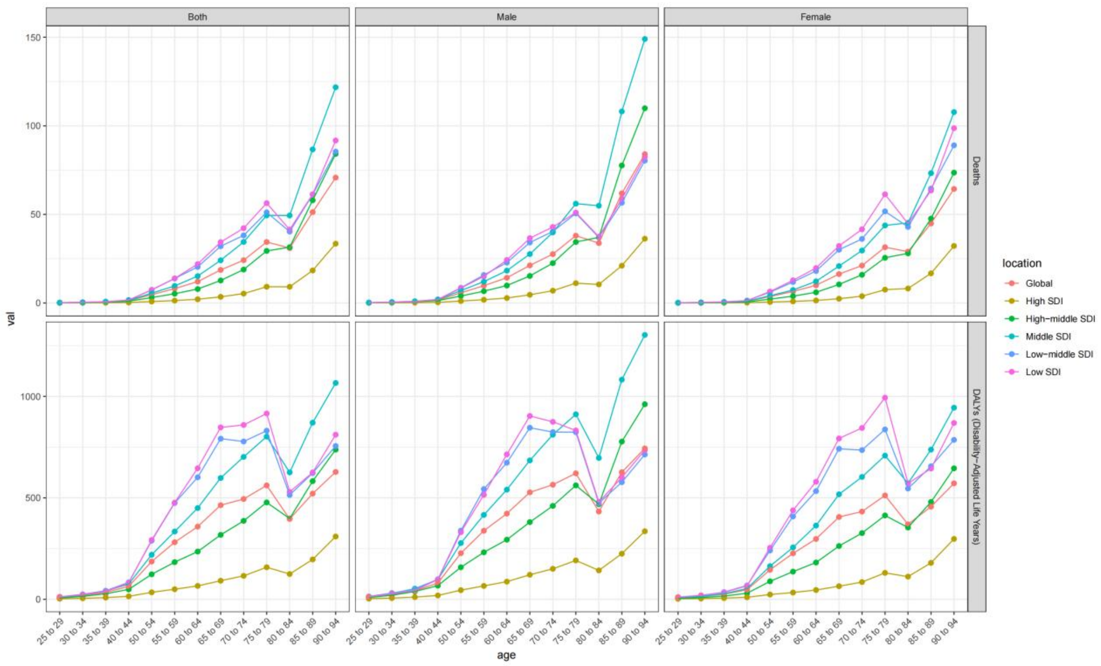

Fig. S31 The global disease burden of kidney dysfunction related intracerebral hemorrhage mortality and disability-adjusted life years rates in different ages.

Note: SDI: socio-demographic index.

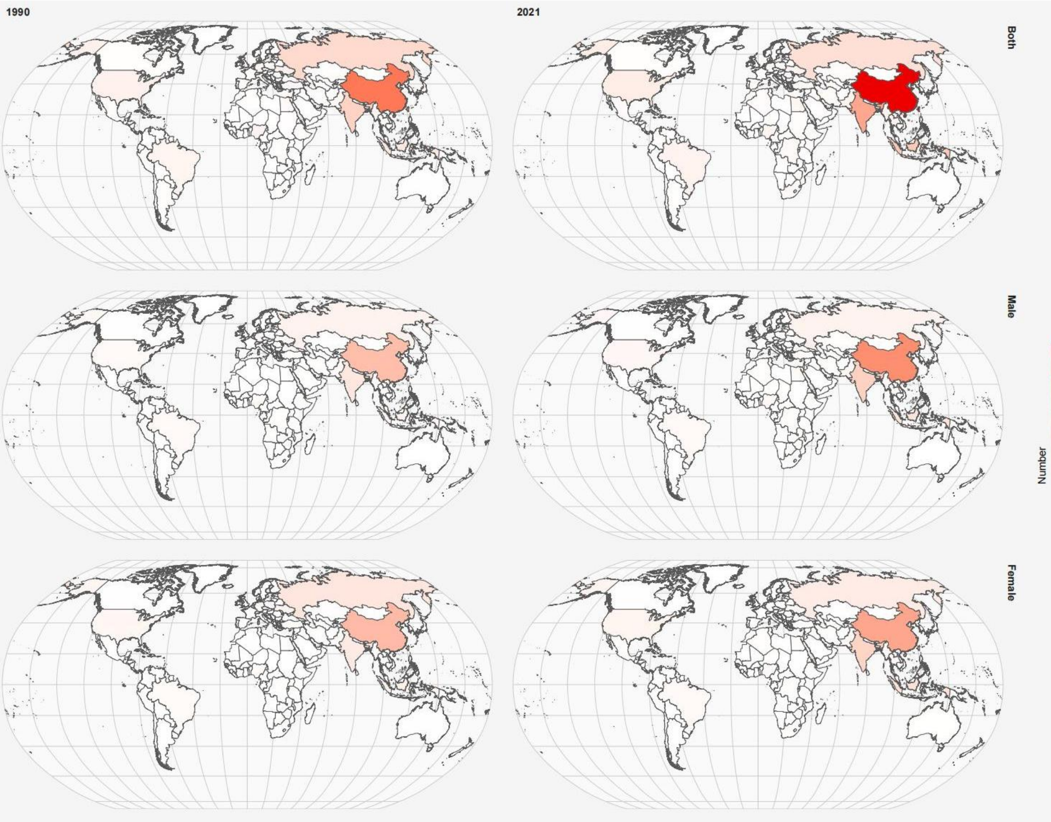
1990
2021
Both
Male
Female
150,000
100,000
50,000
Number
A

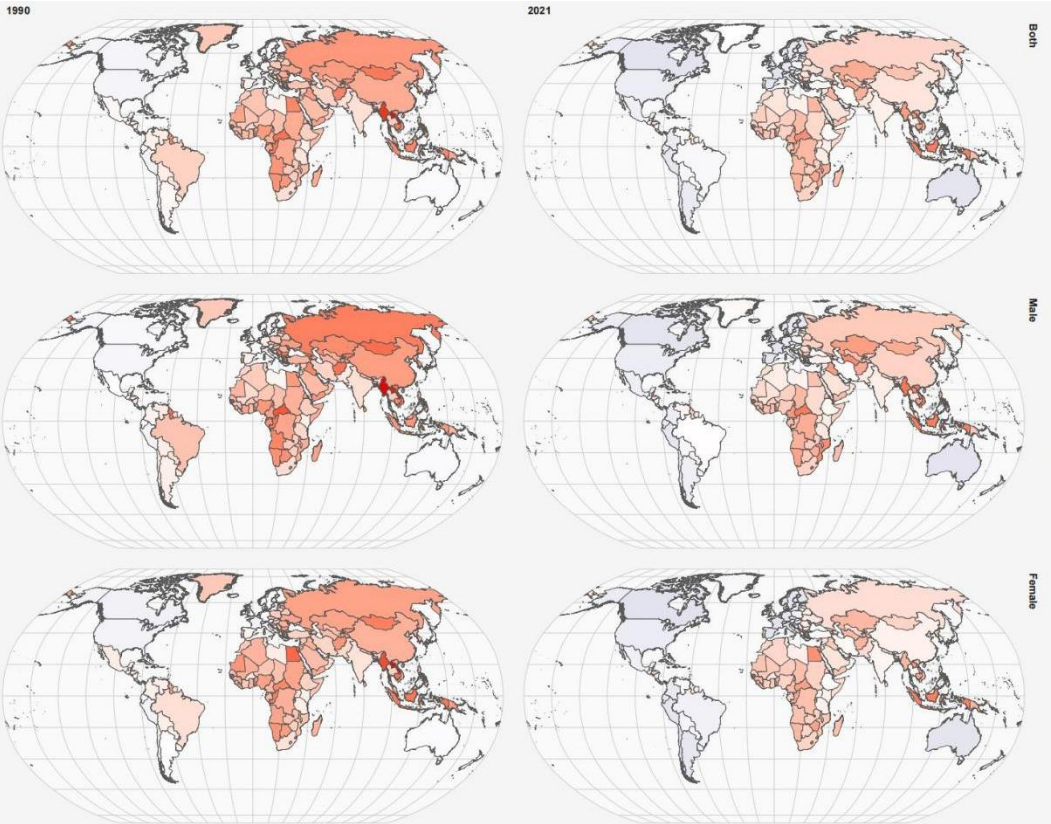
1990
2021
Both
Male
Female
60
40
20
ASR (per 100,000)
B

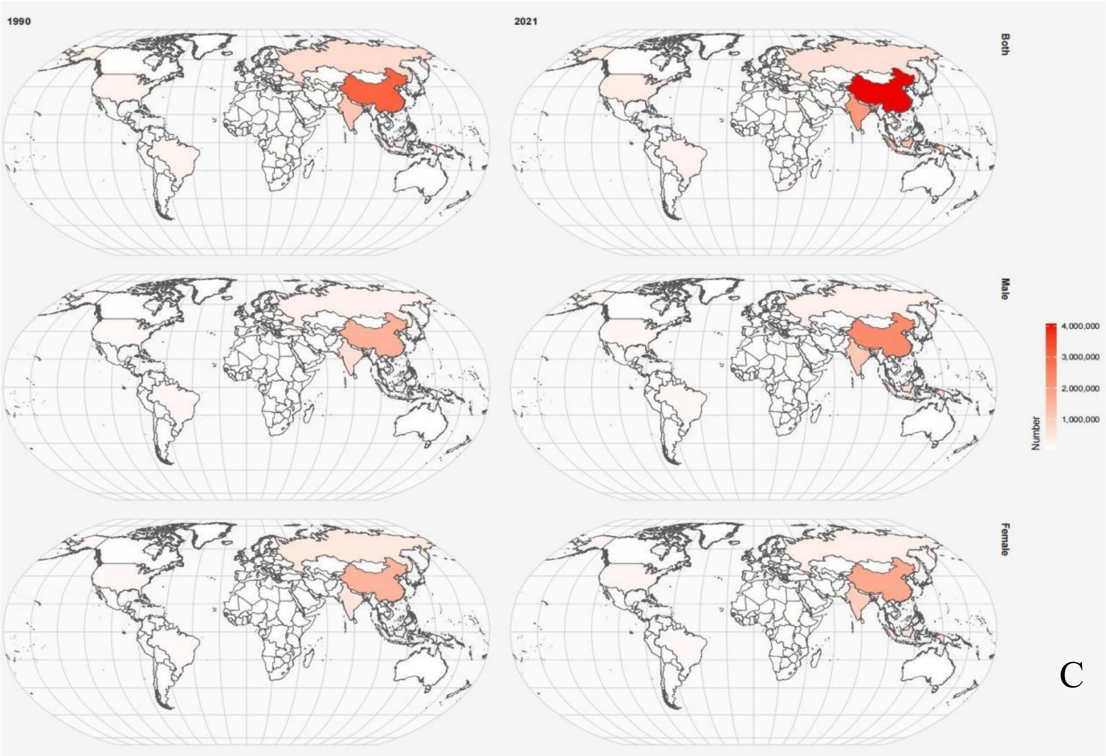

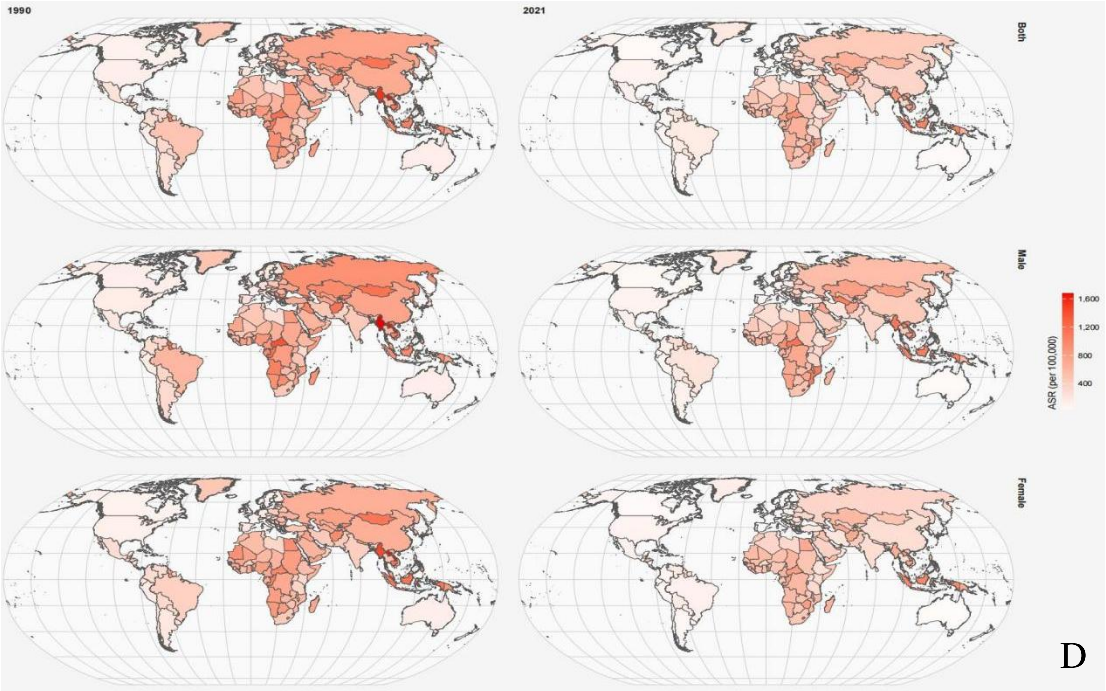

Fig. S32A Comparison of the global disease burden of kidney dysfunction related stroke mortality in males and females across 204 countries and territories between 1990 and 2021; B Comparison of the global disease burden of kidney dysfunction related stroke mortality rate in males and females across 204 countries and territories between 1990 and 2021; C Comparison of the global disease burden of kidney dysfunction related stroke disability-adjusted life years in males and females across 204 countries and territories between 1990 and 2021; D Comparison of the global disease burden of kidney dysfunction related stroke disability-adjusted life years rate in males and females across 204 countries and territories between 1990 and 2021.

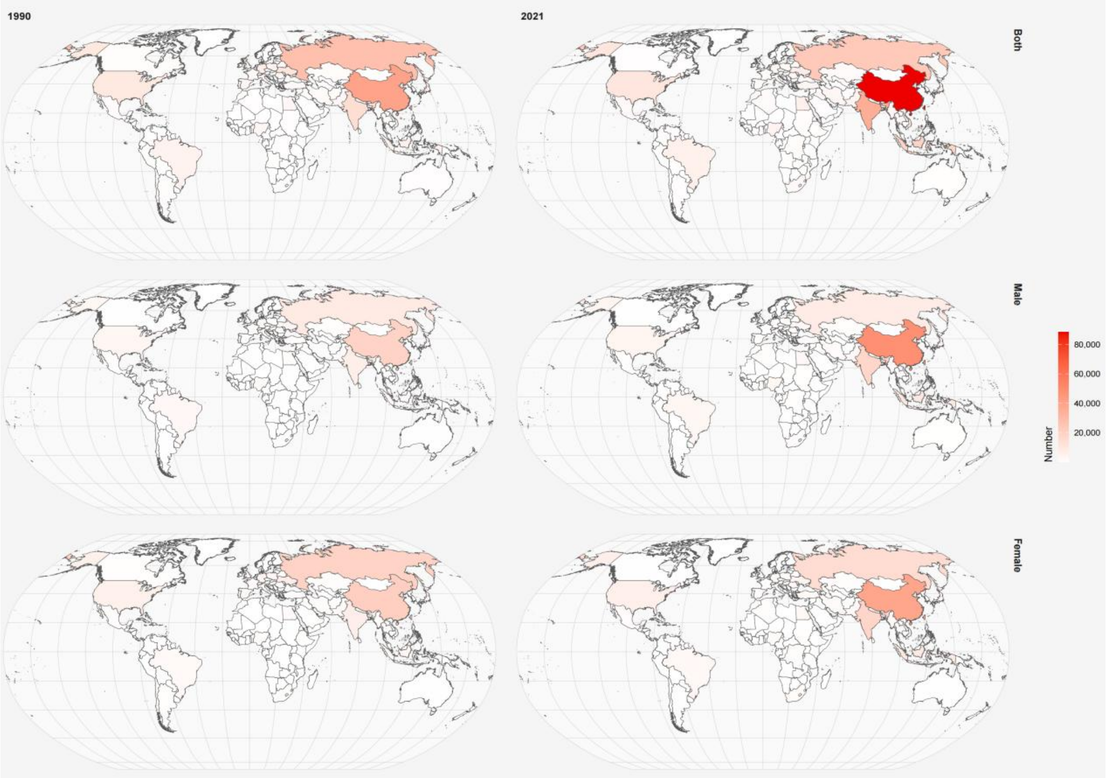
1990
2021
Both
Male
Female
80,000
60,000
40,000
20,000
Number

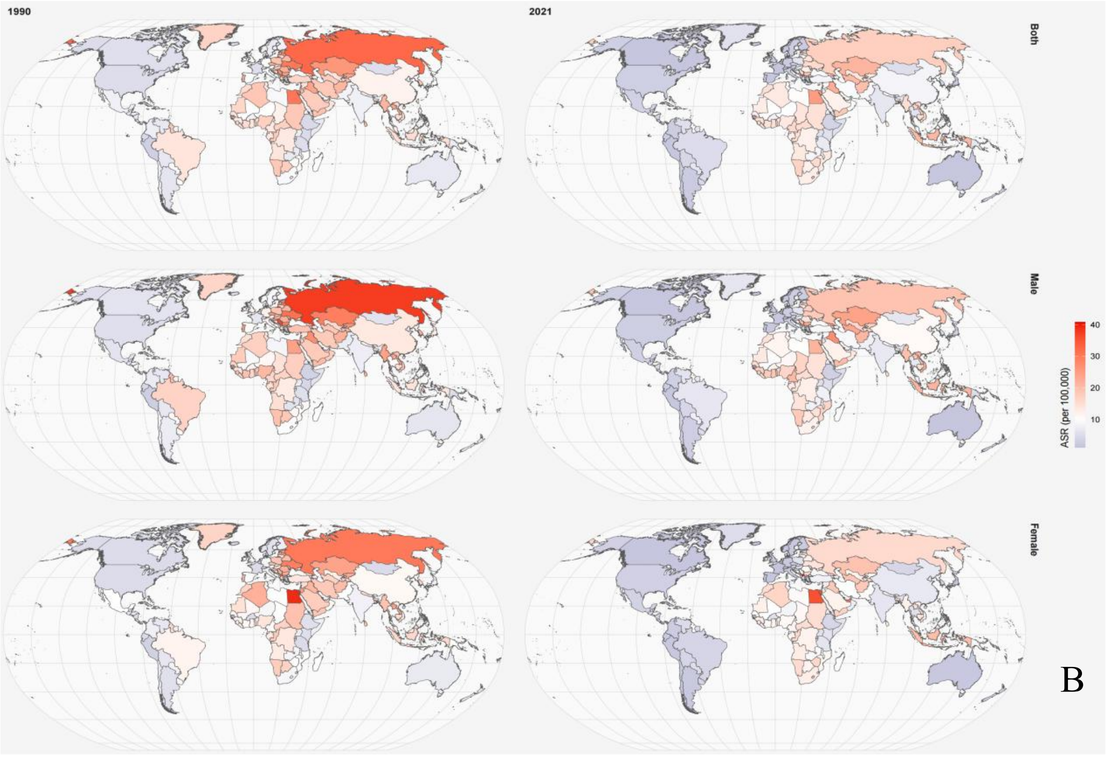
1990
2021
Both
Male
Female
40
30
20
10
ASR (per 100,000)
B

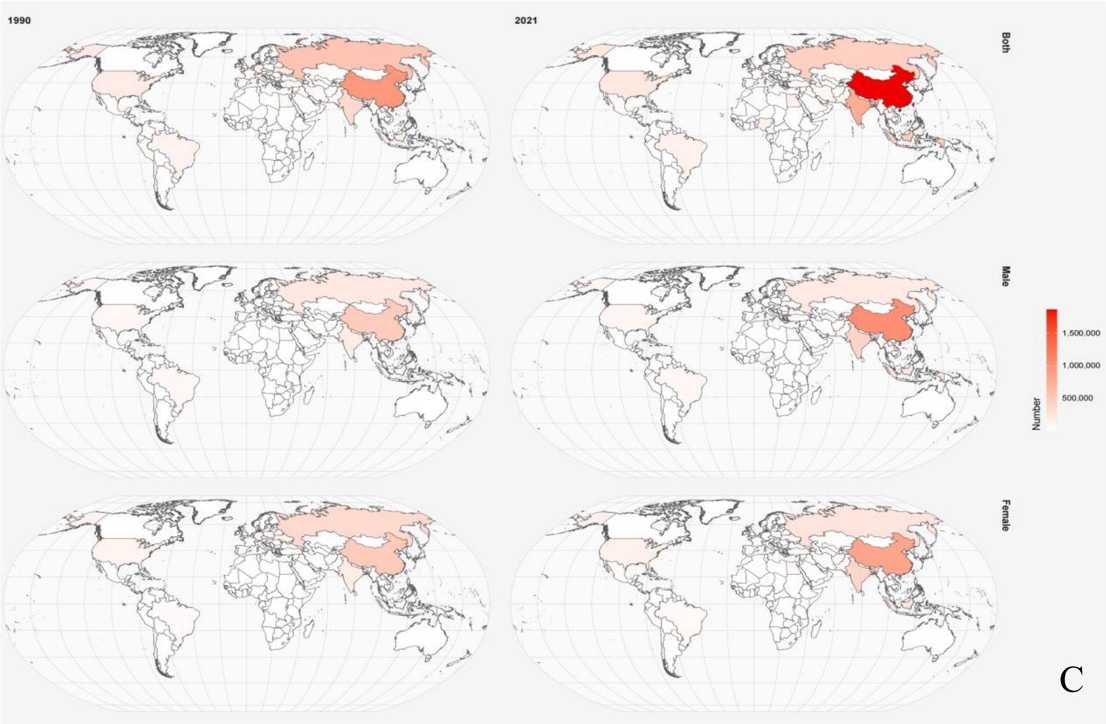

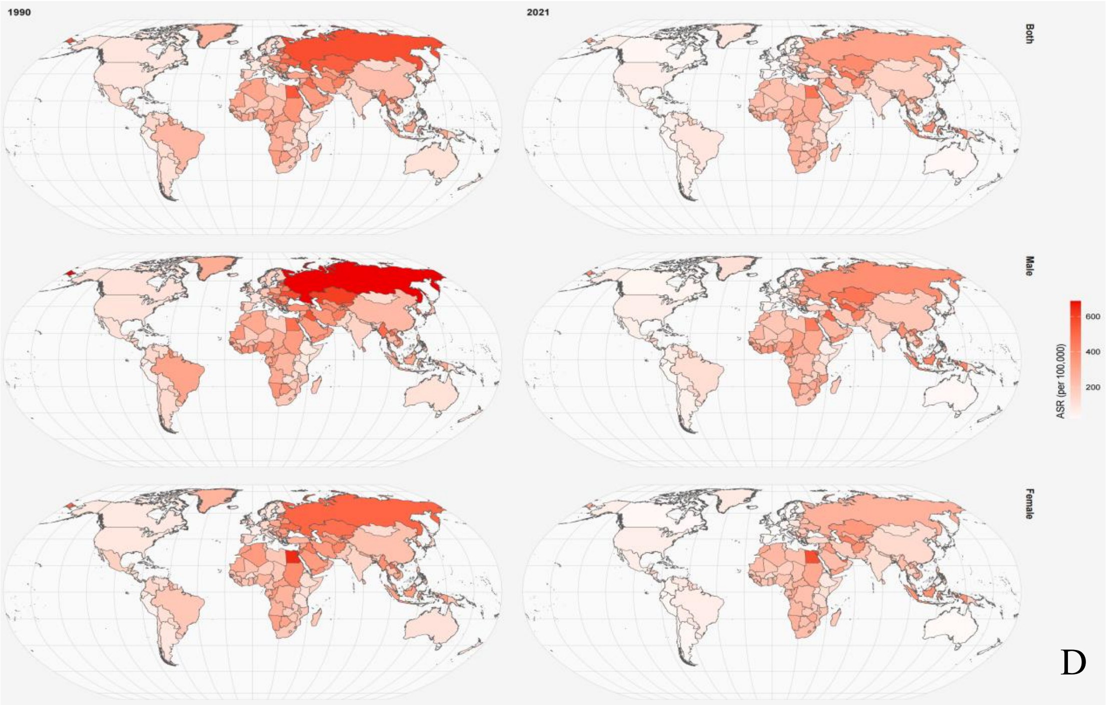

Fig. S33A Comparison of the global disease burden of kidney dysfunction related ischemic stroke mortality in males and females across 204 countries and territories between 1990 and 2021; B Comparison of the global disease burden of kidney dysfunction related ischemic stroke mortality rate in males and females across 204 countries and territories between 1990 and 2021; C Comparison of the global disease burden of kidney dysfunction related ischemic stroke disability-adjusted life years in males and females across 204 countries and territories between 1990 and 2021; D Comparison of the global disease burden of kidney dysfunction related ischemic stroke disability-adjusted life years rate in males and females across 204 countries and territories between 1990 and 2021.

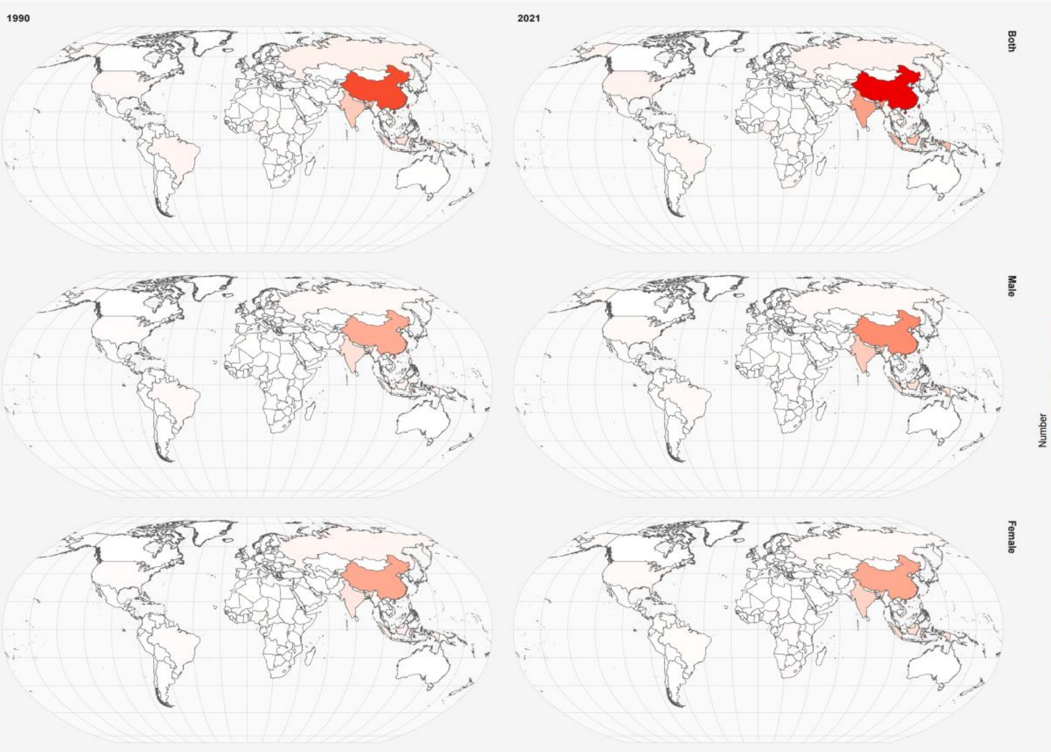
1990
2021
Both
Male
Female
100,000
75,000
50,000
25,000
Number

A

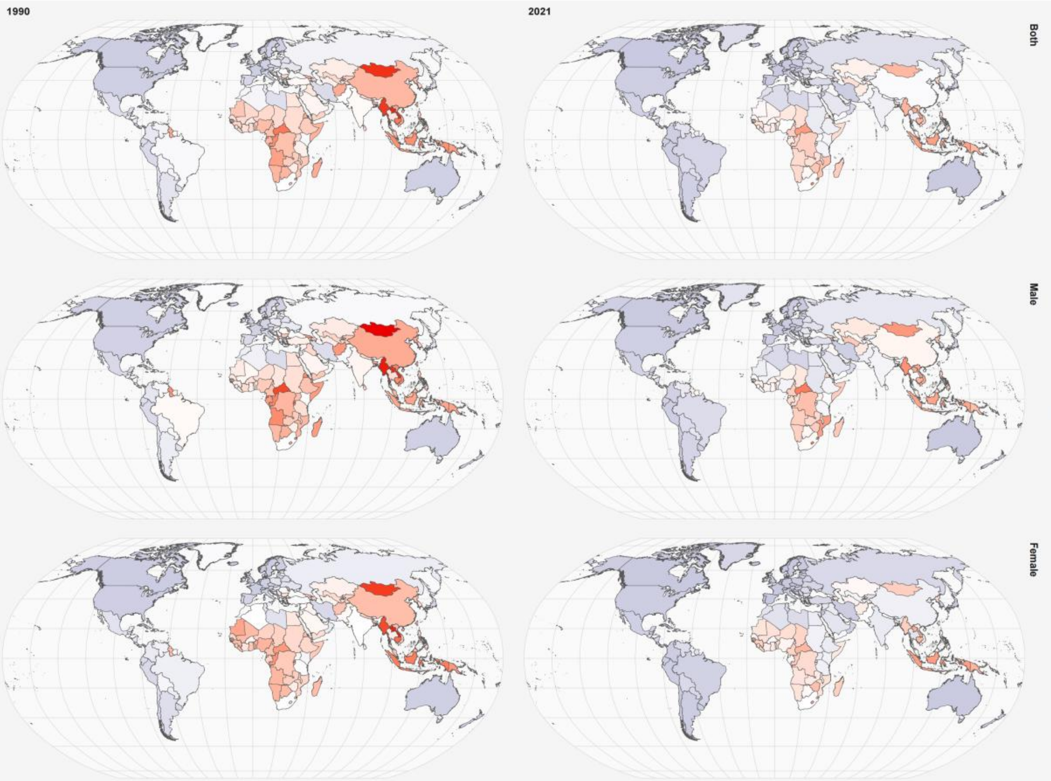
1990
2021
Both
Male
Female
40
30
20
10
ASR (per 100,000)

B

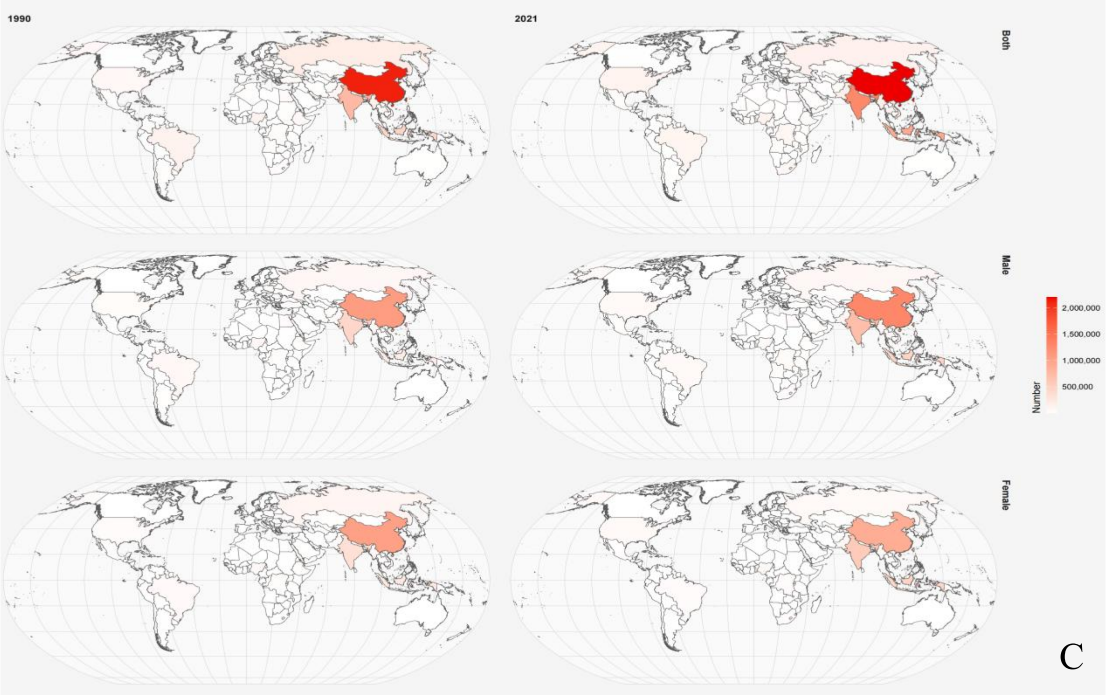

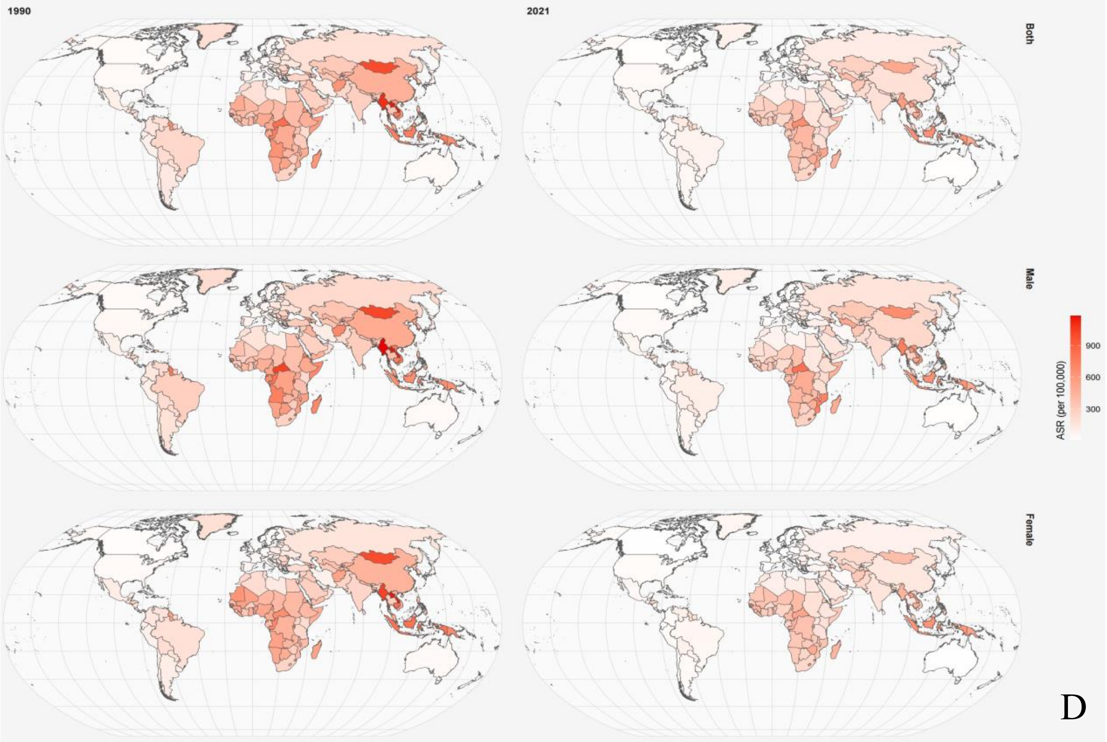

Fig. S34A Comparison of the global disease burden of kidney dysfunction related intracerebral hemorrhage mortality in males and females across 204 countries and territories between 1990 and 2021; B Comparison of the global disease burden of kidney dysfunction related intracerebral hemorrhage mortality rate in males and females across 204 countries and territories between 1990 and 2021; C Comparison of the global disease burden of kidney dysfunction related intracerebral hemorrhage disability-adjusted life years in males and females across 204 countries and territories between 1990 and 2021; D Comparison of the global disease burden of kidney dysfunction related intracerebral hemorrhage disability-adjusted life years rate in males and females across 204 countries and territories between 1990 and 2021.

A

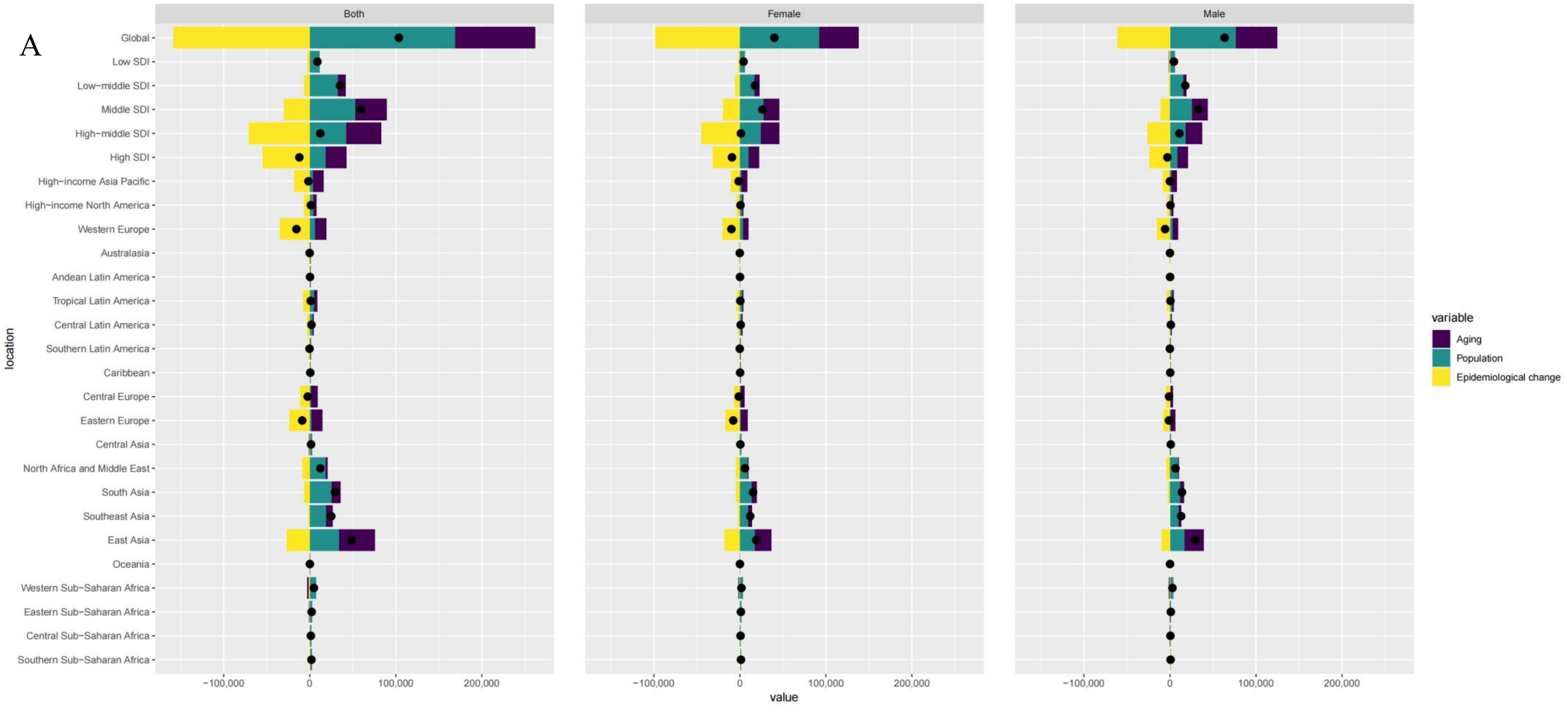

Both
Female
Male
Global
Low SDI
Low−middle SDI
Middle SDI
High−middle SDI
High SDI
High−income Asia Pacific
High−income North America
Western Europe
Australasia
Andean Latin America
Tropical Latin America
Central Latin America
Southern Latin America
Caribbean
Central Europe
Eastern Europe
Central Asia
North Africa and Middle East
South Asia
Southeast Asia
East Asia
Oceania
Western Sub−Saharan Africa
Eastern Sub−Saharan Africa
Central Sub−Saharan Africa
Southern Sub−Saharan Africa
location
−100,000
0
100,000
200,000
value
variable
Aging
Population
Epidemiological change

B

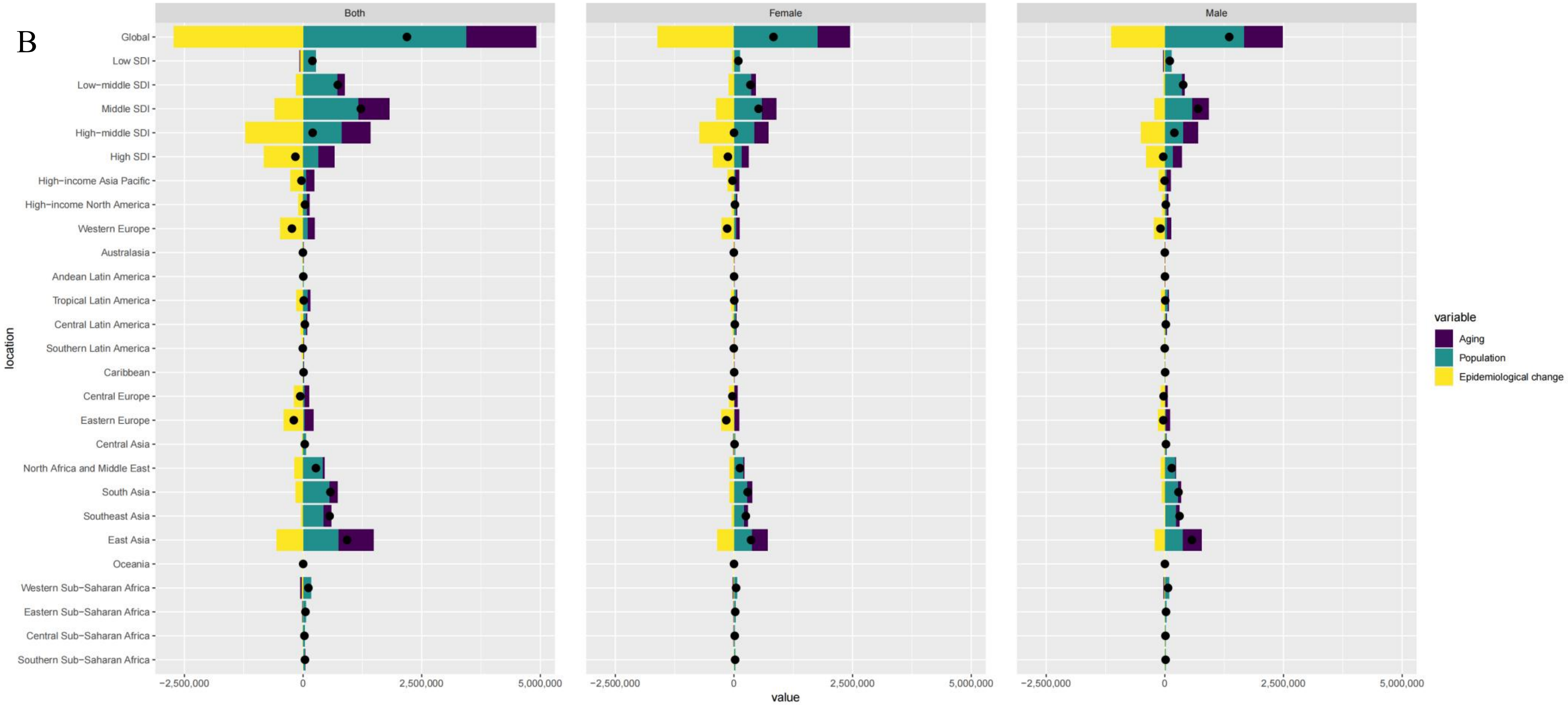

Fig. S35A Decomposition analysis of kidney dysfunction related ischemic stroke change in mortality by SDI and 21 GBD region, 1990 to 2021; B Decomposition analysis of kidney dysfunction related ischemic stroke change in disability-adjusted life years by SDI and 21 GBD region, 1990 to 2021

Notes: SDI: socio-demographic index; GBD: Global Burden of Disease; Black dots represent the total change contributed by all three components. A positive value for each component indicates a corresponding positive contribution, and a negative value indicates a corresponding negative contribution.

A

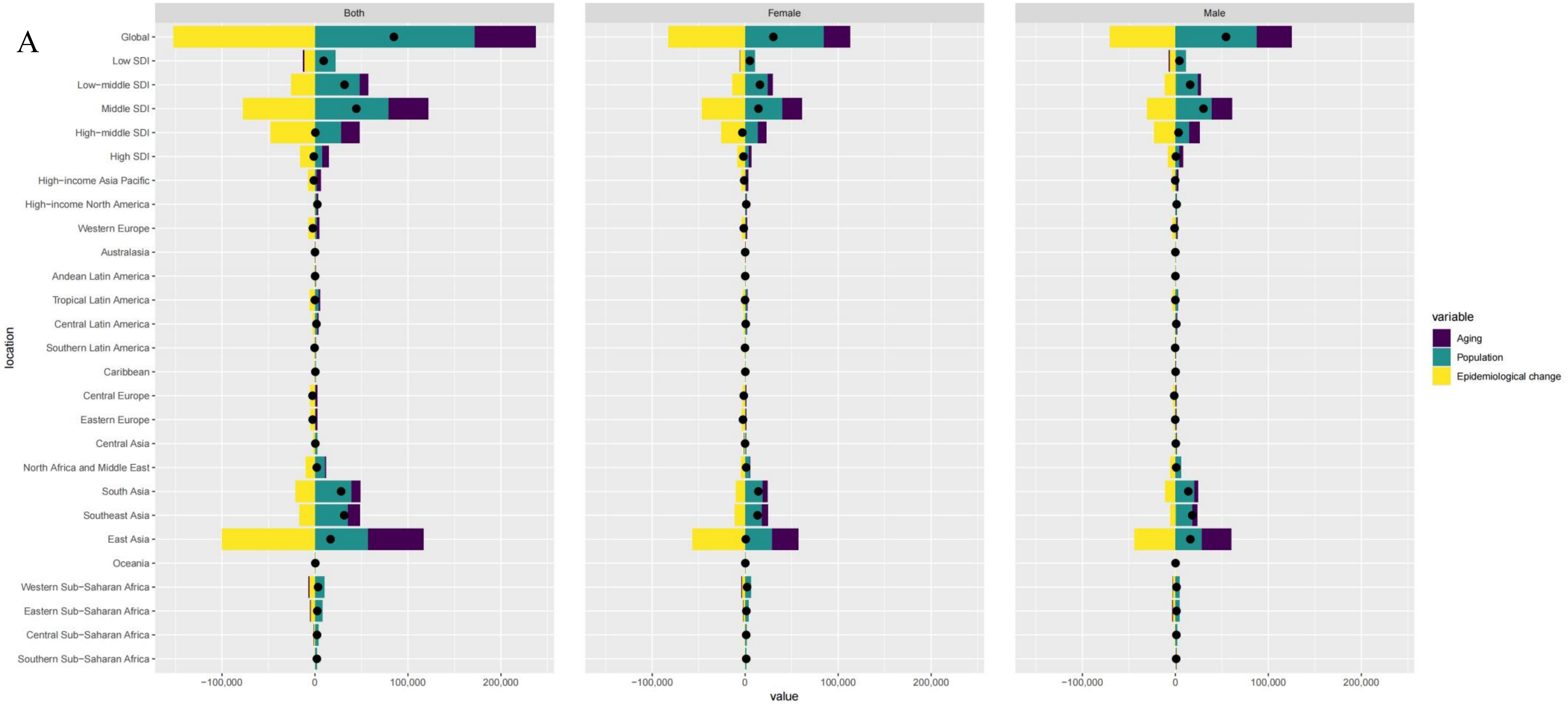

Both
Female
Male
Global
Low SDI
Low−middle SDI
Middle SDI
High−middle SDI
High SDI
High−income Asia Pacific
High−income North America
Western Europe
Australasia
Andean Latin America
Tropical Latin America
Central Latin America
Southern Latin America
Caribbean
Central Europe
Eastern Europe
Central Asia
North Africa and Middle East
South Asia
Southeast Asia
East Asia
Oceania
Western Sub−Saharan Africa
Eastern Sub−Saharan Africa
Central Sub−Saharan Africa
Southern Sub−Saharan Africa
location
−100,000
0
100,000
200,000
value
variable
Aging
Population
Epidemiological change

B

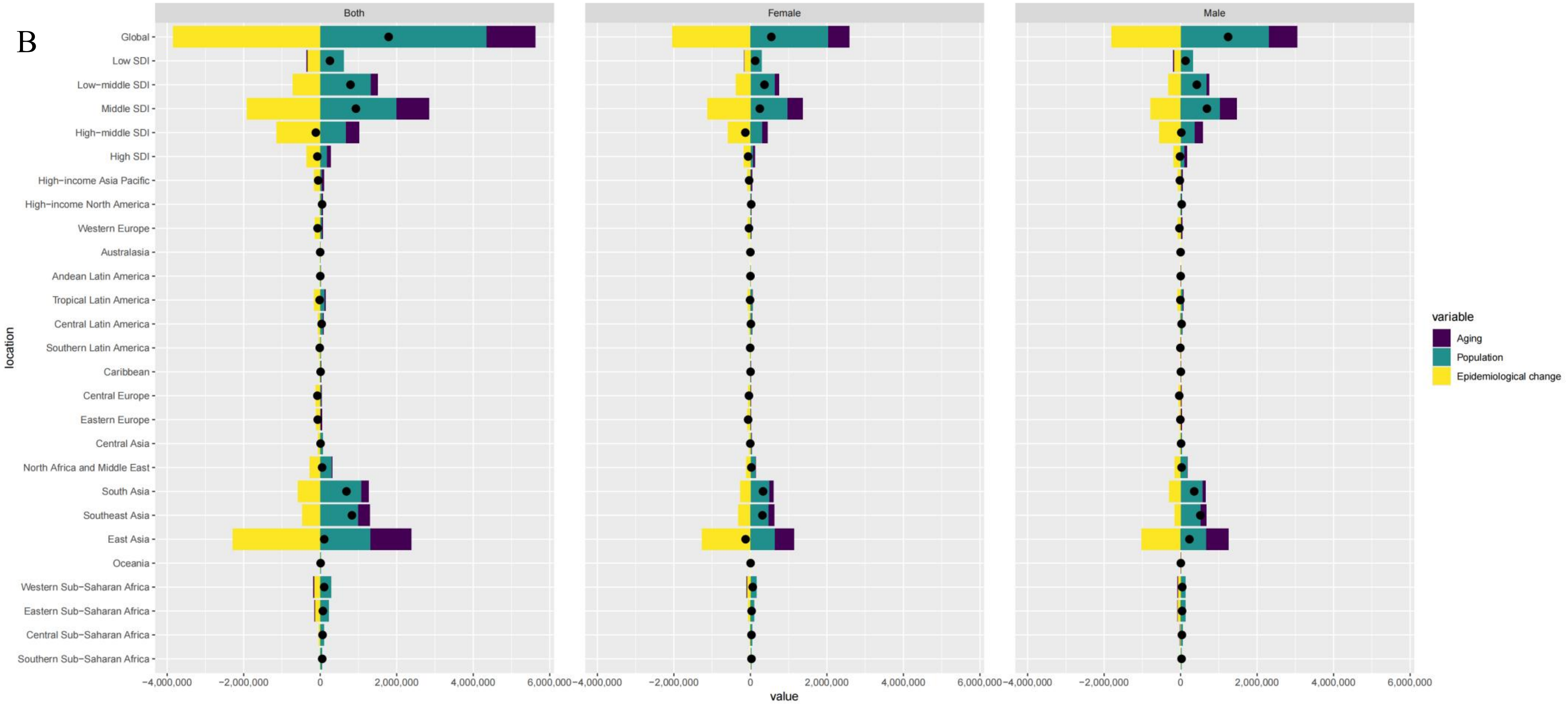

Fig. S36A Decomposition analysis of kidney dysfunction related intracerebral hemorrhage change in mortality by SDI and 21 GBD region, 1990 to 2021; B Decomposition analysis of kidney dysfunction related intracerebral hemorrhage change in disability-adjusted life years by SDI and 21 GBD region, 1990 to 2021.

Notes: SDI: socio-demographic index; GBD: Global Burden of Disease; Black dots represent the total change contributed by all three components. A positive value for each component indicates a corresponding positive contribution, and a negative value indicates a corresponding negative contribution.

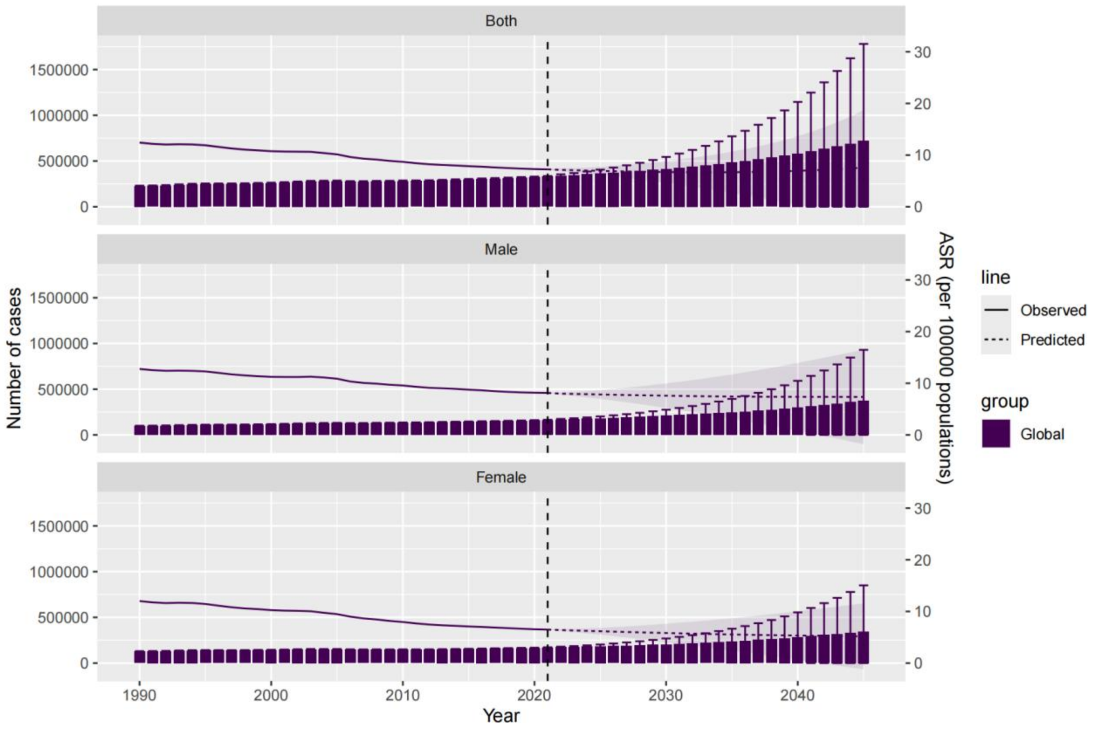

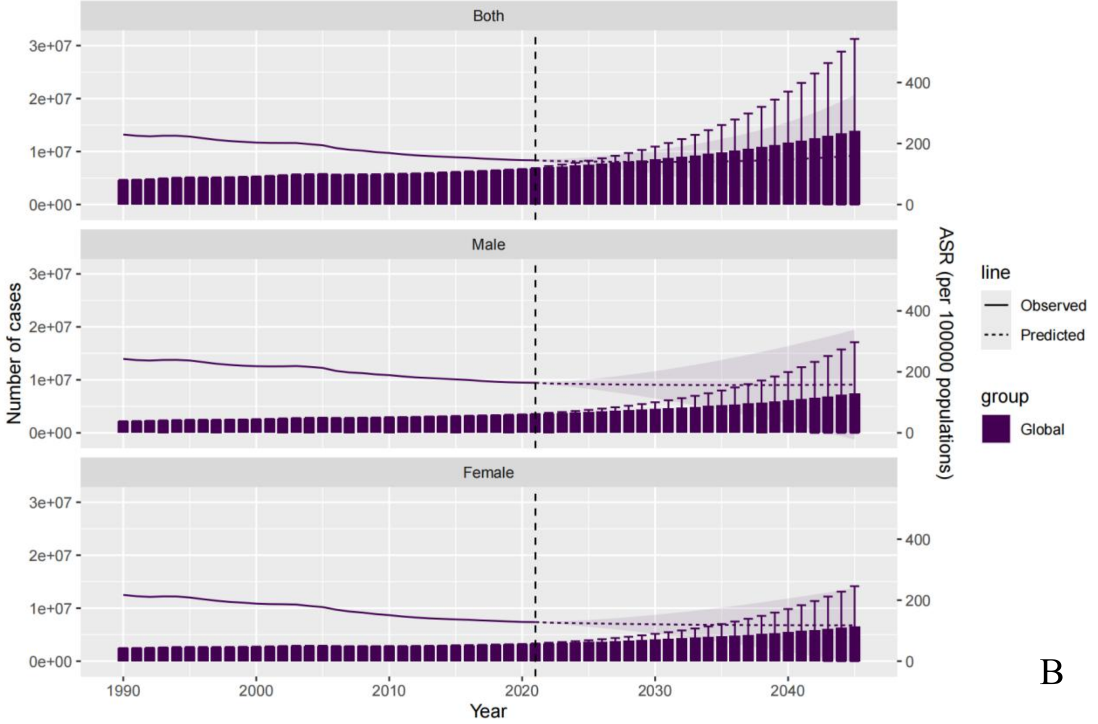

B

Fig. S37A Future forecasts of GBD in kidney dysfunction related ischemic stroke mortality using bayesian age-period-cohort model; B Future forecasts of GBD in kidney dysfunction related ischemic stroke disability-adjusted life years using bayesian age-period-cohort model.

Notes: The line graphs show the change of age-standardized rates (ASR), and the bar graphs show the change of burden.

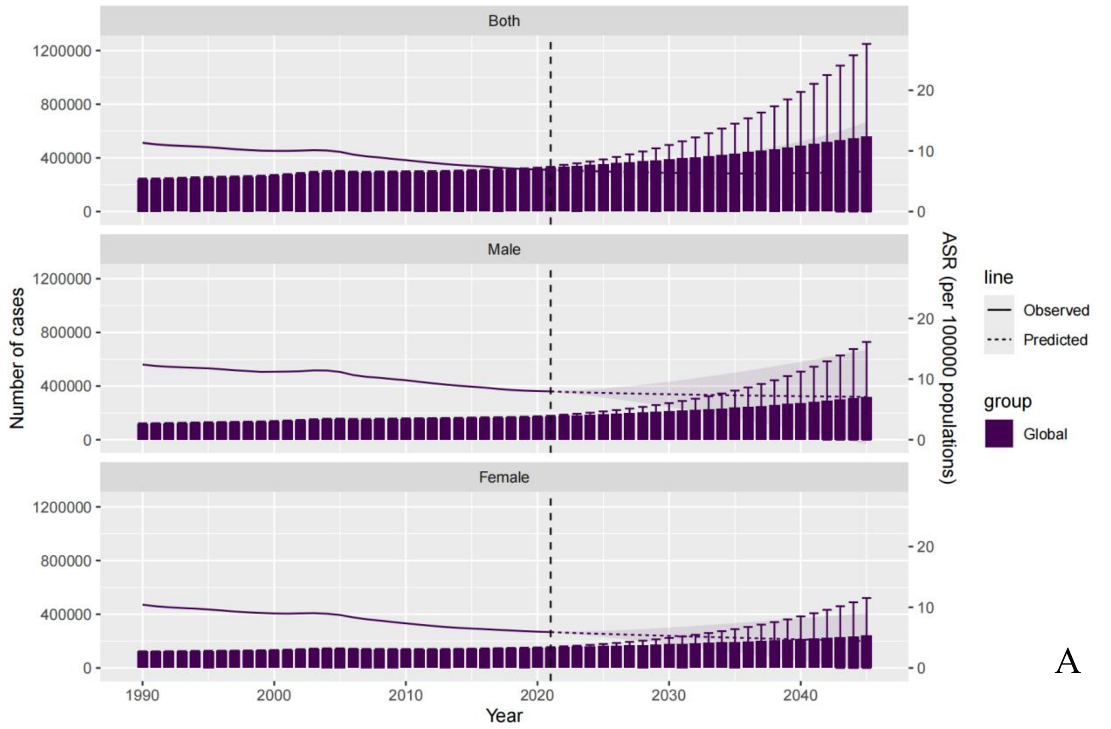

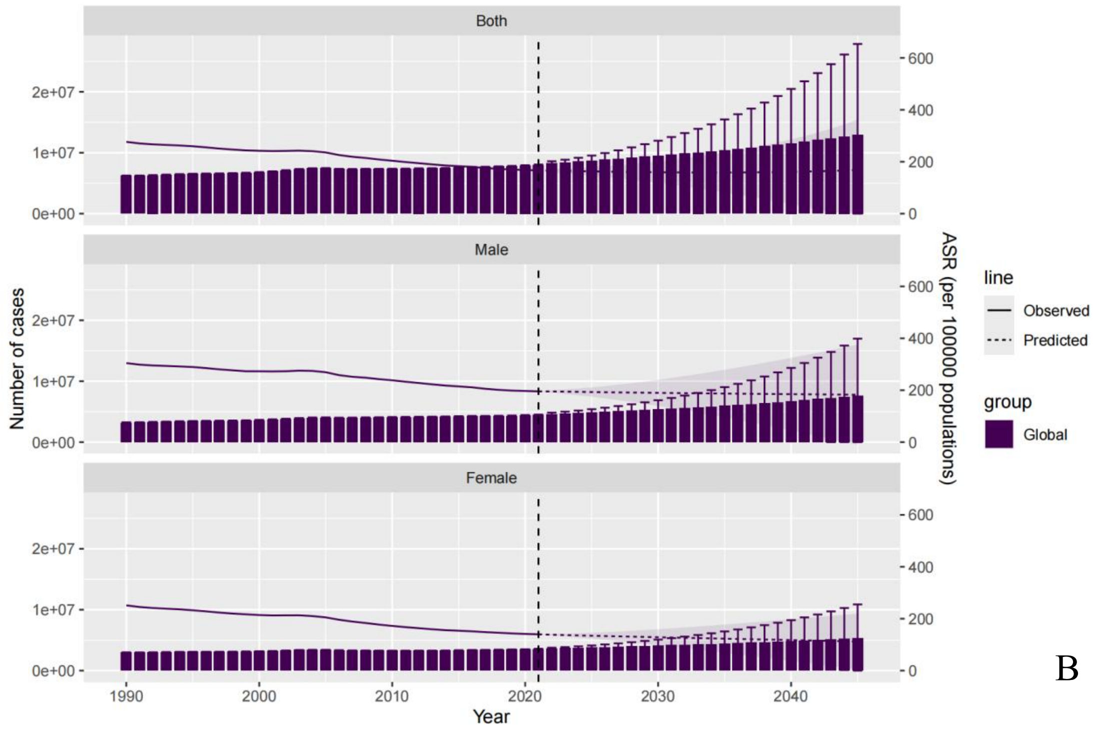

Fig. S38A Future forecasts of GBD in kidney dysfunction related intracerebral hemorrhage mortality using bayesian age-period-cohort model; B Future forecasts of GBD in kidney dysfunction related intracerebral hemorrhage disability-adjusted life years using bayesian age-period-cohort model.

Notes: The line graphs show the change of age-standardized rates (ASR), and the bar graphs show the change of burden.

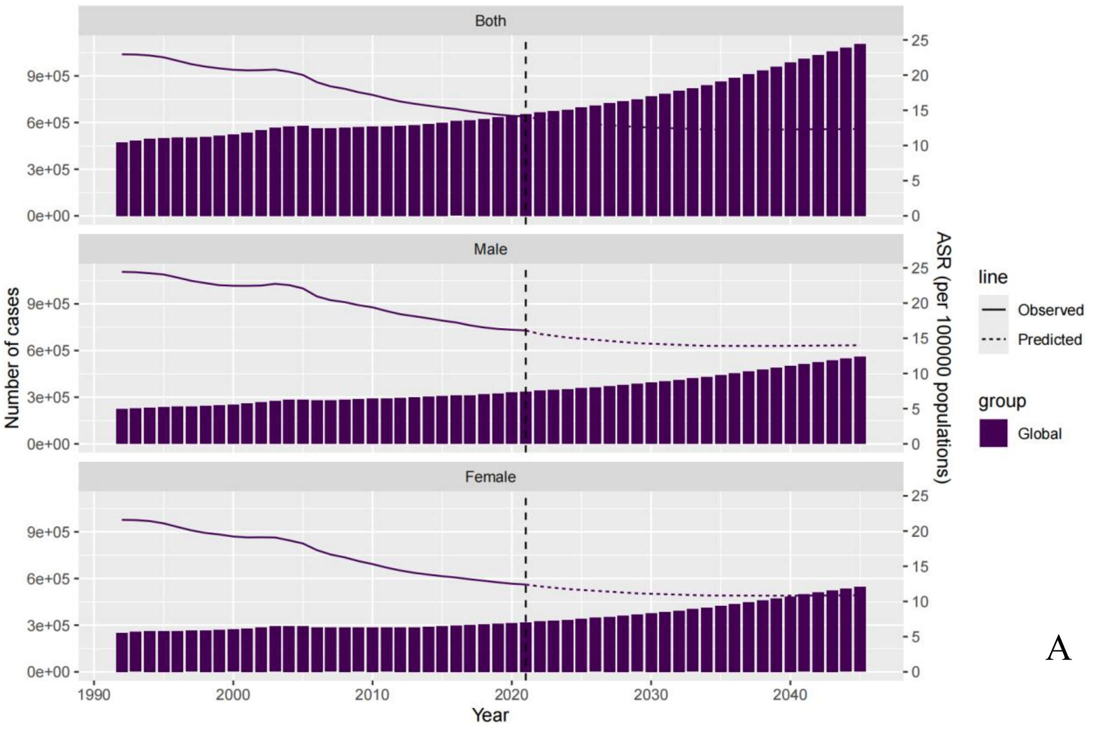

A

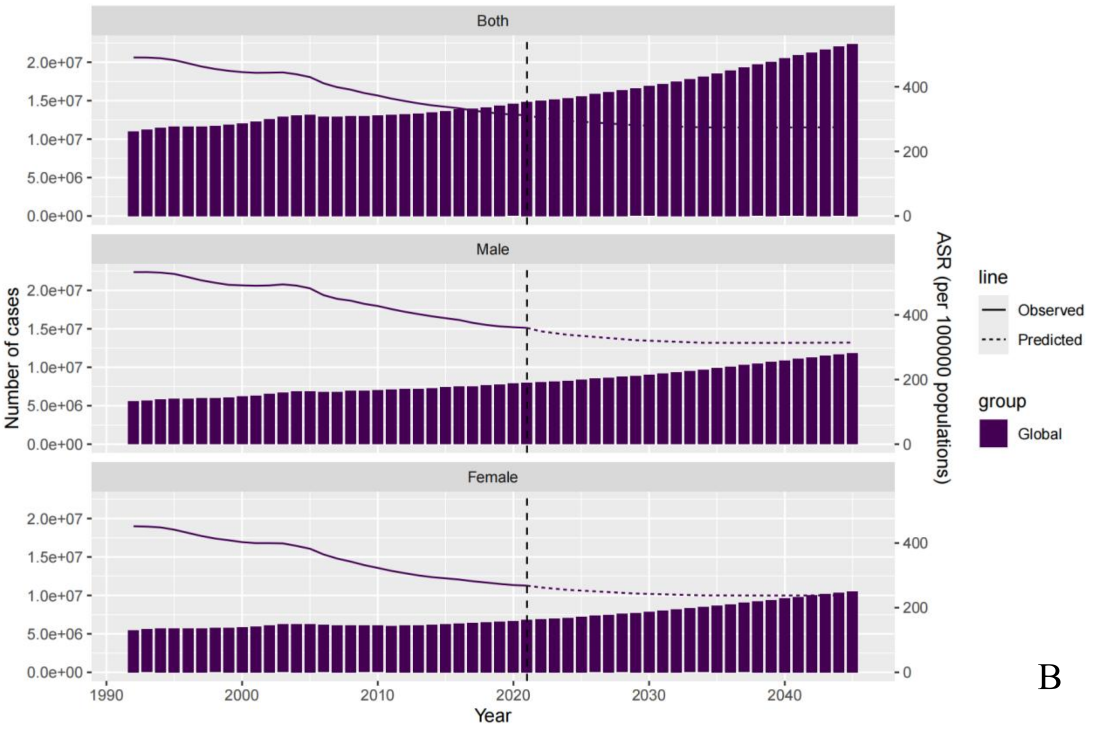

B

Fig. S39A Future forecasts of GBD in kidney dysfunction related stroke mortality using nordpred model; B Future forecasts of GBD in kidney dysfunction related stroke disability-adjusted life years using nordpred model.

Notes: The line graphs show the change of age-standardized rates (ASR), and the bar graphs show the change of burden.

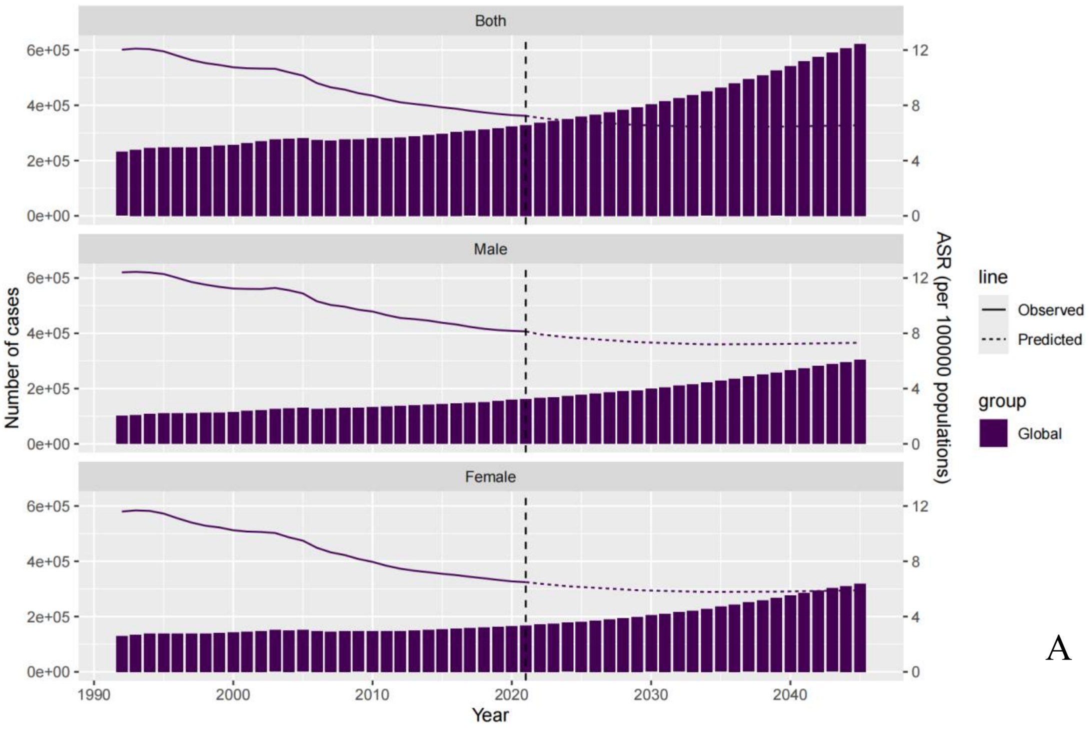

A

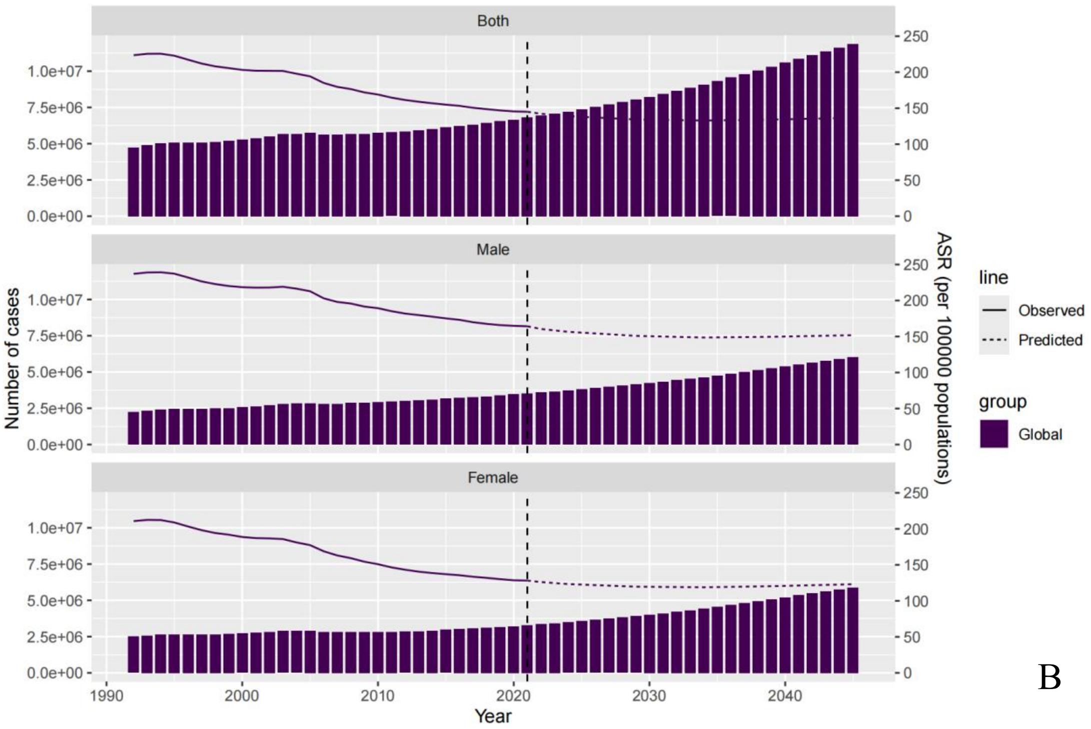

B

Fig. S40A Future forecasts of GBD in kidney dysfunction related ischemic stroke mortality using nordpred model; B Future forecasts of GBD in kidney dysfunction related ischemic stroke disability-adjusted life years using nordpred model.

Notes: The line graphs show the change of age-standardized rates (ASR), and the bar graphs show the change of burden.

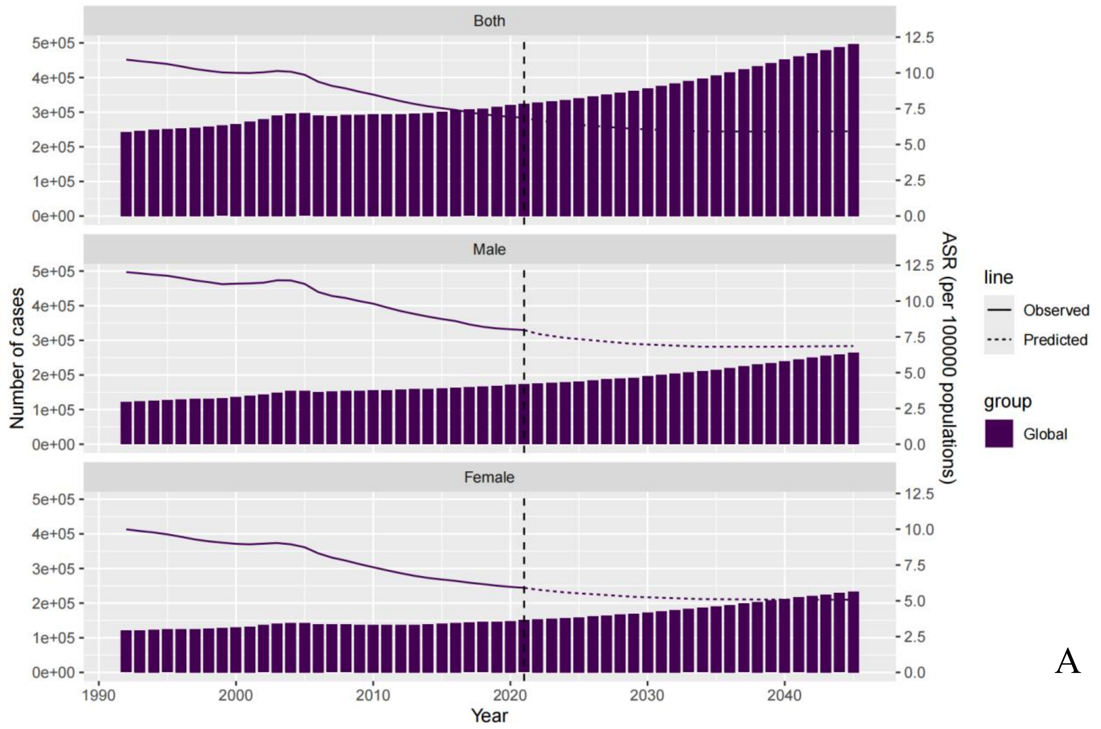

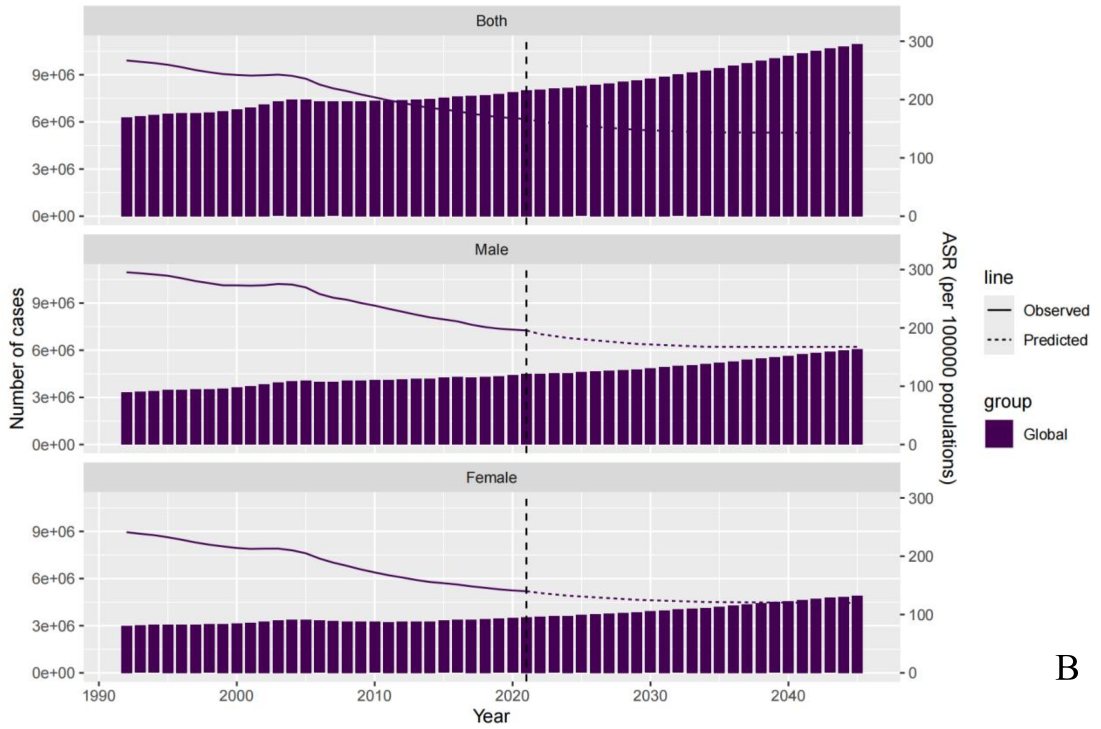

Fig. S41A Future forecasts of GBD in kidney dysfunction related intracerebral hemorrhage mortality using nordpred model; B Future forecasts of GBD in kidney dysfunction related intracerebral hemorrhage disability-adjusted life years using nordpred model.

Notes: The line graphs show the change of age-standardized rates (ASR), and the bar graphs show the change of burden.
