# Supplementary material for: Global burden and trends of stroke attributable to kidney dysfunction from 1990 to 2021
Source: Clin Kidney J. 2025 May 23;18(9):sfaf160. doi: 10.1093/ckj/sfaf160 (PMC12399972; doi:10.1093/ckj/sfaf160)
Supplement: sfaf160_Supplemental_Files [file sfaf160_supplemental_files.zip › Tables S1-12 R3.docx]

**Table S1. Global, regional, and national/territorial trends in ischemic stroke attributable kidney dysfunction burden: mortality, and DALYs (1990–2021).**

| **Location** | **1990** | | **2021** | | **EAPC 95%CI** |
| --- | --- | --- | --- | --- | --- |
|  | **Number** | **ASR, per**  **100,000 persons** | **Number** | **ASR, per**  **100,000 persons** |  |
| **Deaths** |  |  |  |  |  |
| Global | 225704.3 (143330.6, 317278.7) | 12.4 (7.6, 17.8) | 329237.4 (205507.5, 467377.9) | 7.2 (4.5, 10.3) | -1.9 (-2.0, -1.8) |
| High SDI | 52844.4 (30548.8, 77349.4) | 8.5 (4.9, 12.5) | 40691.8 (22390.6, 60800.7) | 2.9 (1.6, 4.3) | -3.8 (-4.0, -3.6) |
| High-middle SDI | 83836.9 (52888.8, 117836.1) | 18.4 (11.2, 26.2) | 96040.2 (58397.2, 138129.5) | 8.9 (5.4, 12.8) | -2.7 (-2.9, -2.5) |
| Middle SDI | 51340.4 (34056.8, 71420.0) | 12.1 (7.6, 17.2) | 110754.8 (70725.3, 157781.3) | 8.5 (5.3, 12.3) | -1.0 (-1.1, -0.9) |
| Low-middle SDI | 28229.3 (18605.1, 39717.6) | 11.2 (7.0, 16.0) | 63444.1 (40699.3, 89085.8) | 9.8 (6.1, 13.9) | -0.5 (-0.5, -0.4) |
| Low SDI | 9125.8 (5875.4, 13545.7) | 10.2 (6.3, 15.4) | 17970.6 (11307.2, 26479.1) | 8.8 (5.3, 13.0) | -0.5 (-0.6, -0.5) |
| High-income Asia Pacific | 10657.0 (5917.5, 15897.0) | 11.0 (5.9, 16.6) | 9060.5 (4493.6, 14215.5) | 2.5 (1.3, 3.9) | -5.1 (-5.2, -4.9) |
| High-income North America | 10529.6 (5907.0, 15520.0) | 5.1 (2.9, 7.5) | 11810.1 (6544.3, 17690.5) | 2.9 (1.6, 4.3) | -2.4 (-2.7, -2.1) |
| Western Europe | 31333.7 (17540.0, 46453.7) | 9.2 (5.1, 13.6) | 15865.2 (8454.3, 24094.5) | 2.3 (1.2, 3.4) | -4.7 (-4.9, -4.5) |
| Australasia | 838.7 (458.0, 1255.8) | 6.8 (3.6, 10.3) | 678.7 (348.2, 1040.5) | 1.9 (1.0, 2.9) | -4.4 (-4.5, -4.3) |
| Andean Latin America | 386.7 (232.8, 562.1) | 4.0 (2.4, 5.9) | 687.0 (396.0, 1037.3) | 2.3 (1.3, 3.4) | -2.1 (-2.3, -1.8) |
| Tropical Latin America | 5538.2 (3664.9, 7552.2) | 14.0 (8.9, 19.4) | 6647.8 (4229.9, 9253.2) | 5.0 (3.1, 7.0) | -3.1 (-3.3, -3.0) |
| Central Latin America | 2682.7 (1714.1, 3723.4) | 7.3 (4.6, 10.3) | 4643.5 (2903.4, 6505.1) | 3.7 (2.3, 5.2) | -2.4 (-2.6, -2.2) |
| Southern Latin America | 1618.1 (938.4, 2390.6) | 7.0 (4.0, 10.4) | 1336.8 (763.6, 1999.6) | 2.6 (1.5, 3.9) | -2.8 (-3.0, -2.7) |
| Caribbean | 972.3 (611.4, 1377.8) | 7.5 (4.6, 10.7) | 1566.8 (955.5, 2283.3) | 5.3 (3.2, 7.7) | -1.1 (-1.1, -1.0) |
| Central Europe | 14507.4 (8808.4, 20668.1) | 20.0 (11.8, 28.8) | 12085.1 (6972.2, 17658.7) | 8.9 (5.2, 13.0) | -3.0 (-3.1, -2.8) |
| Eastern Europe | 42019.8 (26588.6, 58820.1) | 30.6 (18.9, 43.2) | 33158.0 (20232.6, 47226.8) | 16.5 (10.1, 23.4) | -2.8 (-3.3, -2.3) |
| Central Asia | 4039.1 (2670.9, 5512.7) | 17.7 (11.5, 24.4) | 5532.6 (3613.6, 7638.4) | 15.4 (9.8, 21.6) | -0.9 (-1.2, -0.6) |
| North Africa and Middle East | 13569.3 (8764.8, 19371.2) | 19.0 (11.7, 27.6) | 25898.8 (15951.8, 36969.8) | 13.0 (7.7, 18.8) | -1.2 (-1.2, -1.1) |
| South Asia | 19780.1 (12579.3, 29264.5) | 8.7 (5.3, 12.9) | 49055.3 (30473.9, 73307.9) | 7.4 (4.5, 11.1) | -0.7 (-0.8, -0.6) |
| Southeast Asia | 15584.9 (10322.3, 21934.2) | 14.8 (9.5, 21.1) | 40607.7 (25756.9, 57866.1) | 14.0 (8.7, 20.0) | -0.1 (-0.2, 0.1) |
| East Asia | 41713.1 (26798.5, 59834.7) | 11.8 (7.1, 17.3) | 90406.6 (54362.4, 134114.1) | 8.3 (4.9, 12.4) | -0.8 (-1.1, -0.5) |
| Oceania | 79.0 (47.0, 120.0) | 8.0 (4.5, 12.3) | 182.1 (109.9, 277.7) | 6.9 (4.0, 10.6) | -0.6 (-0.6, -0.5) |
| Western Sub-Saharan Africa | 5606.4 (3516.2, 8386.9) | 15.1 (9.1, 22.9) | 10359.4 (6602.2, 14830.1) | 13.3 (8.2, 19.2) | -0.4 (-0.5, -0.3) |
| Eastern Sub-Saharan Africa | 1986.0 (1212.5, 3008.9) | 7.0 (4.1, 10.7) | 4168.9 (2490.1, 6340.4) | 6.4 (3.7, 9.9) | -0.4 (-0.4, -0.3) |
| Central Sub-Saharan Africa | 1087.2 (664.6, 1620.3) | 13.8 (8.0, 21.0) | 2354.9 (1351.7, 3708.3) | 12.6 (6.9, 20.3) | -0.5 (-0.6, -0.4) |
| Southern Sub-Saharan Africa | 1175.0 (738.7, 1684.3) | 9.9 (6.1, 14.4) | 3131.9 (2067.4, 4353.2) | 13.0 (8.3, 18.4) | 1.0 (0.5, 1.5) |
| Afghanistan | 672.4 (368.8, 1099.7) | 21.2 (11.3, 35.0) | 777.3 (431.8, 1277.3) | 19.6 (10.7, 32.2) | -0.4 (-0.5, -0.2) |
| Albania | 65.2 (37.1, 99.4) | 7.5 (4.1, 11.6) | 133.0 (65.4, 214.2) | 5.8 (2.8, 9.4) | -0.4 (-0.7, -0.2) |
| Algeria | 776.9 (440.8, 1228.4) | 18.6 (10.0, 30.0) | 1822.8 (983.0, 2877.6) | 13.3 (6.9, 20.9) | -0.9 (-0.9, -0.8) |
| American Samoa | 0.7 (0.4, 1.0) | 8.4 (4.9, 12.5) | 1.3 (0.8, 2.1) | 6.6 (3.8, 10.2) | -1.0 (-1.2, -0.9) |
| Andorra | 1.1 (0.6, 1.8) | 4.2 (2.2, 7.1) | 1.9 (0.9, 3.3) | 2.0 (1.0, 3.4) | -2.2 (-2.5, -2.0) |
| Angola | 185.4 (110.6, 283.7) | 14.0 (8.0, 21.8) | 525.1 (307.3, 801.5) | 13.3 (7.6, 20.7) | -0.4 (-0.5, -0.3) |
| Antigua and Barbuda | 2.7 (1.6, 3.9) | 8.2 (5.0, 11.8) | 2.6 (1.6, 3.8) | 5.3 (3.2, 7.7) | -1.6 (-1.8, -1.4) |
| Argentina | 1028.6 (590.7, 1541.5) | 6.4 (3.6, 9.6) | 759.1 (436.5, 1140.1) | 2.3 (1.3, 3.5) | -2.8 (-3.0, -2.6) |
| Armenia | 195.3 (125.4, 270.5) | 15.9 (10.0, 22.2) | 257.3 (156.6, 364.4) | 10.7 (6.6, 15.1) | -2.3 (-2.7, -1.9) |
| Australia | 692.8 (376.1, 1038.9) | 6.8 (3.6, 10.3) | 537.2 (273.5, 824.7) | 1.7 (0.9, 2.7) | -4.6 (-4.8, -4.5) |
| Austria | 712.2 (393.1, 1059.6) | 9.9 (5.5, 14.7) | 245.7 (131.5, 369.6) | 1.9 (1.1, 2.9) | -5.7 (-6.0, -5.3) |
| Azerbaijan | 223.7 (138.4, 323.3) | 9.7 (5.9, 14.0) | 357.5 (206.4, 549.3) | 8.6 (4.9, 13.3) | -0.1 (-0.4, 0.2) |
| Bahamas | 4.2 (2.6, 6.0) | 5.8 (3.5, 8.4) | 7.4 (4.4, 11.0) | 4.1 (2.4, 6.1) | -1.2 (-1.3, -1.0) |
| Bahrain | 8.9 (5.6, 13.0) | 16.9 (9.8, 25.6) | 22.4 (13.2, 33.7) | 9.6 (5.2, 14.8) | -2.3 (-2.8, -1.8) |
| Bangladesh | 2859.2 (1625.4, 4483.6) | 14.0 (7.8, 21.9) | 7894.3 (4250.8, 12480.9) | 12.9 (6.8, 20.3) | -0.4 (-0.8, -0.1) |
| Barbados | 17.5 (10.4, 25.8) | 9.9 (5.9, 14.4) | 17.9 (10.2, 26.6) | 6.1 (3.5, 9.1) | -1.8 (-2.0, -1.6) |
| Belarus | 1421.2 (900.6, 2002.3) | 20.6 (13.0, 29.1) | 1315.7 (772.3, 1929.2) | 14.3 (8.4, 20.9) | -1.9 (-2.3, -1.5) |
| Belgium | 787.5 (433.4, 1175.0) | 8.7 (4.8, 12.9) | 367.2 (196.6, 559.2) | 2.1 (1.2, 3.2) | -4.2 (-4.4, -4.1) |
| Belize | 2.1 (1.3, 3.0) | 4.2 (2.5, 6.1) | 5.1 (3.1, 7.4) | 3.8 (2.2, 5.5) | -0.8 (-1.3, -0.2) |
| Benin | 143.9 (87.6, 217.3) | 15.4 (9.2, 23.4) | 291.8 (172.0, 448.2) | 13.8 (8.0, 21.3) | -0.3 (-0.4, -0.2) |
| Bermuda | 2.1 (1.3, 3.1) | 6.8 (4.1, 10.0) | 2.3 (1.3, 3.4) | 2.6 (1.5, 4.0) | -3.2 (-3.4, -3.0) |
| Bhutan | 7.8 (4.3, 12.7) | 8.8 (4.6, 14.7) | 21.7 (11.8, 34.6) | 7.3 (4.0, 11.8) | -0.6 (-0.7, -0.5) |
| Bolivia (Plurinational State of) | 90.7 (48.1, 147.1) | 6.6 (3.4, 10.7) | 164.2 (82.0, 271.0) | 4.1 (2.0, 6.7) | -1.5 (-1.6, -1.4) |
| Bosnia and Herzegovina | 303.4 (185.9, 443.7) | 17.5 (10.4, 25.8) | 457.3 (253.1, 686.9) | 12.8 (7.1, 19.2) | -1.3 (-1.5, -1.2) |
| Botswana | 35.9 (21.4, 53.4) | 17.5 (9.9, 26.6) | 67.5 (39.9, 103.0) | 12.2 (7.0, 18.9) | -1.1 (-1.3, -0.9) |
| Brazil | 5438.5 (3601.4, 7413.3) | 14.1 (8.9, 19.6) | 6467.2 (4114.8, 9010.8) | 4.9 (3.1, 6.9) | -3.2 (-3.3, -3.1) |
| Brunei Darussalam | 6.1 (3.6, 9.3) | 14.7 (8.4, 22.4) | 7.8 (4.4, 11.7) | 6.7 (3.5, 10.2) | -2.3 (-2.5, -2.0) |
| Bulgaria | 1228.7 (770.5, 1716.5) | 25.6 (14.8, 36.8) | 1687.5 (978.8, 2457.4) | 19.7 (11.4, 28.7) | -0.7 (-0.9, -0.5) |
| Burkina Faso | 141.4 (82.2, 228.3) | 8.1 (4.6, 13.1) | 314.4 (179.6, 499.4) | 8.3 (4.7, 13.2) | 0.3 (0.2, 0.4) |
| Burundi | 120.9 (63.6, 197.4) | 11.8 (6.1, 19.5) | 128.4 (68.5, 213.9) | 7.3 (3.8, 12.2) | -2.1 (-2.4, -1.9) |
| Cabo Verde | 11.7 (6.6, 17.8) | 8.8 (5.0, 13.3) | 26.2 (14.9, 40.6) | 11.8 (6.8, 18.3) | 0.7 (0.3, 1.0) |
| Cambodia | 264.1 (159.8, 399.1) | 15.2 (8.9, 23.2) | 687.4 (407.1, 1032.2) | 14.5 (8.4, 21.8) | -0.2 (-0.4, -0.1) |
| Cameroon | 233.0 (135.5, 363.8) | 13.2 (7.5, 20.5) | 785.9 (455.9, 1241.2) | 15.9 (9.1, 25.0) | 0.7 (0.2, 1.2) |
| Canada | 933.6 (524.3, 1376.9) | 5.3 (3.0, 7.9) | 778.7 (412.0, 1195.3) | 1.6 (0.9, 2.5) | -4.1 (-4.3, -3.9) |
| Central African Republic | 65.4 (37.4, 102.5) | 16.9 (9.3, 27.0) | 102.3 (56.3, 167.0) | 15.3 (8.0, 25.1) | -0.4 (-0.5, -0.4) |
| Chad | 159.5 (90.8, 260.4) | 12.1 (6.7, 19.8) | 326.9 (186.2, 528.0) | 14.0 (7.8, 22.6) | 0.4 (0.3, 0.6) |
| Chile | 371.5 (216.1, 546.5) | 7.7 (4.4, 11.5) | 413.1 (229.4, 628.1) | 2.8 (1.6, 4.2) | -2.7 (-3.0, -2.5) |
| China | 40386.7 (25903.1, 58046.0) | 11.9 (7.1, 17.5) | 88360.0 (53027.6, 131237.3) | 8.4 (4.9, 12.6) | -0.8 (-1.0, -0.5) |
| Colombia | 595.8 (384.3, 822.6) | 7.6 (4.8, 10.6) | 842.4 (504.2, 1234.3) | 2.8 (1.7, 4.1) | -3.7 (-3.9, -3.4) |
| Comoros | 7.0 (3.9, 11.1) | 9.1 (5.0, 14.6) | 13.6 (7.2, 22.1) | 6.6 (3.4, 10.7) | -1.4 (-1.6, -1.2) |
| Congo | 72.1 (41.8, 111.7) | 18.1 (10.0, 28.3) | 138.4 (80.4, 216.5) | 14.8 (8.3, 23.4) | -0.9 (-1.0, -0.8) |
| Cook Islands | 0.4 (0.2, 0.6) | 7.2 (4.0, 11.0) | 0.6 (0.3, 0.9) | 3.9 (2.1, 6.2) | -2.1 (-2.2, -1.9) |
| Costa Rica | 52.8 (32.5, 74.9) | 6.0 (3.7, 8.6) | 104.8 (61.8, 152.0) | 3.5 (2.1, 5.1) | -2.2 (-2.6, -1.7) |
| Coted'Ivoire | 219.6 (133.7, 334.2) | 15.3 (9.1, 23.3) | 610.1 (352.6, 964.4) | 14.2 (8.1, 22.4) | -0.3 (-0.5, -0.1) |
| Croatia | 544.7 (324.2, 792.9) | 19.5 (11.5, 28.4) | 379.1 (208.0, 567.0) | 6.4 (3.6, 9.6) | -3.8 (-3.9, -3.6) |
| Cuba | 318.3 (194.8, 458.8) | 5.9 (3.6, 8.5) | 532.0 (318.9, 776.3) | 4.7 (2.8, 6.8) | -0.8 (-0.9, -0.7) |
| Cyprus | 49.4 (25.3, 77.6) | 19.3 (9.3, 30.9) | 45.7 (23.1, 72.7) | 4.6 (2.3, 7.3) | -5.0 (-5.4, -4.6) |
| Czechia | 1637.7 (973.7, 2360.8) | 21.7 (12.8, 31.5) | 526.8 (293.8, 784.6) | 4.0 (2.2, 6.0) | -5.9 (-6.3, -5.4) |
| Democratic People's Republic of Korea | 697.2 (403.7, 1077.1) | 10.3 (5.8, 16.2) | 1555.8 (895.7, 2439.5) | 9.3 (5.3, 14.8) | -0.4 (-0.6, -0.2) |
| Democratic Republic of the Congo | 718.9 (414.2, 1123.3) | 13.3 (7.3, 21.1) | 1518.2 (793.9, 2607.3) | 12.1 (6.2, 21.1) | -0.5 (-0.5, -0.4) |
| Denmark | 365.4 (205.0, 543.6) | 7.2 (4.1, 10.7) | 208.3 (115.6, 310.6) | 2.7 (1.5, 4.0) | -3.5 (-3.7, -3.3) |
| Djibouti | 3.1 (1.7, 5.4) | 7.3 (3.8, 12.6) | 15.3 (8.2, 25.4) | 7.2 (3.8, 12.1) | -0.2 (-0.2, -0.1) |
| Dominica | 3.1 (1.8, 4.6) | 9.5 (5.6, 14.2) | 3.3 (1.9, 5.1) | 8.0 (4.5, 12.3) | -0.6 (-0.7, -0.5) |
| Dominican Republic | 89.5 (53.2, 134.4) | 5.7 (3.3, 8.6) | 263.1 (147.6, 415.6) | 5.0 (2.8, 7.8) | 0.0 (-0.3, 0.3) |
| Ecuador | 115.4 (71.8, 164.5) | 4.5 (2.8, 6.5) | 201.9 (115.3, 308.4) | 2.4 (1.4, 3.7) | -1.9 (-2.1, -1.6) |
| Egypt | 3028.3 (1753.3, 4790.1) | 30.5 (17.0, 48.8) | 5951.3 (3637.8, 8801.1) | 25.1 (14.5, 37.7) | -0.1 (-0.3, 0.1) |
| El Salvador | 67.2 (40.5, 97.4) | 4.4 (2.7, 6.5) | 102.4 (57.3, 158.0) | 2.8 (1.6, 4.4) | -1.5 (-1.7, -1.2) |
| Equatorial Guinea | 11.3 (6.4, 18.1) | 15.3 (8.5, 24.9) | 22.6 (12.0, 37.0) | 12.3 (6.4, 20.1) | -1.0 (-1.2, -0.8) |
| Eritrea | 27.2 (14.0, 47.2) | 8.3 (4.2, 14.3) | 66.4 (34.8, 111.4) | 7.5 (3.8, 12.6) | -0.4 (-0.5, -0.3) |
| Estonia | 299.0 (188.0, 423.4) | 26.9 (16.8, 38.2) | 91.5 (53.4, 134.3) | 5.0 (3.0, 7.2) | -7.0 (-7.6, -6.4) |
| Eswatini | 16.3 (9.3, 25.0) | 15.3 (8.5, 24.0) | 33.3 (18.3, 53.6) | 16.4 (8.8, 26.2) | 0.6 (0.2, 1.1) |
| Ethiopia | 330.6 (173.1, 570.8) | 4.6 (2.4, 8.0) | 649.3 (368.8, 1049.9) | 3.6 (2.0, 5.9) | -1.1 (-1.2, -1.0) |
| Fiji | 10.4 (6.3, 15.7) | 8.1 (4.7, 12.4) | 21.7 (12.7, 33.7) | 7.7 (4.4, 12.1) | -0.5 (-0.7, -0.3) |
| Finland | 339.7 (200.3, 500.8) | 8.3 (4.9, 12.2) | 231.9 (124.7, 353.6) | 2.5 (1.4, 3.9) | -3.7 (-3.8, -3.6) |
| France | 2794.6 (1451.2, 4233.1) | 5.4 (2.9, 8.1) | 1874.4 (953.7, 2878.0) | 1.6 (0.9, 2.5) | -4.1 (-4.4, -3.9) |
| Gabon | 34.1 (19.6, 53.3) | 13.4 (7.5, 21.2) | 48.4 (27.3, 75.4) | 12.2 (6.7, 19.1) | -0.4 (-0.6, -0.3) |
| Gambia | 20.8 (12.2, 32.6) | 14.5 (8.2, 22.8) | 70.3 (39.2, 111.5) | 16.7 (9.2, 26.5) | 0.5 (0.4, 0.6) |
| Georgia | 509.3 (305.6, 734.3) | 16.3 (9.7, 23.5) | 727.3 (438.1, 1044.4) | 20.4 (12.5, 29.1) | 0.7 (0.1, 1.4) |
| Germany | 7477.7 (4059.1, 11254.0) | 9.9 (5.4, 14.8) | 3476.8 (1850.0, 5318.9) | 2.5 (1.4, 3.8) | -4.6 (-4.9, -4.2) |
| Ghana | 345.3 (198.1, 546.0) | 13.8 (7.7, 21.9) | 987.8 (549.4, 1561.9) | 14.7 (7.9, 23.3) | 0.4 (0.2, 0.7) |
| Greece | 1264.5 (691.0, 1866.7) | 15.8 (8.6, 23.4) | 870.2 (446.1, 1324.4) | 4.2 (2.2, 6.3) | -5.0 (-5.4, -4.6) |
| Greenland | 1.7 (1.0, 2.5) | 16.7 (9.0, 25.7) | 1.4 (0.8, 2.2) | 5.7 (3.0, 9.1) | -3.8 (-3.9, -3.6) |
| Grenada | 6.3 (3.8, 9.0) | 14.0 (8.7, 19.9) | 3.9 (2.4, 5.6) | 7.8 (4.5, 11.4) | -1.9 (-2.1, -1.7) |
| Guam | 1.8 (1.1, 2.6) | 7.2 (4.2, 10.9) | 2.5 (1.5, 3.7) | 2.2 (1.3, 3.2) | -3.4 (-3.9, -3.0) |
| Guatemala | 74.4 (46.9, 105.3) | 5.9 (3.5, 8.4) | 192.4 (114.3, 276.6) | 3.6 (2.1, 5.2) | -2.1 (-2.5, -1.8) |
| Guinea | 189.7 (105.9, 299.8) | 12.5 (6.8, 19.8) | 360.3 (204.2, 564.5) | 14.5 (8.1, 22.8) | 0.8 (0.7, 0.9) |
| Guinea-Bissau | 33.2 (19.7, 51.7) | 20.0 (11.5, 31.3) | 52.2 (30.9, 79.5) | 19.8 (11.4, 30.3) | 0.1 (0.1, 0.2) |
| Guyana | 31.6 (20.9, 44.2) | 17.9 (11.6, 25.3) | 30.5 (18.8, 44.8) | 10.8 (6.5, 16.1) | -1.1 (-1.3, -0.9) |
| Haiti | 211.7 (123.8, 320.5) | 17.0 (9.6, 26.3) | 361.1 (195.1, 585.0) | 13.3 (7.0, 21.6) | -0.7 (-0.7, -0.6) |
| Honduras | 75.5 (44.5, 112.7) | 8.2 (4.8, 12.4) | 314.3 (180.0, 483.5) | 11.3 (6.3, 17.5) | 1.2 (0.9, 1.4) |
| Hungary | 1430.3 (874.3, 2053.8) | 18.6 (11.2, 27.0) | 696.2 (400.2, 1030.5) | 5.7 (3.3, 8.5) | -4.2 (-4.4, -4.0) |
| Iceland | 8.6 (4.8, 12.9) | 5.0 (2.8, 7.5) | 6.1 (3.2, 9.4) | 1.5 (0.8, 2.4) | -3.9 (-4.1, -3.8) |
| India | 14324.7 (9122.9, 21100.3) | 7.8 (4.7, 11.6) | 35746.2 (22321.0, 53617.7) | 6.5 (4.0, 9.8) | -0.7 (-0.8, -0.6) |
| Indonesia | 5836.2 (3757.2, 8416.2) | 15.2 (9.4, 22.3) | 18757.2 (11298.1, 28091.1) | 20.7 (12.2, 31.1) | 1.1 (1.0, 1.3) |
| Iran (Islamic Republic of) | 1879.1 (1267.9, 2608.1) | 18.2 (11.2, 26.3) | 3462.7 (2119.8, 4976.7) | 9.4 (5.6, 13.7) | -2.2 (-2.3, -2.1) |
| Iraq | 948.0 (568.7, 1402.7) | 24.0 (14.3, 35.5) | 2201.3 (1280.5, 3340.0) | 22.4 (12.6, 34.3) | -0.8 (-1.0, -0.6) |
| Ireland | 266.4 (166.9, 371.0) | 12.1 (7.4, 16.9) | 128.2 (75.0, 184.5) | 2.6 (1.6, 3.8) | -4.8 (-5.0, -4.6) |
| Israel | 141.3 (82.8, 206.8) | 5.7 (3.2, 8.3) | 125.2 (66.6, 192.0) | 1.6 (0.9, 2.4) | -4.6 (-4.8, -4.4) |
| Italy | 4714.7 (2677.4, 6973.1) | 9.5 (5.4, 14.1) | 2761.7 (1424.5, 4294.6) | 2.3 (1.2, 3.6) | -4.7 (-4.9, -4.4) |
| Jamaica | 96.6 (58.2, 139.7) | 9.3 (5.6, 13.5) | 116.3 (65.5, 173.8) | 6.7 (3.8, 10.0) | -0.9 (-1.3, -0.5) |
| Japan | 8437.3 (4535.8, 12647.4) | 9.9 (5.2, 15.0) | 7122.1 (3484.4, 11135.4) | 2.3 (1.2, 3.6) | -4.9 (-5.1, -4.7) |
| Jordan | 105.9 (64.9, 156.8) | 19.8 (11.7, 29.7) | 263.9 (154.2, 400.7) | 9.3 (5.2, 14.3) | -3.0 (-3.4, -2.7) |
| Kazakhstan | 1550.0 (1000.1, 2182.2) | 25.9 (16.3, 36.9) | 1617.9 (1014.3, 2286.7) | 21.3 (12.9, 30.4) | -1.2 (-1.7, -0.7) |
| Kenya | 163.1 (92.3, 258.5) | 4.9 (2.7, 7.9) | 469.7 (266.8, 725.0) | 5.8 (3.1, 9.1) | 0.7 (0.6, 0.8) |
| Kiribati | 1.3 (0.8, 1.9) | 9.0 (5.2, 13.9) | 2.5 (1.5, 3.9) | 9.7 (5.5, 15.1) | 0.2 (0.2, 0.3) |
| Kuwait | 14.7 (9.2, 20.8) | 6.3 (3.8, 9.1) | 50.3 (29.0, 76.6) | 4.3 (2.4, 6.6) | -1.0 (-2.1, 0.1) |
| Kyrgyzstan | 351.4 (227.5, 493.1) | 24.0 (15.3, 33.9) | 283.1 (183.6, 389.8) | 12.6 (7.9, 17.7) | -2.6 (-2.9, -2.2) |
| Lao People's Democratic Republic | 207.6 (125.6, 312.7) | 24.0 (14.0, 36.7) | 311.4 (183.4, 474.6) | 16.6 (9.7, 25.4) | -1.3 (-1.4, -1.2) |
| Latvia | 560.7 (344.1, 800.4) | 28.6 (17.5, 40.8) | 450.6 (264.1, 655.4) | 16.7 (10.1, 24.1) | -2.2 (-2.4, -1.9) |
| Lebanon | 123.3 (68.7, 195.3) | 12.8 (6.9, 20.4) | 171.3 (92.8, 271.2) | 4.7 (2.6, 7.4) | -3.4 (-3.7, -3.1) |
| Lesotho | 43.7 (24.8, 68.5) | 11.7 (6.4, 18.6) | 83.7 (47.9, 132.7) | 19.5 (10.8, 31.1) | 2.6 (2.1, 3.1) |
| Liberia | 72.0 (44.2, 107.9) | 14.2 (8.4, 21.3) | 111.2 (63.8, 176.5) | 13.7 (7.8, 21.7) | -0.2 (-0.3, -0.1) |
| Libya | 80.2 (44.8, 128.0) | 9.2 (5.1, 14.7) | 241.2 (130.4, 392.5) | 10.4 (5.5, 17.0) | 0.9 (0.7, 1.1) |
| Lithuania | 377.7 (238.5, 531.3) | 15.2 (9.6, 21.3) | 395.5 (229.8, 578.7) | 10.2 (6.1, 14.7) | -1.6 (-1.9, -1.2) |
| Luxembourg | 43.7 (25.3, 63.6) | 14.5 (8.3, 21.2) | 16.0 (8.9, 23.9) | 2.3 (1.3, 3.4) | -5.7 (-5.9, -5.6) |
| Madagascar | 210.2 (119.7, 325.3) | 10.1 (5.6, 15.8) | 358.3 (195.7, 575.3) | 9.6 (5.1, 15.5) | -0.3 (-0.3, -0.2) |
| Malawi | 120.2 (68.6, 190.5) | 8.3 (4.5, 13.3) | 273.0 (148.1, 442.5) | 9.7 (5.1, 15.9) | 0.3 (0.1, 0.5) |
| Malaysia | 542.9 (348.1, 790.2) | 12.6 (8.0, 18.4) | 1252.9 (773.6, 1840.5) | 9.6 (5.8, 14.3) | -0.6 (-0.8, -0.4) |
| Maldives | 5.5 (3.6, 8.1) | 17.1 (10.4, 25.6) | 11.2 (6.5, 17.2) | 7.9 (4.6, 12.1) | -2.8 (-2.9, -2.7) |
| Mali | 155.3 (86.6, 254.4) | 10.2 (5.5, 16.8) | 335.6 (185.5, 555.5) | 9.8 (5.3, 16.1) | 0.1 (-0.1, 0.2) |
| Malta | 21.0 (12.5, 30.1) | 9.7 (5.6, 14.0) | 14.2 (7.7, 21.9) | 2.2 (1.2, 3.4) | -4.8 (-5.1, -4.6) |
| Marshall Islands | 0.7 (0.4, 1.1) | 10.8 (6.0, 17.2) | 1.1 (0.6, 1.7) | 9.5 (5.2, 15.2) | -0.5 (-0.5, -0.4) |
| Mauritania | 73.3 (42.4, 117.5) | 16.1 (9.1, 26.0) | 120.3 (64.9, 197.7) | 12.7 (6.8, 20.8) | -0.9 (-1.1, -0.7) |
| Mauritius | 76.2 (52.5, 102.9) | 22.6 (15.1, 31.1) | 75.0 (49.4, 103.2) | 8.0 (5.2, 11.2) | -4.5 (-5.1, -4.0) |
| Mexico | 1422.7 (893.6, 1991.7) | 7.9 (4.8, 11.2) | 2101.2 (1315.2, 2966.1) | 3.4 (2.1, 4.8) | -2.7 (-2.9, -2.5) |
| Micronesia (Federated States of) | 2.4 (1.4, 3.7) | 11.3 (6.2, 17.9) | 2.7 (1.5, 4.3) | 9.8 (5.3, 15.6) | -0.5 (-0.6, -0.5) |
| Monaco | 5.6 (2.9, 8.8) | 11.4 (5.9, 18.0) | 3.2 (1.6, 5.0) | 4.5 (2.3, 7.2) | -3.1 (-3.3, -2.9) |
| Mongolia | 25.5 (14.9, 38.6) | 5.3 (3.0, 8.1) | 46.6 (27.0, 71.3) | 5.1 (2.8, 7.9) | -0.3 (-0.7, 0.2) |
| Montenegro | 16.6 (9.2, 26.3) | 5.4 (3.0, 8.5) | 41.0 (22.1, 64.4) | 9.0 (4.8, 14.1) | 1.9 (1.7, 2.1) |
| Morocco | 1057.6 (590.1, 1689.8) | 15.5 (8.5, 24.9) | 2393.5 (1279.8, 3843.0) | 14.8 (7.7, 23.8) | 0.0 (-0.1, 0.0) |
| Mozambique | 250.2 (144.7, 390.4) | 10.4 (5.8, 16.5) | 586.2 (313.8, 958.2) | 13.3 (6.9, 22.0) | 1.2 (1.1, 1.4) |
| Myanmar | 2097.3 (1258.8, 3188.0) | 21.4 (12.5, 32.4) | 3265.6 (1889.2, 5039.4) | 15.1 (8.6, 23.5) | -1.3 (-1.4, -1.2) |
| Namibia | 44.5 (27.3, 64.6) | 18.4 (10.7, 27.4) | 88.5 (52.1, 135.0) | 16.7 (9.6, 25.9) | -0.6 (-0.8, -0.3) |
| Nauru | 0.3 (0.2, 0.4) | 15.6 (8.7, 24.2) | 0.3 (0.2, 0.5) | 14.8 (8.3, 23.3) | -0.3 (-0.6, 0.0) |
| Nepal | 467.2 (259.2, 734.2) | 12.6 (6.7, 20.0) | 992.9 (539.0, 1642.0) | 9.6 (5.0, 15.9) | -1.0 (-1.2, -0.9) |
| Netherlands | 828.8 (473.9, 1225.3) | 7.1 (4.1, 10.5) | 679.9 (377.6, 1006.9) | 3.0 (1.7, 4.4) | -3.4 (-3.7, -3.1) |
| New Zealand | 146.0 (81.2, 216.9) | 6.9 (3.8, 10.3) | 141.5 (73.2, 216.2) | 2.7 (1.4, 4.1) | -3.3 (-3.5, -3.2) |
| Nicaragua | 46.0 (27.9, 67.5) | 6.7 (4.0, 9.9) | 91.5 (54.2, 137.6) | 3.8 (2.2, 5.8) | -1.8 (-2.0, -1.6) |
| Niger | 98.0 (52.6, 167.3) | 9.9 (5.1, 16.9) | 298.8 (160.1, 505.8) | 9.9 (5.2, 16.7) | 0.2 (0.1, 0.2) |
| Nigeria | 3278.7 (1948.1, 5101.4) | 17.1 (9.9, 26.8) | 4788.1 (2949.9, 7018.6) | 13.4 (8.1, 19.7) | -0.9 (-1.0, -0.7) |
| Niue | 0.1 (0.1, 0.2) | 10.2 (5.9, 15.9) | 0.1 (0.1, 0.2) | 8.5 (4.9, 13.1) | -0.8 (-0.9, -0.7) |
| North Macedonia | 278.6 (168.9, 404.4) | 32.7 (19.3, 48.1) | 468.5 (263.3, 704.7) | 36.2 (19.4, 55.3) | 0.0 (-0.6, 0.6) |
| Northern Mariana Islands | 0.4 (0.3, 0.7) | 8.8 (5.0, 13.6) | 1.1 (0.7, 1.7) | 6.0 (3.5, 9.0) | -1.7 (-1.9, -1.4) |
| Norway | 333.4 (191.7, 491.7) | 7.6 (4.4, 11.2) | 126.9 (68.4, 193.7) | 1.9 (1.0, 2.9) | -4.8 (-4.9, -4.6) |
| Oman | 40.9 (23.2, 64.2) | 13.9 (7.7, 21.8) | 70.3 (41.4, 107.3) | 9.9 (5.6, 15.3) | -0.5 (-0.8, -0.2) |
| Pakistan | 2121.4 (1207.8, 3388.2) | 8.5 (4.7, 13.7) | 4400.2 (2652.3, 6815.3) | 9.1 (5.3, 14.3) | -0.1 (-0.2, 0.1) |
| Palau | 0.5 (0.3, 0.7) | 11.3 (6.5, 17.3) | 0.9 (0.5, 1.3) | 10.0 (5.8, 15.4) | -0.2 (-0.3, -0.1) |
| Palestine | 95.3 (54.0, 146.4) | 24.5 (13.6, 38.0) | 142.3 (85.5, 210.8) | 14.7 (8.5, 22.0) | -1.7 (-2.0, -1.4) |
| Panama | 52.1 (32.6, 74.5) | 7.2 (4.4, 10.4) | 109.3 (63.0, 161.6) | 4.5 (2.6, 6.6) | -1.7 (-1.9, -1.5) |
| Papua New Guinea | 43.5 (23.1, 72.4) | 7.5 (3.8, 12.5) | 114.3 (62.0, 188.1) | 6.9 (3.6, 11.3) | -0.4 (-0.5, -0.2) |
| Paraguay | 99.7 (60.1, 146.1) | 9.4 (5.6, 13.9) | 180.6 (103.3, 276.1) | 6.5 (3.7, 9.9) | -0.9 (-1.1, -0.7) |
| Peru | 180.6 (103.0, 279.8) | 3.2 (1.8, 4.9) | 320.9 (172.4, 514.3) | 1.8 (1.0, 2.9) | -2.4 (-2.9, -1.9) |
| Philippines | 1251.8 (819.1, 1752.6) | 11.6 (7.3, 16.4) | 3528.9 (2328.6, 4958.1) | 9.9 (6.3, 14.1) | -0.3 (-0.4, -0.2) |
| Poland | 4227.5 (2553.6, 6034.0) | 19.2 (11.4, 27.6) | 2773.7 (1601.7, 4093.3) | 6.2 (3.6, 9.1) | -4.1 (-4.3, -4.0) |
| Portugal | 1460.9 (858.6, 2113.3) | 21.3 (12.2, 31.1) | 602.0 (327.4, 919.0) | 3.2 (1.8, 4.8) | -6.5 (-6.7, -6.4) |
| Puerto Rico | 79.0 (47.6, 114.6) | 4.3 (2.6, 6.3) | 65.2 (36.4, 98.4) | 1.3 (0.8, 2.0) | -4.1 (-4.3, -3.9) |
| Qatar | 4.1 (2.3, 6.3) | 15.2 (8.3, 23.6) | 11.9 (6.5, 19.3) | 5.9 (3.1, 9.6) | -3.6 (-4.4, -2.8) |
| Republic of Korea | 2096.0 (1236.6, 3101.3) | 19.0 (10.3, 28.9) | 1874.5 (926.4, 3025.8) | 3.7 (1.8, 5.9) | -6.0 (-6.2, -5.7) |
| Republic of Moldova | 336.9 (212.5, 474.7) | 18.0 (10.8, 25.7) | 401.3 (261.0, 557.4) | 12.0 (7.8, 16.7) | -1.1 (-1.5, -0.6) |
| Romania | 2726.7 (1608.5, 3964.3) | 22.3 (12.6, 32.9) | 2884.4 (1572.0, 4365.3) | 12.2 (6.8, 18.3) | -2.5 (-2.7, -2.2) |
| Russian Federation | 28119.6 (17831.5, 39433.2) | 32.5 (20.1, 46.1) | 23490.5 (14239.6, 33685.8) | 17.4 (10.6, 24.9) | -2.9 (-3.4, -2.3) |
| Rwanda | 129.8 (72.8, 207.1) | 12.0 (6.5, 19.3) | 147.1 (75.4, 244.3) | 6.5 (3.2, 10.8) | -2.9 (-3.3, -2.5) |
| Saint Kitts and Nevis | 4.0 (2.5, 5.8) | 19.8 (11.9, 28.7) | 2.9 (1.8, 4.1) | 10.4 (6.2, 15.1) | -1.9 (-2.1, -1.7) |
| Saint Lucia | 6.8 (4.2, 9.7) | 17.3 (10.4, 25.1) | 9.3 (5.4, 13.8) | 7.3 (4.2, 10.8) | -3.6 (-4.0, -3.1) |
| Saint Vincent and the Grenadines | 3.9 (2.4, 5.6) | 10.7 (6.4, 15.5) | 4.9 (3.0, 7.1) | 6.9 (4.1, 10.0) | -1.3 (-1.5, -1.0) |
| Samoa | 3.1 (1.8, 4.8) | 8.9 (5.0, 13.7) | 4.8 (2.8, 7.4) | 7.5 (4.3, 11.7) | -0.6 (-0.7, -0.5) |
| San Marino | 1.5 (0.8, 2.2) | 7.0 (3.7, 10.8) | 1.2 (0.6, 1.9) | 2.0 (1.0, 3.4) | -3.4 (-3.8, -3.1) |
| Sao,me and Principe | 3.8 (2.3, 5.8) | 12.6 (7.5, 19.0) | 6.8 (4.1, 10.2) | 14.5 (8.7, 21.9) | 0.7 (0.6, 0.8) |
| Saudi Arabia | 435.4 (249.6, 674.0) | 17.8 (9.9, 27.7) | 820.8 (495.4, 1252.2) | 12.0 (6.9, 18.7) | -1.5 (-1.7, -1.4) |
| Senegal | 200.5 (118.2, 311.7) | 14.0 (8.1, 22.1) | 418.3 (237.1, 670.0) | 12.6 (7.0, 20.2) | -0.5 (-0.5, -0.4) |
| Serbia | 1220.4 (719.1, 1803.2) | 27.2 (15.8, 40.6) | 1409.0 (817.7, 2127.0) | 14.7 (8.5, 22.2) | -2.5 (-2.8, -2.3) |
| Seychelles | 4.2 (2.6, 6.1) | 13.5 (8.3, 19.7) | 5.0 (3.0, 7.4) | 9.3 (5.5, 13.8) | -0.8 (-1.1, -0.6) |
| Sierra Leone | 153.1 (90.7, 231.9) | 16.0 (9.3, 24.4) | 242.6 (136.6, 379.4) | 15.0 (8.4, 23.5) | 0.0 (-0.2, 0.2) |
| Singapore | 117.6 (74.3, 164.4) | 11.9 (7.3, 16.9) | 56.0 (31.5, 84.3) | 1.3 (0.7, 1.9) | -7.1 (-7.5, -6.6) |
| Slovakia | 438.3 (255.6, 638.9) | 13.9 (8.0, 20.3) | 352.0 (200.2, 532.4) | 6.6 (3.7, 10.0) | -2.5 (-2.5, -2.4) |
| Slovenia | 157.4 (93.4, 230.7) | 11.7 (6.9, 17.1) | 100.7 (53.6, 153.7) | 3.3 (1.8, 4.9) | -4.2 (-4.4, -3.9) |
| Solomon Islands | 4.2 (2.4, 6.9) | 9.6 (5.1, 15.6) | 11.3 (6.1, 18.9) | 9.2 (4.9, 15.4) | -0.1 (-0.3, 0.0) |
| Somalia | 54.0 (25.0, 97.3) | 7.0 (3.2, 12.5) | 111.2 (53.5, 198.9) | 5.8 (2.7, 10.4) | -0.6 (-0.6, -0.6) |
| South Africa | 871.5 (527.6, 1267.1) | 9.3 (5.5, 13.6) | 2475.5 (1609.2, 3481.1) | 12.4 (7.8, 17.7) | 1.0 (0.4, 1.5) |
| South Sudan | 77.7 (41.7, 128.3) | 7.0 (3.7, 11.6) | 83.4 (43.5, 144.2) | 6.0 (3.0, 10.3) | -0.7 (-0.9, -0.5) |
| Spain | 3019.8 (1638.2, 4534.5) | 10.1 (5.4, 15.3) | 1394.8 (706.3, 2174.5) | 1.8 (0.9, 2.7) | -5.6 (-5.8, -5.4) |
| Sri Lanka | 846.6 (529.3, 1214.7) | 20.3 (12.4, 29.4) | 1743.5 (934.5, 2715.8) | 13.8 (7.3, 21.5) | -0.5 (-0.8, -0.2) |
| Sudan | 775.7 (434.4, 1211.2) | 18.3 (9.9, 28.9) | 1253.6 (670.1, 2037.7) | 14.7 (7.8, 23.9) | -0.8 (-0.9, -0.8) |
| Suriname | 10.1 (6.1, 15.0) | 8.4 (5.0, 12.5) | 19.7 (10.9, 30.9) | 6.3 (3.4, 9.9) | -0.9 (-1.2, -0.6) |
| Sweden | 700.5 (402.0, 1034.3) | 7.1 (4.1, 10.5) | 376.1 (204.4, 576.5) | 2.4 (1.3, 3.7) | -3.6 (-3.8, -3.3) |
| Switzerland | 470.4 (259.5, 706.9) | 7.0 (3.9, 10.5) | 239.1 (124.7, 367.1) | 1.8 (1.0, 2.7) | -4.2 (-4.4, -4.1) |
| Syrian Arab Republic | 334.0 (198.1, 510.3) | 14.5 (8.4, 22.4) | 630.4 (355.5, 989.5) | 11.5 (6.3, 18.2) | -1.3 (-1.5, -1.0) |
| Taiwan (Province of China) | 629.2 (417.5, 871.1) | 9.4 (5.9, 13.3) | 490.8 (283.3, 729.0) | 2.0 (1.2, 3.0) | -4.7 (-5.0, -4.5) |
| Tajikistan | 232.1 (142.4, 344.0) | 17.5 (10.6, 26.0) | 328.9 (198.4, 489.7) | 15.0 (8.7, 22.5) | -0.8 (-1.2, -0.4) |
| Thailand | 1469.9 (896.2, 2183.6) | 9.8 (5.8, 14.6) | 3703.2 (2154.6, 5595.0) | 6.2 (3.6, 9.4) | -2.2 (-2.4, -1.9) |
| Timor-Leste | 12.3 (7.4, 18.5) | 13.1 (7.6, 19.7) | 56.5 (31.5, 89.0) | 14.7 (8.1, 23.2) | 0.6 (0.5, 0.8) |
| Togo | 73.4 (44.8, 111.0) | 14.9 (8.8, 22.7) | 211.8 (120.2, 327.7) | 14.8 (8.2, 23.1) | -0.2 (-0.3, 0.0) |
| Tokelau | 0.1 (0.0, 0.1) | 10.5 (5.8, 16.3) | 0.1 (0.0, 0.1) | 7.4 (4.1, 11.7) | -1.2 (-1.3, -1.2) |
| Tonga | 1.3 (0.7, 2.0) | 5.4 (3.1, 8.3) | 2.2 (1.2, 3.4) | 5.2 (2.9, 8.3) | 0.1 (0.0, 0.2) |
| Trinidad and,bago | 48.2 (30.7, 68.5) | 12.5 (7.7, 18.0) | 63.6 (36.5, 94.9) | 6.3 (3.6, 9.5) | -2.4 (-2.6, -2.2) |
| Tunisia | 281.0 (161.1, 440.4) | 13.4 (7.4, 21.4) | 645.3 (337.7, 1059.9) | 10.0 (5.2, 16.5) | -1.1 (-1.3, -1.0) |
| Turkey | 2480.9 (1493.2, 3720.9) | 16.3 (9.6, 24.6) | 3776.9 (2095.1, 5822.7) | 8.4 (4.6, 13.0) | -2.0 (-2.3, -1.7) |
| Turkmenistan | 157.1 (102.9, 216.6) | 17.4 (11.1, 24.4) | 381.2 (236.1, 547.2) | 20.8 (12.6, 30.0) | 0.2 (-0.3, 0.7) |
| Tuvalu | 0.3 (0.2, 0.5) | 12.3 (7.0, 19.0) | 0.5 (0.3, 0.7) | 9.9 (5.5, 15.3) | -0.7 (-0.8, -0.7) |
| Uganda | 189.4 (104.3, 309.9) | 7.3 (3.9, 12.2) | 310.2 (164.3, 518.5) | 5.6 (2.9, 9.4) | -1.6 (-1.9, -1.3) |
| Ukraine | 10904.7 (6872.8, 15238.9) | 30.2 (18.6, 42.4) | 7012.8 (3952.1, 10476.0) | 15.7 (8.9, 23.4) | -2.9 (-3.2, -2.5) |
| United Arab Emirates | 26.2 (15.2, 40.7) | 17.5 (9.9, 27.2) | 76.3 (45.7, 116.3) | 11.1 (6.1, 17.1) | 0.4 (-0.2, 1.0) |
| United Kingdom | 5499.4 (3016.6, 8175.9) | 10.0 (5.5, 14.8) | 2054.6 (1094.2, 3118.4) | 2.3 (1.2, 3.4) | -5.0 (-5.2, -4.8) |
| United Republic of Tanzania | 225.7 (124.2, 369.5) | 5.2 (2.8, 8.7) | 735.3 (374.9, 1224.2) | 7.1 (3.6, 11.8) | 1.1 (0.9, 1.3) |
| United States of America | 9594.1 (5370.8, 14153.7) | 5.1 (2.8, 7.5) | 11029.9 (6105.5, 16447.2) | 3.1 (1.7, 4.6) | -2.2 (-2.6, -1.9) |
| United States Virgin Islands | 2.0 (1.2, 3.1) | 6.0 (3.3, 9.2) | 2.7 (1.5, 4.2) | 2.6 (1.4, 4.2) | -2.5 (-2.6, -2.3) |
| Uruguay | 217.9 (122.4, 325.6) | 10.1 (5.6, 15.2) | 164.6 (89.7, 247.0) | 4.4 (2.5, 6.5) | -2.8 (-3.0, -2.6) |
| Uzbekistan | 794.8 (531.3, 1084.7) | 13.5 (9.0, 18.5) | 1533.0 (1004.3, 2162.7) | 13.7 (8.6, 19.6) | -0.6 (-1.0, -0.3) |
| Vanuatu | 2.5 (1.5, 3.9) | 11.5 (6.3, 17.9) | 6.1 (3.6, 9.5) | 9.7 (5.5, 15.0) | -0.7 (-0.8, -0.6) |
| Venezuela (Bolivarian Republic of) | 296.2 (184.0, 421.4) | 6.7 (4.1, 9.6) | 785.2 (447.0, 1178.8) | 5.5 (3.1, 8.3) | -0.9 (-1.2, -0.6) |
| Viet Nam | 2947.6 (1706.7, 4544.5) | 15.5 (8.9, 24.1) | 7153.4 (4059.0, 11237.9) | 16.1 (9.1, 25.3) | 0.6 (0.3, 0.8) |
| Yemen | 393.2 (218.8, 633.8) | 19.4 (10.4, 31.6) | 1089.1 (583.7, 1785.4) | 18.4 (9.6, 30.4) | -0.4 (-0.4, -0.3) |
| Zambia | 75.7 (40.6, 128.4) | 7.1 (3.7, 12.1) | 218.1 (118.3, 357.5) | 8.9 (4.7, 14.6) | 0.6 (0.5, 0.7) |
| Zimbabwe | 163.1 (99.0, 248.8) | 10.7 (6.2, 16.7) | 383.4 (231.4, 574.5) | 15.4 (8.9, 23.2) | 1.8 (1.3, 2.3) |
| **DALYs** |  |  |  |  |  |
| Global | 4619125.3 (3138719.8, 6312514.5) | 229.6 (151.8, 317.6) | 6808070.0 (4519000.4, 9385703.3) | 145.0 (95.4, 200.8) | -1.7 (-1.7, -1.6) |
| High SDI | 928290.2 (582479.0, 1311158.0) | 148.5 (93.2, 209.9) | 767038.8 (469257.5, 1108585.4) | 61.3 (38.5, 87.6) | -3.2 (-3.3, -3.0) |
| High-middle SDI | 1648384.7 (1105788.4, 2252621.8) | 324.9 (212.4, 449.4) | 1850798.7 (1207112.8, 2580565.9) | 168.9 (109.6, 236.2) | -2.5 (-2.7, -2.2) |
| Middle SDI | 1178095.3 (824574.4, 1602997.7) | 233.4 (157.4, 323.3) | 2395986.7 (1609493.4, 3313227.7) | 169.6 (111.8, 236.7) | -1.0 (-1.0, -0.9) |
| Low-middle SDI | 641574.7 (444030.1, 883886.6) | 216.2 (144.9, 302.1) | 1374292.5 (931086.5, 1894351.4) | 188.0 (124.3, 261.7) | -0.5 (-0.5, -0.5) |
| Low SDI | 216657.4 (146229.6, 313225.2) | 200.7 (130.7, 294.4) | 413613.1 (274229.6, 593255.2) | 169.5 (108.9, 245.5) | -0.6 (-0.7, -0.6) |
| High-income Asia Pacific | 202092.5 (124287.8, 290190.0) | 192.9 (115.6, 280.3) | 168782.1 (95020.4, 255718.9) | 57.6 (34.3, 85.6) | -4.2 (-4.4, -4.0) |
| High-income North America | 193055.4 (117987.5, 276781.7) | 94.5 (58.0, 135.3) | 234304.7 (143495.0, 338335.4) | 61.6 (38.1, 88.7) | -1.9 (-2.1, -1.6) |
| Western Europe | 488262.5 (295506.8, 701873.7) | 143.0 (87.2, 205.0) | 253332.2 (148260.9, 373822.0) | 41.1 (25.1, 59.6) | -4.2 (-4.4, -4.0) |
| Australasia | 13917.3 (8286.8, 20166.0) | 107.9 (63.0, 157.5) | 11344.9 (6401.9, 16917.9) | 34.1 (19.6, 50.7) | -4.0 (-4.1, -3.8) |
| Andean Latin America | 7823.5 (5036.1, 11044.5) | 74.6 (47.3, 106.1) | 13385.2 (8260.6, 19580.0) | 42.4 (26.0, 62.2) | -2.0 (-2.2, -1.8) |
| Tropical Latin America | 112414.7 (78834.3, 148605.7) | 249.7 (169.4, 336.1) | 127196.4 (87167.3, 170855.4) | 92.1 (62.4, 124.5) | -3.2 (-3.3, -3.0) |
| Central Latin America | 53748.2 (36687.3, 72610.7) | 131.0 (87.4, 178.8) | 92068.0 (61512.2, 125246.2) | 69.5 (45.9, 95.1) | -2.2 (-2.4, -2.0) |
| Southern Latin America | 30305.3 (18894.6, 43664.6) | 123.5 (75.5, 179.3) | 25174.2 (15485.2, 36630.9) | 50.4 (31.2, 73.3) | -2.7 (-2.8, -2.5) |
| Caribbean | 18557.0 (12278.9, 25570.1) | 134.6 (88.1, 186.5) | 29633.4 (19166.3, 42109.7) | 100.1 (64.7, 142.4) | -0.9 (-1.0, -0.8) |
| Central Europe | 263412.5 (172574.8, 364243.6) | 337.7 (216.6, 471.0) | 204251.7 (127469.6, 288372.1) | 156.0 (98.5, 219.3) | -2.9 (-3.0, -2.7) |
| Eastern Europe | 803603.2 (539812.5, 1095107.7) | 542.8 (359.0, 744.9) | 608476.3 (398034.8, 844225.8) | 307.1 (201.5, 425.8) | -2.6 (-3.1, -2.2) |
| Central Asia | 91703.6 (65209.4, 120795.8) | 373.8 (261.7, 496.4) | 127867.6 (90286.1, 170005.4) | 312.7 (214.5, 421.6) | -1.1 (-1.3, -0.8) |
| North Africa and Middle East | 306941.4 (208591.6, 426102.5) | 365.4 (239.8, 516.3) | 577843.9 (378934.5, 803199.5) | 250.9 (159.4, 353.2) | -1.2 (-1.2, -1.2) |
| South Asia | 454709.2 (305096.1, 658944.7) | 166.5 (107.8, 243.0) | 1031052.7 (678443.4, 1516789.0) | 138.4 (88.7, 204.2) | -0.8 (-0.9, -0.7) |
| Southeast Asia | 368186.4 (254380.7, 504686.6) | 295.6 (199.4, 410.4) | 928397.0 (616789.1, 1297591.2) | 280.2 (182.4, 394.5) | -0.1 (-0.2, 0.0) |
| East Asia | 978804.1 (662920.5, 1371681.2) | 229.6 (148.5, 328.4) | 1906611.5 (1226043.8, 2751838.6) | 162.3 (102.4, 236.0) | -0.8 (-1.0, -0.6) |
| Oceania | 2122.1 (1355.5, 3109.8) | 161.3 (98.6, 239.6) | 4723.6 (3056.7, 6954.6) | 139.5 (86.8, 208.2) | -0.6 (-0.6, -0.5) |
| Western Sub-Saharan Africa | 126000.7 (83849.8, 182913.6) | 292.6 (188.8, 429.5) | 238918.0 (160684.7, 331142.8) | 255.4 (166.8, 358.3) | -0.4 (-0.5, -0.3) |
| Eastern Sub-Saharan Africa | 48944.6 (31483.9, 71963.3) | 139.9 (87.0, 208.9) | 100563.7 (63724.7, 147685.1) | 127.8 (78.5, 190.9) | -0.4 (-0.4, -0.3) |
| Central Sub-Saharan Africa | 27143.7 (17603.6, 39010.1) | 272.0 (168.7, 399.2) | 56280.5 (35183.2, 84520.6) | 240.4 (143.0, 369.0) | -0.6 (-0.7, -0.5) |
| Southern Sub-Saharan Africa | 27377.4 (18405.6, 37479.5) | 202.7 (133.0, 281.0) | 67862.7 (47289.2, 91798.2) | 244.8 (165.4, 335.9) | 0.7 (0.3, 1.1) |
| Afghanistan | 15626.9 (8926.8, 25212.6) | 431.1 (241.6, 700.2) | 18959.7 (11008.9, 30799.3) | 393.5 (225.1, 635.7) | -0.4 (-0.6, -0.3) |
| Albania | 1272.5 (782.6, 1858.3) | 130.1 (77.5, 192.9) | 2232.3 (1219.1, 3455.4) | 93.5 (50.5, 145.3) | -0.8 (-1.0, -0.5) |
| Algeria | 17196.1 (10350.9, 26260.8) | 314.6 (180.6, 490.8) | 36712.7 (21671.2, 56188.0) | 219.5 (124.9, 337.4) | -1.2 (-1.2, -1.1) |
| American Samoa | 18.1 (11.9, 25.7) | 173.7 (109.6, 250.0) | 33.4 (21.8, 48.6) | 140.6 (89.1, 206.5) | -0.9 (-1.0, -0.8) |
| Andorra | 20.2 (11.5, 32.0) | 70.5 (39.3, 112.7) | 31.4 (17.2, 50.3) | 34.6 (19.1, 55.4) | -2.2 (-2.4, -2.0) |
| Angola | 4766.9 (3035.9, 6995.8) | 282.0 (172.3, 422.8) | 12912.6 (8088.0, 18893.5) | 257.6 (155.7, 384.2) | -0.5 (-0.6, -0.4) |
| Antigua and Barbuda | 44.7 (28.5, 63.2) | 140.8 (91.2, 197.2) | 48.8 (32.0, 68.2) | 90.0 (57.8, 127.2) | -1.7 (-1.9, -1.5) |
| Argentina | 19414.1 (11990.4, 28249.7) | 113.0 (68.5, 165.7) | 15029.7 (9285.5, 21958.5) | 47.0 (29.1, 68.6) | -2.6 (-2.7, -2.4) |
| Armenia | 4224.6 (2939.1, 5668.5) | 313.0 (214.0, 423.5) | 5076.8 (3377.5, 6918.7) | 211.6 (141.1, 288.6) | -2.2 (-2.5, -1.9) |
| Australia | 11475.8 (6800.7, 16683.9) | 107.2 (62.2, 157.1) | 9130.5 (5129.4, 13643.1) | 32.4 (18.6, 48.3) | -4.1 (-4.3, -4.0) |
| Austria | 10870.9 (6445.9, 15749.4) | 152.6 (91.3, 219.9) | 4881.4 (2869.6, 7135.4) | 42.9 (26.0, 62.1) | -4.3 (-4.6, -4.0) |
| Azerbaijan | 5417.1 (3615.9, 7449.8) | 211.5 (139.8, 292.1) | 8777.7 (5628.3, 12624.5) | 179.4 (111.6, 260.9) | -0.5 (-0.7, -0.2) |
| Bahamas | 85.1 (55.6, 119.6) | 107.7 (69.1, 152.4) | 149.3 (94.7, 215.1) | 73.7 (45.6, 107.3) | -1.2 (-1.3, -1.1) |
| Bahrain | 213.8 (138.9, 307.7) | 293.6 (180.3, 434.4) | 558.4 (350.5, 814.1) | 158.8 (92.5, 239.0) | -2.6 (-3.0, -2.2) |
| Bangladesh | 55657.9 (33604.0, 86346.8) | 244.2 (144.7, 378.8) | 141125.2 (80564.1, 220865.3) | 207.3 (116.4, 324.1) | -0.6 (-0.8, -0.3) |
| Barbados | 278.2 (174.5, 400.5) | 159.3 (101.3, 227.6) | 292.7 (177.9, 426.3) | 100.1 (60.7, 146.0) | -1.7 (-1.9, -1.5) |
| Belarus | 29323.8 (19662.7, 40307.7) | 412.9 (275.2, 568.3) | 25543.7 (16328.5, 36125.4) | 281.3 (180.1, 397.4) | -2.0 (-2.4, -1.5) |
| Belgium | 11979.5 (7116.9, 17299.6) | 132.5 (79.4, 190.8) | 6000.7 (3510.7, 8811.7) | 39.8 (24.2, 57.6) | -3.7 (-3.9, -3.6) |
| Belize | 38.0 (24.6, 54.1) | 75.8 (48.9, 107.9) | 100.1 (65.7, 140.3) | 67.4 (43.2, 95.5) | -0.7 (-1.2, -0.3) |
| Benin | 2995.6 (1921.2, 4401.5) | 294.7 (186.4, 434.9) | 6303.7 (3918.3, 9428.7) | 257.8 (157.6, 388.1) | -0.4 (-0.5, -0.3) |
| Bermuda | 38.6 (25.1, 54.9) | 117.9 (75.6, 168.7) | 37.7 (23.1, 54.9) | 46.8 (29.1, 67.8) | -3.0 (-3.3, -2.8) |
| Bhutan | 180.4 (106.4, 280.9) | 166.7 (94.2, 264.6) | 418.2 (244.2, 639.6) | 133.4 (77.2, 205.0) | -0.8 (-0.8, -0.7) |
| Bolivia (Plurinational State of) | 1887.0 (1041.8, 3012.2) | 119.7 (65.2, 191.4) | 3216.2 (1716.0, 5183.3) | 71.0 (37.2, 114.9) | -1.7 (-1.8, -1.5) |
| Bosnia and Herzegovina | 6323.9 (4139.6, 8952.7) | 322.7 (206.1, 461.2) | 8116.4 (4843.2, 11796.3) | 228.4 (136.8, 331.8) | -1.4 (-1.5, -1.3) |
| Botswana | 834.3 (525.0, 1204.5) | 330.5 (199.5, 485.7) | 1582.8 (1010.5, 2311.7) | 240.8 (149.1, 355.9) | -1.0 (-1.2, -0.8) |
| Brazil | 110624.7 (77614.8, 146233.9) | 252.1 (171.1, 339.3) | 123897.0 (84838.7, 166409.7) | 91.7 (62.1, 123.9) | -3.2 (-3.4, -3.1) |
| Brunei Darussalam | 143.5 (90.9, 208.6) | 298.3 (184.9, 436.6) | 204.0 (126.6, 294.0) | 132.9 (77.5, 195.6) | -2.6 (-2.8, -2.4) |
| Bulgaria | 24162.1 (16070.7, 32909.2) | 410.7 (257.5, 573.1) | 28325.3 (17718.2, 40116.7) | 337.7 (212.9, 476.8) | -0.5 (-0.7, -0.4) |
| Burkina Faso | 3346.0 (2066.4, 5220.9) | 159.4 (96.2, 250.0) | 7120.0 (4327.1, 10979.2) | 158.8 (94.8, 245.8) | 0.2 (0.1, 0.3) |
| Burundi | 2787.1 (1578.8, 4401.4) | 237.7 (132.0, 378.5) | 3121.6 (1787.5, 4980.2) | 141.6 (78.5, 229.2) | -2.2 (-2.4, -2.0) |
| Cabo Verde | 230.8 (141.2, 342.9) | 178.3 (110.1, 263.6) | 517.4 (314.8, 781.1) | 224.2 (136.4, 338.0) | 0.4 (0.1, 0.7) |
| Cambodia | 6070.6 (3868.6, 8962.1) | 288.5 (178.6, 429.8) | 14711.3 (9201.1, 21596.5) | 257.6 (157.3, 380.4) | -0.5 (-0.6, -0.4) |
| Cameroon | 5415.1 (3346.0, 8257.0) | 256.5 (155.0, 391.4) | 18703.1 (11492.2, 28727.8) | 311.2 (188.1, 478.4) | 0.7 (0.2, 1.3) |
| Canada | 16705.9 (10150.9, 24082.2) | 92.8 (55.8, 134.3) | 16002.5 (9351.3, 23785.0) | 37.4 (22.1, 55.4) | -3.1 (-3.2, -2.9) |
| Central African Republic | 1643.1 (992.6, 2495.8) | 330.5 (192.8, 509.4) | 2607.5 (1536.6, 4095.8) | 290.3 (163.3, 460.2) | -0.5 (-0.6, -0.5) |
| Chad | 3493.4 (2121.7, 5527.4) | 238.6 (142.2, 379.4) | 7609.4 (4580.2, 12002.7) | 274.0 (161.5, 432.2) | 0.4 (0.2, 0.6) |
| Chile | 7023.6 (4371.3, 10057.3) | 136.0 (82.9, 196.4) | 7487.8 (4496.7, 11001.5) | 51.5 (31.0, 75.6) | -2.8 (-2.9, -2.6) |
| China | 946137.4 (639249.4, 1328441.3) | 230.5 (148.7, 330.4) | 1856478.9 (1189805.2, 2684735.8) | 163.9 (103.0, 238.8) | -0.8 (-1.0, -0.5) |
| Colombia | 12369.8 (8437.1, 16691.9) | 141.9 (94.7, 193.4) | 16276.1 (10451.1, 23114.6) | 54.0 (34.6, 76.8) | -3.6 (-3.9, -3.3) |
| Comoros | 171.3 (102.7, 260.7) | 183.9 (107.7, 283.1) | 311.3 (179.6, 486.2) | 131.1 (74.0, 206.1) | -1.4 (-1.6, -1.2) |
| Congo | 1769.0 (1092.9, 2653.2) | 357.8 (213.1, 541.8) | 3368.6 (2097.3, 5058.4) | 286.1 (172.3, 434.6) | -1.0 (-1.1, -0.9) |
| Cook Islands | 9.0 (5.7, 13.2) | 146.4 (89.7, 216.2) | 13.1 (8.1, 19.6) | 92.2 (56.9, 138.4) | -1.6 (-1.7, -1.4) |
| Costa Rica | 1003.8 (663.6, 1382.3) | 109.6 (71.9, 151.4) | 1911.4 (1220.9, 2682.4) | 64.0 (40.7, 90.0) | -2.1 (-2.5, -1.7) |
| Coted'Ivoire | 5744.8 (3652.7, 8507.1) | 308.6 (191.9, 458.9) | 15062.9 (9176.4, 23216.1) | 281.8 (168.9, 433.1) | -0.4 (-0.6, -0.2) |
| Croatia | 9460.2 (6039.6, 13337.3) | 319.0 (201.0, 452.0) | 6058.9 (3597.9, 8810.1) | 108.8 (66.0, 157.1) | -3.6 (-3.7, -3.5) |
| Cuba | 5774.7 (3741.1, 8138.6) | 103.0 (66.5, 145.3) | 9300.4 (5910.1, 13233.7) | 84.0 (53.7, 119.2) | -0.6 (-0.7, -0.5) |
| Cyprus | 757.0 (414.5, 1156.5) | 247.0 (125.2, 387.2) | 635.7 (348.0, 986.0) | 59.5 (32.0, 92.4) | -5.0 (-5.3, -4.7) |
| Czechia | 28363.8 (18019.1, 39864.9) | 367.4 (232.3, 517.2) | 9752.3 (5882.4, 14053.7) | 76.8 (46.6, 110.5) | -5.4 (-5.7, -5.1) |
| Democratic People's Republic of Korea | 17659.4 (10955.7, 26166.0) | 222.0 (134.5, 332.9) | 36790.2 (22569.3, 55071.2) | 207.5 (126.1, 312.6) | -0.3 (-0.5, -0.1) |
| Democratic Republic of the Congo | 17931.7 (11001.4, 26815.1) | 259.1 (152.3, 394.7) | 35746.1 (20714.2, 58187.3) | 229.2 (127.5, 379.2) | -0.6 (-0.6, -0.5) |
| Denmark | 6018.1 (3656.6, 8657.7) | 122.0 (75.1, 174.7) | 3378.3 (2014.2, 4865.3) | 46.0 (27.7, 66.1) | -3.5 (-3.7, -3.3) |
| Djibouti | 85.9 (50.0, 140.4) | 149.0 (83.6, 245.7) | 411.8 (240.7, 654.2) | 146.7 (82.5, 236.7) | -0.2 (-0.2, -0.1) |
| Dominica | 52.2 (32.9, 76.0) | 156.5 (97.7, 228.8) | 58.1 (35.1, 87.0) | 132.6 (79.0, 199.6) | -0.6 (-0.7, -0.4) |
| Dominican Republic | 1831.7 (1162.8, 2670.5) | 100.2 (62.3, 147.1) | 5286.3 (3177.9, 8093.1) | 97.7 (58.5, 149.7) | 0.2 (0.1, 0.4) |
| Ecuador | 2399.6 (1587.5, 3342.9) | 86.5 (56.5, 121.0) | 3877.1 (2397.0, 5679.6) | 44.2 (27.2, 64.9) | -2.0 (-2.3, -1.7) |
| Egypt | 68043.3 (40798.7, 106791.1) | 549.3 (320.1, 866.5) | 141609.4 (90055.3, 205642.9) | 471.4 (287.6, 693.7) | -0.1 (-0.3, 0.1) |
| El Salvador | 1375.4 (880.6, 1940.0) | 87.5 (55.7, 123.6) | 1940.6 (1172.5, 2881.4) | 55.9 (34.0, 82.8) | -1.5 (-1.7, -1.2) |
| Equatorial Guinea | 273.2 (166.2, 423.0) | 306.1 (181.0, 478.8) | 524.4 (304.6, 829.2) | 234.7 (133.3, 371.9) | -1.1 (-1.3, -0.9) |
| Eritrea | 783.9 (442.6, 1304.7) | 170.0 (92.9, 282.7) | 1721.8 (980.6, 2730.6) | 146.0 (80.3, 235.0) | -0.5 (-0.6, -0.5) |
| Estonia | 5564.4 (3730.5, 7664.3) | 490.4 (328.0, 676.6) | 1650.4 (1046.1, 2350.8) | 103.3 (67.5, 145.5) | -6.4 (-6.9, -5.9) |
| Eswatini | 359.4 (217.9, 533.8) | 284.0 (167.2, 428.0) | 769.4 (450.9, 1202.6) | 309.0 (176.3, 484.1) | 0.6 (0.2, 1.0) |
| Ethiopia | 9036.4 (5131.9, 14794.1) | 97.8 (54.1, 161.2) | 15137.8 (9194.8, 23428.5) | 72.3 (43.1, 112.9) | -1.2 (-1.3, -1.1) |
| Fiji | 306.0 (199.1, 442.7) | 180.4 (113.4, 265.4) | 589.5 (379.6, 860.1) | 162.6 (100.8, 240.7) | -0.6 (-0.7, -0.4) |
| Finland | 5969.4 (3757.9, 8568.7) | 145.1 (91.2, 208.2) | 3959.0 (2322.3, 5822.9) | 48.2 (29.0, 70.4) | -3.4 (-3.5, -3.4) |
| France | 39928.0 (22571.0, 58944.7) | 79.2 (45.9, 115.9) | 29294.7 (16638.2, 43643.8) | 30.4 (18.2, 44.7) | -3.3 (-3.5, -3.1) |
| Gabon | 759.8 (475.3, 1118.0) | 265.9 (162.4, 396.4) | 1121.3 (680.8, 1676.0) | 236.3 (139.7, 355.9) | -0.5 (-0.6, -0.4) |
| Gambia | 504.4 (309.0, 768.9) | 292.2 (175.1, 448.4) | 1557.6 (915.2, 2404.5) | 322.1 (187.2, 498.5) | 0.3 (0.2, 0.4) |
| Georgia | 9938.0 (6402.0, 13861.1) | 302.3 (192.8, 423.1) | 12685.5 (8233.9, 17583.4) | 371.9 (245.0, 511.4) | 0.6 (0.1, 1.1) |
| Germany | 120487.2 (71040.8, 177416.3) | 160.5 (96.1, 235.1) | 64591.0 (37571.5, 95284.8) | 52.6 (32.3, 76.2) | -3.7 (-4.1, -3.4) |
| Ghana | 8694.5 (5306.7, 13384.8) | 282.9 (168.3, 436.7) | 24381.0 (14434.1, 37293.4) | 294.2 (169.8, 452.6) | 0.4 (0.2, 0.6) |
| Greece | 18627.9 (10851.5, 26860.6) | 222.7 (128.8, 322.2) | 11546.9 (6360.3, 17203.9) | 64.5 (37.5, 94.4) | -4.7 (-5.0, -4.4) |
| Greenland | 36.0 (22.4, 52.1) | 279.6 (163.8, 414.6) | 29.8 (18.3, 45.1) | 99.4 (57.6, 153.8) | -3.6 (-3.7, -3.4) |
| Grenada | 109.8 (70.3, 153.4) | 262.4 (171.3, 362.5) | 76.1 (48.8, 107.3) | 133.9 (83.2, 191.6) | -2.1 (-2.3, -2.0) |
| Guam | 49.2 (32.7, 69.4) | 145.2 (91.9, 209.0) | 88.4 (58.5, 125.1) | 77.3 (50.8, 109.8) | -1.8 (-2.1, -1.6) |
| Guatemala | 1596.1 (1058.4, 2203.6) | 102.0 (65.1, 143.2) | 3615.6 (2317.5, 5038.1) | 63.4 (40.1, 88.7) | -2.1 (-2.4, -1.8) |
| Guinea | 4177.5 (2531.1, 6372.5) | 245.4 (145.5, 376.9) | 8019.6 (4827.2, 12209.5) | 284.6 (168.5, 434.9) | 0.8 (0.6, 0.9) |
| Guinea-Bissau | 819.4 (509.2, 1255.8) | 414.2 (251.8, 636.1) | 1329.0 (818.8, 1989.8) | 390.3 (235.6, 585.2) | -0.1 (-0.1, 0.0) |
| Guyana | 674.3 (463.9, 924.7) | 349.0 (236.1, 482.1) | 629.4 (408.1, 907.8) | 197.2 (124.7, 286.8) | -1.3 (-1.5, -1.1) |
| Haiti | 4589.6 (2771.7, 6844.8) | 304.6 (179.0, 460.1) | 7648.6 (4292.3, 12281.8) | 233.1 (127.8, 374.4) | -0.7 (-0.8, -0.7) |
| Honduras | 1575.8 (978.7, 2322.4) | 152.9 (93.8, 225.4) | 6067.3 (3623.5, 9230.3) | 194.7 (114.3, 297.1) | 1.0 (0.8, 1.2) |
| Hungary | 27470.5 (17975.8, 38043.7) | 341.8 (221.4, 475.5) | 12879.0 (8031.9, 18435.0) | 113.0 (71.5, 160.9) | -4.0 (-4.2, -3.8) |
| Iceland | 143.4 (86.6, 207.4) | 85.3 (52.0, 123.0) | 100.3 (57.2, 149.5) | 27.9 (16.2, 41.4) | -3.7 (-3.9, -3.6) |
| India | 342658.9 (229794.9, 495307.1) | 154.9 (100.1, 225.0) | 767456.0 (503895.8, 1136285.2) | 125.6 (80.5, 186.3) | -0.9 (-1.0, -0.8) |
| Indonesia | 147802.7 (99520.5, 206236.4) | 313.0 (205.0, 443.4) | 444680.7 (282534.4, 651404.1) | 394.4 (245.0, 580.3) | 0.8 (0.7, 1.0) |
| Iran (Islamic Republic of) | 45845.3 (32499.5, 61990.2) | 354.8 (236.5, 495.1) | 74088.1 (49421.4, 101789.3) | 183.7 (119.4, 255.6) | -2.2 (-2.3, -2.1) |
| Iraq | 21061.6 (13354.5, 30519.9) | 501.9 (316.0, 728.0) | 49862.7 (30580.7, 74122.9) | 426.0 (254.6, 638.0) | -1.0 (-1.2, -0.9) |
| Ireland | 4380.7 (2890.0, 5981.8) | 188.4 (122.4, 258.9) | 1975.3 (1249.0, 2781.3) | 42.0 (26.6, 59.1) | -4.9 (-5.1, -4.7) |
| Israel | 2672.1 (1680.9, 3786.8) | 100.2 (62.2, 142.9) | 2472.7 (1470.3, 3596.4) | 33.6 (20.2, 48.7) | -4.0 (-4.2, -3.8) |
| Italy | 71511.2 (43337.3, 102778.1) | 140.9 (85.1, 202.9) | 36641.9 (20350.9, 55669.9) | 34.9 (20.3, 52.1) | -4.6 (-4.9, -4.4) |
| Jamaica | 1619.9 (1034.5, 2287.1) | 157.8 (101.3, 222.3) | 1905.8 (1145.6, 2787.7) | 112.2 (67.7, 164.1) | -0.9 (-1.3, -0.5) |
| Japan | 153585.4 (91577.8, 222192.8) | 170.2 (99.4, 248.5) | 129966.4 (71912.5, 197941.4) | 55.6 (33.1, 82.4) | -3.8 (-4.0, -3.7) |
| Jordan | 2542.5 (1641.6, 3652.0) | 392.4 (246.4, 571.1) | 6644.6 (4178.1, 9705.5) | 185.0 (112.0, 274.6) | -3.0 (-3.3, -2.6) |
| Kazakhstan | 34622.4 (24081.3, 46933.9) | 529.8 (361.0, 725.4) | 34866.4 (23569.3, 47617.0) | 396.3 (260.2, 548.2) | -1.6 (-2.1, -1.1) |
| Kenya | 3893.3 (2382.7, 5880.3) | 99.2 (59.4, 151.3) | 11379.5 (6972.1, 16838.6) | 110.9 (64.9, 168.3) | 0.5 (0.4, 0.6) |
| Kiribati | 38.1 (25.3, 54.8) | 207.9 (132.9, 304.9) | 75.0 (48.2, 109.9) | 213.4 (132.0, 317.3) | 0.1 (0.0, 0.1) |
| Kuwait | 435.4 (289.3, 602.2) | 139.6 (89.4, 195.9) | 1412.8 (898.7, 2055.0) | 92.2 (56.6, 136.2) | -1.1 (-2.1, -0.2) |
| Kyrgyzstan | 7518.9 (5248.3, 10133.5) | 481.8 (332.3, 653.9) | 7333.1 (5124.4, 9804.7) | 286.2 (194.7, 387.6) | -2.2 (-2.6, -1.9) |
| Lao People's Democratic Republic | 5046.3 (3179.0, 7483.7) | 486.8 (299.4, 728.4) | 7234.2 (4491.7, 10712.6) | 324.3 (198.7, 482.1) | -1.4 (-1.5, -1.4) |
| Latvia | 10117.9 (6636.1, 14035.3) | 509.1 (333.8, 705.7) | 7214.9 (4538.8, 10235.3) | 294.7 (191.2, 413.2) | -2.2 (-2.5, -2.0) |
| Lebanon | 2685.2 (1601.3, 4131.0) | 244.6 (142.3, 379.2) | 3313.5 (1983.3, 4992.0) | 95.9 (58.3, 143.6) | -3.1 (-3.4, -2.8) |
| Lesotho | 882.9 (541.5, 1332.0) | 212.4 (126.6, 324.9) | 1824.7 (1096.5, 2847.8) | 361.0 (211.2, 565.7) | 2.5 (2.1, 2.9) |
| Liberia | 1605.0 (1036.3, 2337.4) | 273.6 (172.7, 401.1) | 2589.9 (1570.3, 4024.2) | 261.3 (156.0, 406.2) | -0.2 (-0.3, -0.1) |
| Libya | 1803.8 (1079.1, 2767.5) | 184.6 (109.1, 284.7) | 5847.8 (3394.0, 9130.4) | 214.1 (122.0, 336.6) | 0.9 (0.7, 1.1) |
| Lithuania | 7630.5 (5133.3, 10432.2) | 306.7 (206.9, 418.2) | 6739.8 (4259.7, 9659.1) | 195.7 (127.5, 276.8) | -1.3 (-1.7, -1.0) |
| Luxembourg | 664.2 (409.7, 945.6) | 215.1 (132.5, 306.1) | 240.9 (143.2, 352.6) | 37.1 (22.5, 54.0) | -5.6 (-5.8, -5.4) |
| Madagascar | 5011.3 (3047.0, 7496.6) | 204.2 (120.6, 308.4) | 9453.2 (5606.0, 14495.2) | 190.9 (108.6, 297.2) | -0.3 (-0.4, -0.2) |
| Malawi | 2929.0 (1780.5, 4476.4) | 163.9 (95.9, 254.4) | 6353.3 (3663.5, 9990.8) | 185.1 (103.4, 295.5) | 0.2 (-0.1, 0.4) |
| Malaysia | 13147.3 (8855.6, 18582.4) | 279.2 (186.7, 395.2) | 30890.2 (20313.5, 43602.1) | 209.6 (135.2, 299.3) | -0.7 (-0.8, -0.6) |
| Maldives | 150.5 (101.1, 215.2) | 356.1 (231.1, 517.3) | 242.8 (151.0, 357.3) | 149.3 (92.3, 220.1) | -3.2 (-3.3, -3.1) |
| Mali | 3747.6 (2253.5, 5923.8) | 199.5 (115.8, 317.7) | 7853.3 (4631.5, 12639.9) | 187.5 (107.8, 301.5) | -0.1 (-0.2, 0.1) |
| Malta | 355.4 (226.4, 497.6) | 155.5 (97.4, 219.2) | 220.5 (129.3, 327.0) | 36.1 (21.3, 53.4) | -4.9 (-5.1, -4.7) |
| Marshall Islands | 17.2 (10.7, 25.7) | 219.1 (131.3, 332.1) | 31.8 (19.8, 48.3) | 198.7 (118.3, 304.8) | -0.4 (-0.4, -0.3) |
| Mauritania | 1666.0 (1020.9, 2587.4) | 323.3 (194.4, 505.0) | 2576.2 (1479.6, 4118.9) | 240.2 (135.8, 384.0) | -1.1 (-1.3, -1.0) |
| Mauritius | 1855.0 (1315.9, 2462.2) | 484.6 (336.7, 650.4) | 1761.8 (1228.3, 2357.8) | 178.2 (122.2, 240.8) | -4.3 (-4.9, -3.8) |
| Mexico | 28004.0 (18911.3, 38016.4) | 135.5 (89.2, 185.9) | 43954.4 (29457.6, 60472.1) | 66.0 (43.6, 91.4) | -2.4 (-2.5, -2.2) |
| Micronesia (Federated States of) | 58.9 (36.3, 88.1) | 237.4 (142.8, 358.5) | 74.2 (45.8, 112.3) | 208.9 (124.8, 319.0) | -0.5 (-0.5, -0.4) |
| Monaco | 79.7 (43.6, 122.9) | 173.0 (97.2, 264.0) | 45.5 (25.0, 69.8) | 70.9 (39.9, 108.1) | -3.0 (-3.2, -2.8) |
| Mongolia | 717.4 (470.4, 1012.4) | 129.0 (82.7, 184.4) | 1467.0 (972.4, 2073.6) | 125.1 (79.7, 180.8) | -0.2 (-0.5, 0.1) |
| Montenegro | 315.1 (191.1, 473.9) | 97.7 (58.8, 147.3) | 683.6 (395.6, 1037.9) | 136.4 (77.5, 208.4) | 1.2 (1.2, 1.3) |
| Morocco | 23161.5 (13612.5, 36112.9) | 308.7 (179.4, 483.6) | 51760.2 (29244.7, 80919.1) | 287.3 (159.7, 451.8) | -0.1 (-0.2, -0.1) |
| Mozambique | 5908.7 (3599.9, 8931.2) | 207.6 (122.7, 318.4) | 14309.3 (8101.0, 22709.5) | 269.2 (148.5, 430.4) | 1.3 (1.1, 1.5) |
| Myanmar | 50224.8 (31327.3, 75864.8) | 433.5 (266.2, 653.7) | 71402.5 (43800.6, 106475.3) | 292.7 (176.9, 439.5) | -1.4 (-1.5, -1.3) |
| Namibia | 1016.2 (657.1, 1439.5) | 344.6 (212.5, 496.9) | 1871.3 (1158.7, 2758.4) | 302.3 (182.4, 450.8) | -0.7 (-0.9, -0.4) |
| Nauru | 7.6 (4.7, 11.4) | 340.8 (203.9, 512.0) | 9.5 (5.9, 14.3) | 326.2 (196.6, 491.9) | -0.3 (-0.6, 0.0) |
| Nepal | 10465.8 (6169.3, 16097.3) | 239.3 (136.7, 370.8) | 20515.5 (11908.5, 32848.0) | 175.1 (98.9, 282.3) | -1.2 (-1.4, -1.0) |
| Netherlands | 13885.4 (8522.4, 19898.5) | 119.5 (73.5, 171.1) | 10722.7 (6436.9, 15460.1) | 49.7 (30.1, 71.5) | -3.4 (-3.6, -3.1) |
| New Zealand | 2441.6 (1463.9, 3507.3) | 111.5 (66.0, 161.0) | 2214.4 (1257.5, 3311.4) | 43.7 (25.0, 65.1) | -3.2 (-3.4, -3.1) |
| Nicaragua | 970.0 (628.3, 1382.2) | 126.9 (80.8, 182.1) | 1995.3 (1274.4, 2881.9) | 78.1 (49.4, 113.2) | -1.6 (-1.8, -1.5) |
| Niger | 2429.7 (1434.6, 3992.6) | 193.9 (110.3, 319.7) | 6887.2 (4015.4, 11236.0) | 184.0 (104.3, 300.5) | -0.1 (-0.1, 0.0) |
| Nigeria | 71224.7 (45173.8, 107568.9) | 326.6 (202.1, 496.1) | 108199.6 (70802.8, 153762.4) | 254.1 (162.8, 363.5) | -0.9 (-1.0, -0.8) |
| Niue | 2.7 (1.7, 4.1) | 212.4 (132.8, 315.5) | 2.1 (1.3, 3.0) | 179.1 (111.3, 262.0) | -0.7 (-0.8, -0.7) |
| North Macedonia | 5185.2 (3357.2, 7269.3) | 567.2 (358.1, 805.9) | 8002.3 (4808.3, 11737.7) | 522.5 (298.5, 780.2) | -0.6 (-1.0, -0.2) |
| Northern Mariana Islands | 13.5 (8.8, 19.5) | 178.6 (110.8, 263.2) | 30.9 (20.5, 43.7) | 127.3 (81.3, 184.3) | -1.4 (-1.6, -1.2) |
| Norway | 5398.6 (3313.3, 7740.3) | 127.0 (78.6, 181.4) | 2334.9 (1375.3, 3473.5) | 37.6 (22.4, 55.7) | -4.2 (-4.3, -4.1) |
| Oman | 1007.2 (598.3, 1550.8) | 291.3 (170.3, 450.1) | 1876.6 (1180.2, 2778.1) | 193.4 (117.1, 289.5) | -0.8 (-1.0, -0.6) |
| Pakistan | 45746.1 (27745.0, 70452.4) | 161.9 (96.1, 251.9) | 101537.7 (64874.8, 152882.3) | 175.0 (108.6, 266.6) | -0.1 (-0.2, 0.1) |
| Palau | 12.0 (7.7, 17.5) | 240.7 (150.2, 353.7) | 23.4 (15.2, 33.9) | 214.1 (133.9, 315.4) | -0.2 (-0.3, -0.2) |
| Palestine | 1868.9 (1116.2, 2805.5) | 430.8 (252.4, 651.7) | 3003.7 (1920.6, 4342.6) | 255.3 (157.1, 374.3) | -1.7 (-2.0, -1.5) |
| Panama | 984.8 (652.9, 1370.9) | 128.1 (83.8, 179.4) | 1891.2 (1163.3, 2715.8) | 77.7 (47.8, 111.6) | -1.8 (-2.0, -1.6) |
| Papua New Guinea | 1145.4 (655.3, 1824.8) | 146.4 (80.3, 235.6) | 2880.9 (1712.1, 4537.4) | 132.2 (75.4, 210.0) | -0.4 (-0.5, -0.3) |
| Paraguay | 1790.0 (1149.6, 2545.9) | 159.7 (101.2, 228.6) | 3299.4 (2003.4, 4905.5) | 111.6 (67.0, 166.6) | -1.0 (-1.2, -0.9) |
| Peru | 3536.8 (2162.8, 5309.4) | 57.9 (35.0, 87.3) | 6291.9 (3695.2, 9712.6) | 34.6 (20.3, 53.5) | -2.2 (-2.6, -1.7) |
| Philippines | 29222.6 (20004.8, 39946.2) | 213.4 (141.9, 295.2) | 88522.6 (61240.3, 121064.9) | 211.5 (142.8, 292.8) | 0.1 (0.0, 0.2) |
| Poland | 73521.5 (47839.3, 101619.1) | 316.5 (203.2, 439.6) | 46344.0 (28974.7, 66050.3) | 109.3 (69.1, 155.0) | -3.9 (-4.0, -3.8) |
| Portugal | 22742.6 (14213.2, 32212.4) | 307.4 (187.6, 439.3) | 8001.9 (4628.2, 11912.0) | 47.0 (28.1, 69.0) | -6.4 (-6.6, -6.3) |
| Puerto Rico | 1353.2 (871.1, 1925.4) | 69.7 (44.4, 99.4) | 1170.4 (708.1, 1706.4) | 27.4 (17.1, 39.6) | -3.4 (-3.5, -3.2) |
| Qatar | 117.8 (73.3, 173.8) | 276.8 (162.6, 418.2) | 437.1 (266.3, 665.3) | 108.2 (62.2, 169.0) | -3.4 (-4.1, -2.7) |
| Republic of Korea | 45564.6 (29222.7, 65313.4) | 343.3 (207.0, 504.8) | 36732.2 (20635.2, 55967.1) | 70.8 (39.6, 108.2) | -5.8 (-6.0, -5.5) |
| Republic of Moldova | 6742.0 (4560.0, 9239.7) | 313.7 (204.8, 435.9) | 8549.6 (5923.5, 11505.7) | 255.3 (176.5, 344.4) | -0.4 (-0.9, 0.0) |
| Romania | 49643.9 (31475.3, 69903.0) | 360.8 (221.3, 514.9) | 47174.4 (28440.8, 68318.5) | 209.5 (129.0, 300.5) | -2.4 (-2.7, -2.1) |
| Russian Federation | 539536.2 (362996.2, 736455.8) | 571.9 (377.9, 787.2) | 426899.8 (276689.4, 593051.6) | 318.5 (206.7, 442.6) | -2.7 (-3.3, -2.2) |
| Rwanda | 3170.6 (1874.3, 4927.6) | 238.3 (136.3, 372.7) | 3427.5 (1934.9, 5408.9) | 122.0 (66.3, 195.3) | -3.0 (-3.4, -2.6) |
| Saint Kitts and Nevis | 72.0 (46.5, 101.1) | 334.9 (214.3, 473.7) | 57.2 (37.4, 79.1) | 176.2 (111.0, 248.8) | -2.0 (-2.2, -1.8) |
| Saint Lucia | 118.6 (77.5, 165.6) | 270.6 (172.5, 381.4) | 152.0 (93.4, 219.3) | 116.9 (71.5, 168.9) | -3.2 (-3.6, -2.8) |
| Saint Vincent and the Grenadines | 69.0 (44.5, 97.3) | 178.0 (112.8, 252.4) | 86.8 (55.9, 122.5) | 115.0 (73.2, 163.0) | -1.3 (-1.5, -1.1) |
| Samoa | 75.7 (48.3, 110.3) | 182.6 (113.1, 269.2) | 118.0 (74.7, 171.2) | 162.2 (100.6, 237.8) | -0.4 (-0.5, -0.4) |
| San Marino | 22.3 (12.8, 33.4) | 108.1 (61.8, 162.0) | 18.4 (10.0, 29.3) | 37.6 (21.0, 58.9) | -3.0 (-3.3, -2.8) |
| Sao,me and Principe | 85.3 (55.1, 123.6) | 254.1 (161.7, 369.9) | 162.2 (104.1, 236.9) | 293.9 (186.0, 430.1) | 0.6 (0.4, 0.7) |
| Saudi Arabia | 9949.4 (6010.8, 15049.0) | 342.2 (202.2, 520.5) | 24226.7 (15287.7, 36208.5) | 240.5 (146.5, 362.1) | -1.3 (-1.5, -1.2) |
| Senegal | 4652.0 (2884.2, 7016.4) | 283.8 (172.9, 432.7) | 9219.9 (5522.4, 14391.8) | 240.8 (141.9, 377.1) | -0.6 (-0.7, -0.6) |
| Serbia | 22005.8 (13814.0, 31741.4) | 435.7 (266.2, 636.2) | 23170.2 (14132.1, 34180.9) | 241.8 (147.3, 356.4) | -2.4 (-2.6, -2.2) |
| Seychelles | 89.6 (57.8, 128.2) | 285.9 (183.9, 409.4) | 116.7 (74.5, 167.2) | 194.4 (122.6, 279.5) | -1.0 (-1.1, -0.8) |
| Sierra Leone | 3396.9 (2105.7, 5010.4) | 319.2 (195.5, 472.9) | 5597.1 (3311.8, 8555.6) | 296.5 (173.6, 453.8) | 0.0 (-0.2, 0.2) |
| Singapore | 2799.0 (1864.5, 3858.0) | 255.2 (166.1, 355.0) | 1879.6 (1169.4, 2739.9) | 40.4 (24.9, 59.1) | -6.1 (-6.3, -5.9) |
| Slovakia | 8715.9 (5507.4, 12312.1) | 266.7 (166.7, 377.9) | 6971.8 (4282.9, 10080.2) | 129.4 (79.0, 188.0) | -2.4 (-2.5, -2.3) |
| Slovenia | 2759.6 (1750.6, 3932.1) | 203.5 (129.6, 289.3) | 1568.1 (905.0, 2336.7) | 55.9 (33.1, 82.6) | -4.3 (-4.5, -4.0) |
| Solomon Islands | 120.7 (73.8, 183.9) | 199.1 (116.4, 308.5) | 306.3 (186.6, 476.8) | 192.4 (113.4, 303.5) | -0.1 (-0.2, 0.0) |
| Somalia | 1458.7 (762.1, 2497.2) | 142.9 (72.0, 245.0) | 3114.9 (1686.0, 5213.1) | 118.0 (61.1, 201.0) | -0.6 (-0.7, -0.6) |
| South Africa | 20602.7 (13534.4, 28717.9) | 195.1 (125.7, 274.4) | 52626.2 (36286.5, 72123.0) | 232.6 (156.1, 322.4) | 0.6 (0.1, 1.0) |
| South Sudan | 1789.4 (1024.6, 2849.4) | 139.9 (78.7, 223.7) | 2063.9 (1169.6, 3383.4) | 119.7 (65.8, 198.1) | -0.7 (-0.9, -0.5) |
| Spain | 45695.3 (26889.1, 66450.0) | 148.7 (86.5, 217.1) | 22412.9 (12580.6, 33538.7) | 33.9 (20.0, 49.8) | -4.7 (-5.0, -4.5) |
| Sri Lanka | 17046.6 (11143.0, 23957.0) | 345.2 (220.7, 489.3) | 33152.0 (18892.5, 50413.0) | 238.0 (134.1, 363.3) | -0.7 (-0.9, -0.5) |
| Sudan | 17648.4 (10211.6, 27318.1) | 364.1 (207.0, 565.5) | 29424.7 (16588.5, 47078.7) | 293.7 (163.8, 469.3) | -0.8 (-0.9, -0.8) |
| Suriname | 198.6 (128.3, 287.9) | 154.8 (98.4, 225.8) | 391.4 (233.3, 591.5) | 117.3 (69.0, 178.2) | -0.9 (-1.2, -0.6) |
| Sweden | 11228.9 (6845.5, 16293.4) | 118.7 (73.1, 171.5) | 6223.0 (3592.7, 9363.3) | 44.3 (25.9, 66.6) | -3.3 (-3.4, -3.2) |
| Switzerland | 6855.0 (4051.3, 10028.5) | 105.8 (63.6, 153.7) | 3788.5 (2162.0, 5613.6) | 32.0 (18.8, 47.0) | -3.8 (-3.9, -3.7) |
| Syrian Arab Republic | 7868.4 (4944.5, 11681.7) | 290.5 (178.6, 435.0) | 14238.8 (8538.6, 21666.7) | 216.2 (125.6, 332.5) | -1.5 (-1.7, -1.2) |
| Taiwan (Province of China) | 15007.4 (10477.2, 20286.8) | 194.3 (129.7, 267.9) | 13342.4 (8441.9, 19023.5) | 56.9 (36.0, 81.2) | -3.8 (-4.0, -3.6) |
| Tajikistan | 5096.2 (3383.2, 7254.2) | 358.2 (235.4, 512.0) | 7743.6 (5034.9, 11072.3) | 290.7 (182.1, 422.0) | -0.9 (-1.3, -0.6) |
| Thailand | 36962.9 (24220.8, 53044.0) | 210.5 (135.0, 304.0) | 85416.8 (54951.7, 122197.3) | 143.2 (91.8, 205.4) | -1.8 (-2.0, -1.6) |
| Timor-Leste | 302.2 (194.3, 439.3) | 251.6 (157.0, 368.0) | 1229.1 (721.7, 1906.9) | 279.4 (161.3, 434.4) | 0.6 (0.4, 0.7) |
| Togo | 1767.7 (1133.9, 2591.1) | 297.3 (186.0, 439.5) | 5226.1 (3137.6, 7892.6) | 289.9 (169.9, 440.8) | -0.2 (-0.4, -0.1) |
| Tokelau | 1.5 (0.9, 2.2) | 208.2 (124.5, 310.6) | 1.3 (0.8, 1.9) | 153.7 (93.8, 233.9) | -1.1 (-1.1, -1.0) |
| Tonga | 32.6 (20.6, 47.1) | 118.0 (72.8, 172.3) | 47.9 (29.3, 71.8) | 112.2 (68.2, 168.5) | -0.1 (-0.1, 0.0) |
| Trinidad and,bago | 939.8 (629.2, 1301.6) | 218.0 (143.1, 304.8) | 1189.6 (729.1, 1737.8) | 113.6 (68.7, 166.6) | -2.5 (-2.7, -2.2) |
| Tunisia | 5940.3 (3594.9, 9076.0) | 240.8 (141.0, 373.4) | 12793.3 (7262.8, 20334.1) | 183.3 (102.6, 292.8) | -1.1 (-1.2, -1.0) |
| Turkey | 53622.4 (34269.9, 77946.6) | 311.9 (195.8, 457.1) | 72316.6 (43291.5, 108098.8) | 149.0 (87.8, 224.3) | -2.4 (-2.6, -2.2) |
| Turkmenistan | 3997.4 (2767.6, 5375.8) | 394.7 (268.1, 535.6) | 9772.9 (6463.5, 13605.1) | 467.8 (303.3, 655.9) | 0.3 (-0.1, 0.8) |
| Tuvalu | 8.0 (5.2, 11.8) | 248.9 (152.9, 370.9) | 10.8 (6.7, 15.9) | 203.3 (123.1, 301.9) | -0.7 (-0.7, -0.6) |
| Uganda | 4487.0 (2658.5, 6963.6) | 146.8 (84.5, 230.7) | 7652.3 (4464.5, 12019.6) | 113.7 (64.4, 180.6) | -1.4 (-1.7, -1.2) |
| Ukraine | 204688.4 (136485.7, 280100.6) | 531.1 (349.3, 730.2) | 131878.1 (80041.2, 191626.9) | 300.9 (183.5, 436.5) | -2.5 (-2.8, -2.2) |
| United Arab Emirates | 784.1 (485.5, 1174.2) | 370.0 (224.0, 557.9) | 3218.2 (2105.6, 4614.3) | 212.8 (126.9, 317.9) | -0.4 (-0.9, 0.0) |
| United Kingdom | 87568.7 (52056.2, 125733.4) | 159.7 (95.9, 228.5) | 33590.6 (19534.6, 49095.2) | 40.5 (24.2, 58.6) | -4.7 (-4.9, -4.5) |
| United Republic of Tanzania | 5574.5 (3310.2, 8691.6) | 106.6 (61.1, 168.9) | 16872.5 (9461.3, 26963.5) | 139.8 (76.8, 224.6) | 1.0 (0.8, 1.1) |
| United States of America | 176309.2 (107362.5, 253317.3) | 94.7 (58.0, 135.9) | 218268.7 (133389.6, 314719.2) | 64.6 (39.8, 93.0) | -1.7 (-2.0, -1.5) |
| United States Virgin Islands | 40.6 (24.4, 61.1) | 101.8 (59.7, 154.3) | 50.0 (29.9, 76.6) | 48.3 (28.7, 74.2) | -2.3 (-2.5, -2.2) |
| Uruguay | 3866.1 (2359.4, 5595.3) | 175.9 (106.7, 255.2) | 2655.3 (1567.4, 3868.3) | 78.0 (47.5, 112.3) | -2.8 (-3.0, -2.7) |
| Uzbekistan | 20171.6 (14464.4, 26495.0) | 325.4 (231.8, 428.4) | 40144.5 (28190.2, 54175.6) | 302.3 (205.9, 414.3) | -0.8 (-1.1, -0.5) |
| Vanuatu | 70.5 (43.9, 104.7) | 241.5 (144.3, 362.4) | 175.1 (113.1, 258.3) | 210.5 (131.0, 313.6) | -0.6 (-0.6, -0.5) |
| Venezuela (Bolivarian Republic of) | 5868.4 (3875.9, 8137.9) | 121.9 (79.3, 169.8) | 14416.2 (8762.0, 21106.5) | 94.4 (56.5, 138.9) | -1.2 (-1.4, -0.9) |
| Viet Nam | 59732.6 (36215.8, 90115.6) | 289.6 (173.5, 438.9) | 147741.7 (88035.9, 226329.9) | 298.7 (176.1, 458.9) | 0.6 (0.4, 0.8) |
| Yemen | 9351.3 (5394.8, 14897.5) | 383.4 (216.4, 614.5) | 24999.5 (14040.0, 40318.5) | 354.9 (195.1, 574.9) | -0.4 (-0.5, -0.4) |
| Zambia | 1822.7 (1066.7, 2928.2) | 140.0 (79.4, 227.6) | 5145.3 (2987.9, 8114.0) | 168.9 (95.5, 269.3) | 0.5 (0.4, 0.6) |
| Zimbabwe | 3682.1 (2377.1, 5390.0) | 199.1 (123.8, 297.6) | 9188.3 (5883.7, 13408.0) | 295.4 (182.3, 434.2) | 1.8 (1.3, 2.3) |

**Notes:** ASR: age-standardized rates; DALYs: disability-adjusted life years; EAPCs: estimated annual percentage changes.

**Table S2. Global, regional, and national/territorial trends in intracerebral hemorrhage attributable kidney dysfunction burden: mortality, and DALYs (1990–2021).**

| **Location** | **1990** | | **2021** | | **EAPC 95%CI** |
| --- | --- | --- | --- | --- | --- |
|  | **Number** | **ASR, per**  **100,000 persons** | **Number** | **ASR, per**  **100,000 persons** |  |
| **Deaths** |  |  |  |  |  |
| Global | 240096.8 (166883.0, 322027.6) | 11.4 (7.7, 15.4) | 324946.2 (220387.6, 443171.6) | 6.9 (4.6, 9.4) | -1.7 (-1.8, -1.5) |
| High SDI | 21322.2 (14087.6, 29258.6) | 3.5 (2.3, 4.7) | 20061.4 (12450.7, 28511.4) | 1.6 (1.1, 2.3) | -2.6 (-2.7, -2.5) |
| High-middle SDI | 59824.2 (40912.0, 81632.8) | 11.5 (7.7, 15.8) | 60234.8 (39122.3, 85043.2) | 5.5 (3.6, 7.8) | -2.6 (-2.9, -2.3) |
| Middle SDI | 90660.9 (62840.2, 123906.3) | 17.6 (11.7, 24.6) | 135160.5 (90549.5, 187320.3) | 9.5 (6.2, 13.3) | -1.9 (-2.1, -1.7) |
| Low-middle SDI | 48260.0 (33832.3, 64206.2) | 15.1 (10.3, 20.3) | 80069.4 (55307.6, 108543.1) | 10.4 (7.1, 14.3) | -1.3 (-1.3, -1.2) |
| Low SDI | 19797.0 (13621.3, 26701.7) | 16.7 (11.2, 23.0) | 29162.1 (19853.2, 40301.4) | 11.1 (7.3, 15.5) | -1.5 (-1.5, -1.4) |
| High-income Asia Pacific | 6271.3 (4021.7, 8820.3) | 5.8 (3.7, 8.3) | 5072.3 (2875.5, 7573.7) | 1.8 (1.1, 2.6) | -3.9 (-4.1, -3.7) |
| High-income North America | 3806.5 (2495.6, 5255.6) | 1.9 (1.3, 2.6) | 6408.5 (4133.4, 8966.5) | 1.7 (1.1, 2.4) | -0.5 (-0.8, -0.3) |
| Western Europe | 9157.7 (5913.0, 12704.9) | 2.7 (1.8, 3.8) | 6859.8 (4088.5, 10016.5) | 1.1 (0.7, 1.6) | -3.0 (-3.1, -2.9) |
| Australasia | 276.7 (175.9, 388.9) | 2.1 (1.3, 3.0) | 310.6 (177.3, 463.5) | 0.9 (0.6, 1.4) | -2.7 (-2.7, -2.6) |
| Andean Latin America | 518.7 (345.2, 727.9) | 4.7 (3.1, 6.6) | 665.3 (427.7, 961.1) | 2.1 (1.3, 3.0) | -2.9 (-3.1, -2.7) |
| Tropical Latin America | 4567.5 (3380.1, 5849.8) | 9.0 (6.5, 11.7) | 4449.8 (3220.7, 5789.8) | 3.1 (2.3, 4.1) | -3.6 (-3.6, -3.5) |
| Central Latin America | 2417.3 (1717.7, 3205.2) | 5.6 (3.9, 7.5) | 4030.7 (2779.9, 5384.2) | 3.0 (2.0, 4.0) | -2.5 (-2.7, -2.4) |
| Southern Latin America | 1356.4 (907.2, 1874.9) | 5.4 (3.5, 7.4) | 961.9 (625.0, 1360.3) | 2.0 (1.3, 2.8) | -3.1 (-3.3, -3.0) |
| Caribbean | 1091.5 (755.9, 1465.4) | 7.7 (5.3, 10.3) | 1507.5 (1009.2, 2118.6) | 5.1 (3.4, 7.2) | -1.4 (-1.5, -1.2) |
| Central Europe | 6495.5 (4430.9, 8766.4) | 8.1 (5.4, 11.0) | 3844.3 (2470.1, 5357.8) | 3.0 (2.0, 4.2) | -3.9 (-4.2, -3.6) |
| Eastern Europe | 11015.3 (7650.0, 14849.9) | 7.3 (5.0, 9.9) | 8532.8 (5774.2, 11595.3) | 4.5 (3.0, 6.1) | -2.5 (-3.0, -1.9) |
| Central Asia | 4183.8 (2985.6, 5517.3) | 17.0 (11.9, 22.6) | 4576.2 (3228.5, 6073.2) | 11.1 (7.6, 15.0) | -1.8 (-2.2, -1.4) |
| North Africa and Middle East | 9354.4 (6291.9, 12900.9) | 10.8 (7.0, 15.1) | 11279.3 (7404.1, 15738.1) | 4.8 (3.0, 6.7) | -2.8 (-2.9, -2.7) |
| South Asia | 35555.5 (24155.9, 48480.0) | 11.8 (7.8, 16.4) | 63663.7 (42601.3, 88721.2) | 8.1 (5.3, 11.4) | -1.3 (-1.4, -1.2) |
| Southeast Asia | 34393.3 (24146.0, 46017.1) | 25.7 (17.6, 34.8) | 65856.7 (45090.0, 89786.6) | 18.6 (12.5, 25.7) | -1.0 (-1.2, -0.9) |
| East Asia | 89298.2 (59685.9, 126544.1) | 22.4 (14.1, 32.6) | 106061.0 (66871.6, 154721.8) | 9.3 (5.7, 13.7) | -2.6 (-3.0, -2.3) |
| Oceania | 374.6 (249.8, 538.5) | 25.6 (16.5, 37.1) | 761.8 (501.5, 1101.7) | 20.3 (13.0, 29.7) | -0.8 (-0.8, -0.7) |
| Western Sub-Saharan Africa | 8773.2 (5872.4, 12175.9) | 19.2 (12.5, 27.2) | 12103.3 (8097.6, 16745.9) | 11.9 (7.7, 16.7) | -1.7 (-1.7, -1.6) |
| Eastern Sub-Saharan Africa | 6934.5 (4602.1, 9715.6) | 17.8 (11.5, 25.3) | 9460.5 (6071.8, 13507.8) | 10.9 (6.8, 15.8) | -1.8 (-1.8, -1.7) |
| Central Sub-Saharan Africa | 2614.4 (1689.1, 3703.0) | 23.0 (14.3, 33.2) | 4849.0 (3041.9, 7227.3) | 18.1 (11.0, 27.4) | -0.9 (-1.0, -0.9) |
| Southern Sub-Saharan Africa | 1640.7 (1146.1, 2228.5) | 11.3 (7.7, 15.5) | 3691.2 (2660.0, 4886.5) | 12.1 (8.5, 16.2) | 0.3 (-0.2, 0.8) |
| Afghanistan | 911.0 (505.6, 1441.5) | 24.6 (13.3, 39.4) | 686.0 (371.8, 1097.9) | 13.3 (7.0, 21.6) | -2.4 (-2.7, -2.1) |
| Albania | 186.6 (116.2, 269.8) | 20.1 (12.0, 29.6) | 318.0 (170.4, 492.8) | 13.8 (7.3, 21.4) | -1.2 (-1.4, -1.0) |
| Algeria | 472.6 (262.7, 757.3) | 8.5 (4.5, 14.0) | 712.7 (371.0, 1198.7) | 4.3 (2.1, 7.2) | -2.2 (-2.2, -2.1) |
| American Samoa | 2.1 (1.4, 3.0) | 18.6 (12.2, 26.4) | 3.6 (2.4, 5.2) | 14.4 (9.1, 21.0) | -0.9 (-1.1, -0.8) |
| Andorra | 0.4 (0.2, 0.7) | 1.3 (0.7, 2.2) | 0.7 (0.3, 1.2) | 0.8 (0.4, 1.3) | -1.5 (-1.7, -1.2) |
| Angola | 514.1 (325.9, 744.7) | 26.5 (16.2, 39.0) | 941.2 (574.7, 1403.3) | 16.3 (9.6, 24.7) | -1.9 (-2.0, -1.8) |
| Antigua and Barbuda | 2.9 (1.9, 3.9) | 9.6 (6.6, 12.9) | 3.0 (2.0, 4.0) | 5.1 (3.5, 7.0) | -2.5 (-2.8, -2.2) |
| Argentina | 996.9 (662.2, 1384.2) | 5.6 (3.7, 7.9) | 672.6 (438.0, 952.6) | 2.2 (1.4, 3.0) | -3.0 (-3.2, -2.9) |
| Armenia | 122.4 (84.3, 165.0) | 8.9 (6.0, 12.1) | 64.1 (43.3, 87.1) | 2.7 (1.8, 3.6) | -4.8 (-5.5, -4.1) |
| Australia | 230.8 (145.9, 326.3) | 2.1 (1.3, 3.0) | 258.7 (145.9, 388.8) | 0.9 (0.5, 1.4) | -2.7 (-2.8, -2.6) |
| Austria | 178.1 (111.5, 251.7) | 2.6 (1.6, 3.6) | 102.4 (59.0, 149.6) | 0.9 (0.5, 1.3) | -3.8 (-4.2, -3.5) |
| Azerbaijan | 458.1 (310.3, 637.7) | 18.1 (12.0, 25.5) | 655.5 (409.2, 955.4) | 14.0 (8.5, 20.6) | -0.7 (-1.0, -0.4) |
| Bahamas | 6.2 (4.3, 8.4) | 7.4 (5.1, 10.1) | 8.9 (5.8, 12.6) | 4.0 (2.6, 5.7) | -2.2 (-2.3, -2.1) |
| Bahrain | 8.2 (5.3, 11.6) | 10.8 (6.6, 15.6) | 14.4 (8.7, 21.8) | 4.0 (2.3, 6.2) | -4.1 (-4.6, -3.6) |
| Bangladesh | 4981.1 (3117.8, 7301.0) | 20.3 (12.5, 30.0) | 8835.9 (5155.8, 13674.1) | 12.3 (7.0, 19.2) | -1.5 (-1.9, -1.2) |
| Barbados | 9.5 (6.2, 13.3) | 5.9 (3.9, 8.1) | 10.4 (6.5, 15.0) | 3.6 (2.2, 5.2) | -1.9 (-2.2, -1.7) |
| Belarus | 374.0 (254.1, 528.4) | 5.2 (3.5, 7.4) | 349.3 (222.5, 500.9) | 4.0 (2.6, 5.7) | -1.7 (-2.2, -1.2) |
| Belgium | 271.3 (170.7, 382.2) | 3.1 (1.9, 4.3) | 192.1 (113.3, 281.3) | 1.3 (0.8, 1.9) | -2.8 (-2.9, -2.7) |
| Belize | 3.2 (2.2, 4.3) | 6.2 (4.3, 8.5) | 6.8 (4.7, 9.2) | 4.2 (2.9, 5.8) | -1.8 (-2.3, -1.3) |
| Benin | 209.0 (134.2, 304.0) | 20.0 (12.6, 29.4) | 361.3 (222.3, 533.4) | 13.9 (8.4, 20.8) | -1.2 (-1.3, -1.1) |
| Bermuda | 1.1 (0.7, 1.5) | 3.2 (2.1, 4.5) | 0.9 (0.6, 1.4) | 1.2 (0.8, 1.7) | -3.4 (-3.5, -3.3) |
| Bhutan | 13.3 (7.9, 20.9) | 10.9 (6.2, 17.5) | 19.2 (11.3, 30.2) | 6.0 (3.5, 9.4) | -2.1 (-2.2, -1.9) |
| Bolivia (Plurinational State of) | 145.6 (85.1, 229.8) | 8.5 (4.9, 13.5) | 193.1 (108.0, 311.4) | 4.0 (2.2, 6.4) | -2.5 (-2.6, -2.4) |
| Bosnia and Herzegovina | 96.7 (60.8, 140.7) | 4.7 (2.9, 7.0) | 85.7 (47.5, 132.3) | 2.4 (1.4, 3.8) | -2.5 (-2.8, -2.2) |
| Botswana | 64.6 (40.2, 94.9) | 22.8 (13.8, 34.0) | 81.6 (50.3, 123.7) | 11.2 (6.8, 17.1) | -2.2 (-2.5, -2.0) |
| Brazil | 4471.4 (3312.6, 5727.7) | 9.0 (6.6, 11.7) | 4302.7 (3115.6, 5600.0) | 3.1 (2.2, 4.1) | -3.6 (-3.7, -3.5) |
| Brunei Darussalam | 6.0 (3.7, 8.9) | 11.6 (7.0, 17.3) | 8.7 (5.4, 12.8) | 5.1 (3.0, 7.6) | -2.6 (-2.8, -2.4) |
| Bulgaria | 1050.2 (715.3, 1414.7) | 17.4 (11.2, 24.0) | 451.7 (287.3, 635.6) | 5.6 (3.6, 7.9) | -4.7 (-5.2, -4.2) |
| Burkina Faso | 296.0 (181.6, 437.0) | 13.1 (7.8, 19.7) | 526.8 (310.6, 806.1) | 11.0 (6.3, 17.0) | -0.3 (-0.4, -0.2) |
| Burundi | 325.4 (187.9, 493.6) | 25.9 (14.8, 39.6) | 322.1 (187.9, 492.7) | 12.8 (7.3, 19.9) | -2.8 (-3.1, -2.5) |
| Cabo Verde | 10.8 (6.6, 16.3) | 8.6 (5.3, 12.9) | 17.1 (10.2, 26.5) | 7.2 (4.3, 11.2) | -0.9 (-1.1, -0.6) |
| Cambodia | 770.1 (512.5, 1089.4) | 33.3 (21.7, 47.8) | 1399.6 (874.5, 2081.3) | 22.5 (13.7, 33.7) | -1.4 (-1.5, -1.3) |
| Cameroon | 475.1 (292.9, 709.0) | 20.6 (12.3, 31.3) | 1195.0 (694.7, 1865.9) | 18.2 (10.4, 28.5) | -0.3 (-0.7, 0.0) |
| Canada | 288.7 (183.7, 410.6) | 1.6 (1.0, 2.3) | 365.8 (215.2, 541.2) | 0.8 (0.5, 1.2) | -2.1 (-2.2, -2.0) |
| Central African Republic | 197.8 (119.4, 300.2) | 33.6 (19.7, 51.6) | 301.6 (174.4, 474.7) | 27.2 (15.2, 43.1) | -0.8 (-0.8, -0.7) |
| Chad | 242.0 (151.3, 356.0) | 16.0 (9.9, 23.8) | 473.3 (286.4, 721.7) | 15.6 (9.2, 24.1) | -0.2 (-0.4, 0.0) |
| Chile | 259.5 (173.5, 360.2) | 4.8 (3.1, 6.7) | 225.9 (141.9, 325.1) | 1.6 (1.0, 2.3) | -3.4 (-3.5, -3.2) |
| China | 86599.4 (57748.0, 123205.4) | 22.7 (14.2, 33.1) | 102353.6 (64131.5, 149801.2) | 9.3 (5.7, 13.8) | -2.6 (-3.0, -2.3) |
| Colombia | 579.3 (404.7, 777.0) | 6.3 (4.3, 8.6) | 562.5 (362.6, 803.3) | 1.9 (1.2, 2.7) | -4.8 (-5.0, -4.6) |
| Comoros | 17.7 (10.4, 26.5) | 17.1 (9.9, 25.8) | 23.8 (13.8, 37.5) | 9.4 (5.3, 14.9) | -2.3 (-2.5, -2.0) |
| Congo | 166.5 (102.7, 246.1) | 30.1 (18.0, 45.2) | 236.2 (141.6, 357.6) | 17.4 (10.3, 26.4) | -2.1 (-2.2, -1.9) |
| Cook Islands | 0.9 (0.6, 1.4) | 14.3 (8.9, 21.0) | 1.0 (0.6, 1.4) | 6.7 (4.1, 10.1) | -2.5 (-2.7, -2.3) |
| Costa Rica | 38.8 (26.8, 52.4) | 4.2 (2.9, 5.6) | 58.2 (39.0, 80.1) | 1.9 (1.3, 2.7) | -3.0 (-3.4, -2.7) |
| Coted'Ivoire | 322.4 (200.0, 484.5) | 15.4 (9.3, 23.6) | 723.8 (429.7, 1111.3) | 12.3 (7.1, 18.9) | -0.8 (-0.9, -0.6) |
| Croatia | 231.9 (159.6, 315.0) | 7.2 (4.8, 9.8) | 110.2 (68.4, 159.4) | 2.1 (1.3, 3.0) | -4.4 (-4.7, -4.2) |
| Cuba | 255.3 (171.6, 353.7) | 4.5 (3.0, 6.3) | 283.9 (184.3, 401.5) | 2.6 (1.7, 3.7) | -2.3 (-2.5, -2.0) |
| Cyprus | 23.7 (13.4, 35.8) | 6.8 (3.6, 10.7) | 19.0 (10.6, 30.2) | 1.7 (1.0, 2.8) | -4.7 (-5.0, -4.5) |
| Czechia | 359.5 (242.6, 492.7) | 4.6 (3.1, 6.4) | 136.9 (83.7, 197.8) | 1.1 (0.7, 1.6) | -4.5 (-4.6, -4.3) |
| Democratic People's Republic of Korea | 1653.5 (1014.0, 2415.3) | 21.2 (12.6, 31.6) | 3151.0 (1930.6, 4670.0) | 17.9 (10.8, 26.8) | -0.5 (-0.7, -0.3) |
| Democratic Republic of the Congo | 1639.2 (980.4, 2450.7) | 20.9 (12.1, 31.8) | 3272.2 (1914.9, 5115.5) | 18.4 (10.5, 29.2) | -0.5 (-0.6, -0.4) |
| Denmark | 142.0 (89.3, 201.1) | 3.0 (1.9, 4.2) | 99.9 (60.2, 144.2) | 1.4 (0.8, 2.0) | -2.8 (-3.0, -2.6) |
| Djibouti | 10.0 (5.8, 15.8) | 14.7 (8.4, 23.4) | 31.0 (17.5, 51.0) | 9.6 (5.3, 16.0) | -1.5 (-1.6, -1.5) |
| Dominica | 2.9 (1.9, 4.2) | 8.9 (5.8, 12.7) | 2.8 (1.7, 4.1) | 6.0 (3.7, 8.9) | -1.3 (-1.5, -1.1) |
| Dominican Republic | 134.4 (87.2, 190.9) | 6.9 (4.4, 9.9) | 307.0 (189.4, 468.7) | 5.6 (3.4, 8.5) | -0.2 (-0.4, 0.0) |
| Ecuador | 125.9 (86.8, 172.2) | 4.3 (2.9, 5.9) | 174.0 (108.0, 260.9) | 2.0 (1.2, 2.9) | -2.7 (-2.9, -2.5) |
| Egypt | 1837.7 (938.6, 2954.1) | 14.2 (6.8, 23.7) | 1885.4 (1011.7, 3033.1) | 6.0 (3.1, 9.9) | -2.7 (-2.8, -2.6) |
| El Salvador | 94.1 (61.6, 132.6) | 5.8 (3.8, 8.2) | 93.5 (56.8, 140.6) | 2.7 (1.7, 4.1) | -2.5 (-2.8, -2.2) |
| Equatorial Guinea | 31.0 (19.0, 46.2) | 30.9 (18.5, 46.6) | 28.0 (14.7, 47.0) | 11.1 (5.8, 18.5) | -3.9 (-4.3, -3.5) |
| Eritrea | 144.4 (89.2, 221.2) | 24.7 (14.7, 38.7) | 201.9 (117.7, 319.4) | 14.6 (8.3, 23.2) | -1.7 (-1.8, -1.6) |
| Estonia | 69.9 (46.8, 95.7) | 6.2 (4.1, 8.5) | 21.8 (13.7, 31.1) | 1.4 (0.9, 2.0) | -6.2 (-6.6, -5.7) |
| Eswatini | 33.9 (21.4, 49.3) | 23.7 (14.6, 35.1) | 59.0 (33.4, 95.6) | 20.4 (11.3, 32.9) | 0.0 (-0.6, 0.5) |
| Ethiopia | 1963.6 (1272.5, 2988.7) | 18.9 (11.8, 29.1) | 1675.1 (1033.4, 2499.3) | 7.5 (4.6, 11.4) | -3.4 (-3.6, -3.3) |
| Fiji | 37.8 (24.9, 54.6) | 20.9 (13.3, 30.6) | 60.1 (37.8, 88.8) | 15.6 (9.6, 23.4) | -1.1 (-1.3, -1.0) |
| Finland | 87.4 (54.6, 126.0) | 2.1 (1.3, 3.1) | 85.6 (50.6, 126.5) | 1.1 (0.7, 1.6) | -2.1 (-2.2, -2.0) |
| France | 973.4 (591.2, 1397.3) | 2.0 (1.3, 2.9) | 809.0 (448.9, 1219.0) | 0.8 (0.5, 1.2) | -3.2 (-3.4, -3.0) |
| Gabon | 65.7 (39.8, 98.3) | 21.7 (12.9, 32.9) | 69.9 (41.2, 109.6) | 13.2 (7.6, 20.7) | -1.7 (-1.8, -1.6) |
| Gambia | 29.0 (17.6, 44.0) | 15.9 (9.4, 24.4) | 78.4 (46.9, 121.6) | 15.4 (9.1, 24.1) | -0.2 (-0.3, 0.0) |
| Georgia | 832.8 (569.1, 1136.6) | 24.8 (16.7, 34.1) | 417.1 (273.6, 576.8) | 12.4 (8.2, 17.0) | -2.7 (-3.5, -1.9) |
| Germany | 1715.9 (1050.6, 2534.0) | 2.4 (1.5, 3.4) | 1214.9 (702.7, 1808.7) | 1.0 (0.6, 1.4) | -3.0 (-3.2, -2.7) |
| Ghana | 541.9 (332.8, 812.5) | 16.2 (9.6, 24.7) | 1128.8 (670.8, 1758.9) | 12.7 (7.3, 20.2) | -0.4 (-0.7, -0.2) |
| Greece | 635.8 (398.8, 897.3) | 7.6 (4.7, 10.7) | 461.9 (268.4, 680.9) | 2.8 (1.8, 4.1) | -3.7 (-4.0, -3.4) |
| Greenland | 1.5 (0.9, 2.1) | 9.4 (5.6, 14.0) | 1.5 (0.9, 2.2) | 4.3 (2.5, 6.6) | -2.5 (-2.6, -2.4) |
| Grenada | 4.2 (2.8, 5.7) | 10.7 (7.3, 14.5) | 3.5 (2.3, 4.8) | 5.6 (3.7, 7.8) | -2.3 (-2.5, -2.2) |
| Guam | 3.1 (2.1, 4.3) | 8.3 (5.4, 11.9) | 5.1 (3.4, 7.2) | 4.5 (3.0, 6.3) | -1.7 (-2.1, -1.3) |
| Guatemala | 122.3 (87.3, 163.4) | 6.8 (4.7, 9.3) | 273.5 (181.3, 377.1) | 4.6 (3.0, 6.4) | -2.0 (-2.3, -1.7) |
| Guinea | 304.4 (191.5, 450.5) | 17.1 (10.5, 25.7) | 447.6 (271.5, 678.2) | 15.1 (9.0, 22.9) | -0.2 (-0.3, 0.0) |
| Guinea-Bissau | 64.4 (39.8, 96.8) | 29.9 (18.1, 45.4) | 88.5 (54.3, 133.7) | 23.2 (13.8, 35.5) | -0.7 (-0.8, -0.6) |
| Guyana | 51.5 (36.7, 69.5) | 25.0 (17.6, 33.9) | 36.8 (23.9, 53.0) | 10.4 (6.6, 15.0) | -2.5 (-2.8, -2.3) |
| Haiti | 396.2 (249.1, 568.5) | 23.0 (14.0, 33.6) | 565.2 (331.0, 885.0) | 14.9 (8.5, 23.5) | -1.3 (-1.3, -1.2) |
| Honduras | 103.8 (66.5, 151.5) | 9.5 (6.0, 14.0) | 378.8 (233.7, 569.2) | 11.5 (7.0, 17.4) | 0.8 (0.6, 1.0) |
| Hungary | 585.3 (382.2, 811.8) | 7.3 (4.8, 10.2) | 167.6 (104.8, 240.2) | 1.5 (1.0, 2.2) | -5.4 (-5.7, -5.2) |
| Iceland | 2.9 (1.8, 4.2) | 1.8 (1.1, 2.5) | 2.5 (1.4, 3.7) | 0.7 (0.4, 1.0) | -3.1 (-3.2, -3.0) |
| India | 26858.7 (18222.0, 36821.5) | 10.9 (7.2, 15.3) | 48013.0 (31706.7, 67032.6) | 7.4 (4.8, 10.5) | -1.3 (-1.4, -1.2) |
| Indonesia | 15227.6 (10565.9, 20880.8) | 29.4 (19.8, 41.3) | 33264.8 (21771.3, 46921.9) | 26.6 (17.0, 38.3) | -0.2 (-0.4, -0.1) |
| Iran (Islamic Republic of) | 545.4 (379.6, 735.3) | 4.1 (2.7, 5.7) | 740.0 (495.8, 1021.3) | 1.8 (1.2, 2.5) | -2.6 (-2.7, -2.5) |
| Iraq | 614.1 (385.9, 904.1) | 14.1 (8.8, 20.8) | 1113.1 (654.4, 1707.6) | 8.9 (5.1, 13.7) | -2.2 (-2.4, -2.0) |
| Ireland | 68.4 (46.0, 93.5) | 3.0 (2.0, 4.1) | 46.2 (29.4, 65.3) | 1.0 (0.6, 1.4) | -3.2 (-3.4, -3.0) |
| Israel | 105.8 (69.1, 147.3) | 3.9 (2.5, 5.5) | 92.7 (56.3, 133.7) | 1.3 (0.8, 1.8) | -4.1 (-4.4, -3.9) |
| Italy | 1400.3 (923.8, 1926.1) | 2.8 (1.8, 3.8) | 1190.8 (682.9, 1772.8) | 1.2 (0.7, 1.8) | -2.5 (-2.6, -2.4) |
| Jamaica | 75.7 (52.3, 104.0) | 7.7 (5.4, 10.5) | 97.3 (61.1, 141.1) | 5.8 (3.6, 8.4) | -0.7 (-1.2, -0.1) |
| Japan | 3822.3 (2401.3, 5409.4) | 4.2 (2.6, 6.0) | 4079.4 (2275.2, 6089.7) | 1.8 (1.1, 2.6) | -2.6 (-2.7, -2.4) |
| Jordan | 49.6 (31.0, 73.6) | 7.2 (4.4, 10.8) | 94.4 (56.1, 144.3) | 2.6 (1.5, 4.0) | -3.9 (-4.3, -3.6) |
| Kazakhstan | 868.1 (603.6, 1183.6) | 13.0 (8.9, 18.0) | 1108.7 (748.3, 1520.0) | 12.6 (8.2, 17.4) | -0.8 (-1.1, -0.4) |
| Kenya | 380.5 (238.8, 572.2) | 9.2 (5.6, 14.0) | 983.4 (607.4, 1468.7) | 8.7 (5.2, 13.2) | 0.1 (-0.1, 0.3) |
| Kiribati | 5.5 (3.6, 7.8) | 27.9 (17.9, 40.1) | 9.4 (6.1, 13.9) | 24.2 (15.3, 36.3) | -0.5 (-0.5, -0.4) |
| Kuwait | 7.7 (5.1, 10.7) | 2.3 (1.5, 3.3) | 20.4 (12.5, 30.4) | 1.4 (0.8, 2.0) | -1.7 (-2.5, -0.9) |
| Kyrgyzstan | 291.2 (204.5, 388.1) | 18.2 (12.6, 24.4) | 168.0 (115.6, 225.4) | 6.3 (4.2, 8.6) | -4.2 (-4.9, -3.4) |
| Lao People's Democratic Republic | 499.1 (311.8, 719.7) | 44.0 (26.8, 64.0) | 584.2 (368.8, 866.8) | 23.9 (14.8, 35.7) | -2.2 (-2.3, -2.1) |
| Latvia | 192.4 (126.5, 269.1) | 9.7 (6.4, 13.5) | 70.4 (45.6, 99.8) | 3.1 (2.1, 4.4) | -4.9 (-5.5, -4.3) |
| Lebanon | 118.9 (70.3, 182.2) | 10.7 (6.2, 16.5) | 87.9 (49.5, 139.2) | 2.5 (1.4, 4.0) | -4.9 (-5.2, -4.6) |
| Lesotho | 87.0 (54.6, 127.2) | 19.7 (12.1, 29.3) | 162.5 (100.3, 247.4) | 28.7 (17.3, 43.9) | 2.1 (1.6, 2.6) |
| Liberia | 102.1 (65.6, 149.7) | 16.7 (10.5, 24.7) | 154.0 (90.7, 245.1) | 14.0 (8.1, 22.3) | -0.8 (-0.9, -0.6) |
| Libya | 47.8 (26.0, 77.4) | 4.8 (2.6, 7.8) | 99.9 (53.2, 169.4) | 3.5 (1.8, 6.0) | -0.9 (-1.1, -0.7) |
| Lithuania | 96.3 (65.5, 132.8) | 3.9 (2.6, 5.3) | 76.0 (48.4, 108.1) | 2.3 (1.5, 3.3) | -2.1 (-2.4, -1.8) |
| Luxembourg | 13.9 (9.1, 19.5) | 4.5 (3.0, 6.3) | 8.6 (5.1, 12.5) | 1.3 (0.8, 1.9) | -4.0 (-4.2, -3.8) |
| Madagascar | 658.9 (422.9, 946.3) | 24.4 (15.3, 35.6) | 1096.3 (659.3, 1676.1) | 18.8 (11.1, 29.1) | -0.9 (-1.0, -0.8) |
| Malawi | 308.9 (194.7, 452.9) | 15.6 (9.5, 23.2) | 519.1 (306.7, 785.9) | 13.6 (7.8, 20.9) | -0.8 (-1.0, -0.5) |
| Malaysia | 1030.0 (704.3, 1422.8) | 21.3 (14.4, 29.5) | 1818.1 (1202.2, 2556.5) | 12.0 (7.8, 17.1) | -1.8 (-1.9, -1.7) |
| Maldives | 15.7 (10.7, 21.7) | 33.1 (22.0, 46.3) | 14.0 (8.6, 20.7) | 8.2 (5.0, 12.1) | -5.0 (-5.1, -4.8) |
| Mali | 348.9 (215.3, 522.9) | 16.7 (9.9, 25.5) | 535.1 (320.1, 818.2) | 11.6 (6.7, 18.0) | -1.1 (-1.2, -0.9) |
| Malta | 8.7 (5.8, 12.0) | 3.8 (2.5, 5.2) | 6.4 (3.9, 9.4) | 1.1 (0.7, 1.6) | -4.0 (-4.2, -3.8) |
| Marshall Islands | 2.8 (1.9, 4.0) | 33.0 (21.5, 47.7) | 5.0 (3.1, 7.5) | 27.0 (16.4, 41.6) | -0.6 (-0.7, -0.5) |
| Mauritania | 107.4 (68.3, 161.9) | 20.1 (12.5, 30.7) | 123.4 (71.7, 194.1) | 10.9 (6.2, 17.2) | -2.1 (-2.3, -1.9) |
| Mauritius | 74.0 (53.8, 97.3) | 18.1 (13.0, 24.0) | 70.2 (50.4, 93.0) | 6.9 (4.9, 9.2) | -4.4 (-5.0, -3.8) |
| Mexico | 948.3 (665.1, 1261.1) | 4.4 (3.0, 5.9) | 1603.5 (1110.1, 2170.5) | 2.3 (1.6, 3.2) | -2.4 (-2.5, -2.2) |
| Micronesia (Federated States of) | 9.0 (5.7, 13.1) | 34.6 (21.4, 51.1) | 10.2 (6.5, 15.2) | 26.0 (16.0, 39.4) | -0.9 (-1.0, -0.9) |
| Monaco | 1.4 (0.7, 2.1) | 3.2 (1.7, 4.9) | 0.8 (0.4, 1.3) | 1.3 (0.7, 2.1) | -2.9 (-3.0, -2.7) |
| Mongolia | 229.1 (153.7, 317.7) | 43.0 (28.0, 60.6) | 251.8 (169.6, 346.7) | 22.5 (14.5, 31.7) | -2.5 (-3.1, -2.0) |
| Montenegro | 66.6 (40.9, 96.3) | 20.7 (12.6, 30.0) | 110.4 (65.0, 162.9) | 22.9 (13.2, 34.0) | 0.1 (-0.2, 0.4) |
| Morocco | 644.0 (359.3, 1025.9) | 8.4 (4.6, 13.6) | 949.0 (507.4, 1548.6) | 5.2 (2.7, 8.5) | -1.5 (-1.6, -1.5) |
| Mozambique | 623.8 (396.7, 918.8) | 19.9 (12.4, 29.7) | 1242.5 (731.7, 1908.0) | 20.8 (12.0, 32.1) | 0.7 (0.5, 0.9) |
| Myanmar | 5296.2 (3432.1, 7615.7) | 42.1 (26.8, 60.8) | 6102.6 (3780.5, 9029.9) | 23.3 (14.2, 34.7) | -2.3 (-2.4, -2.1) |
| Namibia | 75.8 (50.0, 107.5) | 22.8 (14.5, 33.5) | 113.8 (69.1, 170.6) | 16.5 (9.8, 25.0) | -1.3 (-1.6, -1.1) |
| Nauru | 0.9 (0.6, 1.3) | 35.0 (21.8, 51.6) | 1.0 (0.6, 1.5) | 30.8 (19.0, 46.1) | -0.5 (-0.9, -0.1) |
| Nepal | 768.4 (478.3, 1148.7) | 15.9 (9.5, 24.3) | 1231.0 (717.7, 1917.7) | 10.0 (5.6, 15.8) | -1.6 (-1.9, -1.3) |
| Netherlands | 270.9 (175.2, 380.8) | 2.4 (1.5, 3.3) | 251.6 (150.0, 366.6) | 1.2 (0.7, 1.7) | -2.7 (-3.0, -2.5) |
| New Zealand | 45.9 (29.5, 64.8) | 2.1 (1.3, 2.9) | 51.9 (30.8, 76.4) | 1.0 (0.6, 1.5) | -2.3 (-2.4, -2.2) |
| Nicaragua | 56.5 (37.9, 78.6) | 7.0 (4.6, 9.8) | 97.4 (62.6, 142.3) | 3.7 (2.4, 5.4) | -2.1 (-2.3, -1.9) |
| Niger | 232.6 (140.2, 351.7) | 16.7 (9.7, 25.9) | 581.7 (328.6, 927.7) | 14.0 (7.7, 22.5) | -0.5 (-0.6, -0.4) |
| Nigeria | 4932.8 (3193.2, 7122.1) | 21.5 (13.6, 31.5) | 4605.3 (2981.5, 6756.6) | 10.0 (6.3, 14.7) | -2.9 (-3.1, -2.7) |
| Niue | 0.3 (0.2, 0.4) | 21.5 (13.5, 31.9) | 0.2 (0.1, 0.3) | 15.6 (9.7, 23.3) | -1.2 (-1.3, -1.2) |
| North Macedonia | 135.5 (89.9, 189.5) | 13.9 (9.0, 19.8) | 151.5 (91.4, 227.7) | 9.3 (5.3, 14.3) | -1.7 (-2.1, -1.3) |
| Northern Mariana Islands | 1.6 (1.0, 2.4) | 18.2 (11.5, 26.8) | 3.1 (2.1, 4.4) | 12.0 (7.5, 17.2) | -1.4 (-1.7, -1.2) |
| Norway | 75.1 (48.5, 105.3) | 1.8 (1.2, 2.6) | 57.9 (34.1, 85.1) | 0.9 (0.6, 1.3) | -2.5 (-2.7, -2.3) |
| Oman | 29.2 (16.3, 46.7) | 8.2 (4.5, 13.2) | 31.6 (18.7, 49.4) | 3.2 (1.9, 5.0) | -2.8 (-3.0, -2.6) |
| Pakistan | 2933.9 (1873.1, 4243.3) | 9.9 (6.2, 14.5) | 5564.6 (3526.7, 8232.9) | 8.8 (5.4, 13.1) | -0.8 (-1.1, -0.6) |
| Palau | 1.1 (0.7, 1.6) | 21.1 (13.3, 31.2) | 1.9 (1.2, 2.7) | 15.3 (9.4, 22.8) | -0.9 (-1.0, -0.8) |
| Palestine | 41.9 (24.2, 64.5) | 9.4 (5.3, 14.6) | 52.3 (32.1, 78.6) | 4.2 (2.5, 6.5) | -2.6 (-3.0, -2.3) |
| Panama | 49.9 (34.3, 67.3) | 6.3 (4.3, 8.5) | 83.6 (52.6, 119.3) | 3.4 (2.2, 4.9) | -2.4 (-2.6, -2.2) |
| Papua New Guinea | 239.0 (149.9, 361.3) | 27.1 (16.5, 41.4) | 534.9 (325.5, 809.5) | 21.8 (13.0, 33.4) | -0.7 (-0.7, -0.7) |
| Paraguay | 96.2 (64.8, 133.0) | 8.1 (5.4, 11.2) | 147.1 (91.4, 219.3) | 4.7 (2.9, 7.0) | -1.8 (-1.9, -1.7) |
| Peru | 247.1 (157.0, 363.8) | 3.8 (2.4, 5.7) | 298.2 (174.9, 467.5) | 1.6 (1.0, 2.5) | -3.3 (-3.8, -2.9) |
| Philippines | 2578.6 (1851.6, 3463.8) | 17.1 (11.9, 23.2) | 7122.6 (5012.1, 9647.5) | 15.7 (10.9, 21.4) | -0.1 (-0.3, 0.2) |
| Poland | 1396.4 (945.4, 1893.2) | 6.0 (4.0, 8.1) | 715.7 (458.5, 1006.8) | 1.8 (1.1, 2.4) | -4.6 (-4.8, -4.4) |
| Portugal | 570.2 (376.0, 784.3) | 7.5 (4.8, 10.3) | 247.3 (152.1, 360.4) | 1.6 (1.0, 2.3) | -5.4 (-5.6, -5.3) |
| Puerto Rico | 48.5 (32.7, 66.9) | 2.4 (1.6, 3.4) | 47.4 (29.2, 68.8) | 1.2 (0.8, 1.7) | -2.9 (-3.2, -2.7) |
| Qatar | 5.1 (3.1, 7.8) | 11.6 (6.6, 17.9) | 11.2 (6.1, 18.4) | 3.0 (1.6, 4.9) | -5.0 (-5.8, -4.2) |
| Republic of Korea | 2362.9 (1485.1, 3386.8) | 17.3 (10.1, 25.7) | 929.0 (515.9, 1451.4) | 1.8 (1.0, 2.8) | -7.7 (-8.0, -7.5) |
| Republic of Moldova | 475.8 (336.8, 637.1) | 20.4 (14.0, 27.7) | 258.3 (179.9, 348.0) | 7.8 (5.4, 10.5) | -4.2 (-4.6, -3.8) |
| Romania | 1547.9 (1038.9, 2155.1) | 10.4 (6.8, 14.7) | 1063.4 (669.8, 1503.4) | 5.0 (3.2, 7.0) | -3.4 (-3.8, -3.0) |
| Russian Federation | 7456.7 (5149.6, 10064.5) | 7.7 (5.3, 10.5) | 6073.8 (4070.6, 8363.7) | 4.7 (3.2, 6.5) | -2.6 (-3.2, -2.0) |
| Rwanda | 470.3 (294.3, 695.8) | 31.3 (19.1, 46.9) | 359.3 (203.7, 568.8) | 11.4 (6.3, 18.5) | -4.4 (-4.8, -3.9) |
| Saint Kitts and Nevis | 3.4 (2.3, 4.8) | 16.3 (11.2, 22.6) | 2.7 (1.8, 3.7) | 7.2 (4.6, 10.0) | -2.9 (-3.2, -2.7) |
| Saint Lucia | 5.4 (3.7, 7.2) | 11.4 (7.8, 15.6) | 6.5 (4.2, 9.2) | 4.9 (3.2, 6.9) | -3.3 (-3.7, -3.0) |
| Saint Vincent and the Grenadines | 3.9 (2.6, 5.3) | 9.8 (6.6, 13.5) | 4.5 (3.1, 6.3) | 5.8 (3.9, 8.0) | -1.8 (-2.0, -1.7) |
| Samoa | 10.6 (6.8, 15.2) | 24.4 (15.2, 35.2) | 14.6 (9.3, 21.1) | 19.2 (12.0, 28.0) | -0.8 (-0.9, -0.6) |
| San Marino | 0.5 (0.3, 0.8) | 2.4 (1.4, 3.7) | 0.4 (0.2, 0.7) | 0.8 (0.4, 1.4) | -2.8 (-3.2, -2.5) |
| Sao,me and Principe | 4.7 (3.1, 6.9) | 13.7 (8.7, 20.1) | 7.0 (4.4, 10.3) | 11.7 (7.3, 17.4) | -0.6 (-0.8, -0.5) |
| Saudi Arabia | 349.1 (206.3, 541.8) | 11.5 (6.7, 18.0) | 640.0 (392.1, 976.0) | 5.9 (3.5, 9.1) | -2.4 (-2.4, -2.3) |
| Senegal | 262.7 (166.1, 385.6) | 15.2 (9.4, 22.6) | 470.0 (274.9, 720.6) | 11.5 (6.6, 17.8) | -0.8 (-1.0, -0.7) |
| Serbia | 486.4 (308.6, 703.8) | 8.9 (5.5, 13.1) | 337.7 (203.6, 523.7) | 3.6 (2.2, 5.6) | -3.6 (-3.9, -3.3) |
| Seychelles | 4.7 (3.2, 6.7) | 15.2 (10.1, 21.4) | 5.3 (3.5, 7.6) | 8.3 (5.4, 12.0) | -1.5 (-1.7, -1.3) |
| Sierra Leone | 175.6 (108.8, 259.8) | 16.1 (9.8, 23.9) | 282.3 (169.6, 431.8) | 14.1 (8.3, 21.7) | -0.2 (-0.4, 0.1) |
| Singapore | 80.1 (55.0, 108.1) | 6.8 (4.6, 9.2) | 55.2 (35.1, 78.7) | 1.2 (0.8, 1.7) | -5.5 (-5.7, -5.3) |
| Slovakia | 203.6 (131.2, 293.7) | 6.2 (3.9, 8.9) | 113.9 (70.9, 172.3) | 2.1 (1.3, 3.2) | -3.6 (-3.8, -3.5) |
| Slovenia | 45.0 (30.1, 62.6) | 3.3 (2.2, 4.6) | 25.8 (14.9, 38.2) | 0.9 (0.6, 1.4) | -4.7 (-5.0, -4.3) |
| Solomon Islands | 22.5 (13.1, 33.7) | 33.9 (19.7, 50.9) | 51.4 (32.5, 77.3) | 29.8 (18.5, 45.2) | -0.3 (-0.4, -0.2) |
| Somalia | 278.6 (163.7, 436.8) | 23.2 (13.5, 36.2) | 485.6 (269.0, 797.1) | 15.8 (8.6, 26.3) | -1.3 (-1.3, -1.2) |
| South Africa | 1113.0 (753.2, 1531.5) | 9.8 (6.5, 13.6) | 2561.4 (1835.2, 3427.3) | 10.4 (7.3, 14.1) | 0.1 (-0.4, 0.6) |
| South Sudan | 230.6 (134.8, 362.2) | 16.9 (9.7, 26.7) | 236.9 (132.5, 384.0) | 12.1 (6.7, 19.7) | -1.3 (-1.7, -1.0) |
| Spain | 1098.4 (689.9, 1556.7) | 3.6 (2.2, 5.1) | 715.1 (407.5, 1072.4) | 1.1 (0.7, 1.6) | -3.8 (-3.9, -3.7) |
| Sri Lanka | 822.1 (543.3, 1158.6) | 15.4 (10.0, 22.0) | 1229.5 (665.1, 1942.5) | 8.5 (4.6, 13.6) | -1.5 (-1.8, -1.3) |
| Sudan | 785.4 (450.2, 1226.3) | 15.9 (8.9, 25.0) | 694.0 (363.6, 1149.5) | 6.7 (3.5, 11.1) | -3.1 (-3.3, -3.0) |
| Suriname | 15.0 (10.1, 20.8) | 10.9 (7.2, 15.2) | 27.4 (17.1, 40.4) | 7.8 (4.8, 11.5) | -1.1 (-1.4, -0.8) |
| Sweden | 197.7 (123.7, 284.1) | 2.2 (1.4, 3.1) | 158.4 (91.1, 237.5) | 1.1 (0.7, 1.7) | -2.1 (-2.2, -2.0) |
| Switzerland | 136.7 (83.5, 198.6) | 2.2 (1.4, 3.2) | 83.4 (47.5, 123.5) | 0.7 (0.4, 1.0) | -3.7 (-3.8, -3.6) |
| Syrian Arab Republic | 307.8 (186.2, 461.8) | 11.1 (6.5, 16.8) | 431.5 (252.5, 672.6) | 6.5 (3.7, 10.3) | -2.3 (-2.6, -1.9) |
| Taiwan (Province of China) | 1045.2 (748.0, 1374.0) | 12.9 (8.9, 17.3) | 556.4 (365.2, 774.3) | 2.4 (1.6, 3.3) | -5.5 (-5.8, -5.1) |
| Tajikistan | 228.8 (148.7, 327.4) | 15.8 (10.1, 22.9) | 321.9 (203.9, 468.6) | 11.8 (7.2, 17.5) | -1.3 (-1.8, -0.9) |
| Thailand | 2544.9 (1683.9, 3618.4) | 14.2 (9.2, 20.4) | 4508.9 (2783.9, 6778.0) | 7.6 (4.7, 11.5) | -2.6 (-2.9, -2.4) |
| Timor-Leste | 37.1 (23.8, 53.8) | 26.9 (16.8, 39.2) | 106.4 (63.5, 161.1) | 23.1 (13.6, 35.4) | -0.4 (-0.5, -0.2) |
| Togo | 111.1 (71.4, 162.9) | 17.2 (10.7, 25.7) | 303.8 (182.4, 461.5) | 15.4 (9.0, 23.7) | -0.3 (-0.4, -0.2) |
| Tokelau | 0.2 (0.1, 0.3) | 24.4 (14.9, 36.7) | 0.1 (0.1, 0.2) | 14.3 (8.7, 21.7) | -1.8 (-1.9, -1.8) |
| Tonga | 3.0 (1.9, 4.5) | 10.5 (6.4, 15.8) | 3.7 (2.3, 5.6) | 8.6 (5.2, 13.1) | -0.4 (-0.6, -0.3) |
| Trinidad and,bago | 33.2 (23.0, 45.1) | 7.4 (5.1, 10.1) | 39.5 (24.8, 57.2) | 3.7 (2.3, 5.3) | -2.8 (-3.1, -2.5) |
| Tunisia | 165.0 (93.8, 259.3) | 6.6 (3.6, 10.5) | 234.8 (116.3, 407.7) | 3.4 (1.6, 5.8) | -2.5 (-2.6, -2.4) |
| Turkey | 1945.8 (1224.3, 2865.4) | 10.9 (6.7, 16.2) | 2027.4 (1201.2, 3103.5) | 4.1 (2.4, 6.3) | -3.2 (-3.6, -2.8) |
| Turkmenistan | 128.8 (91.6, 174.3) | 12.3 (8.6, 17.0) | 338.7 (225.5, 489.2) | 15.3 (9.9, 22.2) | 0.4 (-0.1, 0.9) |
| Tuvalu | 1.2 (0.8, 1.8) | 34.9 (22.5, 51.1) | 1.2 (0.8, 1.8) | 21.4 (13.5, 31.7) | -1.5 (-1.6, -1.4) |
| Uganda | 537.6 (317.3, 825.4) | 16.2 (9.4, 25.1) | 700.4 (390.5, 1108.7) | 9.4 (5.1, 15.1) | -2.5 (-2.7, -2.2) |
| Ukraine | 2350.3 (1586.1, 3264.9) | 5.9 (4.0, 8.3) | 1683.1 (1024.0, 2487.6) | 4.1 (2.5, 6.0) | -1.9 (-2.2, -1.6) |
| United Arab Emirates | 18.8 (10.8, 29.4) | 8.5 (4.8, 13.3) | 57.4 (33.8, 87.9) | 4.2 (2.3, 6.6) | -0.8 (-1.3, -0.2) |
| United Kingdom | 1171.5 (752.0, 1630.4) | 2.2 (1.5, 3.1) | 1006.1 (599.3, 1451.6) | 1.2 (0.8, 1.7) | -1.8 (-2.0, -1.7) |
| United Republic of Tanzania | 712.1 (432.7, 1055.8) | 12.6 (7.5, 18.9) | 1054.8 (593.7, 1692.9) | 8.2 (4.5, 13.2) | -1.4 (-1.5, -1.4) |
| United States of America | 3516.3 (2308.5, 4853.3) | 1.9 (1.3, 2.7) | 6041.2 (3898.9, 8423.0) | 1.8 (1.2, 2.5) | -0.4 (-0.6, -0.1) |
| United States Virgin Islands | 2.3 (1.4, 3.4) | 5.1 (3.1, 7.7) | 2.1 (1.3, 3.3) | 2.1 (1.3, 3.3) | -2.7 (-2.9, -2.5) |
| Uruguay | 99.9 (65.3, 139.6) | 4.6 (3.0, 6.5) | 63.4 (39.0, 91.4) | 2.0 (1.3, 2.8) | -3.2 (-3.5, -2.9) |
| Uzbekistan | 1024.6 (717.5, 1361.0) | 16.6 (11.5, 22.1) | 1250.3 (863.6, 1728.7) | 9.5 (6.3, 13.2) | -2.2 (-2.8, -1.7) |
| Vanuatu | 9.0 (5.7, 13.4) | 28.3 (17.3, 42.1) | 21.3 (13.7, 31.2) | 23.4 (14.6, 34.6) | -0.7 (-0.8, -0.7) |
| Venezuela (Bolivarian Republic of) | 424.4 (294.4, 573.7) | 8.3 (5.7, 11.3) | 879.7 (550.5, 1269.3) | 5.4 (3.3, 7.8) | -2.2 (-2.5, -1.9) |
| Viet Nam | 5443.5 (3363.3, 8278.9) | 25.5 (15.6, 38.9) | 9538.7 (5704.2, 14365.4) | 18.2 (10.7, 27.6) | -0.8 (-0.9, -0.6) |
| Yemen | 444.3 (254.3, 705.4) | 17.5 (9.7, 28.1) | 685.5 (375.0, 1131.8) | 9.4 (5.0, 15.7) | -2.4 (-2.6, -2.2) |
| Zambia | 267.2 (167.3, 398.5) | 18.7 (11.4, 28.3) | 520.1 (302.2, 817.3) | 15.1 (8.6, 23.9) | -1.1 (-1.3, -0.9) |
| Zimbabwe | 266.5 (174.4, 382.7) | 13.1 (8.3, 19.2) | 712.9 (456.4, 1042.0) | 19.9 (12.4, 29.2) | 2.1 (1.5, 2.8) |
| **DALYs** |  |  |  |  |  |
| Global | 6224604.8 (4483293.4, 8204935.0) | 276.6 (197.1, 366.6) | 8012540.7 (5656124.7, 10694840.6) | 166.7 (117.2, 223.1) | -1.7 (-1.8, -1.6) |
| High SDI | 485938.4 (340866.8, 649030.0) | 80.7 (56.9, 107.5) | 410779.5 (276730.5, 561452.5) | 37.6 (26.0, 50.9) | -2.6 (-2.7, -2.5) |
| High-middle SDI | 1464315.1 (1043031.3, 1954394.7) | 264.3 (186.0, 355.1) | 1349383.3 (924472.5, 1851525.4) | 124.7 (85.2, 171.6) | -2.7 (-2.9, -2.4) |
| Middle SDI | 2366666.4 (1697290.6, 3171630.5) | 404.0 (283.3, 548.5) | 3299550.2 (2310202.4, 4462477.8) | 217.5 (150.3, 296.5) | -1.9 (-2.1, -1.8) |
| Low-middle SDI | 1348732.5 (966348.9, 1777381.0) | 376.2 (265.8, 499.0) | 2139648.9 (1517337.5, 2858121.8) | 257.3 (180.6, 345.7) | -1.3 (-1.3, -1.2) |
| Low SDI | 553003.6 (389010.9, 737509.4) | 415.6 (287.6, 559.5) | 806599.2 (561290.8, 1102569.7) | 269.7 (185.2, 370.7) | -1.6 (-1.6, -1.5) |
| High-income Asia Pacific | 153267.2 (104719.9, 208796.1) | 136.6 (92.1, 187.3) | 100430.6 (63363.5, 142773.6) | 43.3 (28.8, 60.0) | -3.8 (-4.0, -3.6) |
| High-income North America | 84791.8 (59070.6, 113382.2) | 44.5 (31.4, 59.2) | 134269.4 (92938.2, 180670.8) | 38.1 (26.7, 51.1) | -0.6 (-0.8, -0.4) |
| Western Europe | 183840.5 (126695.4, 246705.3) | 57.7 (40.2, 76.9) | 114880.1 (74509.0, 161144.9) | 21.7 (14.7, 29.8) | -3.3 (-3.4, -3.2) |
| Australasia | 5603.2 (3774.3, 7658.8) | 42.4 (28.5, 58.1) | 5338.8 (3341.5, 7665.8) | 17.5 (11.2, 24.9) | -2.9 (-3.0, -2.8) |
| Andean Latin America | 14349.4 (9844.3, 19832.8) | 118.9 (81.1, 164.8) | 17036.5 (11374.1, 24210.3) | 51.0 (34.0, 72.6) | -3.0 (-3.3, -2.8) |
| Tropical Latin America | 131709.5 (100071.7, 166856.4) | 239.4 (180.2, 304.5) | 116922.3 (87927.5, 148832.9) | 80.6 (60.3, 102.9) | -3.7 (-3.8, -3.7) |
| Central Latin America | 63635.5 (46773.0, 83008.9) | 132.7 (96.5, 173.8) | 101508.1 (72886.8, 133321.3) | 72.0 (51.4, 94.8) | -2.5 (-2.7, -2.3) |
| Southern Latin America | 35170.0 (24470.4, 47618.8) | 136.2 (94.3, 184.8) | 22633.5 (15461.9, 31254.6) | 48.0 (32.9, 66.1) | -3.4 (-3.5, -3.2) |
| Caribbean | 28929.0 (20603.9, 38338.2) | 197.3 (140.3, 261.7) | 39383.1 (27126.1, 54898.1) | 133.2 (91.6, 185.9) | -1.3 (-1.4, -1.1) |
| Central Europe | 152247.3 (109394.3, 200045.8) | 183.5 (130.7, 242.4) | 78111.9 (53766.2, 105400.4) | 66.0 (46.1, 88.5) | -4.1 (-4.4, -3.8) |
| Eastern Europe | 280777.0 (202292.3, 371822.5) | 181.2 (129.8, 240.4) | 215111.4 (151575.1, 287521.7) | 118.4 (83.5, 158.3) | -2.3 (-2.9, -1.7) |
| Central Asia | 107657.6 (80933.3, 137797.3) | 408.7 (303.9, 526.3) | 117245.4 (86445.4, 151819.5) | 253.7 (183.0, 332.3) | -2.0 (-2.5, -1.6) |
| North Africa and Middle East | 259518.2 (179368.5, 352218.6) | 261.0 (177.3, 357.6) | 309060.0 (210415.8, 425476.8) | 114.5 (76.5, 158.6) | -2.9 (-3.0, -2.8) |
| South Asia | 1005676.0 (701873.6, 1354984.5) | 296.2 (202.9, 402.2) | 1691587.8 (1168769.2, 2324836.5) | 198.6 (135.5, 274.6) | -1.3 (-1.4, -1.3) |
| Southeast Asia | 969856.4 (694051.5, 1285791.9) | 643.0 (455.2, 857.1) | 1799247.8 (1257934.1, 2426669.5) | 465.4 (321.8, 631.5) | -1.0 (-1.1, -0.9) |
| East Asia | 2187656.4 (1519615.5, 3036006.5) | 467.5 (313.7, 660.2) | 2292075.9 (1516157.3, 3264405.7) | 190.5 (124.3, 273.5) | -2.7 (-3.0, -2.4) |
| Oceania | 10794.5 (7244.2, 15512.6) | 618.0 (409.9, 889.6) | 21703.5 (14473.2, 31291.7) | 486.4 (320.4, 702.9) | -0.8 (-0.8, -0.8) |
| Western Sub-Saharan Africa | 235445.9 (162781.7, 319709.1) | 464.0 (315.7, 636.9) | 338730.6 (232430.3, 463177.1) | 288.1 (194.6, 396.2) | -1.7 (-1.7, -1.6) |
| Eastern Sub-Saharan Africa | 193825.2 (130926.3, 268936.7) | 438.5 (292.3, 612.7) | 263396.7 (172745.6, 371861.2) | 262.1 (169.5, 372.5) | -1.9 (-2.0, -1.8) |
| Central Sub-Saharan Africa | 72792.6 (47821.8, 102810.6) | 553.5 (356.4, 786.8) | 134437.0 (85889.0, 198740.6) | 422.1 (265.2, 628.6) | -1.0 (-1.1, -1.0) |
| Southern Sub-Saharan Africa | 47061.8 (33839.1, 62857.5) | 290.7 (207.0, 389.9) | 99430.6 (73542.4, 129912.5) | 294.3 (215.0, 387.0) | 0.2 (-0.3, 0.7) |
| Afghanistan | 24888.2 (14124.8, 38996.0) | 622.6 (348.3, 983.0) | 21463.0 (12071.1, 33862.9) | 336.2 (184.9, 534.3) | -2.4 (-2.7, -2.1) |
| Albania | 3683.8 (2435.2, 5172.9) | 354.9 (227.1, 506.5) | 5002.2 (2849.8, 7594.3) | 210.3 (118.5, 320.6) | -1.7 (-1.9, -1.4) |
| Algeria | 12891.6 (7513.2, 20240.2) | 188.0 (106.3, 299.1) | 17928.7 (9970.0, 29259.0) | 89.9 (48.7, 148.1) | -2.5 (-2.5, -2.4) |
| American Samoa | 62.8 (43.1, 87.5) | 459.1 (311.5, 641.6) | 100.1 (66.4, 142.3) | 360.8 (236.2, 516.0) | -0.9 (-1.1, -0.7) |
| Andorra | 9.1 (5.2, 14.7) | 28.4 (15.9, 46.1) | 13.1 (7.0, 21.4) | 15.3 (8.2, 25.0) | -1.7 (-1.9, -1.5) |
| Angola | 14717.0 (9504.7, 21200.3) | 643.0 (407.4, 931.8) | 26454.6 (16476.6, 39154.8) | 382.9 (234.4, 570.3) | -2.0 (-2.1, -1.9) |
| Antigua and Barbuda | 65.4 (45.9, 86.8) | 230.2 (164.3, 303.3) | 70.4 (50.0, 93.5) | 115.6 (81.2, 154.5) | -2.6 (-2.9, -2.4) |
| Argentina | 26017.5 (17964.3, 35416.7) | 144.9 (99.6, 197.7) | 16027.9 (10927.7, 22238.3) | 53.0 (36.3, 73.4) | -3.2 (-3.4, -3.1) |
| Armenia | 2976.4 (2157.2, 3902.2) | 197.9 (140.9, 261.7) | 1508.1 (1079.1, 1991.7) | 63.9 (45.7, 84.5) | -4.6 (-5.2, -4.0) |
| Australia | 4628.6 (3089.4, 6383.5) | 42.0 (27.9, 58.1) | 4415.9 (2725.5, 6397.4) | 17.1 (10.8, 24.6) | -3.0 (-3.1, -2.9) |
| Austria | 3693.1 (2458.2, 5070.1) | 56.2 (38.0, 76.6) | 1846.6 (1162.2, 2602.4) | 17.9 (11.7, 24.9) | -4.1 (-4.5, -3.8) |
| Azerbaijan | 11508.8 (8185.9, 15599.1) | 413.6 (289.6, 565.8) | 15438.4 (10098.6, 22031.1) | 284.9 (181.7, 410.0) | -1.3 (-1.6, -1.0) |
| Bahamas | 171.0 (122.4, 229.9) | 189.5 (135.0, 255.3) | 245.4 (164.6, 345.3) | 101.9 (67.7, 144.0) | -2.1 (-2.2, -2.0) |
| Bahrain | 237.5 (156.8, 333.8) | 230.3 (147.8, 326.5) | 431.3 (267.3, 643.5) | 81.2 (48.3, 123.0) | -4.4 (-4.9, -3.9) |
| Bangladesh | 133393.6 (86079.6, 193311.8) | 490.9 (315.1, 711.4) | 214573.4 (130295.8, 326944.9) | 276.7 (166.1, 422.9) | -1.7 (-1.9, -1.4) |
| Barbados | 209.4 (144.1, 286.3) | 139.4 (97.6, 188.8) | 232.9 (150.8, 331.1) | 82.8 (53.6, 117.9) | -2.0 (-2.2, -1.7) |
| Belarus | 10546.6 (7433.4, 14576.2) | 148.6 (104.4, 206.0) | 9130.9 (6044.9, 12899.7) | 109.3 (72.4, 154.6) | -1.9 (-2.5, -1.4) |
| Belgium | 5358.1 (3589.6, 7323.0) | 63.2 (42.8, 86.1) | 3250.1 (2083.0, 4584.7) | 24.7 (16.4, 34.3) | -3.1 (-3.2, -3.0) |
| Belize | 79.5 (57.0, 106.7) | 152.7 (109.5, 204.4) | 181.9 (130.1, 244.0) | 103.6 (73.1, 139.8) | -1.8 (-2.3, -1.3) |
| Benin | 5292.4 (3512.6, 7544.8) | 474.8 (313.0, 679.4) | 9573.7 (6029.1, 13922.9) | 322.1 (201.1, 470.2) | -1.3 (-1.4, -1.2) |
| Bermuda | 26.4 (18.0, 36.7) | 75.5 (51.3, 104.9) | 20.2 (13.2, 28.7) | 28.3 (18.7, 39.9) | -3.4 (-3.5, -3.3) |
| Bhutan | 375.2 (226.7, 582.8) | 265.1 (157.9, 414.6) | 471.1 (284.1, 731.7) | 139.0 (83.8, 215.5) | -2.2 (-2.3, -2.1) |
| Bolivia (Plurinational State of) | 3994.3 (2381.7, 6251.1) | 211.5 (125.3, 331.8) | 4870.7 (2779.4, 7765.3) | 93.0 (52.7, 148.7) | -2.7 (-2.9, -2.6) |
| Bosnia and Herzegovina | 2416.1 (1583.0, 3427.0) | 105.6 (67.9, 151.8) | 1734.4 (1012.7, 2604.7) | 50.9 (29.9, 76.3) | -2.7 (-3.0, -2.5) |
| Botswana | 1741.8 (1094.0, 2566.8) | 539.5 (335.5, 796.6) | 2174.2 (1353.2, 3287.3) | 257.8 (160.4, 388.9) | -2.4 (-2.7, -2.1) |
| Brazil | 129244.5 (98275.4, 163777.4) | 240.4 (181.1, 305.9) | 113173.2 (85166.1, 144199.8) | 79.9 (59.8, 102.0) | -3.8 (-3.9, -3.7) |
| Brunei Darussalam | 172.5 (110.8, 247.9) | 281.6 (178.7, 406.7) | 255.5 (165.8, 366.2) | 121.7 (76.4, 176.8) | -2.8 (-3.0, -2.5) |
| Bulgaria | 24291.2 (17291.3, 32006.7) | 364.7 (252.4, 487.3) | 9472.0 (6400.0, 13015.9) | 128.6 (88.1, 176.0) | -4.4 (-4.9, -4.0) |
| Burkina Faso | 8140.1 (5152.1, 11815.5) | 323.3 (201.4, 473.8) | 14348.6 (8686.5, 21662.5) | 264.2 (158.4, 400.6) | -0.4 (-0.5, -0.4) |
| Burundi | 8976.5 (5248.3, 13595.9) | 659.6 (384.4, 997.2) | 9157.8 (5418.9, 13940.7) | 310.4 (181.9, 473.5) | -3.0 (-3.3, -2.7) |
| Cabo Verde | 270.9 (171.9, 396.3) | 225.2 (144.4, 327.7) | 445.0 (273.2, 672.1) | 174.1 (106.8, 263.7) | -1.1 (-1.3, -0.9) |
| Cambodia | 21206.8 (14238.0, 29886.1) | 809.5 (539.0, 1145.2) | 36082.7 (23040.2, 53466.1) | 510.9 (321.6, 758.5) | -1.7 (-1.8, -1.6) |
| Cameroon | 13338.3 (8480.3, 19549.3) | 507.8 (317.2, 751.9) | 34536.5 (20420.8, 53583.5) | 449.7 (264.5, 697.9) | -0.3 (-0.7, 0.1) |
| Canada | 6324.8 (4220.6, 8820.0) | 34.9 (23.3, 48.7) | 7251.9 (4596.9, 10408.4) | 18.5 (11.9, 26.4) | -2.0 (-2.1, -2.0) |
| Central African Republic | 5717.4 (3472.2, 8707.3) | 836.0 (501.5, 1273.6) | 8988.7 (5241.2, 14186.1) | 665.9 (383.9, 1047.2) | -0.8 (-0.9, -0.8) |
| Chad | 6362.9 (4098.2, 9174.7) | 397.4 (254.2, 575.1) | 13488.8 (8332.0, 20332.5) | 387.0 (236.6, 585.8) | -0.2 (-0.4, 0.0) |
| Chile | 6717.7 (4653.1, 9168.7) | 118.2 (81.4, 161.7) | 5276.1 (3515.1, 7364.3) | 37.5 (25.1, 52.3) | -3.5 (-3.7, -3.3) |
| China | 2118242.3 (1467152.6, 2953532.4) | 471.0 (315.0, 668.2) | 2200339.0 (1445244.0, 3147436.7) | 189.6 (122.8, 273.4) | -2.7 (-3.0, -2.4) |
| Colombia | 15179.7 (10935.7, 20072.6) | 150.2 (107.1, 199.4) | 13071.6 (8804.2, 18362.2) | 42.9 (28.9, 60.4) | -4.8 (-5.0, -4.6) |
| Comoros | 494.0 (289.6, 738.9) | 423.0 (247.8, 632.9) | 623.3 (368.8, 969.0) | 221.0 (129.6, 345.1) | -2.4 (-2.7, -2.1) |
| Congo | 4627.0 (2876.3, 6846.5) | 741.0 (455.2, 1100.0) | 6726.3 (4048.7, 10231.5) | 414.7 (249.7, 626.1) | -2.2 (-2.4, -2.1) |
| Cook Islands | 24.8 (16.2, 35.8) | 345.0 (223.6, 499.3) | 23.1 (14.9, 34.0) | 165.9 (106.3, 245.5) | -2.3 (-2.5, -2.1) |
| Costa Rica | 953.1 (683.8, 1260.4) | 97.6 (69.9, 129.1) | 1409.7 (987.5, 1897.5) | 46.4 (32.4, 62.5) | -3.0 (-3.3, -2.6) |
| Coted'Ivoire | 9940.1 (6268.0, 14789.2) | 392.7 (244.0, 588.9) | 21709.0 (13155.5, 33234.5) | 307.9 (185.0, 469.8) | -0.9 (-1.0, -0.7) |
| Croatia | 5616.7 (4011.6, 7478.4) | 164.2 (115.8, 220.3) | 2110.1 (1389.1, 2980.0) | 43.3 (29.0, 60.7) | -4.8 (-5.1, -4.6) |
| Cuba | 6341.4 (4432.4, 8592.8) | 112.0 (78.3, 151.6) | 6558.4 (4440.2, 9087.9) | 62.7 (42.5, 86.8) | -2.3 (-2.6, -2.1) |
| Cyprus | 447.9 (270.6, 660.2) | 111.9 (63.7, 169.6) | 342.7 (204.9, 523.2) | 30.2 (17.9, 46.2) | -4.5 (-4.7, -4.3) |
| Czechia | 8252.0 (5806.6, 11030.8) | 108.3 (76.3, 144.6) | 2732.1 (1775.7, 3839.8) | 23.6 (15.5, 33.0) | -4.7 (-4.9, -4.6) |
| Democratic People's Republic of Korea | 43301.3 (27234.0, 62364.9) | 479.6 (296.7, 697.9) | 78184.3 (49378.0, 114371.3) | 422.0 (264.5, 620.7) | -0.4 (-0.6, -0.2) |
| Democratic Republic of the Congo | 45204.8 (27419.2, 67337.4) | 497.1 (296.5, 745.8) | 89619.0 (53258.5, 139097.8) | 426.1 (250.2, 665.1) | -0.6 (-0.7, -0.5) |
| Denmark | 2738.6 (1833.6, 3773.3) | 61.1 (41.6, 83.7) | 1741.2 (1117.9, 2437.8) | 25.9 (16.9, 36.0) | -3.1 (-3.3, -2.9) |
| Djibouti | 296.2 (174.4, 470.1) | 355.9 (208.5, 562.2) | 902.0 (514.8, 1474.4) | 230.4 (130.6, 377.8) | -1.6 (-1.7, -1.5) |
| Dominica | 66.2 (45.1, 92.5) | 203.5 (139.1, 284.1) | 66.4 (42.6, 96.9) | 140.2 (89.2, 205.3) | -1.2 (-1.4, -1.0) |
| Dominican Republic | 3671.5 (2460.0, 5135.0) | 169.6 (112.6, 237.8) | 8211.1 (5223.0, 12347.3) | 145.2 (92.2, 218.4) | 0.0 (-0.2, 0.2) |
| Ecuador | 3593.8 (2551.8, 4843.3) | 113.3 (80.1, 152.8) | 4305.1 (2780.0, 6360.5) | 46.9 (30.2, 69.4) | -3.0 (-3.2, -2.8) |
| Egypt | 50835.7 (27396.5, 79623.6) | 323.8 (166.7, 520.0) | 53086.6 (29400.5, 83980.1) | 140.9 (76.2, 226.3) | -2.6 (-2.7, -2.5) |
| El Salvador | 2499.9 (1686.6, 3483.7) | 147.4 (99.3, 205.1) | 2333.3 (1473.5, 3445.4) | 69.6 (44.1, 102.6) | -2.5 (-2.9, -2.1) |
| Equatorial Guinea | 860.5 (533.6, 1283.2) | 759.0 (465.7, 1134.4) | 780.1 (414.0, 1312.0) | 260.4 (138.9, 433.9) | -4.0 (-4.5, -3.6) |
| Eritrea | 4572.8 (2836.4, 6991.5) | 631.9 (386.7, 974.2) | 5960.1 (3490.2, 9470.2) | 351.2 (204.3, 554.7) | -1.9 (-2.1, -1.8) |
| Estonia | 1700.7 (1187.0, 2284.9) | 151.4 (105.5, 203.6) | 455.9 (306.1, 636.3) | 34.2 (23.5, 47.4) | -6.3 (-6.8, -5.8) |
| Eswatini | 906.2 (582.0, 1309.5) | 556.2 (353.3, 807.2) | 1656.7 (941.6, 2707.7) | 496.5 (281.7, 805.2) | 0.1 (-0.5, 0.8) |
| Ethiopia | 56427.5 (37076.5, 86054.5) | 473.3 (306.5, 723.2) | 44230.6 (27936.8, 65037.1) | 176.4 (110.6, 260.5) | -3.7 (-3.9, -3.5) |
| Fiji | 1127.0 (753.4, 1616.1) | 516.0 (339.9, 742.6) | 1630.4 (1045.7, 2393.0) | 367.5 (232.3, 541.7) | -1.2 (-1.4, -1.1) |
| Finland | 1883.8 (1243.8, 2630.7) | 47.9 (31.8, 66.7) | 1539.2 (980.2, 2199.0) | 21.6 (14.2, 30.5) | -2.4 (-2.5, -2.3) |
| France | 18637.6 (12287.6, 25874.2) | 41.3 (27.8, 56.6) | 13057.5 (8051.3, 18877.6) | 16.0 (10.4, 22.7) | -3.4 (-3.6, -3.2) |
| Gabon | 1666.0 (1033.1, 2467.3) | 517.2 (317.2, 770.4) | 1868.3 (1116.9, 2923.2) | 307.5 (182.5, 480.9) | -1.8 (-1.9, -1.7) |
| Gambia | 827.1 (511.3, 1240.3) | 394.8 (241.7, 594.3) | 2168.3 (1316.6, 3313.1) | 376.2 (228.2, 575.5) | -0.3 (-0.4, -0.1) |
| Georgia | 19617.9 (14080.8, 25998.3) | 565.2 (402.1, 753.3) | 8683.6 (6048.3, 11683.7) | 269.9 (189.4, 361.7) | -3.0 (-3.7, -2.2) |
| Germany | 35250.2 (23071.3, 50130.0) | 50.8 (33.8, 71.8) | 21625.8 (13557.9, 31064.3) | 20.1 (13.2, 28.3) | -3.3 (-3.5, -3.0) |
| Ghana | 16261.7 (10194.6, 23980.8) | 419.3 (259.8, 624.2) | 33068.3 (20168.8, 50538.1) | 318.8 (191.6, 492.4) | -0.6 (-0.8, -0.3) |
| Greece | 11737.9 (7859.5, 16066.4) | 138.9 (92.8, 190.4) | 7345.9 (4637.7, 10439.5) | 54.2 (36.3, 74.9) | -3.5 (-3.8, -3.3) |
| Greenland | 39.4 (25.5, 57.1) | 208.0 (131.1, 302.7) | 36.0 (23.0, 53.1) | 93.1 (57.4, 139.2) | -2.6 (-2.6, -2.5) |
| Grenada | 101.8 (70.8, 136.7) | 278.7 (196.2, 371.2) | 88.7 (61.0, 120.8) | 134.2 (91.2, 183.8) | -2.5 (-2.6, -2.3) |
| Guam | 88.0 (60.7, 122.9) | 195.2 (131.9, 274.3) | 153.1 (104.4, 212.0) | 136.0 (92.3, 189.1) | -0.9 (-1.3, -0.6) |
| Guatemala | 3532.8 (2583.7, 4668.8) | 168.3 (121.2, 224.1) | 6786.2 (4691.0, 9233.2) | 107.9 (74.1, 147.0) | -2.2 (-2.5, -1.9) |
| Guinea | 8095.7 (5242.4, 11718.9) | 426.6 (273.8, 621.8) | 12327.7 (7657.7, 18497.1) | 371.8 (229.4, 558.7) | -0.2 (-0.3, -0.1) |
| Guinea-Bissau | 1916.2 (1198.8, 2867.6) | 792.0 (491.7, 1189.1) | 2707.4 (1675.3, 4078.7) | 586.7 (360.8, 884.5) | -0.9 (-0.9, -0.8) |
| Guyana | 1429.5 (1041.6, 1912.7) | 643.6 (465.9, 863.2) | 1013.1 (669.1, 1449.1) | 264.5 (173.3, 379.7) | -2.5 (-2.8, -2.3) |
| Haiti | 11151.0 (7159.8, 15911.9) | 578.2 (365.1, 830.0) | 15917.6 (9518.7, 24862.5) | 366.0 (215.5, 572.2) | -1.3 (-1.4, -1.2) |
| Honduras | 2874.5 (1893.0, 4137.6) | 235.7 (154.3, 340.1) | 9299.5 (5831.7, 13858.7) | 259.2 (161.8, 387.0) | 0.5 (0.3, 0.7) |
| Hungary | 13255.8 (9131.4, 17876.4) | 166.5 (114.6, 224.5) | 3623.5 (2401.4, 5057.6) | 36.2 (24.3, 50.3) | -5.4 (-5.6, -5.1) |
| Iceland | 57.4 (37.8, 80.3) | 36.0 (23.9, 50.1) | 43.2 (27.3, 61.8) | 13.1 (8.4, 18.6) | -3.4 (-3.5, -3.3) |
| India | 773577.2 (537760.2, 1048035.8) | 279.5 (190.8, 382.2) | 1286221.9 (878194.8, 1772481.9) | 186.6 (126.0, 258.4) | -1.3 (-1.5, -1.2) |
| Indonesia | 446926.2 (316612.7, 604041.8) | 750.9 (523.2, 1025.7) | 926434.9 (616533.5, 1294036.5) | 643.8 (422.5, 907.8) | -0.4 (-0.6, -0.3) |
| Iran (Islamic Republic of) | 15746.0 (11235.3, 20982.2) | 100.2 (69.5, 135.6) | 19685.4 (13910.0, 26441.5) | 43.6 (30.3, 59.0) | -2.6 (-2.8, -2.5) |
| Iraq | 17778.3 (11509.0, 25816.0) | 376.9 (243.3, 547.2) | 31947.8 (19245.7, 48604.3) | 218.6 (129.9, 333.1) | -2.4 (-2.6, -2.2) |
| Ireland | 1412.6 (991.5, 1891.4) | 61.4 (43.0, 82.3) | 860.1 (581.9, 1179.5) | 19.2 (13.1, 26.3) | -3.6 (-3.8, -3.4) |
| Israel | 2161.4 (1488.8, 2938.8) | 79.8 (54.9, 108.5) | 1733.0 (1140.8, 2398.3) | 25.3 (16.9, 34.9) | -4.3 (-4.6, -4.0) |
| Italy | 29042.1 (20261.1, 38763.3) | 59.5 (41.7, 79.2) | 18749.5 (11730.4, 26823.7) | 22.7 (15.0, 31.5) | -3.0 (-3.1, -2.9) |
| Jamaica | 1829.7 (1306.5, 2470.2) | 191.6 (137.8, 257.4) | 2298.2 (1490.3, 3303.0) | 136.0 (88.2, 195.4) | -0.9 (-1.4, -0.3) |
| Japan | 91234.3 (61619.3, 124943.4) | 97.2 (65.1, 133.9) | 78170.4 (48885.1, 111735.6) | 45.6 (30.7, 63.0) | -2.4 (-2.5, -2.2) |
| Jordan | 1479.5 (944.7, 2163.9) | 177.9 (112.1, 262.3) | 2665.1 (1651.2, 4001.1) | 59.6 (36.1, 90.1) | -4.2 (-4.6, -3.8) |
| Kazakhstan | 22981.0 (16746.5, 30487.9) | 320.6 (230.7, 428.2) | 27444.9 (19280.2, 36890.6) | 275.9 (190.1, 374.0) | -1.2 (-1.7, -0.8) |
| Kenya | 9905.8 (6347.8, 14698.6) | 211.8 (134.5, 315.7) | 26403.6 (16685.3, 38870.1) | 198.4 (123.0, 295.2) | 0.1 (-0.2, 0.3) |
| Kiribati | 171.3 (115.0, 241.8) | 751.2 (499.2, 1063.6) | 297.8 (194.7, 441.3) | 646.2 (419.2, 957.9) | -0.5 (-0.6, -0.5) |
| Kuwait | 263.8 (181.8, 363.8) | 59.7 (40.5, 82.8) | 663.0 (425.2, 966.3) | 32.5 (20.4, 47.9) | -1.9 (-2.7, -1.1) |
| Kyrgyzstan | 7865.3 (5779.2, 10208.1) | 466.1 (339.6, 607.8) | 4969.5 (3514.7, 6557.2) | 167.3 (116.7, 222.9) | -4.2 (-4.9, -3.4) |
| Lao People's Democratic Republic | 14479.9 (9169.3, 20900.5) | 1158.8 (726.8, 1674.5) | 16624.7 (10630.0, 24559.2) | 595.5 (379.0, 880.3) | -2.4 (-2.5, -2.3) |
| Latvia | 4418.2 (3049.3, 6017.9) | 223.8 (154.5, 304.8) | 1492.3 (1024.4, 2066.2) | 75.7 (52.9, 104.2) | -4.8 (-5.3, -4.2) |
| Lebanon | 3114.8 (1901.3, 4715.1) | 251.7 (151.5, 383.3) | 1896.2 (1141.4, 2906.8) | 56.6 (34.5, 86.3) | -5.0 (-5.4, -4.7) |
| Lesotho | 2106.2 (1349.7, 3045.7) | 447.6 (284.1, 650.8) | 4360.8 (2691.4, 6697.8) | 695.8 (426.3, 1067.6) | 2.3 (1.8, 2.9) |
| Liberia | 2739.5 (1793.7, 3986.8) | 413.9 (269.4, 603.5) | 4540.7 (2721.4, 7187.8) | 342.8 (204.1, 541.7) | -0.8 (-1.0, -0.7) |
| Libya | 1353.2 (769.1, 2131.1) | 118.7 (66.7, 188.6) | 3026.8 (1685.8, 5011.0) | 90.5 (49.4, 151.4) | -0.8 (-1.0, -0.6) |
| Lithuania | 2595.6 (1831.1, 3534.7) | 105.4 (74.2, 143.6) | 1648.2 (1110.7, 2278.4) | 57.6 (39.5, 78.9) | -2.4 (-2.7, -2.1) |
| Luxembourg | 283.2 (194.7, 386.4) | 94.1 (65.0, 128.1) | 146.3 (93.8, 206.8) | 24.4 (15.9, 34.3) | -4.5 (-4.7, -4.3) |
| Madagascar | 18707.4 (12216.1, 26567.2) | 617.3 (399.2, 882.1) | 33034.8 (19975.5, 50409.9) | 465.4 (279.7, 710.8) | -1.0 (-1.0, -0.9) |
| Malawi | 8517.3 (5439.7, 12374.6) | 377.4 (237.9, 552.2) | 14539.6 (8723.0, 21923.4) | 328.1 (194.9, 496.0) | -0.8 (-1.0, -0.5) |
| Malaysia | 27997.9 (19479.8, 38235.0) | 527.2 (366.5, 719.9) | 47908.1 (32321.2, 66576.4) | 292.6 (195.8, 408.4) | -1.8 (-2.0, -1.6) |
| Maldives | 475.9 (328.8, 661.5) | 853.8 (583.3, 1187.0) | 384.2 (242.6, 565.4) | 184.9 (117.3, 269.8) | -5.4 (-5.6, -5.2) |
| Mali | 9986.3 (6329.0, 14751.9) | 419.3 (261.0, 625.8) | 15201.4 (9349.8, 22967.2) | 285.6 (173.1, 434.2) | -1.2 (-1.3, -1.0) |
| Malta | 185.6 (128.2, 250.0) | 78.3 (53.7, 105.8) | 120.3 (77.4, 169.3) | 22.5 (14.8, 31.4) | -4.0 (-4.2, -3.9) |
| Marshall Islands | 80.6 (54.7, 114.6) | 827.4 (557.6, 1175.3) | 156.6 (99.7, 237.2) | 698.0 (437.2, 1061.2) | -0.5 (-0.6, -0.4) |
| Mauritania | 2883.5 (1878.6, 4259.7) | 500.6 (323.6, 743.7) | 3257.6 (1939.2, 5082.6) | 260.8 (154.4, 406.8) | -2.3 (-2.4, -2.1) |
| Mauritius | 2168.0 (1598.3, 2838.0) | 493.5 (361.7, 647.3) | 1978.5 (1449.6, 2586.0) | 195.4 (142.0, 256.7) | -4.1 (-4.7, -3.5) |
| Mexico | 24680.0 (18026.6, 32223.7) | 101.1 (72.9, 132.7) | 41685.3 (30021.2, 55406.5) | 57.8 (41.3, 77.2) | -2.2 (-2.3, -2.0) |
| Micronesia (Federated States of) | 256.1 (163.9, 372.1) | 893.8 (569.3, 1300.4) | 311.2 (200.7, 461.2) | 679.0 (432.0, 1014.5) | -0.9 (-1.0, -0.8) |
| Monaco | 24.6 (14.1, 37.4) | 65.0 (38.3, 97.4) | 14.1 (8.1, 21.8) | 27.3 (15.9, 42.0) | -2.8 (-3.0, -2.7) |
| Mongolia | 5819.3 (4079.4, 7864.1) | 997.7 (688.9, 1359.9) | 6912.0 (4857.9, 9298.9) | 515.1 (351.5, 703.6) | -2.5 (-3.1, -2.0) |
| Montenegro | 1343.7 (878.2, 1882.3) | 398.6 (258.4, 560.7) | 1898.0 (1184.9, 2733.1) | 368.7 (226.0, 534.6) | -0.6 (-0.8, -0.3) |
| Morocco | 17698.2 (10316.0, 27695.8) | 212.1 (122.6, 332.7) | 24932.5 (13867.1, 40376.9) | 125.6 (68.9, 203.7) | -1.7 (-1.7, -1.6) |
| Mozambique | 17121.7 (11025.9, 25021.3) | 487.5 (310.9, 716.9) | 36135.8 (21391.1, 55584.0) | 527.3 (311.0, 808.8) | 0.8 (0.6, 1.1) |
| Myanmar | 153099.0 (100411.0, 221102.3) | 1103.5 (719.2, 1592.1) | 165360.9 (104301.9, 243092.4) | 579.1 (362.2, 854.0) | -2.4 (-2.6, -2.2) |
| Namibia | 2006.5 (1345.0, 2813.1) | 538.2 (353.8, 766.5) | 2965.5 (1815.9, 4420.4) | 378.5 (230.6, 565.4) | -1.5 (-1.8, -1.1) |
| Nauru | 28.7 (18.2, 42.3) | 950.9 (602.8, 1396.4) | 32.2 (20.4, 48.2) | 848.8 (535.2, 1264.0) | -0.4 (-0.9, 0.0) |
| Nepal | 21264.2 (13626.9, 31206.4) | 389.6 (244.6, 578.4) | 31310.8 (18947.2, 47854.8) | 235.5 (140.5, 362.7) | -1.7 (-2.0, -1.4) |
| Netherlands | 5491.5 (3725.5, 7538.4) | 49.4 (33.7, 67.6) | 4276.5 (2734.6, 6055.0) | 21.3 (13.8, 30.0) | -3.2 (-3.5, -3.0) |
| New Zealand | 974.6 (660.5, 1334.7) | 44.6 (30.3, 61.0) | 923.0 (593.9, 1306.7) | 19.5 (12.7, 27.4) | -2.8 (-2.9, -2.7) |
| Nicaragua | 1530.5 (1061.3, 2093.8) | 171.3 (117.8, 235.2) | 2570.8 (1698.4, 3700.0) | 91.5 (60.3, 131.7) | -2.1 (-2.2, -1.9) |
| Niger | 6611.4 (4091.9, 9876.7) | 404.1 (244.7, 609.6) | 15753.2 (9105.1, 24893.9) | 328.4 (186.8, 521.3) | -0.6 (-0.7, -0.5) |
| Nigeria | 127268.4 (85165.5, 180200.7) | 506.7 (333.9, 723.7) | 125757.8 (83439.3, 183203.3) | 235.2 (154.2, 343.1) | -2.9 (-3.1, -2.7) |
| Niue | 6.2 (4.0, 9.2) | 527.1 (341.8, 777.9) | 4.6 (2.9, 6.7) | 383.8 (243.7, 563.3) | -1.3 (-1.4, -1.2) |
| North Macedonia | 3224.5 (2228.2, 4386.6) | 309.4 (210.5, 425.8) | 3252.1 (2050.7, 4795.1) | 180.3 (110.4, 269.5) | -2.1 (-2.4, -1.8) |
| Northern Mariana Islands | 55.5 (35.9, 81.1) | 453.8 (295.1, 657.5) | 88.6 (59.7, 123.7) | 288.3 (189.7, 408.0) | -1.5 (-1.8, -1.3) |
| Norway | 1456.8 (988.8, 1993.3) | 38.3 (26.4, 52.0) | 976.1 (618.9, 1389.8) | 16.8 (10.9, 23.7) | -2.9 (-3.1, -2.8) |
| Oman | 855.0 (492.5, 1360.1) | 203.3 (115.8, 321.6) | 998.0 (604.4, 1541.3) | 73.8 (44.8, 114.0) | -3.1 (-3.2, -2.9) |
| Pakistan | 77065.7 (50784.5, 109356.7) | 240.6 (157.2, 343.5) | 159010.7 (103414.6, 232575.1) | 217.0 (139.3, 318.5) | -0.8 (-1.1, -0.5) |
| Palau | 32.5 (21.2, 47.8) | 552.9 (359.1, 812.8) | 55.5 (35.6, 80.9) | 408.8 (258.6, 602.0) | -0.8 (-0.9, -0.8) |
| Palestine | 1062.0 (634.8, 1621.2) | 214.4 (127.0, 327.6) | 1414.6 (893.6, 2088.6) | 94.3 (58.5, 140.6) | -2.7 (-3.0, -2.4) |
| Panama | 1235.3 (879.3, 1643.1) | 147.4 (104.5, 196.3) | 1987.1 (1299.8, 2788.8) | 81.9 (53.5, 114.9) | -2.3 (-2.5, -2.1) |
| Papua New Guinea | 6843.4 (4280.8, 10409.2) | 645.8 (400.6, 980.8) | 15201.6 (9282.4, 23004.2) | 509.1 (309.9, 769.9) | -0.8 (-0.8, -0.7) |
| Paraguay | 2465.0 (1719.9, 3356.0) | 195.9 (135.9, 267.1) | 3749.1 (2399.7, 5484.2) | 113.4 (72.2, 166.6) | -1.8 (-1.9, -1.7) |
| Peru | 6761.3 (4423.0, 9774.0) | 96.2 (62.7, 139.4) | 7860.7 (4783.6, 12122.5) | 41.4 (25.2, 63.9) | -3.3 (-3.7, -2.8) |
| Philippines | 73991.2 (53954.8, 98610.3) | 417.4 (301.3, 558.7) | 209877.3 (150214.0, 282851.9) | 418.6 (297.0, 565.7) | 0.2 (0.0, 0.4) |
| Poland | 32965.1 (23656.3, 43375.5) | 137.6 (98.3, 181.4) | 14974.8 (10285.8, 20417.2) | 39.9 (27.8, 54.1) | -4.6 (-4.8, -4.4) |
| Portugal | 11943.1 (8260.7, 16065.2) | 154.5 (106.2, 208.6) | 4311.6 (2833.7, 6108.9) | 32.4 (22.0, 45.2) | -5.5 (-5.6, -5.3) |
| Puerto Rico | 1176.8 (827.1, 1591.1) | 59.0 (41.5, 79.8) | 1019.2 (661.4, 1451.8) | 29.2 (19.3, 41.3) | -2.9 (-3.2, -2.7) |
| Qatar | 166.3 (102.6, 251.0) | 240.4 (144.1, 364.2) | 412.6 (239.0, 659.9) | 61.2 (34.2, 98.4) | -4.9 (-5.6, -4.3) |
| Republic of Korea | 59578.3 (39694.0, 83180.5) | 369.6 (235.9, 526.4) | 20460.9 (12627.2, 30472.7) | 40.1 (24.7, 59.8) | -7.7 (-8.0, -7.4) |
| Republic of Moldova | 12015.1 (8807.3, 15777.0) | 481.8 (347.5, 638.3) | 6259.1 (4502.9, 8298.9) | 191.8 (137.9, 254.8) | -4.1 (-4.5, -3.6) |
| Romania | 37025.3 (26167.5, 50070.9) | 236.7 (165.0, 322.9) | 22344.0 (15110.4, 30577.6) | 113.9 (78.2, 154.8) | -3.6 (-4.0, -3.1) |
| Russian Federation | 188284.8 (135132.3, 249656.7) | 188.2 (134.1, 250.3) | 150985.7 (105609.1, 203955.5) | 121.3 (84.9, 163.9) | -2.4 (-3.1, -1.7) |
| Rwanda | 13502.3 (8518.2, 19958.4) | 794.1 (496.0, 1177.3) | 9582.8 (5561.5, 15015.2) | 262.1 (149.5, 414.2) | -4.8 (-5.3, -4.3) |
| Saint Kitts and Nevis | 80.4 (56.8, 109.4) | 405.1 (289.8, 546.6) | 71.7 (48.7, 97.1) | 171.7 (114.3, 234.8) | -3.1 (-3.4, -2.8) |
| Saint Lucia | 133.1 (94.6, 176.2) | 275.0 (194.6, 364.5) | 158.3 (106.1, 219.9) | 117.6 (78.5, 163.6) | -3.2 (-3.5, -2.9) |
| Saint Vincent and the Grenadines | 90.8 (63.7, 122.8) | 227.8 (159.9, 308.1) | 113.2 (79.2, 153.9) | 141.6 (98.6, 193.1) | -1.7 (-1.9, -1.6) |
| Samoa | 283.0 (184.2, 404.0) | 582.3 (375.4, 832.8) | 393.7 (257.1, 566.3) | 472.2 (305.6, 680.5) | -0.7 (-0.8, -0.5) |
| San Marino | 9.0 (5.5, 13.6) | 45.8 (28.0, 68.9) | 6.9 (3.7, 11.3) | 16.9 (9.2, 27.6) | -2.7 (-2.9, -2.4) |
| Sao,me and Principe | 125.1 (83.7, 178.0) | 343.0 (227.9, 489.9) | 205.3 (132.7, 298.9) | 297.5 (191.1, 433.3) | -0.7 (-0.9, -0.4) |
| Saudi Arabia | 10051.7 (6083.8, 15518.7) | 278.0 (166.6, 427.7) | 22245.0 (13708.9, 33826.5) | 147.9 (90.7, 224.7) | -2.1 (-2.2, -2.1) |
| Senegal | 7398.0 (4772.2, 10676.4) | 387.5 (248.2, 562.3) | 12800.5 (7714.1, 19381.4) | 280.8 (167.3, 426.4) | -1.0 (-1.1, -0.9) |
| Serbia | 11813.3 (7783.9, 16708.0) | 193.5 (124.8, 277.6) | 6882.8 (4321.2, 10435.5) | 77.0 (48.5, 116.3) | -3.7 (-4.1, -3.4) |
| Seychelles | 125.5 (86.4, 174.4) | 406.1 (280.2, 563.1) | 147.3 (100.0, 207.4) | 214.8 (144.5, 304.6) | -1.7 (-1.8, -1.5) |
| Sierra Leone | 4736.7 (2995.5, 6908.6) | 404.2 (255.1, 589.8) | 7999.4 (4905.0, 12121.9) | 350.9 (214.2, 532.5) | -0.2 (-0.4, 0.0) |
| Singapore | 2282.2 (1587.0, 3051.4) | 176.9 (122.0, 237.2) | 1543.8 (1028.8, 2159.6) | 32.4 (21.4, 45.5) | -5.4 (-5.6, -5.3) |
| Slovakia | 4833.5 (3259.8, 6771.0) | 146.3 (98.3, 205.0) | 2500.6 (1639.3, 3679.8) | 48.3 (31.5, 71.0) | -3.8 (-3.9, -3.6) |
| Slovenia | 1091.5 (763.6, 1485.0) | 79.9 (55.9, 108.5) | 448.3 (277.2, 647.4) | 17.9 (11.3, 25.6) | -5.5 (-5.9, -5.2) |
| Solomon Islands | 654.1 (380.5, 982.9) | 819.2 (479.7, 1227.6) | 1507.6 (965.8, 2245.0) | 726.7 (462.5, 1087.1) | -0.3 (-0.4, -0.2) |
| Somalia | 8508.1 (5004.7, 13425.2) | 577.0 (341.6, 899.3) | 14347.2 (7919.5, 23578.8) | 384.6 (212.6, 632.3) | -1.4 (-1.5, -1.3) |
| South Africa | 33521.9 (23528.2, 45188.7) | 265.1 (184.3, 358.8) | 68349.1 (50230.2, 90062.5) | 253.3 (184.1, 335.8) | -0.2 (-0.7, 0.3) |
| South Sudan | 6021.6 (3578.1, 9423.7) | 408.9 (241.6, 641.7) | 6703.8 (3794.9, 10853.4) | 291.1 (163.5, 470.3) | -1.4 (-1.7, -1.0) |
| Spain | 21830.7 (14776.0, 29854.5) | 72.6 (49.2, 99.2) | 11675.9 (7282.3, 16777.9) | 20.8 (13.6, 29.2) | -4.1 (-4.2, -4.0) |
| Sri Lanka | 20980.5 (14210.0, 29335.9) | 345.5 (231.4, 484.4) | 28214.3 (15534.3, 44252.0) | 187.1 (102.3, 294.5) | -1.8 (-2.0, -1.6) |
| Sudan | 21871.3 (12797.5, 33842.6) | 397.8 (231.2, 617.0) | 20287.0 (10791.1, 33247.9) | 165.4 (88.3, 271.6) | -3.1 (-3.3, -3.0) |
| Suriname | 405.7 (279.4, 554.1) | 274.9 (187.9, 377.3) | 737.1 (476.0, 1067.6) | 202.2 (129.6, 294.1) | -1.1 (-1.4, -0.8) |
| Sweden | 3810.3 (2497.4, 5354.6) | 45.6 (30.3, 63.7) | 2725.6 (1659.6, 3968.8) | 21.9 (13.7, 31.7) | -2.4 (-2.5, -2.3) |
| Switzerland | 2566.3 (1676.3, 3603.8) | 43.8 (29.2, 61.0) | 1401.8 (866.5, 2010.6) | 13.3 (8.5, 18.8) | -3.9 (-4.0, -3.9) |
| Syrian Arab Republic | 9287.0 (5856.8, 13666.3) | 283.4 (175.8, 420.0) | 11558.5 (7013.2, 17801.1) | 153.2 (90.9, 238.0) | -2.5 (-2.8, -2.2) |
| Taiwan (Province of China) | 26112.8 (19260.3, 33859.4) | 291.5 (210.3, 382.3) | 13552.6 (9463.6, 18254.8) | 60.6 (42.3, 81.7) | -5.1 (-5.5, -4.8) |
| Tajikistan | 5930.1 (4051.4, 8229.4) | 382.6 (258.7, 534.3) | 8504.0 (5580.1, 12171.6) | 260.9 (166.6, 378.0) | -1.7 (-2.0, -1.3) |
| Thailand | 69577.7 (47251.8, 97678.4) | 338.7 (227.6, 477.4) | 114211.3 (73034.2, 168765.6) | 197.2 (125.6, 292.1) | -2.3 (-2.6, -2.1) |
| Timor-Leste | 1102.8 (717.9, 1594.9) | 651.8 (419.9, 941.4) | 2722.5 (1641.3, 4098.1) | 554.7 (332.5, 838.3) | -0.4 (-0.6, -0.2) |
| Togo | 3243.9 (2126.2, 4674.7) | 431.6 (279.9, 628.5) | 8837.3 (5395.8, 13311.8) | 375.5 (226.3, 568.7) | -0.4 (-0.6, -0.3) |
| Tokelau | 4.4 (2.8, 6.6) | 592.7 (371.8, 885.9) | 2.9 (1.8, 4.3) | 355.1 (223.3, 530.7) | -1.8 (-1.8, -1.7) |
| Tonga | 80.7 (51.9, 117.4) | 255.1 (162.2, 373.8) | 91.9 (57.8, 137.3) | 205.1 (128.6, 306.7) | -0.5 (-0.6, -0.5) |
| Trinidad and,bago | 858.7 (612.9, 1145.5) | 182.2 (129.3, 243.4) | 997.1 (643.8, 1426.0) | 92.7 (59.6, 132.8) | -2.7 (-3.1, -2.4) |
| Tunisia | 4225.4 (2501.5, 6553.7) | 148.8 (86.4, 232.4) | 5670.0 (2949.6, 9606.2) | 76.3 (39.4, 129.7) | -2.5 (-2.5, -2.4) |
| Turkey | 52260.7 (33966.6, 75534.4) | 260.6 (167.5, 378.7) | 46995.0 (28983.7, 71011.0) | 90.1 (55.1, 136.7) | -3.6 (-3.9, -3.3) |
| Turkmenistan | 3758.4 (2757.1, 4978.3) | 332.1 (240.6, 443.6) | 9881.4 (6730.9, 14092.7) | 403.4 (271.3, 578.5) | 0.4 (-0.1, 0.9) |
| Tuvalu | 35.0 (23.5, 49.9) | 885.1 (586.3, 1274.4) | 33.0 (21.7, 47.8) | 546.7 (355.8, 797.3) | -1.5 (-1.6, -1.4) |
| Uganda | 14165.4 (8467.9, 21626.2) | 384.0 (228.4, 587.3) | 19173.5 (10900.5, 30044.4) | 221.8 (125.2, 348.3) | -2.6 (-2.9, -2.3) |
| Ukraine | 61216.0 (42746.3, 83843.3) | 154.8 (107.8, 212.3) | 45139.3 (28304.0, 66094.4) | 114.9 (72.0, 168.5) | -1.7 (-2.0, -1.3) |
| United Arab Emirates | 639.2 (376.2, 991.0) | 205.4 (120.2, 317.3) | 2098.2 (1281.5, 3146.0) | 86.5 (49.7, 132.5) | -1.7 (-2.1, -1.3) |
| United Kingdom | 23658.7 (16285.4, 31833.4) | 47.6 (33.3, 63.4) | 16976.1 (11004.1, 23570.9) | 23.0 (15.4, 31.4) | -2.4 (-2.6, -2.3) |
| United Republic of Tanzania | 19143.7 (11857.7, 28069.7) | 302.6 (185.3, 446.9) | 27830.3 (16111.4, 44117.8) | 189.2 (108.3, 301.0) | -1.7 (-1.7, -1.6) |
| United States of America | 78425.6 (54744.8, 104809.1) | 45.6 (32.2, 60.5) | 126979.3 (87975.6, 170569.0) | 40.5 (28.4, 54.1) | -0.5 (-0.7, -0.2) |
| United States Virgin Islands | 61.3 (39.1, 90.3) | 123.6 (78.0, 183.2) | 49.6 (30.7, 75.3) | 53.8 (33.2, 82.1) | -2.5 (-2.7, -2.4) |
| Uruguay | 2433.1 (1664.9, 3321.3) | 116.3 (79.7, 158.6) | 1328.3 (872.3, 1855.6) | 45.9 (30.7, 63.5) | -3.5 (-3.8, -3.3) |
| Uzbekistan | 27200.3 (20076.4, 34977.7) | 416.9 (305.9, 537.9) | 33903.5 (24192.2, 46008.2) | 223.2 (156.2, 305.3) | -2.4 (-2.9, -2.0) |
| Vanuatu | 271.8 (171.4, 405.4) | 709.5 (445.3, 1053.0) | 645.4 (418.4, 943.9) | 598.6 (384.7, 876.6) | -0.7 (-0.7, -0.6) |
| Venezuela (Bolivarian Republic of) | 11149.8 (8003.6, 14850.4) | 200.5 (142.8, 267.4) | 22364.6 (14389.6, 32056.0) | 131.3 (83.9, 188.7) | -2.2 (-2.5, -1.9) |
| Viet Nam | 136322.0 (85802.4, 206232.4) | 604.2 (378.4, 914.5) | 246791.7 (150576.9, 368270.8) | 430.7 (260.8, 644.4) | -0.8 (-0.9, -0.6) |
| Yemen | 12671.0 (7358.2, 20037.8) | 431.9 (248.0, 684.8) | 19366.8 (10879.0, 31624.5) | 225.7 (125.0, 370.2) | -2.5 (-2.7, -2.3) |
| Zambia | 7326.5 (4665.5, 10777.3) | 443.9 (279.5, 657.9) | 14542.5 (8547.5, 22796.5) | 354.0 (206.9, 553.3) | -1.2 (-1.4, -1.0) |
| Zimbabwe | 6779.2 (4514.4, 9633.1) | 298.1 (195.7, 427.3) | 19924.2 (12838.1, 29312.6) | 485.9 (310.8, 711.6) | 2.4 (1.7, 3.1) |

**Notes:** ASR: age-standardized rates; DALYs: disability-adjusted life years; EAPCs: estimated annual percentage changes.

**Table S3. National/territorial trends in stroke attributable kidney dysfunction burden: mortality, and DALYs (1990–2021).**

| **Location** | **1990** | | **2021** | | **EAPC (95%CI)** |
| --- | --- | --- | --- | --- | --- |
|  | **Number** | **ASR, per**  **100,000 persons** | **Number** | **ASR, per**  **100,000 persons** |  |
| **Deaths** |  |  |  |  |  |
| Afghanistan | 1583.5 (925.0, 2420.6) | 45.8 (26.0, 71.0) | 1463.3 (854.7, 2230.1) | 32.9 (18.7, 50.8) | -1.3 (-1.5, -1.1) |
| Albania | 251.7 (155.4, 365.9) | 27.6 (16.4, 40.8) | 451.1 (242.1, 695.1) | 19.6 (10.4, 30.3) | -1.0 (-1.2, -0.8) |
| Algeria | 1249.5 (717.1, 1941.0) | 27.1 (14.7, 42.9) | 2535.5 (1396.2, 3935.0) | 17.5 (9.3, 27.3) | -1.2 (-1.3, -1.2) |
| American Samoa | 2.8 (1.9, 3.9) | 27.0 (17.3, 38.3) | 5.0 (3.2, 7.2) | 21.0 (13.2, 30.7) | -1.0 (-1.1, -0.8) |
| Andorra | 1.5 (0.8, 2.4) | 5.6 (2.9, 9.2) | 2.6 (1.3, 4.4) | 2.8 (1.3, 4.6) | -2.0 (-2.3, -1.8) |
| Angola | 699.6 (446.2, 1008.4) | 40.4 (24.8, 59.3) | 1466.3 (901.0, 2171.8) | 29.6 (17.6, 44.7) | -1.3 (-1.4, -1.2) |
| Antigua and Barbuda | 5.6 (3.6, 7.8) | 17.8 (11.6, 24.5) | 5.6 (3.7, 7.8) | 10.4 (6.7, 14.7) | -2.1 (-2.3, -1.8) |
| Argentina | 2025.5 (1263.1, 2913.9) | 12.0 (7.3, 17.4) | 1431.6 (879.2, 2082.1) | 4.5 (2.8, 6.5) | -2.9 (-3.1, -2.8) |
| Armenia | 317.7 (212.1, 434.3) | 24.8 (16.2, 34.2) | 321.4 (199.7, 451.2) | 13.4 (8.4, 18.7) | -3.0 (-3.5, -2.6) |
| Australia | 923.6 (522.3, 1359.5) | 8.9 (4.9, 13.2) | 795.9 (422.3, 1213.3) | 2.7 (1.4, 4.0) | -4.1 (-4.2, -4.0) |
| Austria | 890.3 (505.4, 1308.6) | 12.5 (7.1, 18.3) | 348.1 (190.0, 518.2) | 2.8 (1.6, 4.2) | -5.2 (-5.5, -4.8) |
| Azerbaijan | 681.9 (458.7, 934.5) | 27.8 (18.3, 38.5) | 1012.9 (637.8, 1453.0) | 22.6 (13.8, 32.7) | -0.5 (-0.7, -0.2) |
| Bahamas | 10.3 (7.0, 14.3) | 13.2 (8.7, 18.3) | 16.3 (10.2, 23.6) | 8.0 (4.9, 11.8) | -1.7 (-1.8, -1.6) |
| Bahrain | 17.1 (11.1, 24.2) | 27.6 (16.6, 40.6) | 36.8 (22.3, 54.4) | 13.6 (7.6, 20.6) | -2.9 (-3.4, -2.5) |
| Bangladesh | 7840.3 (4973.0, 11301.1) | 34.3 (21.3, 49.8) | 16730.3 (9867.1, 25266.7) | 25.2 (14.5, 38.3) | -1.0 (-1.4, -0.7) |
| Barbados | 27.0 (16.9, 38.8) | 15.7 (9.9, 22.4) | 28.2 (16.7, 41.3) | 9.7 (5.7, 14.2) | -1.9 (-2.1, -1.6) |
| Belarus | 1795.1 (1177.7, 2496.5) | 25.9 (16.8, 36.1) | 1665.0 (998.0, 2425.0) | 18.3 (11.0, 26.6) | -1.9 (-2.3, -1.4) |
| Belgium | 1058.8 (607.8, 1550.4) | 11.7 (6.8, 17.2) | 559.3 (311.0, 837.6) | 3.4 (2.0, 5.1) | -3.8 (-3.9, -3.7) |
| Belize | 5.2 (3.5, 7.3) | 10.4 (6.9, 14.6) | 11.8 (7.8, 16.5) | 8.0 (5.1, 11.3) | -1.4 (-1.9, -0.8) |
| Benin | 352.9 (231.6, 494.8) | 35.4 (22.8, 50.0) | 653.1 (412.8, 947.5) | 27.7 (17.2, 40.5) | -0.8 (-0.9, -0.7) |
| Bermuda | 3.2 (2.0, 4.5) | 10.0 (6.3, 14.3) | 3.2 (1.9, 4.7) | 3.9 (2.3, 5.7) | -3.2 (-3.4, -3.1) |
| Bhutan | 21.1 (12.5, 33.0) | 19.8 (11.1, 31.5) | 40.9 (23.5, 64.0) | 13.3 (7.6, 20.9) | -1.3 (-1.4, -1.2) |
| Bolivia (Plurinational State of) | 236.3 (137.1, 367.3) | 15.0 (8.5, 23.5) | 357.3 (193.3, 566.8) | 8.0 (4.3, 12.8) | -2.0 (-2.2, -1.9) |
| Bosnia and Herzegovina | 400.1 (253.8, 572.3) | 22.2 (13.6, 32.2) | 543.0 (304.2, 815.0) | 15.2 (8.5, 22.8) | -1.6 (-1.7, -1.4) |
| Botswana | 100.5 (62.9, 144.5) | 40.3 (24.2, 58.9) | 149.1 (92.4, 222.9) | 23.4 (14.2, 35.4) | -1.7 (-1.9, -1.5) |
| Brazil | 9909.8 (6927.6, 13137.1) | 23.1 (15.5, 31.3) | 10769.9 (7242.2, 14584.1) | 8.0 (5.3, 11.0) | -3.4 (-3.4, -3.3) |
| Brunei Darussalam | 12.2 (7.6, 17.8) | 26.3 (15.8, 39.0) | 16.5 (9.9, 24.3) | 11.8 (6.6, 17.7) | -2.4 (-2.7, -2.2) |
| Bulgaria | 2278.9 (1480.4, 3126.2) | 43.0 (25.9, 60.8) | 2139.2 (1267.4, 3099.7) | 25.3 (15.0, 36.6) | -2.0 (-2.2, -1.8) |
| Burkina Faso | 437.4 (285.5, 629.8) | 21.2 (13.4, 31.0) | 841.2 (521.1, 1248.8) | 19.3 (11.7, 28.9) | -0.1 (-0.1, 0.0) |
| Burundi | 446.3 (256.5, 667.7) | 37.8 (21.3, 57.0) | 450.5 (264.7, 688.4) | 20.1 (11.4, 31.2) | -2.6 (-2.9, -2.3) |
| Cabo Verde | 22.5 (13.7, 33.1) | 17.4 (10.7, 25.5) | 43.3 (25.5, 65.6) | 19.0 (11.2, 28.9) | 0.0 (-0.4, 0.3) |
| Cambodia | 1034.2 (684.6, 1450.7) | 48.5 (31.1, 69.0) | 2087.0 (1295.7, 3070.6) | 37.0 (22.3, 54.6) | -1.0 (-1.1, -0.9) |
| Cameroon | 708.1 (450.1, 1028.4) | 33.8 (20.8, 49.6) | 1980.9 (1184.5, 3025.9) | 34.0 (20.1, 52.0) | 0.1 (-0.3, 0.5) |
| Canada | 1222.3 (708.1, 1777.2) | 6.9 (4.0, 10.1) | 1144.4 (628.3, 1733.7) | 2.5 (1.4, 3.7) | -3.5 (-3.7, -3.4) |
| Central African Republic | 263.3 (162.2, 387.9) | 50.6 (30.0, 75.4) | 403.8 (239.0, 618.0) | 42.5 (24.3, 65.4) | -0.6 (-0.7, -0.6) |
| Chad | 401.4 (252.6, 578.9) | 28.1 (17.2, 41.0) | 800.3 (490.0, 1188.5) | 29.6 (17.7, 44.3) | 0.1 (-0.1, 0.3) |
| Chile | 631.0 (391.5, 904.3) | 12.5 (7.5, 18.1) | 639.0 (373.8, 947.4) | 4.3 (2.6, 6.4) | -3.0 (-3.2, -2.8) |
| China | 126986.1 (83774.2, 181101.8) | 34.5 (21.4, 50.6) | 190713.6 (118059.8, 279641.1) | 17.7 (10.7, 26.2) | -1.9 (-2.2, -1.6) |
| Colombia | 1175.1 (796.3, 1588.4) | 13.9 (9.2, 19.0) | 1404.9 (872.8, 2030.8) | 4.7 (2.9, 6.7) | -4.2 (-4.4, -4.0) |
| Comoros | 24.7 (14.7, 36.8) | 26.2 (15.3, 39.3) | 37.4 (21.7, 58.2) | 15.9 (9.0, 24.9) | -1.9 (-2.2, -1.7) |
| Congo | 238.6 (150.9, 347.2) | 48.3 (29.3, 71.0) | 374.6 (228.3, 561.8) | 32.2 (19.2, 48.6) | -1.6 (-1.7, -1.5) |
| Cook Islands | 1.3 (0.8, 1.9) | 21.5 (13.2, 31.4) | 1.5 (0.9, 2.3) | 10.6 (6.4, 15.9) | -2.3 (-2.5, -2.1) |
| Costa Rica | 91.7 (59.7, 126.6) | 10.2 (6.6, 14.1) | 162.9 (100.9, 231.3) | 5.4 (3.4, 7.7) | -2.5 (-2.9, -2.1) |
| Coted'Ivoire | 542.0 (347.2, 792.5) | 30.7 (19.1, 45.2) | 1333.9 (814.3, 2028.8) | 26.5 (15.9, 40.4) | -0.5 (-0.7, -0.4) |
| Croatia | 776.6 (483.7, 1107.8) | 26.7 (16.3, 38.3) | 489.4 (276.3, 726.3) | 8.5 (4.9, 12.6) | -3.9 (-4.1, -3.8) |
| Cuba | 573.6 (369.7, 807.6) | 10.4 (6.7, 14.7) | 815.8 (507.0, 1174.3) | 7.3 (4.5, 10.4) | -1.4 (-1.5, -1.2) |
| Cyprus | 73.0 (39.4, 112.2) | 26.2 (13.1, 41.2) | 64.7 (34.2, 101.4) | 6.3 (3.3, 10.0) | -4.9 (-5.3, -4.6) |
| Czechia | 1997.2 (1218.5, 2849.6) | 26.4 (15.9, 37.8) | 663.7 (377.8, 982.0) | 5.1 (2.9, 7.5) | -5.6 (-6.0, -5.2) |
| Democratic People's Republic of Korea | 2350.8 (1458.5, 3422.0) | 31.6 (19.0, 46.8) | 4706.8 (2870.5, 6961.1) | 27.2 (16.4, 40.6) | -0.5 (-0.7, -0.3) |
| Democratic Republic of the Congo | 2358.1 (1436.1, 3463.3) | 34.2 (20.0, 51.0) | 4790.4 (2759.1, 7466.6) | 30.5 (17.0, 48.3) | -0.5 (-0.6, -0.4) |
| Denmark | 507.5 (297.3, 738.8) | 10.2 (6.0, 14.7) | 308.2 (175.0, 452.7) | 4.1 (2.3, 6.0) | -3.3 (-3.5, -3.1) |
| Djibouti | 13.1 (7.8, 20.6) | 22.0 (12.8, 34.8) | 46.3 (26.5, 75.0) | 16.9 (9.4, 27.5) | -1.0 (-1.1, -1.0) |
| Dominica | 6.0 (3.8, 8.6) | 18.3 (11.6, 26.4) | 6.1 (3.7, 9.0) | 14.0 (8.3, 21.0) | -0.9 (-1.1, -0.8) |
| Dominican Republic | 223.9 (142.9, 321.3) | 12.6 (7.8, 18.2) | 570.1 (343.5, 875.3) | 10.5 (6.3, 16.2) | -0.1 (-0.3, 0.1) |
| Ecuador | 241.3 (161.0, 333.4) | 8.9 (5.8, 12.3) | 375.8 (223.0, 566.7) | 4.4 (2.6, 6.6) | -2.2 (-2.5, -2.0) |
| Egypt | 4866.0 (3066.6, 7083.2) | 44.7 (26.6, 67.3) | 7836.6 (4938.2, 11410.6) | 31.1 (18.5, 46.2) | -0.8 (-0.9, -0.6) |
| El Salvador | 161.3 (104.5, 226.0) | 10.3 (6.6, 14.4) | 196.0 (115.8, 293.4) | 5.6 (3.3, 8.3) | -2.0 (-2.3, -1.7) |
| Equatorial Guinea | 42.2 (26.4, 62.2) | 46.2 (28.0, 69.1) | 50.5 (27.4, 82.2) | 23.4 (12.6, 37.7) | -2.6 (-3.0, -2.3) |
| Eritrea | 171.5 (107.0, 256.2) | 33.0 (19.8, 49.8) | 268.3 (157.4, 417.9) | 22.0 (12.5, 34.6) | -1.3 (-1.4, -1.2) |
| Estonia | 368.9 (235.2, 517.4) | 33.1 (21.0, 46.5) | 113.4 (67.1, 165.3) | 6.4 (3.9, 9.2) | -6.8 (-7.4, -6.2) |
| Eswatini | 50.2 (31.3, 72.7) | 39.0 (23.5, 57.5) | 92.3 (52.5, 147.6) | 36.7 (20.4, 58.3) | 0.3 (-0.3, 0.8) |
| Ethiopia | 2294.1 (1499.7, 3428.5) | 23.5 (14.7, 35.3) | 2324.3 (1450.2, 3460.6) | 11.1 (6.8, 16.8) | -2.8 (-3.0, -2.7) |
| Fiji | 48.2 (31.5, 69.6) | 29.1 (18.3, 42.5) | 81.8 (51.5, 120.3) | 23.4 (14.2, 34.7) | -0.9 (-1.1, -0.8) |
| Finland | 427.0 (256.2, 623.7) | 10.4 (6.2, 15.2) | 317.5 (176.2, 479.9) | 3.6 (2.1, 5.4) | -3.3 (-3.4, -3.2) |
| France | 3768.0 (2043.2, 5619.9) | 7.4 (4.1, 11.0) | 2683.4 (1409.4, 4080.2) | 2.5 (1.4, 3.7) | -3.9 (-4.0, -3.7) |
| Gabon | 99.9 (61.4, 147.7) | 35.1 (21.0, 52.7) | 118.2 (70.1, 181.2) | 25.4 (14.6, 39.0) | -1.2 (-1.3, -1.0) |
| Gambia | 49.8 (30.8, 73.3) | 30.3 (18.2, 45.0) | 148.8 (89.5, 222.7) | 32.0 (19.0, 48.1) | 0.1 (0.0, 0.2) |
| Georgia | 1342.1 (901.1, 1815.6) | 41.1 (27.2, 56.0) | 1144.4 (714.0, 1621.5) | 32.8 (20.8, 46.1) | -1.0 (-1.4, -0.6) |
| Germany | 9193.7 (5157.2, 13659.1) | 12.2 (6.9, 18.1) | 4691.8 (2564.5, 7098.9) | 3.5 (2.0, 5.2) | -4.2 (-4.5, -3.9) |
| Ghana | 887.3 (554.1, 1295.8) | 30.0 (18.2, 44.4) | 2116.6 (1279.5, 3181.9) | 27.3 (16.0, 41.5) | 0.0 (-0.3, 0.3) |
| Greece | 1900.3 (1096.1, 2754.3) | 23.4 (13.3, 34.1) | 1332.2 (718.8, 1993.7) | 7.1 (4.0, 10.4) | -4.5 (-4.9, -4.2) |
| Greenland | 3.1 (1.9, 4.6) | 26.1 (15.1, 39.1) | 2.8 (1.7, 4.3) | 9.9 (5.6, 15.4) | -3.2 (-3.4, -3.1) |
| Grenada | 10.4 (6.7, 14.6) | 24.7 (16.3, 34.1) | 7.4 (4.7, 10.4) | 13.3 (8.2, 19.0) | -2.1 (-2.2, -1.9) |
| Guam | 4.8 (3.2, 6.8) | 15.6 (9.8, 22.4) | 7.6 (5.0, 10.8) | 6.6 (4.3, 9.4) | -2.4 (-2.8, -2.0) |
| Guatemala | 196.7 (136.2, 265.5) | 12.7 (8.3, 17.5) | 465.9 (296.6, 652.6) | 8.3 (5.2, 11.6) | -2.1 (-2.4, -1.7) |
| Guinea | 494.0 (315.4, 716.7) | 29.6 (18.4, 43.5) | 808.0 (496.8, 1203.4) | 29.6 (17.8, 44.2) | 0.3 (0.1, 0.4) |
| Guinea-Bissau | 97.6 (62.6, 143.0) | 49.9 (31.2, 73.6) | 140.7 (88.8, 206.1) | 43.0 (26.5, 63.4) | -0.3 (-0.4, -0.3) |
| Guyana | 83.0 (58.0, 113.0) | 42.9 (29.3, 58.9) | 67.2 (42.9, 97.4) | 21.2 (13.2, 31.0) | -1.9 (-2.1, -1.7) |
| Haiti | 607.9 (388.5, 864.6) | 40.0 (24.6, 58.3) | 926.3 (543.6, 1443.6) | 28.2 (16.0, 44.3) | -1.0 (-1.1, -0.9) |
| Honduras | 179.2 (115.1, 255.6) | 17.7 (11.1, 25.4) | 693.2 (426.1, 1020.9) | 22.8 (13.7, 33.8) | 1.0 (0.8, 1.2) |
| Hungary | 2015.6 (1263.7, 2856.8) | 26.0 (16.0, 37.1) | 863.9 (504.9, 1271.9) | 7.3 (4.3, 10.6) | -4.5 (-4.6, -4.3) |
| Iceland | 11.5 (6.6, 17.0) | 6.8 (3.9, 10.0) | 8.6 (4.6, 13.1) | 2.2 (1.2, 3.4) | -3.7 (-3.9, -3.6) |
| India | 41183.4 (28199.7, 55753.5) | 18.8 (12.2, 25.9) | 83759.2 (55852.6, 115735.6) | 14.0 (9.0, 19.5) | -1.0 (-1.1, -0.9) |
| Indonesia | 21063.9 (14496.9, 28780.8) | 44.6 (29.6, 62.3) | 52022.0 (34083.6, 71714.6) | 47.4 (30.1, 66.2) | 0.3 (0.1, 0.4) |
| Iran (Islamic Republic of) | 2424.5 (1655.7, 3324.0) | 22.3 (14.0, 31.9) | 4202.8 (2624.7, 5993.7) | 11.3 (6.8, 16.3) | -2.3 (-2.4, -2.1) |
| Iraq | 1562.1 (967.6, 2267.8) | 38.1 (23.3, 55.4) | 3314.4 (1959.6, 5003.6) | 31.3 (17.9, 47.6) | -1.3 (-1.5, -1.1) |
| Ireland | 334.7 (212.8, 461.6) | 15.0 (9.4, 20.9) | 174.4 (104.8, 248.4) | 3.6 (2.2, 5.2) | -4.4 (-4.6, -4.2) |
| Israel | 247.1 (153.2, 351.4) | 9.6 (5.8, 13.7) | 217.9 (123.2, 324.9) | 2.9 (1.7, 4.2) | -4.4 (-4.6, -4.2) |
| Italy | 6115.0 (3608.3, 8902.6) | 12.3 (7.2, 18.0) | 3952.5 (2105.5, 6068.1) | 3.5 (2.0, 5.3) | -4.0 (-4.2, -3.9) |
| Jamaica | 172.2 (112.1, 241.4) | 17.0 (11.1, 23.7) | 213.6 (126.7, 312.3) | 12.5 (7.4, 18.2) | -0.8 (-1.2, -0.3) |
| Japan | 12259.6 (6937.0, 18025.0) | 14.1 (7.8, 20.9) | 11201.5 (5760.4, 17209.2) | 4.2 (2.4, 6.2) | -4.1 (-4.2, -3.9) |
| Jordan | 155.5 (98.6, 226.9) | 27.0 (16.5, 40.0) | 358.4 (215.3, 537.7) | 11.8 (6.8, 18.0) | -3.2 (-3.6, -2.9) |
| Kazakhstan | 2418.1 (1628.5, 3337.7) | 39.0 (25.6, 54.4) | 2726.6 (1763.7, 3803.7) | 33.9 (21.1, 47.8) | -1.0 (-1.5, -0.6) |
| Kenya | 543.6 (335.7, 819.5) | 14.1 (8.5, 21.5) | 1453.1 (882.7, 2172.2) | 14.4 (8.4, 22.1) | 0.3 (0.2, 0.5) |
| Kiribati | 6.8 (4.5, 9.6) | 36.8 (23.5, 53.1) | 11.9 (7.7, 17.6) | 33.9 (21.2, 50.5) | -0.3 (-0.3, -0.2) |
| Kuwait | 22.3 (14.5, 31.2) | 8.6 (5.3, 12.3) | 70.7 (41.6, 106.5) | 5.6 (3.2, 8.6) | -1.2 (-2.2, -0.2) |
| Kyrgyzstan | 642.6 (437.6, 866.2) | 42.2 (28.3, 57.3) | 451.1 (299.8, 613.1) | 18.9 (12.2, 26.2) | -3.2 (-3.7, -2.7) |
| Lao People's Democratic Republic | 706.7 (452.8, 1011.2) | 67.9 (42.4, 98.1) | 895.6 (565.2, 1314.1) | 40.5 (25.0, 59.8) | -1.9 (-2.0, -1.8) |
| Latvia | 753.0 (473.3, 1065.0) | 38.3 (24.0, 54.2) | 521.0 (309.9, 753.8) | 19.8 (12.2, 28.4) | -2.7 (-3.1, -2.4) |
| Lebanon | 242.2 (142.7, 366.7) | 23.4 (13.4, 35.9) | 259.2 (144.9, 401.6) | 7.2 (4.1, 11.1) | -4.0 (-4.3, -3.7) |
| Lesotho | 130.7 (82.1, 190.5) | 31.4 (19.1, 46.7) | 246.2 (149.7, 371.6) | 48.2 (28.5, 73.3) | 2.3 (1.8, 2.8) |
| Liberia | 174.1 (114.2, 251.2) | 30.8 (19.7, 44.9) | 265.2 (159.4, 408.6) | 27.7 (16.4, 42.6) | -0.5 (-0.6, -0.4) |
| Libya | 128.0 (73.1, 200.7) | 13.9 (7.8, 22.0) | 341.0 (190.5, 547.0) | 13.9 (7.6, 22.5) | 0.4 (0.2, 0.6) |
| Lithuania | 474.0 (305.3, 660.7) | 19.1 (12.3, 26.5) | 471.6 (277.9, 686.3) | 12.5 (7.6, 17.9) | -1.7 (-2.0, -1.3) |
| Luxembourg | 57.6 (34.5, 82.8) | 19.0 (11.3, 27.4) | 24.6 (14.1, 36.3) | 3.6 (2.1, 5.3) | -5.2 (-5.3, -5.1) |
| Madagascar | 869.1 (554.6, 1248.4) | 34.6 (21.4, 50.5) | 1454.6 (875.0, 2215.9) | 28.4 (16.5, 44.0) | -0.7 (-0.8, -0.6) |
| Malawi | 429.1 (266.8, 624.4) | 23.8 (14.3, 35.4) | 792.1 (466.5, 1202.8) | 23.2 (13.2, 35.9) | -0.4 (-0.6, -0.2) |
| Malaysia | 1572.9 (1077.6, 2169.3) | 33.8 (23.0, 46.9) | 3071.0 (2022.0, 4302.9) | 21.6 (13.9, 30.7) | -1.3 (-1.4, -1.2) |
| Maldives | 21.2 (14.6, 29.3) | 50.1 (33.1, 70.4) | 25.2 (15.2, 37.3) | 16.1 (9.7, 23.8) | -4.1 (-4.2, -3.9) |
| Mali | 504.2 (323.2, 723.3) | 26.8 (16.5, 39.2) | 870.7 (536.1, 1292.6) | 21.3 (12.8, 31.9) | -0.6 (-0.7, -0.5) |
| Malta | 29.7 (18.3, 42.2) | 13.4 (8.1, 19.3) | 20.7 (11.6, 31.2) | 3.3 (1.9, 5.0) | -4.6 (-4.8, -4.3) |
| Marshall Islands | 3.5 (2.3, 4.9) | 43.8 (28.3, 63.0) | 6.0 (3.8, 9.0) | 36.5 (22.0, 54.8) | -0.5 (-0.6, -0.4) |
| Mauritania | 180.7 (115.8, 265.0) | 36.2 (22.6, 53.9) | 243.6 (141.3, 377.3) | 23.6 (13.4, 36.6) | -1.5 (-1.7, -1.3) |
| Mauritius | 150.2 (106.8, 199.9) | 40.7 (28.2, 55.0) | 145.1 (100.5, 195.2) | 15.0 (10.2, 20.3) | -4.5 (-5.0, -3.9) |
| Mexico | 2371.0 (1557.9, 3255.7) | 12.3 (7.8, 17.0) | 3704.7 (2424.1, 5133.6) | 5.7 (3.7, 8.0) | -2.6 (-2.7, -2.4) |
| Micronesia (Federated States of) | 11.4 (7.2, 16.4) | 45.9 (28.4, 67.1) | 12.9 (8.2, 19.2) | 35.8 (21.9, 54.1) | -0.8 (-0.9, -0.8) |
| Monaco | 6.9 (3.6, 10.9) | 14.5 (7.8, 22.7) | 4.0 (2.1, 6.2) | 5.8 (3.1, 9.1) | -3.1 (-3.3, -2.9) |
| Mongolia | 254.6 (170.8, 353.8) | 48.3 (31.4, 68.2) | 298.4 (197.9, 414.7) | 27.6 (17.4, 39.3) | -2.2 (-2.7, -1.7) |
| Montenegro | 83.2 (50.7, 121.4) | 26.1 (15.7, 38.2) | 151.4 (88.8, 223.1) | 31.9 (18.2, 47.2) | 0.5 (0.2, 0.8) |
| Morocco | 1701.6 (1000.5, 2619.0) | 23.9 (13.8, 37.2) | 3342.5 (1869.1, 5293.2) | 19.9 (10.9, 31.7) | -0.5 (-0.5, -0.4) |
| Mozambique | 874.0 (552.4, 1283.0) | 30.3 (18.5, 45.2) | 1828.7 (1067.1, 2784.3) | 34.1 (19.2, 52.6) | 0.9 (0.7, 1.1) |
| Myanmar | 7393.5 (4793.5, 10580.1) | 63.4 (40.3, 91.4) | 9368.2 (5824.4, 13864.2) | 38.4 (23.4, 57.3) | -1.9 (-2.0, -1.8) |
| Namibia | 120.2 (79.6, 166.2) | 41.3 (25.9, 58.7) | 202.3 (124.0, 300.2) | 33.2 (19.8, 49.8) | -1.0 (-1.2, -0.7) |
| Nauru | 1.1 (0.7, 1.7) | 50.6 (31.2, 74.2) | 1.3 (0.8, 2.0) | 45.6 (27.9, 68.5) | -0.4 (-0.8, -0.1) |
| Nepal | 1235.6 (772.5, 1802.3) | 28.5 (17.0, 42.5) | 2223.8 (1315.3, 3405.5) | 19.5 (11.2, 30.3) | -1.3 (-1.6, -1.1) |
| Netherlands | 1099.7 (651.4, 1597.4) | 9.5 (5.6, 13.7) | 931.6 (529.7, 1374.0) | 4.1 (2.4, 6.1) | -3.2 (-3.5, -2.9) |
| New Zealand | 191.9 (111.4, 280.6) | 9.0 (5.1, 13.2) | 193.5 (104.1, 291.6) | 3.7 (2.0, 5.6) | -3.1 (-3.2, -2.9) |
| Nicaragua | 102.4 (67.0, 144.2) | 13.7 (8.7, 19.5) | 188.9 (118.3, 275.4) | 7.5 (4.7, 11.0) | -1.9 (-2.1, -1.7) |
| Niger | 330.6 (206.9, 481.1) | 26.6 (16.0, 39.3) | 880.5 (527.1, 1348.5) | 23.9 (13.9, 36.9) | -0.2 (-0.3, -0.1) |
| Nigeria | 8211.5 (5382.2, 11475.2) | 38.6 (24.6, 54.7) | 9393.4 (6077.9, 13540.9) | 23.3 (14.7, 33.8) | -1.9 (-2.0, -1.7) |
| Niue | 0.4 (0.3, 0.6) | 31.7 (19.8, 47.0) | 0.3 (0.2, 0.4) | 24.2 (14.8, 35.8) | -1.1 (-1.2, -1.0) |
| North Macedonia | 414.1 (261.6, 581.5) | 46.7 (28.6, 66.6) | 619.9 (360.9, 922.9) | 45.5 (25.0, 69.0) | -0.4 (-0.9, 0.1) |
| Northern Mariana Islands | 2.1 (1.3, 3.0) | 27.0 (16.7, 39.8) | 4.2 (2.8, 6.0) | 17.9 (11.2, 25.8) | -1.5 (-1.7, -1.3) |
| Norway | 408.6 (240.3, 596.4) | 9.4 (5.6, 13.7) | 184.8 (102.6, 278.8) | 2.8 (1.6, 4.2) | -4.2 (-4.3, -4.0) |
| Oman | 70.1 (40.3, 108.7) | 22.1 (12.4, 34.3) | 101.8 (61.2, 154.9) | 13.1 (7.6, 20.1) | -1.2 (-1.5, -0.9) |
| Pakistan | 5055.3 (3161.5, 7406.8) | 18.4 (11.2, 27.4) | 9964.7 (6294.4, 14686.8) | 17.9 (10.9, 26.8) | -0.5 (-0.7, -0.3) |
| Palau | 1.6 (1.0, 2.3) | 32.4 (20.0, 47.7) | 2.7 (1.7, 4.0) | 25.3 (15.3, 37.8) | -0.6 (-0.7, -0.5) |
| Palestine | 137.1 (80.0, 207.8) | 33.9 (19.3, 51.9) | 194.6 (119.8, 285.6) | 18.9 (11.2, 28.2) | -1.9 (-2.2, -1.6) |
| Panama | 102.0 (67.8, 140.7) | 13.5 (8.8, 18.7) | 193.0 (115.8, 279.7) | 7.9 (4.7, 11.4) | -2.0 (-2.2, -1.8) |
| Papua New Guinea | 282.6 (177.1, 422.3) | 34.6 (20.9, 52.5) | 649.2 (396.6, 976.4) | 28.7 (17.0, 43.5) | -0.6 (-0.7, -0.6) |
| Paraguay | 195.9 (127.3, 274.0) | 17.5 (11.2, 24.7) | 327.7 (198.3, 484.3) | 11.1 (6.6, 16.5) | -1.3 (-1.5, -1.2) |
| Peru | 427.8 (265.5, 633.4) | 7.0 (4.3, 10.4) | 619.1 (353.5, 964.5) | 3.4 (1.9, 5.3) | -2.9 (-3.4, -2.4) |
| Philippines | 3830.5 (2679.6, 5209.7) | 28.6 (19.3, 39.5) | 10651.5 (7353.8, 14613.6) | 25.5 (17.2, 35.6) | -0.2 (-0.3, 0.0) |
| Poland | 5623.9 (3502.3, 7914.0) | 25.2 (15.4, 35.6) | 3489.3 (2059.5, 5098.8) | 8.0 (4.8, 11.6) | -4.3 (-4.4, -4.1) |
| Portugal | 2031.0 (1238.6, 2889.3) | 28.8 (17.1, 41.4) | 849.3 (480.8, 1280.9) | 4.8 (2.8, 7.1) | -6.2 (-6.4, -6.1) |
| Puerto Rico | 127.5 (81.1, 181.1) | 6.7 (4.2, 9.6) | 112.6 (66.0, 166.8) | 2.5 (1.5, 3.7) | -3.6 (-3.8, -3.4) |
| Qatar | 9.2 (5.5, 13.9) | 26.7 (15.2, 40.9) | 23.0 (12.9, 37.1) | 8.9 (4.8, 14.3) | -4.2 (-4.9, -3.4) |
| Republic of Korea | 4458.9 (2772.4, 6408.8) | 36.3 (20.9, 53.9) | 2803.5 (1456.6, 4416.9) | 5.5 (2.8, 8.6) | -6.7 (-6.9, -6.4) |
| Republic of Moldova | 812.6 (560.3, 1094.5) | 38.3 (25.3, 52.7) | 659.7 (441.7, 903.6) | 19.8 (13.3, 27.2) | -2.6 (-2.8, -2.3) |
| Romania | 4274.7 (2706.3, 6063.7) | 32.7 (19.8, 47.2) | 3947.8 (2251.3, 5836.3) | 17.2 (10.0, 25.2) | -2.8 (-3.1, -2.5) |
| Russian Federation | 35576.3 (22990.1, 49492.1) | 40.2 (25.4, 56.6) | 29564.3 (18312.2, 42037.9) | 22.1 (13.7, 31.4) | -2.8 (-3.4, -2.2) |
| Rwanda | 600.1 (373.2, 886.4) | 43.3 (26.0, 65.1) | 506.4 (287.9, 792.0) | 17.9 (9.8, 28.5) | -3.9 (-4.4, -3.5) |
| Saint Kitts and Nevis | 7.5 (4.9, 10.3) | 36.1 (23.4, 50.4) | 5.6 (3.6, 7.7) | 17.6 (10.9, 25.0) | -2.4 (-2.6, -2.1) |
| Saint Lucia | 12.1 (7.9, 16.8) | 28.8 (18.2, 40.4) | 15.8 (9.6, 22.8) | 12.2 (7.4, 17.6) | -3.5 (-3.9, -3.1) |
| Saint Vincent and the Grenadines | 7.7 (5.0, 10.8) | 20.5 (13.0, 28.8) | 9.5 (6.1, 13.3) | 12.7 (8.1, 17.9) | -1.5 (-1.7, -1.3) |
| Samoa | 13.8 (8.7, 19.7) | 33.2 (20.6, 48.1) | 19.3 (12.2, 27.9) | 26.7 (16.5, 38.8) | -0.7 (-0.8, -0.6) |
| San Marino | 1.9 (1.1, 2.9) | 9.4 (5.1, 14.3) | 1.5 (0.8, 2.5) | 2.8 (1.4, 4.7) | -3.3 (-3.6, -2.9) |
| Sao,me and Principe | 8.6 (5.6, 12.3) | 26.3 (16.8, 37.9) | 13.8 (8.8, 20.0) | 26.3 (16.5, 38.3) | 0.0 (-0.1, 0.2) |
| Saudi Arabia | 784.5 (466.0, 1195.6) | 29.3 (16.9, 45.0) | 1460.8 (904.8, 2186.9) | 17.9 (10.5, 27.3) | -1.8 (-1.9, -1.7) |
| Senegal | 463.2 (295.5, 677.2) | 29.2 (18.2, 43.3) | 888.3 (533.3, 1350.4) | 24.1 (14.2, 36.9) | -0.6 (-0.7, -0.6) |
| Serbia | 1706.8 (1052.2, 2459.4) | 36.1 (21.7, 52.8) | 1746.7 (1031.7, 2612.0) | 18.3 (10.8, 27.4) | -2.8 (-3.0, -2.5) |
| Seychelles | 8.9 (6.0, 12.5) | 28.7 (19.2, 40.2) | 10.3 (6.7, 14.4) | 17.6 (11.3, 24.9) | -1.2 (-1.4, -1.0) |
| Sierra Leone | 328.7 (206.4, 478.9) | 32.1 (19.8, 47.1) | 524.8 (318.6, 784.0) | 29.1 (17.4, 43.6) | -0.1 (-0.3, 0.1) |
| Singapore | 197.7 (130.3, 270.5) | 18.7 (11.9, 26.0) | 111.2 (67.1, 162.9) | 2.4 (1.5, 3.6) | -6.4 (-6.7, -6.1) |
| Slovakia | 641.9 (393.4, 915.7) | 20.1 (12.1, 28.7) | 465.9 (273.4, 695.5) | 8.7 (5.1, 13.1) | -2.8 (-2.9, -2.7) |
| Slovenia | 202.3 (123.8, 293.1) | 14.9 (9.1, 21.6) | 126.4 (68.7, 191.9) | 4.2 (2.3, 6.3) | -4.3 (-4.4, -4.1) |
| Solomon Islands | 26.7 (15.9, 39.7) | 43.4 (25.6, 64.8) | 62.7 (39.8, 93.3) | 39.0 (24.2, 58.5) | -0.3 (-0.4, -0.2) |
| Somalia | 332.5 (194.7, 519.1) | 30.1 (17.4, 46.9) | 596.8 (334.2, 960.0) | 21.6 (11.8, 35.0) | -1.1 (-1.2, -1.0) |
| South Africa | 1984.5 (1285.6, 2789.0) | 19.0 (12.1, 27.1) | 5036.9 (3451.6, 6890.7) | 22.9 (15.2, 31.7) | 0.6 (0.0, 1.1) |
| South Sudan | 308.3 (180.0, 477.2) | 23.9 (13.7, 37.1) | 320.3 (181.1, 518.6) | 18.1 (10.0, 29.4) | -1.1 (-1.4, -0.8) |
| Spain | 4118.2 (2335.0, 6081.6) | 13.7 (7.7, 20.4) | 2109.9 (1121.6, 3221.8) | 2.9 (1.6, 4.3) | -5.0 (-5.2, -4.9) |
| Sri Lanka | 1668.7 (1098.3, 2325.4) | 35.7 (22.8, 50.4) | 2973.0 (1626.9, 4574.0) | 22.3 (12.1, 34.4) | -0.9 (-1.2, -0.7) |
| Sudan | 1561.1 (935.2, 2359.0) | 34.2 (19.9, 52.3) | 1947.7 (1089.3, 3057.9) | 21.4 (11.8, 33.7) | -1.7 (-1.8, -1.7) |
| Suriname | 25.0 (16.4, 35.2) | 19.2 (12.4, 27.3) | 47.1 (28.5, 70.2) | 14.1 (8.4, 21.0) | -1.0 (-1.3, -0.7) |
| Sweden | 898.2 (529.8, 1318.6) | 9.3 (5.5, 13.6) | 534.5 (296.5, 811.4) | 3.6 (2.0, 5.4) | -3.2 (-3.3, -3.0) |
| Switzerland | 607.1 (344.4, 900.6) | 9.2 (5.3, 13.6) | 322.5 (171.7, 489.5) | 2.5 (1.4, 3.8) | -4.1 (-4.2, -4.0) |
| Syrian Arab Republic | 641.8 (393.7, 948.3) | 25.6 (15.3, 38.3) | 1061.9 (613.1, 1645.7) | 18.1 (10.0, 28.2) | -1.7 (-1.9, -1.4) |
| Taiwan (Province of China) | 1674.4 (1173.2, 2233.5) | 22.3 (14.8, 30.5) | 1047.1 (652.1, 1493.9) | 4.4 (2.8, 6.3) | -5.1 (-5.4, -4.8) |
| Tajikistan | 460.8 (304.8, 651.2) | 33.3 (21.7, 47.4) | 650.8 (413.0, 942.1) | 26.8 (16.4, 39.4) | -1.0 (-1.5, -0.6) |
| Thailand | 4014.8 (2655.0, 5653.9) | 24.0 (15.5, 34.2) | 8212.0 (5000.2, 12154.6) | 13.9 (8.4, 20.5) | -2.4 (-2.7, -2.2) |
| Timor-Leste | 49.4 (31.8, 71.5) | 39.9 (25.0, 58.2) | 162.9 (99.2, 241.7) | 37.8 (22.6, 56.6) | 0.0 (-0.2, 0.2) |
| Togo | 184.5 (120.8, 262.9) | 32.1 (20.4, 46.3) | 515.6 (317.0, 765.1) | 30.2 (18.1, 45.1) | -0.2 (-0.4, -0.1) |
| Tokelau | 0.3 (0.2, 0.4) | 34.9 (21.5, 51.9) | 0.2 (0.1, 0.3) | 21.7 (13.2, 32.8) | -1.6 (-1.7, -1.6) |
| Tonga | 4.3 (2.7, 6.3) | 15.9 (9.6, 23.6) | 5.9 (3.5, 8.9) | 13.8 (8.2, 21.0) | -0.3 (-0.4, -0.1) |
| Trinidad and,bago | 81.4 (53.9, 113.2) | 19.9 (12.8, 28.1) | 103.2 (61.4, 150.8) | 10.0 (5.9, 14.7) | -2.5 (-2.8, -2.3) |
| Tunisia | 446.0 (263.7, 684.8) | 20.0 (11.4, 31.3) | 880.1 (467.6, 1428.6) | 13.3 (7.0, 21.8) | -1.5 (-1.6, -1.4) |
| Turkey | 4426.7 (2765.8, 6438.5) | 27.2 (16.6, 39.9) | 5804.3 (3349.5, 8759.0) | 12.5 (7.1, 19.0) | -2.4 (-2.7, -2.1) |
| Turkmenistan | 285.9 (204.3, 378.0) | 29.8 (20.6, 40.0) | 719.9 (472.8, 1010.5) | 36.0 (23.0, 51.0) | 0.3 (-0.2, 0.8) |
| Tuvalu | 1.5 (1.0, 2.2) | 47.1 (30.1, 68.0) | 1.6 (1.1, 2.4) | 31.2 (19.3, 46.0) | -1.3 (-1.4, -1.2) |
| Uganda | 727.0 (439.6, 1105.5) | 23.5 (13.9, 36.3) | 1010.6 (565.6, 1581.6) | 15.0 (8.2, 23.7) | -2.2 (-2.4, -1.9) |
| Ukraine | 13255.0 (8516.6, 18385.3) | 36.1 (22.8, 50.3) | 8695.9 (4983.2, 12887.8) | 19.8 (11.4, 29.2) | -2.7 (-3.0, -2.4) |
| United Arab Emirates | 45.0 (26.7, 68.2) | 26.0 (15.0, 39.6) | 133.7 (81.4, 201.0) | 15.3 (8.6, 23.2) | 0.0 (-0.5, 0.6) |
| United Kingdom | 6670.8 (3769.8, 9809.0) | 12.2 (6.9, 17.9) | 3060.6 (1693.0, 4566.9) | 3.5 (2.0, 5.1) | -4.2 (-4.4, -4.0) |
| United Republic of Tanzania | 937.8 (575.3, 1397.0) | 17.8 (10.5, 27.1) | 1790.1 (1016.9, 2829.3) | 15.3 (8.5, 24.3) | -0.5 (-0.6, -0.4) |
| United States of America | 13110.4 (7685.5, 18987.1) | 7.0 (4.1, 10.2) | 17071.1 (10002.8, 24867.2) | 4.9 (2.9, 7.1) | -1.6 (-1.9, -1.3) |
| United States Virgin Islands | 4.3 (2.6, 6.4) | 11.1 (6.6, 16.5) | 4.8 (2.8, 7.4) | 4.8 (2.8, 7.3) | -2.6 (-2.7, -2.4) |
| Uruguay | 317.8 (189.0, 464.5) | 14.7 (8.7, 21.6) | 228.0 (129.3, 337.5) | 6.4 (3.7, 9.3) | -3.0 (-3.2, -2.7) |
| Uzbekistan | 1819.3 (1264.3, 2426.2) | 30.1 (20.8, 40.2) | 2783.3 (1868.0, 3888.3) | 23.1 (15.0, 32.8) | -1.4 (-1.8, -1.0) |
| Vanuatu | 11.6 (7.3, 17.1) | 39.8 (24.3, 59.1) | 27.5 (17.5, 39.9) | 33.1 (20.5, 48.4) | -0.7 (-0.8, -0.7) |
| Venezuela (Bolivarian Republic of) | 720.6 (484.9, 988.6) | 15.1 (9.9, 20.8) | 1664.9 (1008.1, 2418.2) | 10.9 (6.5, 15.9) | -1.6 (-1.9, -1.3) |
| Viet Nam | 8391.0 (5177.8, 12653.4) | 41.0 (25.0, 62.2) | 16692.1 (10013.4, 24917.0) | 34.3 (20.3, 51.4) | -0.2 (-0.4, 0.0) |
| Yemen | 837.5 (494.6, 1290.1) | 36.9 (21.1, 57.6) | 1774.6 (988.2, 2836.4) | 27.8 (15.0, 45.0) | -1.2 (-1.3, -1.1) |
| Zambia | 342.9 (214.7, 508.0) | 25.8 (15.6, 38.9) | 738.2 (427.8, 1154.2) | 23.9 (13.6, 37.8) | -0.6 (-0.7, -0.4) |
| Zimbabwe | 429.6 (277.9, 619.4) | 23.8 (14.7, 35.3) | 1096.3 (698.7, 1581.8) | 35.3 (21.7, 51.3) | 2.0 (1.4, 2.6) |
| **DALYs** |  |  |  |  |  |
| Afghanistan | 40515.1 (24211.1, 61312.1) | 1053.7 (619.6, 1609.5) | 40422.7 (24523.3, 60630.1) | 729.6 (435.3, 1098.5) | -1.5 (-1.7, -1.3) |
| Albania | 4956.3 (3266.5, 6986.5) | 485.0 (309.0, 695.0) | 7234.5 (4152.6, 10874.1) | 303.8 (172.7, 458.2) | -1.4 (-1.6, -1.2) |
| Algeria | 30087.7 (18276.6, 45622.0) | 502.6 (292.0, 773.7) | 54641.4 (32600.4, 82344.1) | 309.5 (178.5, 469.6) | -1.6 (-1.6, -1.6) |
| American Samoa | 80.9 (55.8, 111.8) | 632.8 (426.8, 880.0) | 133.5 (89.7, 187.9) | 501.3 (330.9, 711.5) | -0.9 (-1.0, -0.8) |
| Andorra | 29.3 (17.0, 46.2) | 98.9 (56.1, 157.2) | 44.6 (24.5, 70.7) | 50.0 (27.7, 79.1) | -2.0 (-2.2, -1.8) |
| Angola | 19483.8 (12774.2, 27706.5) | 925.0 (592.1, 1326.1) | 39367.1 (25015.0, 57245.3) | 640.5 (397.7, 940.2) | -1.5 (-1.6, -1.4) |
| Antigua and Barbuda | 110.1 (74.8, 149.0) | 371.0 (256.8, 497.5) | 119.2 (82.5, 160.9) | 205.6 (139.8, 280.4) | -2.3 (-2.5, -2.0) |
| Argentina | 45431.7 (30187.4, 63294.1) | 257.8 (169.3, 361.3) | 31057.5 (20348.2, 44021.5) | 100.0 (65.9, 141.3) | -2.9 (-3.1, -2.8) |
| Armenia | 7201.1 (5149.5, 9494.4) | 510.9 (358.2, 680.7) | 6584.9 (4461.4, 8890.0) | 275.5 (187.0, 372.2) | -3.0 (-3.4, -2.6) |
| Australia | 16104.4 (9911.8, 22928.8) | 149.2 (90.3, 214.0) | 13546.4 (7912.8, 19933.1) | 49.5 (29.6, 72.3) | -3.8 (-3.9, -3.6) |
| Austria | 14564.0 (8924.2, 20726.0) | 208.8 (129.7, 295.1) | 6728.0 (4059.3, 9702.3) | 60.8 (38.0, 86.6) | -4.2 (-4.5, -3.9) |
| Azerbaijan | 16925.9 (12041.2, 22489.6) | 625.1 (438.3, 836.8) | 24216.1 (16244.1, 33643.9) | 464.3 (302.6, 651.2) | -1.0 (-1.3, -0.7) |
| Bahamas | 256.1 (180.4, 346.4) | 297.3 (207.0, 404.0) | 394.7 (260.7, 559.3) | 175.6 (113.9, 251.0) | -1.8 (-1.9, -1.7) |
| Bahrain | 451.3 (301.6, 628.5) | 523.9 (334.1, 747.4) | 989.6 (629.1, 1428.4) | 240.0 (143.1, 355.1) | -3.3 (-3.7, -2.9) |
| Bangladesh | 189051.6 (124898.0, 268465.4) | 735.1 (480.7, 1045.4) | 355698.6 (219334.7, 528410.8) | 484.0 (294.1, 721.8) | -1.3 (-1.5, -1.0) |
| Barbados | 487.7 (322.4, 680.9) | 298.7 (201.1, 412.5) | 525.5 (329.9, 753.4) | 182.8 (114.8, 262.6) | -1.8 (-2.1, -1.6) |
| Belarus | 39870.4 (27731.3, 54284.5) | 561.5 (387.9, 766.7) | 34674.6 (22467.4, 48915.5) | 390.7 (253.5, 550.7) | -2.0 (-2.4, -1.5) |
| Belgium | 17337.5 (10789.8, 24501.0) | 195.7 (123.2, 275.5) | 9250.8 (5648.4, 13358.5) | 64.5 (41.0, 91.4) | -3.5 (-3.6, -3.4) |
| Belize | 117.6 (82.5, 159.8) | 228.5 (160.2, 310.5) | 281.9 (197.3, 382.3) | 171.0 (117.2, 234.1) | -1.4 (-1.9, -0.9) |
| Benin | 8288.0 (5635.2, 11455.6) | 769.6 (518.2, 1067.1) | 15877.4 (10349.7, 22739.6) | 579.8 (374.0, 832.4) | -0.9 (-1.0, -0.8) |
| Bermuda | 65.0 (44.2, 89.7) | 193.4 (129.9, 268.5) | 57.9 (36.6, 83.5) | 75.0 (48.1, 107.5) | -3.2 (-3.3, -3.0) |
| Bhutan | 555.6 (343.1, 851.2) | 431.8 (259.6, 668.4) | 889.3 (537.9, 1350.7) | 272.4 (163.8, 414.4) | -1.6 (-1.7, -1.5) |
| Bolivia (Plurinational State of) | 5881.3 (3526.4, 9019.3) | 331.2 (196.0, 509.5) | 8086.9 (4561.9, 12601.4) | 164.1 (91.3, 256.8) | -2.3 (-2.5, -2.2) |
| Bosnia and Herzegovina | 8740.0 (5903.3, 12090.4) | 428.4 (281.6, 599.9) | 9850.9 (5940.5, 14318.5) | 279.3 (169.2, 405.5) | -1.7 (-1.8, -1.5) |
| Botswana | 2576.1 (1646.5, 3696.9) | 870.0 (544.9, 1253.4) | 3757.1 (2433.0, 5526.2) | 498.6 (318.4, 734.0) | -1.8 (-2.0, -1.6) |
| Brazil | 239869.2 (176428.4, 309695.8) | 492.5 (353.2, 644.6) | 237070.2 (170598.5, 310054.9) | 171.6 (122.3, 225.5) | -3.5 (-3.6, -3.4) |
| Brunei Darussalam | 316.0 (206.9, 449.4) | 579.9 (372.9, 830.3) | 459.5 (298.1, 649.9) | 254.5 (156.7, 367.1) | -2.7 (-2.9, -2.5) |
| Bulgaria | 48453.3 (33317.4, 64763.7) | 775.3 (509.2, 1058.2) | 37797.3 (24172.6, 53141.4) | 466.3 (301.7, 652.7) | -2.1 (-2.2, -1.9) |
| Burkina Faso | 11486.0 (7711.9, 16249.2) | 482.7 (318.0, 689.1) | 21468.5 (13641.0, 31355.0) | 423.0 (266.1, 619.9) | -0.2 (-0.3, -0.2) |
| Burundi | 11763.6 (6928.7, 17504.5) | 897.3 (525.0, 1336.1) | 12279.4 (7418.7, 18480.3) | 452.0 (268.3, 684.6) | -2.8 (-3.1, -2.5) |
| Cabo Verde | 501.7 (322.6, 715.7) | 403.5 (262.2, 572.8) | 962.4 (598.4, 1421.6) | 398.3 (247.0, 589.0) | -0.3 (-0.6, -0.1) |
| Cambodia | 27277.4 (18403.0, 38066.9) | 1097.9 (730.5, 1540.8) | 50793.9 (32567.6, 74140.9) | 768.5 (483.8, 1124.0) | -1.3 (-1.4, -1.2) |
| Cameroon | 18753.4 (12324.7, 26679.7) | 764.3 (493.2, 1094.6) | 53239.6 (32741.1, 80096.4) | 761.0 (464.0, 1144.1) | 0.1 (-0.4, 0.5) |
| Canada | 23030.7 (14366.4, 32695.8) | 127.7 (79.1, 181.9) | 23254.4 (14136.3, 34135.7) | 55.9 (34.6, 81.6) | -2.8 (-2.9, -2.6) |
| Central African Republic | 7360.5 (4601.5, 10864.7) | 1166.5 (717.3, 1722.0) | 11596.2 (6969.3, 17727.4) | 956.2 (566.8, 1454.1) | -0.7 (-0.8, -0.7) |
| Chad | 9856.3 (6464.9, 13927.6) | 636.0 (411.8, 903.5) | 21098.2 (13332.0, 30904.4) | 661.0 (411.7, 971.5) | 0.0 (-0.2, 0.3) |
| Chile | 13741.3 (9120.4, 19134.8) | 254.2 (165.9, 356.5) | 12763.9 (8125.3, 18302.0) | 89.0 (56.8, 127.4) | -3.1 (-3.2, -2.9) |
| China | 3064379.6 (2114240.5, 4267935.1) | 701.6 (465.3, 996.1) | 4056817.9 (2650272.2, 5790180.8) | 353.4 (227.1, 508.6) | -1.9 (-2.2, -1.7) |
| Colombia | 27549.5 (19512.5, 36599.8) | 292.1 (203.4, 391.1) | 29347.7 (19446.2, 41293.8) | 96.9 (64.1, 136.5) | -4.2 (-4.5, -4.0) |
| Comoros | 665.2 (402.0, 988.0) | 606.9 (364.7, 901.9) | 934.6 (563.5, 1424.2) | 352.1 (209.3, 538.4) | -2.1 (-2.3, -1.8) |
| Congo | 6396.0 (4091.1, 9279.5) | 1098.8 (690.5, 1597.7) | 10095.0 (6287.2, 15028.5) | 700.8 (432.6, 1039.9) | -1.8 (-1.9, -1.6) |
| Cook Islands | 33.8 (22.3, 48.2) | 491.4 (319.1, 703.7) | 36.2 (23.6, 52.5) | 258.0 (167.7, 375.8) | -2.1 (-2.3, -1.9) |
| Costa Rica | 1956.9 (1360.3, 2624.5) | 207.2 (143.1, 278.6) | 3321.1 (2218.3, 4548.4) | 110.4 (73.4, 151.5) | -2.5 (-2.8, -2.1) |
| Coted'Ivoire | 15684.9 (10274.0, 22706.2) | 701.3 (452.3, 1017.6) | 36771.9 (23126.8, 55459.0) | 589.8 (368.3, 888.3) | -0.7 (-0.8, -0.5) |
| Croatia | 15076.9 (10060.3, 20793.4) | 483.2 (317.1, 671.6) | 8169.1 (4989.7, 11771.7) | 152.1 (95.1, 217.4) | -4.0 (-4.2, -3.8) |
| Cuba | 12116.1 (8235.0, 16572.0) | 214.9 (146.0, 294.0) | 15858.8 (10420.0, 22224.8) | 146.6 (96.9, 205.2) | -1.4 (-1.6, -1.3) |
| Cyprus | 1205.0 (695.8, 1801.0) | 358.8 (192.2, 552.7) | 978.4 (560.2, 1482.6) | 89.7 (50.5, 136.4) | -4.9 (-5.1, -4.6) |
| Czechia | 36615.8 (23869.1, 50854.3) | 475.7 (309.3, 661.4) | 12484.4 (7665.7, 17824.4) | 100.4 (62.3, 142.9) | -5.2 (-5.5, -5.0) |
| Democratic People's Republic of Korea | 60960.7 (39013.0, 87047.1) | 701.6 (441.1, 1011.9) | 114974.5 (73293.6, 166972.2) | 629.5 (398.2, 919.2) | -0.4 (-0.5, -0.2) |
| Democratic Republic of the Congo | 63136.4 (39348.3, 91972.0) | 756.1 (460.3, 1108.4) | 125365.1 (75020.8, 192115.5) | 655.3 (383.5, 1011.5) | -0.6 (-0.7, -0.5) |
| Denmark | 8756.6 (5530.8, 12328.4) | 183.2 (117.6, 256.3) | 5119.5 (3154.5, 7249.6) | 71.9 (45.0, 101.3) | -3.4 (-3.6, -3.2) |
| Djibouti | 382.1 (231.0, 595.5) | 505.0 (302.7, 785.1) | 1313.7 (775.6, 2089.0) | 377.1 (219.2, 601.8) | -1.1 (-1.2, -1.0) |
| Dominica | 118.4 (79.6, 165.4) | 360.0 (241.7, 503.2) | 124.5 (79.4, 181.7) | 272.7 (171.8, 400.1) | -0.9 (-1.1, -0.7) |
| Dominican Republic | 5503.1 (3677.6, 7726.2) | 269.8 (177.6, 380.9) | 13497.3 (8563.7, 20230.7) | 242.9 (153.6, 364.4) | 0.1 (-0.1, 0.2) |
| Ecuador | 5993.5 (4205.8, 8094.6) | 199.8 (138.8, 271.0) | 8182.2 (5192.2, 11950.4) | 91.2 (57.6, 133.3) | -2.5 (-2.8, -2.3) |
| Egypt | 118878.9 (77813.2, 169173.0) | 873.1 (549.5, 1272.0) | 194696.0 (126847.8, 279593.0) | 612.3 (383.4, 891.4) | -0.8 (-1.0, -0.7) |
| El Salvador | 3875.3 (2616.0, 5340.1) | 234.9 (158.0, 323.5) | 4273.9 (2692.4, 6207.8) | 125.5 (79.5, 181.9) | -2.1 (-2.4, -1.8) |
| Equatorial Guinea | 1133.7 (717.6, 1661.3) | 1065.1 (664.9, 1567.0) | 1304.5 (742.3, 2105.0) | 495.1 (281.2, 790.0) | -2.9 (-3.3, -2.6) |
| Eritrea | 5356.7 (3377.2, 7988.2) | 801.9 (497.2, 1196.9) | 7681.9 (4629.6, 11922.0) | 497.2 (295.2, 768.4) | -1.6 (-1.7, -1.4) |
| Estonia | 7265.1 (4926.8, 9902.8) | 641.7 (434.4, 876.2) | 2106.3 (1359.0, 2973.4) | 137.5 (91.7, 191.7) | -6.4 (-6.9, -5.8) |
| Eswatini | 1265.6 (815.2, 1805.6) | 840.2 (530.6, 1207.4) | 2426.1 (1411.7, 3873.9) | 805.5 (464.2, 1274.8) | 0.3 (-0.3, 0.9) |
| Ethiopia | 65463.9 (43542.7, 98165.9) | 571.0 (372.4, 854.8) | 59368.4 (38189.9, 86837.0) | 248.7 (158.4, 366.1) | -3.1 (-3.3, -3.0) |
| Fiji | 1433.0 (966.0, 2032.0) | 696.4 (460.6, 993.7) | 2219.9 (1451.9, 3213.2) | 530.1 (339.4, 772.3) | -1.1 (-1.2, -0.9) |
| Finland | 7853.2 (5046.9, 11096.2) | 192.9 (124.2, 272.3) | 5498.2 (3318.3, 7962.0) | 69.9 (43.5, 100.0) | -3.2 (-3.2, -3.1) |
| France | 58565.6 (34857.4, 84420.7) | 120.5 (73.7, 171.6) | 42352.2 (24908.5, 62238.7) | 46.5 (28.9, 66.9) | -3.4 (-3.5, -3.2) |
| Gabon | 2425.8 (1553.2, 3507.9) | 783.1 (494.2, 1141.5) | 2989.6 (1852.4, 4505.3) | 543.7 (330.6, 819.1) | -1.3 (-1.4, -1.2) |
| Gambia | 1331.4 (848.1, 1933.6) | 687.0 (431.4, 1000.1) | 3725.9 (2304.1, 5498.1) | 698.4 (429.6, 1029.5) | 0.0 (-0.2, 0.1) |
| Georgia | 29555.9 (21062.4, 38819.5) | 867.5 (611.7, 1145.6) | 21369.0 (14387.3, 29101.9) | 641.8 (437.3, 868.1) | -1.4 (-1.8, -1.0) |
| Germany | 155737.4 (95466.5, 225460.5) | 211.3 (131.7, 304.2) | 86216.8 (51692.8, 126037.6) | 72.7 (46.1, 104.1) | -3.6 (-3.9, -3.3) |
| Ghana | 24956.2 (16021.1, 36011.3) | 702.2 (443.6, 1018.8) | 57449.4 (36020.1, 85209.4) | 613.0 (377.0, 913.6) | -0.1 (-0.4, 0.1) |
| Greece | 30365.8 (18832.8, 42822.4) | 361.6 (222.9, 511.4) | 18892.7 (11050.3, 27536.9) | 118.7 (74.2, 168.3) | -4.2 (-4.5, -3.9) |
| Greenland | 75.4 (49.4, 106.9) | 487.6 (305.1, 703.3) | 65.8 (42.6, 96.6) | 192.6 (118.4, 288.1) | -3.1 (-3.2, -3.0) |
| Grenada | 211.6 (143.5, 286.9) | 541.1 (373.5, 725.8) | 164.8 (110.6, 227.0) | 268.1 (175.5, 373.4) | -2.3 (-2.4, -2.2) |
| Guam | 137.2 (95.3, 188.8) | 340.4 (228.4, 474.7) | 241.5 (165.8, 330.7) | 213.2 (145.5, 293.5) | -1.3 (-1.6, -1.0) |
| Guatemala | 5129.0 (3698.2, 6792.9) | 270.3 (189.1, 363.0) | 10401.8 (7034.4, 14278.6) | 171.3 (114.6, 235.9) | -2.2 (-2.5, -1.8) |
| Guinea | 12273.2 (8109.3, 17370.5) | 672.0 (438.3, 959.1) | 20347.3 (12998.2, 29924.2) | 656.4 (414.7, 966.1) | 0.2 (0.1, 0.3) |
| Guinea-Bissau | 2735.6 (1778.4, 3997.3) | 1206.2 (776.3, 1763.9) | 4036.4 (2569.2, 5900.9) | 977.0 (617.9, 1424.1) | -0.6 (-0.6, -0.5) |
| Guyana | 2103.7 (1516.1, 2820.9) | 992.6 (706.8, 1337.6) | 1642.5 (1083.8, 2348.9) | 461.8 (299.6, 664.4) | -2.1 (-2.3, -1.9) |
| Haiti | 15740.5 (10300.3, 22140.5) | 882.8 (564.8, 1256.2) | 23566.2 (14243.2, 36484.0) | 599.1 (354.0, 929.3) | -1.1 (-1.2, -1.0) |
| Honduras | 4450.3 (2957.6, 6272.6) | 388.5 (255.8, 548.5) | 15366.8 (9745.1, 22494.9) | 453.9 (284.2, 665.4) | 0.7 (0.5, 0.9) |
| Hungary | 40726.4 (27235.1, 55890.4) | 508.3 (337.4, 699.3) | 16502.5 (10452.1, 23409.4) | 149.2 (96.0, 210.3) | -4.4 (-4.6, -4.2) |
| Iceland | 200.7 (124.8, 285.9) | 121.3 (76.2, 172.0) | 143.5 (84.7, 210.9) | 41.0 (24.7, 59.8) | -3.6 (-3.8, -3.5) |
| India | 1116236.2 (790454.0, 1485605.0) | 434.4 (298.9, 585.2) | 2053677.9 (1423932.6, 2782919.6) | 312.2 (212.7, 426.3) | -1.2 (-1.3, -1.1) |
| Indonesia | 594728.9 (419897.6, 797553.3) | 1063.9 (734.8, 1444.1) | 1371115.5 (919351.7, 1876828.1) | 1038.2 (682.8, 1430.7) | 0.0 (-0.1, 0.1) |
| Iran (Islamic Republic of) | 61591.3 (44031.7, 82456.1) | 455.0 (307.6, 626.9) | 93773.5 (63497.1, 127820.3) | 227.3 (150.0, 313.8) | -2.3 (-2.4, -2.2) |
| Iraq | 38839.9 (25220.5, 55414.1) | 878.8 (567.0, 1253.7) | 81810.6 (50431.8, 121844.4) | 644.6 (389.0, 964.9) | -1.6 (-1.7, -1.4) |
| Ireland | 5793.3 (3894.4, 7849.8) | 249.8 (166.0, 340.2) | 2835.4 (1844.0, 3934.4) | 61.2 (40.0, 84.8) | -4.6 (-4.8, -4.4) |
| Israel | 4833.4 (3201.9, 6681.5) | 180.0 (118.3, 249.9) | 4205.7 (2632.1, 5965.8) | 58.9 (37.5, 83.1) | -4.1 (-4.3, -3.9) |
| Italy | 100553.2 (63769.7, 141331.7) | 200.4 (127.1, 281.6) | 55391.5 (32181.1, 82261.1) | 57.6 (35.5, 83.3) | -4.0 (-4.2, -3.9) |
| Jamaica | 3449.6 (2372.3, 4716.8) | 349.4 (242.3, 475.4) | 4204.0 (2636.3, 6046.1) | 248.2 (155.9, 356.8) | -0.9 (-1.3, -0.4) |
| Japan | 244819.7 (153189.3, 346371.2) | 267.4 (164.5, 381.5) | 208136.7 (121288.4, 309249.9) | 101.2 (64.1, 145.0) | -3.2 (-3.4, -3.1) |
| Jordan | 4022.0 (2659.8, 5733.9) | 570.3 (368.2, 823.8) | 9309.7 (5939.9, 13480.3) | 244.6 (150.6, 359.0) | -3.3 (-3.7, -3.0) |
| Kazakhstan | 57603.4 (41380.8, 76713.5) | 850.4 (599.4, 1142.8) | 62311.3 (42975.3, 84387.1) | 672.3 (451.2, 921.3) | -1.4 (-1.9, -1.0) |
| Kenya | 13799.0 (8855.3, 20372.1) | 311.0 (196.5, 462.2) | 37783.1 (23935.1, 55325.9) | 309.4 (190.1, 460.3) | 0.2 (0.0, 0.4) |
| Kiribati | 209.4 (142.0, 293.1) | 959.2 (640.7, 1350.6) | 372.7 (246.8, 544.6) | 859.6 (560.5, 1257.3) | -0.4 (-0.4, -0.3) |
| Kuwait | 699.2 (475.2, 957.8) | 199.3 (130.7, 277.0) | 2075.8 (1332.0, 2998.7) | 124.7 (77.2, 183.2) | -1.3 (-2.2, -0.4) |
| Kyrgyzstan | 15384.2 (11181.7, 20054.2) | 947.9 (681.3, 1243.5) | 12302.6 (8657.5, 16277.6) | 453.5 (311.9, 607.5) | -3.1 (-3.7, -2.5) |
| Lao People's Democratic Republic | 19526.2 (12682.6, 27932.8) | 1645.6 (1056.8, 2359.1) | 23858.8 (15449.7, 34674.9) | 919.8 (590.2, 1338.3) | -2.1 (-2.2, -2.0) |
| Latvia | 14536.2 (9757.4, 19962.9) | 732.9 (491.9, 1006.6) | 8707.2 (5586.0, 12270.2) | 370.4 (245.5, 515.8) | -2.9 (-3.2, -2.6) |
| Lebanon | 5800.0 (3600.3, 8584.3) | 496.2 (301.8, 740.9) | 5209.7 (3183.8, 7741.0) | 152.6 (94.6, 225.2) | -3.9 (-4.3, -3.6) |
| Lesotho | 2989.1 (1949.1, 4256.0) | 659.9 (423.0, 949.3) | 6185.4 (3815.3, 9347.4) | 1056.8 (643.9, 1597.2) | 2.4 (1.9, 2.9) |
| Liberia | 4344.5 (2919.7, 6191.7) | 687.5 (456.6, 983.4) | 7130.6 (4418.9, 10892.3) | 604.1 (370.8, 920.3) | -0.6 (-0.7, -0.4) |
| Libya | 3157.0 (1902.2, 4779.6) | 303.2 (180.6, 462.7) | 8874.6 (5304.5, 13796.2) | 304.7 (178.5, 476.6) | 0.3 (0.1, 0.5) |
| Lithuania | 10226.1 (7003.9, 13887.5) | 412.1 (282.6, 558.5) | 8388.0 (5384.6, 11912.9) | 253.3 (167.6, 354.8) | -1.6 (-1.9, -1.3) |
| Luxembourg | 947.4 (608.6, 1327.5) | 309.2 (198.9, 432.6) | 387.2 (238.7, 554.3) | 61.5 (38.7, 87.3) | -5.2 (-5.3, -5.1) |
| Madagascar | 23718.7 (15525.5, 33637.9) | 821.5 (529.7, 1174.6) | 42487.9 (26060.9, 63982.7) | 656.3 (397.0, 994.6) | -0.8 (-0.9, -0.7) |
| Malawi | 11446.3 (7305.4, 16415.9) | 541.2 (338.5, 784.9) | 20892.9 (12651.5, 31310.9) | 513.1 (304.8, 774.1) | -0.5 (-0.7, -0.2) |
| Malaysia | 41145.2 (29022.6, 55756.9) | 806.4 (567.1, 1094.4) | 78798.2 (53730.9, 108005.3) | 502.2 (337.8, 693.8) | -1.4 (-1.5, -1.2) |
| Maldives | 626.5 (437.0, 863.1) | 1209.9 (829.3, 1673.6) | 626.9 (399.0, 912.0) | 334.2 (212.2, 484.0) | -4.6 (-4.8, -4.4) |
| Mali | 13733.9 (9053.2, 19480.7) | 618.8 (398.7, 884.9) | 23054.7 (14583.6, 33759.6) | 473.1 (294.8, 695.0) | -0.8 (-0.9, -0.6) |
| Malta | 540.9 (356.0, 747.3) | 233.7 (151.7, 324.8) | 340.8 (207.9, 493.3) | 58.5 (36.4, 84.1) | -4.6 (-4.8, -4.4) |
| Marshall Islands | 97.8 (66.9, 137.6) | 1046.5 (705.8, 1474.5) | 188.4 (121.3, 279.4) | 896.7 (565.1, 1329.5) | -0.4 (-0.5, -0.3) |
| Mauritania | 4549.5 (3008.8, 6511.9) | 823.9 (538.4, 1188.3) | 5833.8 (3547.9, 8901.5) | 501.0 (301.1, 764.8) | -1.8 (-1.9, -1.6) |
| Mauritius | 4023.0 (2937.1, 5282.8) | 978.1 (703.2, 1293.5) | 3740.3 (2692.4, 4911.3) | 373.5 (265.7, 494.5) | -4.2 (-4.8, -3.7) |
| Mexico | 52683.9 (36959.2, 70293.2) | 236.6 (162.2, 318.8) | 85639.7 (59570.6, 115880.8) | 123.8 (85.0, 168.6) | -2.3 (-2.4, -2.1) |
| Micronesia (Federated States of) | 315.0 (204.7, 451.5) | 1131.2 (729.0, 1625.1) | 385.3 (251.0, 565.8) | 887.9 (568.5, 1313.6) | -0.8 (-0.9, -0.7) |
| Monaco | 104.3 (58.2, 158.9) | 238.0 (136.9, 357.8) | 59.6 (33.6, 90.8) | 98.2 (56.7, 148.6) | -3.0 (-3.1, -2.8) |
| Mongolia | 6536.7 (4600.4, 8805.8) | 1126.7 (780.2, 1532.8) | 8379.0 (5864.1, 11277.5) | 640.3 (433.8, 877.2) | -2.2 (-2.7, -1.7) |
| Montenegro | 1658.8 (1083.0, 2337.7) | 496.3 (321.1, 702.7) | 2581.6 (1613.8, 3699.7) | 505.1 (309.5, 729.1) | -0.2 (-0.4, 0.1) |
| Morocco | 40859.7 (25191.3, 61409.8) | 520.8 (317.5, 786.2) | 76692.7 (45277.4, 119013.8) | 412.9 (239.3, 643.4) | -0.7 (-0.7, -0.6) |
| Mozambique | 23030.3 (14861.5, 33392.5) | 695.1 (440.9, 1016.2) | 50445.1 (30032.7, 76295.6) | 796.5 (467.6, 1206.0) | 1.0 (0.8, 1.2) |
| Myanmar | 203323.8 (134197.6, 291147.3) | 1537.0 (1004.9, 2201.1) | 236763.4 (151609.7, 345546.9) | 871.8 (552.0, 1277.0) | -2.1 (-2.3, -2.0) |
| Namibia | 3022.6 (2050.3, 4135.5) | 882.8 (580.6, 1225.8) | 4836.8 (3057.0, 7079.8) | 680.8 (424.5, 1001.0) | -1.1 (-1.4, -0.8) |
| Nauru | 36.3 (23.3, 52.8) | 1291.7 (822.0, 1871.1) | 41.8 (26.8, 62.2) | 1175.0 (746.0, 1745.7) | -0.4 (-0.8, 0.0) |
| Nepal | 31730.0 (20679.8, 45491.2) | 628.9 (398.5, 911.6) | 51826.3 (32104.3, 77260.4) | 410.6 (249.1, 617.4) | -1.5 (-1.7, -1.3) |
| Netherlands | 19376.9 (12306.4, 27245.6) | 168.9 (107.8, 237.0) | 14999.2 (9217.9, 21454.1) | 71.0 (44.2, 101.1) | -3.3 (-3.6, -3.1) |
| New Zealand | 3416.1 (2137.9, 4827.0) | 156.1 (96.9, 221.4) | 3137.4 (1867.1, 4567.9) | 63.1 (38.1, 91.5) | -3.1 (-3.2, -2.9) |
| Nicaragua | 2500.5 (1722.2, 3423.0) | 298.2 (202.4, 411.0) | 4566.1 (3024.5, 6465.9) | 169.6 (111.5, 240.6) | -1.9 (-2.0, -1.7) |
| Niger | 9041.1 (5845.5, 12983.4) | 598.0 (378.8, 864.4) | 22640.3 (13981.7, 34163.2) | 512.4 (311.1, 775.4) | -0.4 (-0.5, -0.3) |
| Nigeria | 198493.1 (135360.4, 271945.9) | 833.2 (558.0, 1150.8) | 233957.4 (158559.1, 331051.2) | 489.3 (325.1, 694.4) | -2.0 (-2.1, -1.8) |
| Niue | 9.0 (5.8, 13.1) | 739.5 (482.3, 1074.8) | 6.7 (4.3, 9.6) | 562.9 (361.3, 811.1) | -1.1 (-1.2, -1.0) |
| North Macedonia | 8409.7 (5648.0, 11422.9) | 876.7 (574.0, 1208.7) | 11254.4 (7010.5, 16267.9) | 702.8 (416.5, 1035.3) | -1.0 (-1.4, -0.7) |
| Northern Mariana Islands | 69.1 (45.1, 99.6) | 632.4 (409.9, 909.8) | 119.4 (81.1, 165.1) | 415.6 (274.1, 583.4) | -1.5 (-1.7, -1.3) |
| Norway | 6855.3 (4308.6, 9712.0) | 165.2 (105.2, 232.8) | 3311.0 (2007.8, 4841.4) | 54.4 (33.5, 79.1) | -3.8 (-3.9, -3.7) |
| Oman | 1862.2 (1119.7, 2846.5) | 494.6 (293.4, 754.6) | 2874.5 (1824.0, 4267.6) | 267.2 (164.9, 399.9) | -1.6 (-1.8, -1.4) |
| Pakistan | 122811.8 (80458.2, 174287.2) | 402.5 (259.5, 577.3) | 260548.4 (171849.2, 375854.6) | 392.0 (253.0, 570.5) | -0.5 (-0.7, -0.3) |
| Palau | 44.5 (29.2, 64.6) | 793.6 (514.8, 1153.2) | 78.8 (51.6, 113.4) | 622.9 (398.2, 906.1) | -0.6 (-0.7, -0.6) |
| Palestine | 2930.9 (1789.6, 4369.6) | 645.2 (387.6, 967.0) | 4418.4 (2869.4, 6336.5) | 349.6 (219.4, 508.3) | -2.0 (-2.3, -1.8) |
| Panama | 2220.1 (1548.6, 2994.0) | 275.6 (190.3, 373.3) | 3878.3 (2472.2, 5482.9) | 159.6 (101.7, 225.6) | -2.0 (-2.2, -1.9) |
| Papua New Guinea | 7988.8 (5054.6, 11950.0) | 792.2 (493.9, 1186.9) | 18082.5 (11224.1, 27123.8) | 641.3 (393.9, 960.5) | -0.7 (-0.7, -0.6) |
| Paraguay | 4255.0 (2921.6, 5788.3) | 355.6 (241.3, 486.2) | 7048.5 (4493.6, 10170.5) | 225.0 (142.0, 326.1) | -1.5 (-1.6, -1.4) |
| Peru | 10298.1 (6692.3, 14840.5) | 154.1 (99.4, 222.9) | 14152.6 (8666.6, 21401.2) | 76.1 (46.5, 115.0) | -2.8 (-3.3, -2.4) |
| Philippines | 103213.8 (74269.1, 137897.2) | 630.8 (445.2, 849.7) | 298399.8 (212442.3, 403240.7) | 630.1 (441.7, 857.7) | 0.2 (0.0, 0.4) |
| Poland | 106486.5 (71590.2, 144910.8) | 454.1 (301.8, 620.5) | 61318.9 (39340.4, 86323.2) | 149.2 (97.2, 208.7) | -4.1 (-4.3, -4.0) |
| Portugal | 34685.7 (22568.7, 47996.6) | 461.9 (295.1, 644.7) | 12313.5 (7512.6, 18039.0) | 79.4 (50.5, 114.2) | -6.1 (-6.2, -5.9) |
| Puerto Rico | 2530.0 (1710.8, 3485.4) | 128.7 (86.6, 177.7) | 2189.7 (1385.0, 3148.6) | 56.7 (36.8, 80.7) | -3.2 (-3.4, -2.9) |
| Qatar | 284.1 (179.8, 416.7) | 517.1 (313.6, 767.5) | 849.8 (517.5, 1301.0) | 169.4 (97.9, 263.5) | -4.1 (-4.7, -3.4) |
| Republic of Korea | 105142.8 (70205.3, 146587.2) | 712.9 (451.5, 1017.1) | 57193.1 (33757.1, 86062.7) | 110.9 (65.2, 167.2) | -6.6 (-6.8, -6.3) |
| Republic of Moldova | 18757.1 (13606.5, 24591.9) | 795.6 (561.5, 1057.0) | 14808.6 (10451.6, 19717.3) | 447.1 (315.1, 596.4) | -2.4 (-2.7, -2.0) |
| Romania | 86669.2 (59241.3, 118895.4) | 597.5 (396.3, 830.5) | 69518.5 (43762.7, 98308.6) | 323.3 (208.2, 452.2) | -2.8 (-3.2, -2.5) |
| Russian Federation | 727821.0 (498306.9, 986082.6) | 760.0 (512.2, 1037.4) | 577885.6 (382333.7, 795513.8) | 439.8 (291.7, 605.4) | -2.7 (-3.2, -2.1) |
| Rwanda | 16672.9 (10545.2, 24503.7) | 1032.3 (642.3, 1525.8) | 13010.4 (7680.8, 19994.6) | 384.1 (221.4, 595.7) | -4.3 (-4.8, -3.9) |
| Saint Kitts and Nevis | 152.4 (105.1, 206.5) | 739.9 (512.6, 1002.2) | 128.9 (86.9, 174.8) | 347.9 (227.3, 480.6) | -2.6 (-2.8, -2.3) |
| Saint Lucia | 251.7 (173.3, 340.1) | 545.6 (369.6, 742.6) | 310.3 (201.0, 435.9) | 234.5 (151.1, 330.1) | -3.2 (-3.5, -2.9) |
| Saint Vincent and the Grenadines | 159.8 (109.2, 218.0) | 405.7 (275.3, 555.5) | 200.0 (135.7, 273.6) | 256.6 (172.6, 352.4) | -1.5 (-1.7, -1.4) |
| Samoa | 358.7 (235.3, 507.9) | 764.9 (494.8, 1086.9) | 511.7 (336.1, 727.2) | 634.4 (411.0, 904.1) | -0.6 (-0.7, -0.5) |
| San Marino | 31.3 (18.5, 46.4) | 153.8 (91.4, 227.7) | 25.3 (14.0, 39.7) | 54.4 (31.1, 84.4) | -2.9 (-3.2, -2.7) |
| Sao,me and Principe | 210.5 (143.0, 295.2) | 597.1 (401.8, 841.1) | 367.6 (244.3, 527.1) | 591.4 (388.8, 848.1) | -0.1 (-0.3, 0.1) |
| Saudi Arabia | 20001.1 (12379.2, 30075.5) | 620.2 (377.2, 933.8) | 46471.7 (29626.9, 68741.3) | 388.4 (241.6, 576.3) | -1.7 (-1.8, -1.6) |
| Senegal | 12050.0 (7911.1, 17208.8) | 671.3 (435.5, 967.1) | 22020.5 (13690.4, 32829.9) | 521.7 (320.2, 780.7) | -0.8 (-0.9, -0.7) |
| Serbia | 33819.2 (22060.9, 47512.6) | 629.2 (398.4, 898.5) | 30053.0 (18652.1, 43930.6) | 318.8 (198.0, 465.5) | -2.8 (-3.0, -2.5) |
| Seychelles | 215.2 (149.5, 295.4) | 691.9 (481.0, 949.2) | 263.9 (179.6, 363.4) | 409.2 (274.7, 566.5) | -1.4 (-1.5, -1.2) |
| Sierra Leone | 8133.7 (5259.3, 11661.0) | 723.4 (464.7, 1039.5) | 13596.5 (8490.8, 20070.0) | 647.5 (401.2, 955.8) | -0.1 (-0.3, 0.1) |
| Singapore | 5081.3 (3483.4, 6843.1) | 432.1 (290.7, 586.8) | 3423.4 (2225.6, 4834.6) | 72.7 (46.8, 103.3) | -5.8 (-6.0, -5.6) |
| Slovakia | 13549.4 (8927.6, 18735.6) | 413.0 (269.9, 572.3) | 9472.4 (6011.0, 13534.7) | 177.7 (112.2, 254.7) | -2.8 (-2.9, -2.8) |
| Slovenia | 3851.1 (2522.6, 5391.8) | 283.4 (186.2, 395.8) | 2016.4 (1186.4, 2978.6) | 73.8 (44.6, 107.9) | -4.6 (-4.8, -4.5) |
| Solomon Islands | 774.8 (466.1, 1149.9) | 1018.3 (613.6, 1509.5) | 1813.8 (1179.9, 2663.1) | 919.0 (591.7, 1355.3) | -0.3 (-0.4, -0.1) |
| Somalia | 9966.8 (5905.0, 15566.7) | 719.8 (426.1, 1111.9) | 17462.1 (9883.2, 27971.1) | 502.6 (283.5, 802.6) | -1.2 (-1.3, -1.1) |
| South Africa | 54124.6 (37170.2, 73384.2) | 460.2 (310.9, 628.7) | 120975.3 (86869.4, 161025.2) | 486.0 (341.6, 653.8) | 0.2 (-0.3, 0.6) |
| South Sudan | 7811.0 (4673.7, 12007.4) | 548.7 (325.0, 844.7) | 8767.7 (5113.0, 14035.6) | 410.8 (236.4, 657.8) | -1.2 (-1.5, -0.9) |
| Spain | 67526.0 (41878.8, 95975.7) | 221.3 (136.5, 315.2) | 34088.7 (19952.4, 50026.6) | 54.6 (33.8, 78.3) | -4.5 (-4.7, -4.3) |
| Sri Lanka | 38027.1 (25902.0, 52462.2) | 690.7 (461.6, 958.3) | 61366.3 (34961.2, 92998.2) | 425.1 (239.9, 646.9) | -1.2 (-1.4, -1.0) |
| Sudan | 39519.7 (24323.0, 59026.2) | 761.8 (463.0, 1143.2) | 49711.7 (28850.0, 76839.4) | 459.2 (264.8, 709.2) | -1.8 (-1.9, -1.8) |
| Suriname | 604.3 (411.6, 829.4) | 429.6 (289.1, 593.9) | 1128.4 (722.6, 1635.7) | 319.5 (202.3, 465.3) | -1.0 (-1.3, -0.8) |
| Sweden | 15039.2 (9405.6, 21564.9) | 164.3 (104.1, 234.2) | 8948.6 (5317.6, 13299.6) | 66.3 (40.1, 97.8) | -3.0 (-3.1, -2.9) |
| Switzerland | 9421.3 (5748.5, 13544.8) | 149.7 (93.2, 213.0) | 5190.2 (3050.6, 7560.6) | 45.3 (27.5, 65.1) | -3.8 (-3.9, -3.8) |
| Syrian Arab Republic | 17155.4 (11041.1, 24804.2) | 573.8 (362.4, 836.1) | 25797.2 (15762.0, 39199.2) | 369.4 (218.8, 566.0) | -1.9 (-2.2, -1.7) |
| Taiwan (Province of China) | 41120.2 (29971.7, 53791.0) | 485.7 (342.4, 646.2) | 26894.9 (18155.7, 36886.3) | 117.5 (79.4, 161.3) | -4.5 (-4.8, -4.2) |
| Tajikistan | 11026.3 (7726.3, 15103.2) | 740.8 (513.1, 1020.5) | 16247.7 (10862.9, 22848.1) | 551.5 (357.3, 786.4) | -1.3 (-1.7, -0.9) |
| Thailand | 106540.7 (73125.2, 147185.9) | 549.2 (371.7, 763.0) | 199628.1 (129869.0, 286695.5) | 340.5 (220.5, 490.1) | -2.1 (-2.3, -1.9) |
| Timor-Leste | 1404.9 (925.1, 2015.4) | 903.4 (587.1, 1294.6) | 3951.7 (2456.2, 5807.9) | 834.2 (513.8, 1230.5) | -0.1 (-0.3, 0.1) |
| Togo | 5011.6 (3369.6, 7042.1) | 729.0 (483.2, 1031.8) | 14063.4 (8862.3, 20616.1) | 665.4 (413.1, 977.7) | -0.3 (-0.5, -0.2) |
| Tokelau | 5.9 (3.8, 8.7) | 800.9 (511.8, 1177.5) | 4.2 (2.7, 6.2) | 508.8 (323.8, 754.8) | -1.6 (-1.6, -1.5) |
| Tonga | 113.2 (73.9, 161.9) | 373.1 (239.5, 536.7) | 139.8 (88.5, 205.5) | 317.3 (200.2, 466.9) | -0.4 (-0.5, -0.3) |
| Trinidad and,bago | 1798.5 (1247.7, 2432.7) | 400.1 (273.5, 545.2) | 2186.6 (1379.7, 3137.1) | 206.2 (128.8, 296.8) | -2.6 (-2.8, -2.3) |
| Tunisia | 10165.6 (6283.0, 15286.9) | 389.6 (234.4, 593.3) | 18463.2 (10571.7, 29042.6) | 259.5 (146.5, 410.3) | -1.5 (-1.6, -1.4) |
| Turkey | 105883.0 (69478.6, 150376.4) | 572.6 (369.9, 819.2) | 119311.5 (73630.1, 176512.5) | 239.2 (145.3, 355.9) | -2.9 (-3.1, -2.6) |
| Turkmenistan | 7755.8 (5774.3, 10028.7) | 726.8 (531.4, 948.8) | 19654.4 (13575.4, 26953.4) | 871.2 (589.2, 1204.1) | 0.4 (-0.1, 0.8) |
| Tuvalu | 43.1 (29.2, 60.4) | 1134.0 (753.0, 1607.6) | 43.8 (28.9, 62.8) | 750.0 (487.5, 1082.5) | -1.3 (-1.4, -1.2) |
| Uganda | 18652.4 (11533.6, 28012.7) | 530.8 (324.6, 801.3) | 26825.7 (15709.4, 41219.0) | 335.5 (193.8, 518.1) | -2.2 (-2.5, -1.9) |
| Ukraine | 265904.4 (180427.4, 360619.0) | 685.9 (460.1, 934.2) | 177017.3 (108461.5, 255745.0) | 415.8 (256.0, 600.5) | -2.3 (-2.6, -2.0) |
| United Arab Emirates | 1423.3 (888.3, 2107.8) | 575.4 (353.3, 852.5) | 5316.3 (3477.0, 7587.1) | 299.3 (179.9, 443.5) | -0.9 (-1.3, -0.4) |
| United Kingdom | 111227.4 (68424.1, 157394.0) | 207.3 (129.4, 291.5) | 50566.7 (30682.0, 72440.8) | 63.5 (39.9, 89.7) | -4.0 (-4.2, -3.9) |
| United Republic of Tanzania | 24718.2 (15589.1, 36152.8) | 409.3 (253.3, 604.7) | 44702.8 (26539.7, 69225.0) | 329.0 (192.2, 511.8) | -0.8 (-0.8, -0.7) |
| United States of America | 254734.8 (162579.2, 356307.7) | 140.3 (90.5, 195.2) | 345248.0 (222487.4, 483357.0) | 105.1 (68.6, 146.4) | -1.3 (-1.5, -1.1) |
| United States Virgin Islands | 101.9 (64.7, 148.0) | 225.3 (140.5, 329.9) | 99.6 (61.7, 148.2) | 102.1 (63.0, 152.6) | -2.4 (-2.6, -2.3) |
| Uruguay | 6299.2 (4066.5, 8873.0) | 292.2 (188.3, 411.7) | 3983.5 (2452.1, 5708.6) | 123.9 (78.6, 175.1) | -3.1 (-3.3, -2.9) |
| Uzbekistan | 47371.9 (34922.4, 61168.6) | 742.4 (543.4, 961.1) | 74048.1 (52458.1, 99964.3) | 525.5 (362.4, 718.8) | -1.6 (-2.0, -1.3) |
| Vanuatu | 342.2 (219.4, 504.8) | 951.0 (603.1, 1398.5) | 820.5 (537.9, 1180.4) | 809.1 (522.7, 1165.0) | -0.6 (-0.7, -0.6) |
| Venezuela (Bolivarian Republic of) | 17018.2 (12000.5, 22889.1) | 322.4 (224.5, 435.4) | 36780.8 (23439.1, 52705.7) | 225.7 (142.1, 324.7) | -1.8 (-2.1, -1.5) |
| Viet Nam | 196054.6 (124430.5, 292202.1) | 893.7 (563.1, 1335.0) | 394533.4 (245069.0, 580615.7) | 729.4 (448.4, 1077.2) | -0.3 (-0.4, -0.1) |
| Yemen | 22022.3 (13245.4, 33716.2) | 815.3 (483.4, 1254.2) | 44366.3 (25751.7, 69588.9) | 580.5 (329.3, 917.1) | -1.4 (-1.5, -1.2) |
| Zambia | 9149.1 (5891.8, 13316.6) | 583.9 (369.6, 858.6) | 19687.8 (11768.8, 30411.3) | 522.9 (308.9, 807.6) | -0.7 (-0.9, -0.6) |
| Zimbabwe | 10461.3 (7010.5, 14751.1) | 497.2 (324.5, 712.8) | 29112.5 (18974.2, 41938.0) | 781.3 (500.5, 1124.2) | 2.1 (1.5, 2.8) |

**Notes:** ASR: age-standardized rates; DALYs: disability-adjusted life years; EAPCs: estimated annual percentage changes.

**Table S4. Mortality, and DALYs of kidney dysfunction exposure-associated stroke and its temporal trends from 1990, 2021 by gender, and age.**

|  | **1990** | | **2021** | | **EAPC 95%CI** |
| --- | --- | --- | --- | --- | --- |
|  | **Number** | **ASR, per**  **100,000 persons** | **Number** | **ASR, per**  **100,000 persons** |  |
| **Deaths** |  |  |  |  |  |
| **Gender** |  |  |  |  |  |
| Female | 248500.4 (161420.1, 343829.8) | 22.5 (14.3, 31.3) | 318921.6 (202650.9, 450077.7) | 12.4 (7.9, 17.5) | -2.1 (-2.2, -1.9) |
| Male | 217300.8 (147774.3, 298488.7) | 25.2 (16.4, 35.3) | 335262.1 (221687.9, 464270.1) | 16.1 (10.4, 22.6) | -1.5 (-1.6, -1.4) |
| **Age** |  |  |  |  |  |
| 25-29 years | 1089.7 (692.8, 1577.8) | 0.3 (0.2, 0.4) | 969.4 (595.0, 1433.3) | 0.2 (0.1, 0.2) | -1.4 (-1.5, -1.2) |
| 30-34 years | 1996.9 (1362.0, 2787.5) | 0.5 (0.4, 0.7) | 2168.7 (1411.6, 3001.4) | 0.4 (0.2, 0.5) | -1.2 (-1.4, -1.1) |
| 35-39 years | 3662.4 (2615.5, 4922.2) | 1.0 (0.7, 1.4) | 3948.2 (2739.5, 5453.5) | 0.7 (0.5, 1.0) | -1.3 (-1.4, -1.2) |
| 40-44 years | 6258.7 (4647.6, 8213.5) | 2.2 (1.6, 2.9) | 7491.2 (5369.2, 10244.0) | 1.5 (1.1, 2.1) | -1.3 (-1.4, -1.2) |
| 45-49 years | 10560.4 (8127.5, 13594.2) | 4.6 (3.5, 5.9) | 13951.8 (10276.3, 18278.4) | 3.0 (2.2, 3.9) | -1.4 (-1.5, -1.3) |
| 50-54 years | 21179.2 (16414.5, 26833.2) | 10.0 (7.7, 12.6) | 26467.2 (19565.4, 33881.7) | 6.0 (4.4, 7.6) | -1.7 (-1.8, -1.6) |
| 55-59 years | 33481.4 (25646.4, 42405.9) | 18.1 (13.9, 22.9) | 42381.6 (32267.2, 53710.0) | 10.7 (8.2, 13.6) | -1.8 (-1.9, -1.7) |
| 60-64 years | 50984.4 (38755.5, 65873.7) | 31.7 (24.1, 41.0) | 60471.2 (46013.7, 78530.3) | 18.9 (14.4, 24.5) | -1.9 (-2.0, -1.8) |
| 65-69 years | 63647.8 (48026.3, 81666.1) | 51.5 (38.9, 66.1) | 85040.3 (63393.5, 110215.1) | 30.8 (23.0, 40.0) | -1.9 (-2.1, -1.8) |
| 70-74 years | 75186.8 (52745.5, 98415.9) | 88.8 (62.3, 116.3) | 104735.8 (72530.3, 139854.8) | 50.9 (35.2, 67.9) | -1.9 (-2.1, -1.7) |
| 75-79 years | 81708.1 (53103.1, 111419.9) | 132.7 (86.3, 181.0) | 100747.2 (65429.8, 140866.4) | 76.4 (49.6, 106.8) | -1.8 (-1.9, -1.6) |
| 80-84 years | 54885.0 (27580.4, 86090.4) | 155.2 (78.0, 243.4) | 81951.7 (42779.9, 126661.0) | 93.6 (48.8, 144.6) | -1.7 (-1.8, -1.6) |
| 85-89 years | 41975.6 (20673.2, 64817.0) | 277.8 (136.8, 428.9) | 76001.8 (38986.0, 116115.4) | 166.2 (85.3, 254.0) | -1.8 (-1.9, -1.6) |
| 90-94 years | 19184.7 (10340.7, 28614.1) | 447.7 (241.3, 667.7) | 47857.5 (25683.1, 71619.6) | 267.5 (143.6, 400.4) | -1.8 (-1.9, -1.7) |
| **DALYs** |  |  |  |  |  |
| **Gender** |  |  |  |  |  |
| Female | 5437548.4 (3748471.8, 7288266.9) | 468.7 (319.9, 631.4) | 6816695.4 (4593478.2, 9310284.1) | 268.0 (180.8, 366.1) | -2.0 (-2.1, -1.9) |
| Male | 5406181.8 (3838141.4, 7277946.7) | 547.0 (377.8, 745.5) | 8003915.3 (5557656.5, 10810123.2) | 359.8 (246.2, 489.6) | -1.4 (-1.5, -1.3) |
| **Age** |  |  |  |  |  |
| 25-29 years | 83765.9 (53136.4, 119048.1) | 18.9 (12.0, 26.9) | 78064.3 (48057.6, 113716.3) | 13.3 (8.2, 19.3) | -1.2 (-1.4, -1.1) |
| 30-34 years | 135656.5 (92708.8, 191816.6) | 35.2 (24.1, 49.8) | 152780.8 (99102.8, 216345.2) | 25.3 (16.4, 35.8) | -1.1 (-1.2, -1.0) |
| 35-39 years | 220071.6 (156736.7, 296961.7) | 62.5 (44.5, 84.3) | 245862.4 (170886.8, 338806.1) | 43.8 (30.5, 60.4) | -1.2 (-1.3, -1.1) |
| 40-44 years | 330479.8 (245814.1, 435706.7) | 115.4 (85.8, 152.1) | 407080.1 (296132.3, 546759.4) | 81.4 (59.2, 109.3) | -1.2 (-1.3, -1.2) |
| 45-49 years | 493227.0 (375240.9, 634313.6) | 212.4 (161.6, 273.2) | 671654.8 (495273.2, 890085.3) | 141.9 (104.6, 188.0) | -1.3 (-1.4, -1.2) |
| 50-54 years | 869669.3 (671056.1, 1110348.5) | 409.1 (315.7, 522.3) | 1119832.2 (826339.0, 1433322.8) | 251.7 (185.7, 322.2) | -1.6 (-1.7, -1.5) |
| 55-59 years | 1201443.7 (919575.9, 1519688.2) | 648.7 (496.5, 820.6) | 1569981.4 (1184505.4, 1990030.2) | 396.7 (299.3, 502.9) | -1.7 (-1.7, -1.6) |
| 60-64 years | 1567075.6 (1191366.3, 2027205.5) | 975.7 (741.8, 1262.2) | 1911449.9 (1451548.8, 2481074.9) | 597.2 (453.5, 775.2) | -1.8 (-1.8, -1.7) |
| 65-69 years | 1642933.3 (1247851.2, 2114588.6) | 1329.1 (1009.5, 1710.7) | 2259280.1 (1677569.5, 2913186.3) | 819.1 (608.2, 1056.1) | -1.8 (-2.0, -1.7) |
| 70-74 years | 1590178.4 (1116336.9, 2070533.9) | 1878.3 (1318.6, 2445.7) | 2287488.0 (1563038.8, 3069093.4) | 1111.3 (759.4, 1491.0) | -1.8 (-2.0, -1.6) |
| 75-79 years | 1377409.3 (895519.4, 1884047.9) | 2237.7 (1454.8, 3060.7) | 1754555.0 (1147748.0, 2459057.7) | 1330.4 (870.3, 1864.6) | -1.7 (-1.8, -1.6) |
| 80-84 years | 723036.2 (364836.4, 1136402.1) | 2043.9 (1031.3, 3212.4) | 1110717.5 (579739.5, 1708710.3) | 1268.2 (661.9, 1951.0) | -1.6 (-1.7, -1.5) |
| 85-89 years | 436429.3 (214734.5, 673662.5) | 2888.1 (1421.0, 4458.1) | 810766.4 (417012.1, 1233377.2) | 1773.3 (912.1, 2697.6) | -1.7 (-1.8, -1.6) |
| 90-94 years | 172354.3 (93273.9, 256643.7) | 4022.1 (2176.7, 5989.1) | 441098.1 (237097.1, 658293.4) | 2465.7 (1325.4, 3679.8) | -1.7 (-1.8, -1.6) |

**Notes:** ASR: age-standardized rates; DALYs: disability-adjusted life years; EAPCs: estimated annual percentage changes.

**Table S5. Mortality, and DALYs of kidney dysfunction exposure-associated ischemic stroke and its temporal trends from 1990, 2021 by gender, and age.**

|  | **1990** | | **2021** | | **EAPC 95%CI** |
| --- | --- | --- | --- | --- | --- |
|  | **Number** | **ASR, per**  **100,000 persons** | **Number** | **ASR, per**  **100,000 persons** |  |
| **Deaths** |  |  |  |  |  |
| **Gender** |  |  |  |  |  |
| Female | 127800.2 (78360.0, 181794.4) | 12.0 (7.2, 17.3) | 167860.2 (101336.5, 243412.1) | 6.5 (3.9, 9.4) | -2.2 (-2.3, -2.1) |
| Male | 97904.2 (64238.0, 137052.6) | 12.8 (8.0, 18.3) | 161377.2 (102920.0, 227718.5) | 8.1 (5.1, 11.6) | -1.6 (-1.7, -1.5) |
| **Age** |  |  |  |  |  |
| 25-29 years | 152.3 (97.7, 221.0) | 0.0 (0.0, 0.1) | 152.3 (92.1, 226.0) | 0.0 (0.0, 0.0) | -0.9 (-1.1, -0.8) |
| 30-34 years | 275.3 (185.2, 390.7) | 0.1 (0.1, 0.1) | 332.8 (213.3, 466.3) | 0.1 (0.0, 0.1) | -0.9 (-1.0, -0.8) |
| 35-39 years | 503.4 (364.0, 684.1) | 0.1 (0.1, 0.2) | 609.1 (417.1, 851.7) | 0.1 (0.1, 0.2) | -1.0 (-1.1, -0.9) |
| 40-44 years | 879.9 (650.6, 1159.3) | 0.3 (0.2, 0.4) | 1164.1 (835.8, 1569.9) | 0.2 (0.2, 0.3) | -1.2 (-1.3, -1.0) |
| 45-49 years | 1756.9 (1316.1, 2310.8) | 0.8 (0.6, 1.0) | 2566.1 (1880.0, 3364.1) | 0.5 (0.4, 0.7) | -1.2 (-1.3, -1.1) |
| 50-54 years | 4238.3 (3292.5, 5414.1) | 2.0 (1.6, 2.6) | 5641.8 (4210.3, 7306.9) | 1.3 (1.0, 1.6) | -1.5 (-1.6, -1.4) |
| 55-59 years | 7632.5 (5849.9, 9800.9) | 4.1 (3.2, 5.3) | 10319.3 (7749.1, 13206.1) | 2.6 (2.0, 3.3) | -1.6 (-1.8, -1.5) |
| 60-64 years | 17755.4 (13589.9, 23022.5) | 11.1 (8.5, 14.3) | 21900.5 (16619.3, 28171.7) | 6.8 (5.2, 8.8) | -1.8 (-2.0, -1.7) |
| 65-69 years | 24060.9 (18266.5, 30985.1) | 19.5 (14.8, 25.1) | 33604.8 (24729.0, 44026.7) | 12.2 (9.0, 16.0) | -2.0 (-2.2, -1.8) |
| 70-74 years | 37459.3 (26539.8, 48797.7) | 44.3 (31.4, 57.6) | 55047.0 (38546.8, 73542.8) | 26.7 (18.7, 35.7) | -1.9 (-2.1, -1.7) |
| 75-79 years | 46563.1 (30487.0, 63854.7) | 75.6 (49.5, 103.7) | 55354.7 (36142.1, 77401.8) | 42.0 (27.4, 58.7) | -1.8 (-2.0, -1.7) |
| 80-84 years | 38124.8 (19117.7, 59765.5) | 107.8 (54.0, 168.9) | 54801.3 (28493.6, 84330.8) | 62.6 (32.5, 96.3) | -1.9 (-1.9, -1.8) |
| 85-89 years | 31110.2 (15524.6, 48230.9) | 205.9 (102.7, 319.2) | 52546.5 (26568.4, 80495.3) | 114.9 (58.1, 176.1) | -2.0 (-2.1, -1.9) |
| 90-94 years | 15192.2 (8049.3, 22641.5) | 354.5 (187.8, 528.4) | 35197.1 (19010.9, 52417.8) | 196.8 (106.3, 293.0) | -2.1 (-2.2, -2.0) |
| **DALYs** |  |  |  |  |  |
| **Gender** |  |  |  |  |  |
| Female | 2450288.1 (1611151.1, 3379295.4) | 217.2 (140.7, 301.8) | 3283089.7 (2128852.1, 4607659.5) | 128.1 (83.1, 179.8) | -1.9 (-2.0, -1.8) |
| Male | 2168837.2 (1501297.5, 2965425.7) | 242.5 (161.8, 337.1) | 3524980.3 (2374888.9, 4842906.7) | 164.3 (108.6, 227.7) | -1.4 (-1.5, -1.3) |
| **Age** |  |  |  |  |  |
| 25-29 years | 19421.8 (12134.0, 28525.1) | 4.4 (2.7, 6.4) | 21331.4 (13251.1, 31148.9) | 3.6 (2.3, 5.3) | -0.6 (-0.7, -0.6) |
| 30-34 years | 28949.1 (19467.1, 41683.0) | 7.5 (5.1, 10.8) | 37985.8 (24875.4, 55821.9) | 6.3 (4.1, 9.2) | -0.6 (-0.6, -0.6) |
| 35-39 years | 43379.6 (30957.2, 59752.8) | 12.3 (8.8, 17.0) | 57503.1 (39423.1, 80511.7) | 10.3 (7.0, 14.4) | -0.7 (-0.7, -0.6) |
| 40-44 years | 61177.9 (45754.9, 80231.8) | 21.4 (16.0, 28.0) | 88489.5 (62473.2, 120453.1) | 17.7 (12.5, 24.1) | -0.8 (-0.9, -0.7) |
| 45-49 years | 99766.3 (74556.6, 127888.5) | 43.0 (32.1, 55.1) | 159129.2 (115567.7, 207686.6) | 33.6 (24.4, 43.9) | -0.9 (-1.0, -0.8) |
| 50-54 years | 200625.7 (153730.7, 257390.7) | 94.4 (72.3, 121.1) | 291511.6 (214157.2, 375300.9) | 65.5 (48.1, 84.4) | -1.2 (-1.3, -1.1) |
| 55-59 years | 311221.4 (238153.5, 403110.4) | 168.1 (128.6, 217.7) | 457320.6 (340459.9, 595427.7) | 115.6 (86.0, 150.5) | -1.3 (-1.5, -1.2) |
| 60-64 years | 586437.3 (447040.4, 765636.1) | 365.1 (278.3, 476.7) | 765223.3 (574627.7, 1001510.3) | 239.1 (179.5, 312.9) | -1.6 (-1.7, -1.5) |
| 65-69 years | 664296.9 (503385.5, 856529.4) | 537.4 (407.2, 692.9) | 979908.3 (721217.6, 1264864.7) | 355.2 (261.5, 458.6) | -1.8 (-1.9, -1.6) |
| 70-74 years | 823998.6 (585829.6, 1070924.7) | 973.3 (692.0, 1265.0) | 1269891.6 (879721.7, 1711505.0) | 616.9 (427.4, 831.5) | -1.7 (-1.9, -1.6) |
| 75-79 years | 807280.7 (535905.8, 1110118.5) | 1311.5 (870.6, 1803.4) | 1014337.5 (665197.5, 1407711.8) | 769.1 (504.4, 1067.4) | -1.7 (-1.8, -1.6) |
| 80-84 years | 509489.1 (256417.0, 802056.2) | 1440.2 (724.8, 2267.2) | 763960.4 (400008.2, 1174897.4) | 872.3 (456.7, 1341.5) | -1.7 (-1.8, -1.7) |
| 85-89 years | 326071.4 (162418.6, 504395.0) | 2157.8 (1074.8, 3337.9) | 572675.9 (290945.3, 869853.6) | 1252.5 (636.3, 1902.5) | -1.9 (-2.0, -1.8) |
| 90-94 years | 137009.7 (72968.8, 204272.4) | 3197.3 (1702.8, 4766.9) | 328802.0 (177074.7, 489009.7) | 1838.0 (989.8, 2733.5) | -2.0 (-2.1, -1.9) |

**Notes:** ASR: age-standardized rates; DALYs: disability-adjusted life years; EAPCs: estimated annual percentage changes.

**Table S6. Mortality, and DALYs of kidney dysfunction exposure-associated intracerebral hemorrhage and its temporal trends from 1990, 2021 by gender, and age.**

|  | **1990** | | **2021** | | **EAPC 95%CI** |
| --- | --- | --- | --- | --- | --- |
|  | **Number** | **ASR, per**  **100,000 persons** | **Number** | **ASR, per**  **100,000 persons** |  |
| **Deaths** |  |  |  |  |  |
| **Gender** |  |  |  |  |  |
| Female | 120700.2 (82546.7, 162621.5) | 10.4 (7.1, 14.1) | 151061.4 (100359.0, 208869.9) | 5.9 (3.9, 8.2) | -1.9 (-2.0, -1.8) |
| Male | 119396.7 (83575.1, 161461.9) | 12.4 (8.4, 17.1) | 173884.8 (117797.0, 237684.9) | 8.0 (5.3, 11.0) | -1.5 (-1.6, -1.3) |
| **Age** |  |  |  |  |  |
| 25-29 years | 937.4 (602.2, 1360.6) | 0.2 (0.1, 0.3) | 817.1 (506.0, 1202.4) | 0.1 (0.1, 0.2) | -1.5 (-1.6, -1.3) |
| 30-34 years | 1721.6 (1171.9, 2400.6) | 0.5 (0.3, 0.6) | 1835.9 (1189.4, 2560.1) | 0.3 (0.2, 0.4) | -1.3 (-1.4, -1.2) |
| 35-39 years | 3159.0 (2247.7, 4251.3) | 0.9 (0.6, 1.2) | 3339.1 (2309.1, 4596.6) | 0.6 (0.4, 0.8) | -1.3 (-1.5, -1.2) |
| 40-44 years | 5378.8 (3970.0, 7080.2) | 1.9 (1.4, 2.5) | 6327.1 (4500.8, 8655.5) | 1.3 (0.9, 1.7) | -1.4 (-1.5, -1.3) |
| 45-49 years | 8803.5 (6773.4, 11312.2) | 3.8 (2.9, 4.9) | 11385.7 (8398.4, 14968.1) | 2.4 (1.8, 3.2) | -1.4 (-1.5, -1.4) |
| 50-54 years | 16940.9 (13043.2, 21502.3) | 8.0 (6.1, 10.1) | 20825.4 (15379.3, 26820.1) | 4.7 (3.5, 6.0) | -1.8 (-1.8, -1.7) |
| 55-59 years | 25848.9 (19810.6, 32861.1) | 14.0 (10.7, 17.7) | 32062.3 (24365.6, 40776.3) | 8.1 (6.2, 10.3) | -1.8 (-1.9, -1.8) |
| 60-64 years | 33229.0 (25246.2, 42888.0) | 20.7 (15.7, 26.7) | 38570.7 (29320.1, 50259.6) | 12.1 (9.2, 15.7) | -1.9 (-2.0, -1.8) |
| 65-69 years | 39587.0 (29607.4, 51016.7) | 32.0 (24.0, 41.3) | 51435.6 (37827.9, 66333.6) | 18.7 (13.7, 24.1) | -1.9 (-2.1, -1.7) |
| 70-74 years | 37727.5 (25964.5, 49786.1) | 44.6 (30.7, 58.8) | 49688.8 (34187.9, 65874.1) | 24.1 (16.6, 32.0) | -1.9 (-2.1, -1.7) |
| 75-79 years | 35145.1 (22613.9, 48185.2) | 57.1 (36.7, 78.3) | 45392.5 (29278.7, 63676.1) | 34.4 (22.2, 48.3) | -1.7 (-1.8, -1.5) |
| 80-84 years | 16760.3 (8394.4, 26328.4) | 47.4 (23.7, 74.4) | 27150.4 (14134.1, 42386.1) | 31.0 (16.1, 48.4) | -1.3 (-1.5, -1.2) |
| 85-89 years | 10865.4 (5298.0, 17029.5) | 71.9 (35.1, 112.7) | 23455.4 (12289.8, 36141.6) | 51.3 (26.9, 79.1) | -1.1 (-1.4, -0.9) |
| 90-94 years | 3992.6 (2139.7, 6025.4) | 93.2 (49.9, 140.6) | 12660.4 (6700.4, 18921.5) | 70.8 (37.5, 105.8) | -0.9 (-1.0, -0.7) |
| **DALYs** |  |  |  |  |  |
| **Gender** |  |  |  |  |  |
| Female | 2987260.3 (2127128.4, 3937780.1) | 251.4 (178.3, 332.1) | 3533605.7 (2450793.2, 4763383.4) | 139.9 (97.1, 188.7) | -2.0 (-2.1, -1.9) |
| Male | 3237344.5 (2331630.2, 4325669.6) | 304.5 (215.8, 409.8) | 4478935.0 (3151692.3, 6008932.1) | 195.6 (136.3, 263.8) | -1.5 (-1.6, -1.4) |
| **Age** |  |  |  |  |  |
| 25-29 years | 64344.1 (41058.8, 92039.4) | 14.5 (9.3, 20.8) | 56732.9 (34552.9, 82914.7) | 9.6 (5.9, 14.1) | -1.4 (-1.6, -1.3) |
| 30-34 years | 106707.5 (73019.7, 149093.7) | 27.7 (19.0, 38.7) | 114794.9 (74451.5, 160635.6) | 19.0 (12.3, 26.6) | -1.3 (-1.4, -1.2) |
| 35-39 years | 176692.0 (125520.4, 239140.8) | 50.2 (35.6, 67.9) | 188359.3 (130590.7, 259641.5) | 33.6 (23.3, 46.3) | -1.3 (-1.4, -1.2) |
| 40-44 years | 269301.9 (198785.6, 354606.5) | 94.0 (69.4, 123.8) | 318590.6 (228445.8, 432831.4) | 63.7 (45.7, 86.5) | -1.3 (-1.4, -1.3) |
| 45-49 years | 393460.7 (301246.8, 507325.0) | 169.5 (129.7, 218.5) | 512525.6 (377722.4, 679635.2) | 108.2 (79.8, 143.5) | -1.4 (-1.5, -1.3) |
| 50-54 years | 669043.6 (512899.5, 849416.2) | 314.7 (241.3, 399.6) | 828320.5 (613411.1, 1065762.6) | 186.2 (137.9, 239.5) | -1.7 (-1.8, -1.7) |
| 55-59 years | 890222.3 (679006.4, 1128112.5) | 480.7 (366.6, 609.1) | 1112660.8 (841239.9, 1415716.9) | 281.2 (212.6, 357.8) | -1.8 (-1.9, -1.8) |
| 60-64 years | 980638.4 (745330.5, 1263604.6) | 610.6 (464.1, 786.8) | 1146226.5 (871815.4, 1486256.1) | 358.1 (272.4, 464.4) | -1.9 (-2.0, -1.8) |
| 65-69 years | 978636.3 (732937.2, 1260864.5) | 791.7 (593.0, 1020.0) | 1279371.8 (943052.2, 1646928.5) | 463.8 (341.9, 597.1) | -1.9 (-2.0, -1.7) |
| 70-74 years | 766179.8 (527193.5, 1014942.1) | 905.0 (622.7, 1198.8) | 1017596.4 (696907.4, 1348435.7) | 494.4 (338.6, 655.1) | -1.9 (-2.1, -1.7) |
| 75-79 years | 570128.6 (366473.7, 783249.2) | 926.2 (595.4, 1272.4) | 740217.6 (477717.5, 1040194.6) | 561.3 (362.2, 788.7) | -1.7 (-1.8, -1.5) |
| 80-84 years | 213547.1 (106967.9, 336337.9) | 603.7 (302.4, 950.8) | 346757.1 (181257.6, 540756.0) | 395.9 (207.0, 617.4) | -1.3 (-1.5, -1.2) |
| 85-89 years | 110357.9 (53915.4, 173004.3) | 730.3 (356.8, 1144.9) | 238090.5 (125307.5, 367406.9) | 520.7 (274.1, 803.6) | -1.1 (-1.4, -0.9) |
| 90-94 years | 35344.6 (18938.1, 53198.2) | 824.8 (441.9, 1241.4) | 112296.1 (59653.0, 167725.0) | 627.7 (333.5, 937.6) | -0.8 (-1.0, -0.7) |

**Notes:** ASR: age-standardized rates; DALYs: disability-adjusted life years; EAPCs: estimated annual percentage changes.

**Table S7. Mortality, and DALYs of future forecasts in kidney dysfunction exposure-associated stroke using bayesian age-period-cohort model.**

| **Year** | **Sex** | **Number** | **ASR** |
| --- | --- | --- | --- |
| **Deaths** |  |  |  |
| 2022 | Both | 672580.3 (646173.7, 698986.9) | 15.8 (15.4, 16.2) |
| 2023 | Both | 685650.7 (649461.5, 721840.0) | 15.7 (15.0, 16.4) |
| 2024 | Both | 699993.9 (649077.2, 750910.6) | 15.6 (14.5, 16.6) |
| 2025 | Both | 715245.5 (645530.2, 784960.8) | 15.5 (14.0, 16.9) |
| 2026 | Both | 730781.7 (638706.0, 822857.3) | 15.4 (13.5, 17.3) |
| 2027 | Both | 746167.7 (628422.7, 863912.7) | 15.3 (12.9, 17.6) |
| 2028 | Both | 761941.3 (615293.5, 908589.1) | 15.1 (12.2, 18.1) |
| 2029 | Both | 778819.6 (599879.0, 957760.2) | 15.0 (11.6, 18.5) |
| 2030 | Both | 796723.2 (581969.0, 1011477.3) | 15.0 (10.9, 19.0) |
| 2031 | Both | 815308.3 (561106.3, 1069510.4) | 14.9 (10.2, 19.5) |
| 2032 | Both | 834369.1 (536920.5, 1131817.6) | 14.8 (9.5, 20.0) |
| 2033 | Both | 854536.7 (509624.3, 1199449.0) | 14.7 (8.8, 20.6) |
| 2034 | Both | 876418.7 (479235.1, 1273602.2) | 14.6 (8.1, 21.2) |
| 2035 | Both | 899855.8 (445203.6, 1354507.9) | 14.6 (7.3, 21.8) |
| 2036 | Both | 924447.4 (406828.2, 1442066.6) | 14.5 (6.5, 22.4) |
| 2037 | Both | 949914.4 (363467.7, 1536361.0) | 14.4 (5.7, 23.1) |
| 2038 | Both | 977110.5 (314929.1, 1639291.8) | 14.4 (4.9, 23.8) |
| 2039 | Both | 1006638.1 (260648.4, 1752627.8) | 14.3 (4.1, 24.5) |
| 2040 | Both | 1037930.8 (199568.8, 1876292.7) | 14.3 (3.3, 25.2) |
| 2041 | Both | 1070306.1 (130683.6, 2009972.5) | 14.2 (2.4, 26.0) |
| 2042 | Both | 1103277.4 (53144.7, 2153668.6) | 14.2 (1.6, 26.8) |
| 2043 | Both | 1137183.6 (0.0, 2308949.5) | 14.1 (0.7, 27.5) |
| 2044 | Both | 1172920.7 (0.0, 2478834.0) | 14.1 (-0.2, 28.3) |
| 2045 | Both | 1210748.0 (0.0, 2665434.2) | 14.0 (-1.1, 29.1) |
| 2022 | Female | 327322.4 (315549.8, 339095.1) | 12.2 (11.9, 12.5) |
| 2023 | Female | 332768.7 (316219.8, 349317.7) | 12.1 (11.6, 12.6) |
| 2024 | Female | 338758.5 (315127.1, 362390.0) | 11.9 (11.2, 12.7) |
| 2025 | Female | 345194.6 (312625.4, 377763.9) | 11.8 (10.7, 12.8) |
| 2026 | Female | 351773.3 (308652.7, 394893.9) | 11.6 (10.2, 13.0) |
| 2027 | Female | 358276.1 (303104.6, 413447.6) | 11.5 (9.8, 13.2) |
| 2028 | Female | 364922.4 (296250.5, 433594.3) | 11.3 (9.2, 13.4) |
| 2029 | Female | 372058.6 (288374.0, 455743.2) | 11.2 (8.7, 13.7) |
| 2030 | Female | 379683.2 (279414.6, 479951.8) | 11.1 (8.2, 14.0) |
| 2031 | Female | 387602.2 (269141.2, 506063.2) | 10.9 (7.6, 14.2) |
| 2032 | Female | 395709.1 (257379.9, 534038.3) | 10.8 (7.1, 14.5) |
| 2033 | Female | 404328.1 (244266.4, 564389.8) | 10.7 (6.5, 14.8) |
| 2034 | Female | 413747.4 (229827.4, 597667.3) | 10.6 (6.0, 15.2) |
| 2035 | Female | 423882.6 (213816.7, 633948.5) | 10.4 (5.4, 15.5) |
| 2036 | Female | 434486.3 (195895.4, 673077.2) | 10.3 (4.8, 15.8) |
| 2037 | Female | 445404.1 (175778.0, 715030.2) | 10.2 (4.3, 16.2) |
| 2038 | Female | 457116.5 (153444.2, 760788.8) | 10.1 (3.7, 16.5) |
| 2039 | Female | 469899.7 (128661.9, 811137.5) | 10.0 (3.1, 16.9) |
| 2040 | Female | 483400.8 (100953.3, 865848.2) | 9.9 (2.5, 17.3) |
| 2041 | Female | 497189.8 (69884.0, 924513.1) | 9.8 (1.9, 17.7) |
| 2042 | Female | 510989.9 (35098.4, 986955.9) | 9.7 (1.3, 18.0) |
| 2043 | Female | 525016.2 (0.0, 1053910.2) | 9.6 (0.8, 18.4) |
| 2044 | Female | 539707.6 (0.0, 1126703.3) | 9.5 (0.2, 18.8) |
| 2045 | Female | 555145.6 (0.0, 1206098.2) | 9.4 (-0.4, 19.2) |
| 2022 | Male | 345257.9 (330623.9, 359891.8) | 13.9 (13.4, 14.4) |
| 2023 | Male | 352882.0 (333241.7, 372522.2) | 13.8 (13.0, 14.5) |
| 2024 | Male | 361235.4 (333950.1, 388520.6) | 13.6 (12.6, 14.6) |
| 2025 | Male | 370050.9 (332904.8, 407197.0) | 13.5 (12.2, 14.8) |
| 2026 | Male | 379008.3 (330053.3, 427963.4) | 13.4 (11.7, 15.1) |
| 2027 | Male | 387891.6 (325318.1, 450465.1) | 13.3 (11.2, 15.4) |
| 2028 | Male | 397018.9 (319043.0, 474994.8) | 13.2 (10.6, 15.7) |
| 2029 | Male | 406761.1 (311505.1, 502017.0) | 13.1 (10.1, 16.1) |
| 2030 | Male | 417040.0 (302554.4, 531525.6) | 13.0 (9.5, 16.5) |
| 2031 | Male | 427706.2 (291965.1, 563447.2) | 12.9 (8.9, 17.0) |
| 2032 | Male | 438660.0 (279540.6, 597779.3) | 12.9 (8.3, 17.5) |
| 2033 | Male | 450208.6 (265357.9, 635059.3) | 12.8 (7.6, 18.0) |
| 2034 | Male | 462671.3 (249407.7, 675934.9) | 12.8 (7.0, 18.6) |
| 2035 | Male | 475973.2 (231386.9, 720559.4) | 12.8 (6.3, 19.2) |
| 2036 | Male | 489961.1 (210932.8, 768989.4) | 12.8 (5.6, 19.9) |
| 2037 | Male | 504510.3 (187689.7, 821330.9) | 12.8 (4.9, 20.7) |
| 2038 | Male | 519994.0 (161484.9, 878503.0) | 12.8 (4.1, 21.5) |
| 2039 | Male | 536738.4 (131986.5, 941490.3) | 12.8 (3.3, 22.3) |
| 2040 | Male | 554530.0 (98615.5, 1010444.5) | 12.9 (2.5, 23.3) |
| 2041 | Male | 573116.3 (60799.5, 1085459.4) | 13.0 (1.6, 24.3) |
| 2042 | Male | 592287.6 (18046.3, 1166712.7) | 13.0 (0.6, 25.5) |
| 2043 | Male | 612167.4 (0.0, 1255039.3) | 13.2 (0.0, 26.7) |
| 2044 | Male | 633213.1 (0.0, 1352130.8) | 13.3 (0.0, 28.1) |
| 2045 | Male | 655602.4 (0.0, 1459336.0) | 13.4 (0.0, 29.6) |
| **DALYs** |  |  |  |
| 2022 | Both | 15222707.0 (14663042.6, 15782371.5) | 308.7 (297.3, 320.0) |
| 2023 | Both | 15531785.9 (14777302.0, 16286269.9) | 306.8 (291.9, 321.7) |
| 2024 | Both | 15857321.8 (14805222.6, 16909420.9) | 305.0 (284.7, 325.2) |
| 2025 | Both | 16194487.8 (14760029.4, 17628946.1) | 303.3 (276.5, 330.2) |
| 2026 | Both | 16536259.2 (14645787.5, 18426731.0) | 301.8 (267.3, 336.3) |
| 2027 | Both | 16878357.1 (14463619.5, 19293094.6) | 300.4 (257.4, 343.4) |
| 2028 | Both | 17225211.9 (14220739.7, 20229684.1) | 299.2 (247.0, 351.4) |
| 2029 | Both | 17584641.4 (13924140.4, 21245142.4) | 298.1 (236.0, 360.2) |
| 2030 | Both | 17955499.9 (13571475.0, 22339524.9) | 297.2 (224.6, 369.8) |
| 2031 | Both | 18335815.4 (13158687.6, 23512943.2) | 296.5 (212.7, 380.2) |
| 2032 | Both | 18726190.6 (12682830.4, 24769550.8) | 296.0 (200.4, 391.5) |
| 2033 | Both | 19132860.7 (12145148.3, 26120573.1) | 295.7 (187.6, 403.7) |
| 2034 | Both | 19561970.9 (11544660.9, 27579280.8) | 295.6 (174.3, 416.8) |
| 2035 | Both | 20010518.9 (10873301.4, 29147736.3) | 295.8 (160.6, 430.9) |
| 2036 | Both | 20475546.4 (10122481.0, 30828611.8) | 296.2 (146.3, 446.1) |
| 2037 | Both | 20956673.6 (9284335.3, 32629012.0) | 296.9 (131.3, 462.4) |
| 2038 | Both | 21462520.4 (8355001.3, 34570039.5) | 297.8 (115.7, 480.0) |
| 2039 | Both | 21999824.1 (7326560.4, 36673087.8) | 299.1 (99.3, 498.9) |
| 2040 | Both | 22561547.0 (6184205.0, 38938889.0) | 300.6 (82.1, 519.2) |
| 2041 | Both | 23142197.0 (4913437.7, 41370956.3) | 302.5 (63.8, 541.1) |
| 2042 | Both | 23739303.0 (3499902.6, 43978703.3) | 304.7 (44.5, 565.0) |
| 2043 | Both | 24355480.8 (1935649.5, 46780947.5) | 307.3 (23.9, 590.8) |
| 2044 | Both | 25000966.9 (615138.3, 49814978.1) | 310.2 (7.3, 618.8) |
| 2045 | Both | 25678609.5 (0.0, 53108368.9) | 313.5 (0.0, 649.2) |
| 2022 | Female | 6978009.8 (6750671.3, 7205348.2) | 264.5 (259.2, 269.8) |
| 2023 | Female | 7110739.9 (6795368.0, 7426111.7) | 262.4 (252.9, 271.8) |
| 2024 | Female | 7250665.7 (6802352.2, 7698979.3) | 260.3 (245.7, 274.9) |
| 2025 | Female | 7396111.4 (6778639.3, 8013583.4) | 258.3 (237.8, 278.7) |
| 2026 | Female | 7543585.2 (6725596.7, 8361573.6) | 256.3 (229.4, 283.2) |
| 2027 | Female | 7690810.8 (6643195.2, 8738426.5) | 254.4 (220.5, 288.3) |
| 2028 | Female | 7839576.1 (6534522.3, 9144629.9) | 252.5 (211.2, 293.8) |
| 2029 | Female | 7993618.0 (6403012.7, 9584223.3) | 250.7 (201.5, 299.8) |
| 2030 | Female | 8152568.9 (6247867.7, 10057270.1) | 248.9 (191.6, 306.2) |
| 2031 | Female | 8315231.4 (6067186.4, 10563276.3) | 247.1 (181.3, 312.9) |
| 2032 | Female | 8481727.3 (5859701.7, 11103752.9) | 245.4 (170.7, 320.0) |
| 2033 | Female | 8655193.9 (5626509.3, 11683878.5) | 243.7 (159.9, 327.5) |
| 2034 | Female | 8838429.2 (5367524.9, 12309333.4) | 242.1 (148.9, 335.3) |
| 2035 | Female | 9029686.1 (5079250.1, 12980122.2) | 240.5 (137.7, 343.3) |
| 2036 | Female | 9227077.5 (4757977.7, 13696177.4) | 238.9 (126.3, 351.6) |
| 2037 | Female | 9430020.9 (4400508.2, 14459533.6) | 237.4 (114.7, 360.1) |
| 2038 | Female | 9643154.3 (4006217.2, 15280091.4) | 235.9 (102.9, 368.9) |
| 2039 | Female | 9869503.0 (3572338.9, 16166667.1) | 234.5 (91.0, 377.9) |
| 2040 | Female | 10104920.1 (3092724.5, 17117115.7) | 233.0 (78.9, 387.1) |
| 2041 | Female | 10345997.6 (2561723.3, 18130271.9) | 231.5 (66.6, 396.4) |
| 2042 | Female | 10590694.8 (1974085.5, 19207304.0) | 230.1 (54.3, 406.0) |
| 2043 | Female | 10840226.8 (1325867.9, 20354909.4) | 228.7 (41.8, 415.6) |
| 2044 | Female | 11099362.7 (615138.3, 21587912.7) | 227.3 (29.2, 425.4) |
| 2045 | Female | 11369356.6 (0.0, 22916137.0) | 225.8 (16.4, 435.3) |
| 2022 | Male | 8244697.3 (7912371.2, 8577023.3) | 356.7 (348.3, 365.0) |
| 2023 | Male | 8421046.1 (7981934.0, 8860158.2) | 354.9 (340.6, 369.3) |
| 2024 | Male | 8606656.0 (8002870.4, 9210441.7) | 353.3 (331.5, 375.1) |
| 2025 | Male | 8798376.4 (7981390.1, 9615362.8) | 351.7 (321.3, 382.2) |
| 2026 | Male | 8992674.0 (7920190.8, 10065157.3) | 350.2 (310.2, 390.2) |
| 2027 | Male | 9187546.2 (7820424.4, 10554668.1) | 348.8 (298.4, 399.1) |
| 2028 | Male | 9385635.8 (7686217.4, 11085054.2) | 347.5 (286.0, 408.9) |
| 2029 | Male | 9591023.4 (7521127.6, 11660919.1) | 346.2 (272.9, 419.4) |
| 2030 | Male | 9802931.1 (7323607.3, 12282254.8) | 344.9 (259.3, 430.6) |
| 2031 | Male | 10020584.0 (7091501.2, 12949666.9) | 343.8 (245.1, 442.4) |
| 2032 | Male | 10244463.3 (6823128.7, 13665797.9) | 342.7 (230.5, 454.8) |
| 2033 | Male | 10477666.8 (6518638.9, 14436694.6) | 341.6 (215.4, 467.9) |
| 2034 | Male | 10723541.7 (6177136.0, 15269947.3) | 340.7 (199.8, 481.5) |
| 2035 | Male | 10980832.8 (5794051.3, 16167614.2) | 339.8 (183.8, 495.7) |
| 2036 | Male | 11248468.9 (5364503.3, 17132434.4) | 338.9 (167.4, 510.4) |
| 2037 | Male | 11526652.7 (4883827.1, 18169478.4) | 338.1 (150.6, 525.6) |
| 2038 | Male | 11819366.1 (4348784.1, 19289948.1) | 337.4 (133.4, 541.3) |
| 2039 | Male | 12130321.1 (3754221.6, 20506420.7) | 336.7 (115.8, 557.5) |
| 2040 | Male | 12456626.9 (3091480.5, 21821773.3) | 335.9 (97.8, 574.1) |
| 2041 | Male | 12796199.4 (2351714.4, 23240684.4) | 335.2 (79.4, 591.1) |
| 2042 | Male | 13148608.2 (1525817.1, 24771399.3) | 334.6 (60.7, 608.5) |
| 2043 | Male | 13515254.0 (609781.6, 26426038.1) | 333.9 (41.6, 626.3) |
| 2044 | Male | 13901604.1 (0.0, 28227065.4) | 333.3 (22.2, 644.3) |
| 2045 | Male | 14309252.9 (0.0, 30192231.9) | 332.5 (2.4, 662.7) |

**Notes:** ASR: age-standardized rates; DALYs: disability-adjusted life years.

**Table S8. Mortality, and DALYs of future forecasts in kidney dysfunction exposure-associated ischemic stroke using bayesian age-period-cohort model.**

| **Year** | **Sex** | **Number** | **ASR** |
| --- | --- | --- | --- |
| **Deaths** |  |  |  |
| 2022 | Both | 339950.9 (325185.4, 354716.5) | 7.1 (6.8, 7.4) |
| 2023 | Both | 347315.0 (326489.4, 368140.6) | 7.1 (6.6, 7.5) |
| 2024 | Both | 355641.4 (325851.7, 385431.0) | 7.0 (6.4, 7.6) |
| 2025 | Both | 364623.5 (323455.0, 405792.0) | 6.9 (6.2, 7.7) |
| 2026 | Both | 373752.0 (319043.8, 428460.2) | 6.9 (5.9, 7.9) |
| 2027 | Both | 382849.8 (312514.3, 453185.2) | 6.8 (5.6, 8.1) |
| 2028 | Both | 392445.5 (304380.8, 480510.3) | 6.8 (5.3, 8.3) |
| 2029 | Both | 402984.1 (294919.4, 511048.7) | 6.8 (4.9, 8.6) |
| 2030 | Both | 414371.4 (283894.2, 544848.5) | 6.7 (4.6, 8.8) |
| 2031 | Both | 426240.3 (270843.9, 581636.7) | 6.7 (4.3, 9.1) |
| 2032 | Both | 438516.9 (255490.2, 621543.6) | 6.7 (3.9, 9.5) |
| 2033 | Both | 451859.3 (238029.6, 665688.9) | 6.7 (3.5, 9.8) |
| 2034 | Both | 466696.1 (218338.6, 715053.5) | 6.7 (3.1, 10.2) |
| 2035 | Both | 482935.9 (195912.1, 769959.8) | 6.7 (2.7, 10.7) |
| 2036 | Both | 500218.7 (170087.7, 830349.7) | 6.7 (2.3, 11.2) |
| 2037 | Both | 518413.1 (140276.5, 896556.6) | 6.8 (1.8, 11.7) |
| 2038 | Both | 538282.2 (106127.8, 970460.3) | 6.8 (1.3, 12.3) |
| 2039 | Both | 560219.6 (66919.7, 1053586.5) | 6.9 (0.8, 12.9) |
| 2040 | Both | 583830.0 (21653.6, 1146172.0) | 7.0 (0.3, 13.7) |
| 2041 | Both | 608584.3 (0.0, 1248213.9) | 7.1 (0.0, 14.5) |
| 2042 | Both | 634240.8 (0.0, 1360315.8) | 7.2 (0.0, 15.4) |
| 2043 | Both | 661189.3 (0.0, 1484469.6) | 7.3 (0.0, 16.4) |
| 2044 | Both | 690057.5 (0.0, 1623613.3) | 7.4 (0.0, 17.5) |
| 2045 | Both | 721094.6 (0.0, 1780305.3) | 7.6 (0.0, 18.7) |
| 2022 | Female | 172704.6 (165742.1, 179667.0) | 6.4 (6.2, 6.6) |
| 2023 | Female | 175816.5 (165744.7, 185888.3) | 6.3 (6.0, 6.6) |
| 2024 | Female | 179364.5 (164770.4, 193958.5) | 6.2 (5.8, 6.7) |
| 2025 | Female | 183258.9 (162990.8, 203527.0) | 6.2 (5.5, 6.8) |
| 2026 | Female | 187239.1 (160271.2, 214207.0) | 6.1 (5.2, 7.0) |
| 2027 | Female | 191208.5 (156547.3, 225869.7) | 6.0 (5.0, 7.1) |
| 2028 | Female | 195423.0 (152075.8, 238770.1) | 6.0 (4.7, 7.3) |
| 2029 | Female | 200110.6 (147005.3, 253215.9) | 5.9 (4.4, 7.4) |
| 2030 | Female | 205244.7 (141237.8, 269251.6) | 5.8 (4.0, 7.6) |
| 2031 | Female | 210603.0 (134520.1, 286685.9) | 5.8 (3.7, 7.8) |
| 2032 | Female | 216128.6 (126705.7, 305551.5) | 5.7 (3.4, 8.0) |
| 2033 | Female | 222193.2 (117931.0, 326455.4) | 5.6 (3.1, 8.2) |
| 2034 | Female | 229014.3 (108144.7, 349884.0) | 5.6 (2.7, 8.5) |
| 2035 | Female | 236547.8 (97104.5, 375991.1) | 5.5 (2.4, 8.7) |
| 2036 | Female | 244576.1 (84475.9, 404676.3) | 5.5 (2.1, 8.9) |
| 2037 | Female | 253009.9 (69972.9, 436051.0) | 5.5 (1.7, 9.2) |
| 2038 | Female | 262307.2 (53460.6, 471164.6) | 5.4 (1.4, 9.5) |
| 2039 | Female | 272648.6 (34590.5, 510734.9) | 5.4 (1.0, 9.8) |
| 2040 | Female | 283762.3 (12874.3, 554713.6) | 5.3 (0.7, 10.0) |
| 2041 | Female | 295301.5 (0.0, 602887.9) | 5.3 (0.3, 10.3) |
| 2042 | Female | 307100.2 (0.0, 655393.4) | 5.3 (-0.1, 10.6) |
| 2043 | Female | 319406.2 (0.0, 713246.0) | 5.2 (-0.5, 10.9) |
| 2044 | Female | 332529.3 (0.0, 777802.4) | 5.2 (-0.8, 11.2) |
| 2045 | Female | 346547.4 (0.0, 850102.9) | 5.2 (-1.2, 11.6) |
| 2022 | Male | 167246.4 (159443.2, 175049.5) | 8.0 (7.8, 8.2) |
| 2023 | Male | 171498.5 (160744.7, 182252.3) | 7.9 (7.5, 8.3) |
| 2024 | Male | 176276.9 (161081.3, 191472.5) | 7.9 (7.3, 8.5) |
| 2025 | Male | 181364.6 (160464.3, 202265.0) | 7.8 (7.0, 8.7) |
| 2026 | Male | 186513.0 (158772.7, 214253.3) | 7.8 (6.7, 8.9) |
| 2027 | Male | 191641.3 (155967.0, 227315.6) | 7.7 (6.3, 9.1) |
| 2028 | Male | 197022.5 (152304.9, 241740.2) | 7.7 (6.0, 9.4) |
| 2029 | Male | 202873.5 (147914.1, 257832.9) | 7.6 (5.6, 9.7) |
| 2030 | Male | 209126.7 (142656.4, 275596.9) | 7.6 (5.2, 10.0) |
| 2031 | Male | 215637.3 (136323.8, 294950.8) | 7.6 (4.8, 10.3) |
| 2032 | Male | 222388.3 (128784.5, 315992.2) | 7.5 (4.4, 10.6) |
| 2033 | Male | 229666.1 (120098.6, 339233.5) | 7.5 (4.0, 11.0) |
| 2034 | Male | 237681.7 (110194.0, 365169.5) | 7.5 (3.6, 11.3) |
| 2035 | Male | 246388.1 (98807.6, 393968.7) | 7.4 (3.2, 11.7) |
| 2036 | Male | 255642.6 (85611.8, 425673.3) | 7.4 (2.7, 12.1) |
| 2037 | Male | 265403.3 (70303.7, 460505.7) | 7.4 (2.3, 12.5) |
| 2038 | Male | 275975.0 (52667.2, 499295.7) | 7.4 (1.8, 13.0) |
| 2039 | Male | 287571.0 (32329.2, 542851.7) | 7.4 (1.3, 13.5) |
| 2040 | Male | 300067.7 (8779.3, 591458.4) | 7.4 (0.8, 13.9) |
| 2041 | Male | 313282.7 (0.0, 645326.0) | 7.4 (0.3, 14.4) |
| 2042 | Male | 327140.6 (0.0, 704922.4) | 7.4 (-0.2, 14.9) |
| 2043 | Male | 341783.1 (0.0, 771223.7) | 7.4 (-0.7, 15.4) |
| 2044 | Male | 357528.3 (0.0, 845810.9) | 7.4 (-1.3, 16.0) |
| 2045 | Male | 374547.2 (0.0, 930202.3) | 7.4 (-1.8, 16.5) |
| **DALYs** |  |  |  |
| 2022 | Both | 6993249.5 (6732525.2, 7253973.8) | 143.1 (137.8, 148.4) |
| 2023 | Both | 7161219.8 (6793531.9, 7528907.7) | 142.4 (135.0, 149.7) |
| 2024 | Both | 7344008.2 (6814826.1, 7873190.3) | 141.7 (131.5, 151.9) |
| 2025 | Both | 7536688.1 (6800996.8, 8272379.4) | 141.1 (127.4, 154.9) |
| 2026 | Both | 7731379.5 (6749286.6, 8713472.4) | 140.6 (122.7, 158.4) |
| 2027 | Both | 7927258.0 (6660399.3, 9194116.7) | 140.1 (117.7, 162.5) |
| 2028 | Both | 8130698.4 (6540967.3, 9720429.6) | 139.8 (112.5, 167.2) |
| 2029 | Both | 8346428.5 (6393996.8, 10298860.3) | 139.7 (107.0, 172.3) |
| 2030 | Both | 8573082.6 (6216450.9, 10929714.4) | 139.6 (101.2, 178.0) |
| 2031 | Both | 8805912.6 (6002267.2, 11609558.1) | 139.6 (95.1, 184.1) |
| 2032 | Both | 9047797.6 (5750307.2, 12345288.0) | 139.8 (88.8, 190.7) |
| 2033 | Both | 9306689.0 (5462804.7, 13150573.3) | 140.1 (82.2, 198.0) |
| 2034 | Both | 9586694.1 (5137363.9, 14036024.4) | 140.6 (75.3, 205.9) |
| 2035 | Both | 9885194.8 (4766652.3, 15003737.2) | 141.3 (68.1, 214.6) |
| 2036 | Both | 10196228.2 (4341418.9, 16051037.5) | 142.2 (60.5, 223.9) |
| 2037 | Both | 10521721.8 (3855759.8, 17187683.9) | 143.2 (52.4, 234.1) |
| 2038 | Both | 10869711.3 (3305749.6, 18433673.0) | 144.5 (43.8, 245.2) |
| 2039 | Both | 11245322.1 (2683118.8, 19807525.3) | 146.0 (34.7, 257.4) |
| 2040 | Both | 11644497.1 (1975902.2, 21313824.2) | 147.8 (24.9, 270.7) |
| 2041 | Both | 12060812.1 (1172027.1, 22953303.3) | 149.7 (14.4, 285.2) |
| 2042 | Both | 12495670.7 (458471.4, 24741921.7) | 152.0 (5.4, 301.1) |
| 2043 | Both | 12952316.4 (21075.9, 26700147.7) | 154.5 (0.2, 318.7) |
| 2044 | Both | 13437746.8 (0.0, 28860580.4) | 157.2 (0.0, 338.1) |
| 2045 | Both | 13954991.2 (0.0, 31252261.0) | 160.3 (0.0, 359.4) |
| 2022 | Female | 3367876.3 (3257414.1, 3478338.5) | 126.4 (123.7, 129.1) |
| 2023 | Female | 3443453.3 (3282875.4, 3604031.2) | 125.5 (120.6, 130.4) |
| 2024 | Female | 3526284.9 (3291024.0, 3761545.8) | 124.7 (117.0, 132.4) |
| 2025 | Female | 3614743.3 (3284795.0, 3944691.6) | 124.0 (113.2, 134.9) |
| 2026 | Female | 3704291.8 (3261956.8, 4146626.9) | 123.3 (109.0, 137.7) |
| 2027 | Female | 3794048.6 (3222281.3, 4365815.9) | 122.6 (104.5, 140.7) |
| 2028 | Female | 3887292.2 (3169174.5, 4605410.0) | 122.0 (99.8, 144.2) |
| 2029 | Female | 3986809.8 (3104557.0, 4869062.5) | 121.4 (94.9, 147.9) |
| 2030 | Female | 4092310.5 (3027291.2, 5157329.8) | 121.0 (89.9, 152.0) |
| 2031 | Female | 4200432.6 (2933735.4, 5467129.8) | 120.4 (84.7, 156.2) |
| 2032 | Female | 4312235.5 (2823306.8, 5801164.1) | 120.0 (79.2, 160.7) |
| 2033 | Female | 4432209.0 (2697809.4, 6166608.6) | 119.6 (73.7, 165.4) |
| 2034 | Female | 4562762.1 (2556597.9, 6568926.2) | 119.2 (68.0, 170.5) |
| 2035 | Female | 4702568.6 (2396329.5, 7008807.7) | 119.0 (62.2, 175.8) |
| 2036 | Female | 4847356.1 (2212168.8, 7482543.4) | 118.7 (56.2, 181.2) |
| 2037 | Female | 4997610.0 (2001658.9, 7993561.1) | 118.4 (50.0, 186.9) |
| 2038 | Female | 5158183.9 (1764051.9, 8552315.9) | 118.3 (43.7, 192.8) |
| 2039 | Female | 5331908.9 (1496217.0, 9167600.9) | 118.1 (37.3, 199.0) |
| 2040 | Female | 5516119.2 (1192612.1, 9839626.2) | 118.0 (30.7, 205.4) |
| 2041 | Female | 5705990.0 (848146.4, 10564550.9) | 117.9 (24.0, 211.9) |
| 2042 | Female | 5901463.2 (458471.4, 11347053.8) | 117.8 (17.1, 218.6) |
| 2043 | Female | 6104438.3 (21075.9, 12195656.5) | 117.8 (10.1, 225.5) |
| 2044 | Female | 6318707.4 (0.0, 13124521.6) | 117.7 (2.9, 232.5) |
| 2045 | Female | 6545555.8 (0.0, 14144509.8) | 117.6 (-4.5, 239.7) |
| 2022 | Male | 3625373.1 (3475111.1, 3775635.2) | 161.8 (157.7, 166.0) |
| 2023 | Male | 3717766.5 (3510656.5, 3924876.5) | 161.2 (153.9, 168.4) |
| 2024 | Male | 3817723.3 (3523802.1, 4111644.5) | 160.6 (149.4, 171.7) |
| 2025 | Male | 3921944.8 (3516201.8, 4327687.8) | 160.0 (144.3, 175.7) |
| 2026 | Male | 4027087.6 (3487329.8, 4566845.4) | 159.4 (138.7, 180.0) |
| 2027 | Male | 4133209.4 (3438118.0, 4828300.8) | 158.8 (132.8, 184.9) |
| 2028 | Male | 4243406.2 (3371792.8, 5115019.6) | 158.4 (126.5, 190.3) |
| 2029 | Male | 4359618.7 (3289439.7, 5429797.7) | 158.0 (119.9, 196.1) |
| 2030 | Male | 4480772.1 (3189159.6, 5772384.6) | 157.6 (113.0, 202.3) |
| 2031 | Male | 4605480.0 (3068531.7, 6142428.3) | 157.3 (105.7, 208.8) |
| 2032 | Male | 4735562.1 (2927000.3, 6544123.9) | 157.0 (98.2, 215.7) |
| 2033 | Male | 4874480.0 (2764995.3, 6983964.7) | 156.8 (90.5, 223.0) |
| 2034 | Male | 5023932.1 (2580766.0, 7467098.2) | 156.6 (82.5, 230.7) |
| 2035 | Male | 5182626.2 (2370322.8, 7994929.5) | 156.5 (74.3, 238.8) |
| 2036 | Male | 5348872.1 (2129250.1, 8568494.1) | 156.5 (65.8, 247.1) |
| 2037 | Male | 5524111.8 (1854100.9, 9194122.8) | 156.5 (57.1, 255.9) |
| 2038 | Male | 5711527.5 (1541697.7, 9881357.2) | 156.6 (48.1, 265.1) |
| 2039 | Male | 5913413.1 (1186901.8, 10639924.5) | 156.7 (38.9, 274.6) |
| 2040 | Male | 6128377.9 (783290.1, 11474198.0) | 156.9 (29.4, 284.4) |
| 2041 | Male | 6354822.0 (323880.7, 12388752.4) | 157.1 (19.7, 294.5) |
| 2042 | Male | 6594207.6 (0.0, 13394868.0) | 157.3 (9.7, 305.0) |
| 2043 | Male | 6847878.1 (0.0, 14504491.2) | 157.6 (-0.6, 315.9) |
| 2044 | Male | 7119039.4 (0.0, 15736058.7) | 157.9 (-11.2, 326.9) |
| 2045 | Male | 7409435.3 (0.0, 17107751.2) | 158.1 (-22.0, 338.2) |

**Notes:** ASR: age-standardized rates; DALYs: disability-adjusted life years.

**Table S9. Mortality, and DALYs of future forecasts in kidney dysfunction exposure-associated intracerebral hemorrhage using bayesian age-period-cohort model.**

| **Year** | **Sex** | **Number** | **ASR** |
| --- | --- | --- | --- |
| **Deaths** |  |  |  |
| 2022 | Both | 333792.3 (319202.5, 348382.1) | 6.8 (6.5, 7.1) |
| 2023 | Both | 339909.0 (320574.7, 359243.3) | 6.7 (6.3, 7.1) |
| 2024 | Both | 346391.4 (319856.5, 372926.3) | 6.7 (6.2, 7.2) |
| 2025 | Both | 353183.2 (317408.7, 388957.7) | 6.6 (5.9, 7.3) |
| 2026 | Both | 360143.7 (313344.6, 406942.7) | 6.6 (5.7, 7.4) |
| 2027 | Both | 367124.7 (307638.8, 426610.6) | 6.5 (5.5, 7.6) |
| 2028 | Both | 374212.5 (300465.8, 447959.3) | 6.5 (5.2, 7.7) |
| 2029 | Both | 381612.6 (292010.1, 471215.0) | 6.4 (4.9, 7.9) |
| 2030 | Both | 389338.4 (282239.7, 496437.0) | 6.4 (4.6, 8.2) |
| 2031 | Both | 397330.7 (271027.1, 523634.4) | 6.4 (4.3, 8.4) |
| 2032 | Both | 405531.9 (258223.9, 552840.0) | 6.3 (4.0, 8.6) |
| 2033 | Both | 414048.9 (243830.7, 584267.1) | 6.3 (3.7, 8.9) |
| 2034 | Both | 423068.8 (227835.8, 618301.8) | 6.3 (3.4, 9.2) |
| 2035 | Both | 432617.9 (210076.7, 655159.0) | 6.3 (3.1, 9.5) |
| 2036 | Both | 442658.3 (190316.7, 694999.9) | 6.3 (2.7, 9.9) |
| 2037 | Both | 453153.7 (168290.5, 738016.9) | 6.3 (2.3, 10.3) |
| 2038 | Both | 464322.8 (143846.9, 784798.6) | 6.3 (2.0, 10.7) |
| 2039 | Both | 476341.4 (116717.7, 835965.0) | 6.3 (1.5, 11.1) |
| 2040 | Both | 489064.8 (86517.9, 891656.0) | 6.4 (1.1, 11.6) |
| 2041 | Both | 502322.4 (52828.1, 951989.8) | 6.4 (0.7, 12.1) |
| 2042 | Both | 515975.8 (15947.7, 1017162.6) | 6.4 (0.2, 12.7) |
| 2043 | Both | 530048.4 (0.0, 1087662.6) | 6.5 (0.0, 13.3) |
| 2044 | Both | 544849.4 (0.0, 1164683.1) | 6.5 (0.0, 14.0) |
| 2045 | Both | 560564.2 (0.0, 1249349.7) | 6.6 (0.0, 14.8) |
| 2022 | Female | 154640.5 (148219.1, 161061.9) | 5.8 (5.7, 6.0) |
| 2023 | Female | 157074.2 (148525.0, 165623.4) | 5.8 (5.5, 6.0) |
| 2024 | Female | 159638.9 (147884.4, 171393.3) | 5.7 (5.3, 6.1) |
| 2025 | Female | 162331.8 (146492.5, 178171.0) | 5.6 (5.1, 6.1) |
| 2026 | Female | 165100.8 (144412.7, 185788.9) | 5.5 (4.9, 6.2) |
| 2027 | Female | 167871.5 (141625.6, 194117.5) | 5.5 (4.7, 6.3) |
| 2028 | Female | 170663.8 (138201.3, 203126.4) | 5.4 (4.4, 6.4) |
| 2029 | Female | 173560.9 (134224.7, 212897.2) | 5.4 (4.2, 6.5) |
| 2030 | Female | 176572.6 (129694.3, 223451.0) | 5.3 (3.9, 6.7) |
| 2031 | Female | 179669.9 (124560.0, 234779.8) | 5.2 (3.7, 6.8) |
| 2032 | Female | 182824.6 (118761.8, 246887.4) | 5.2 (3.4, 6.9) |
| 2033 | Female | 186093.7 (112319.9, 259867.5) | 5.1 (3.1, 7.1) |
| 2034 | Female | 189553.5 (105240.2, 273866.8) | 5.0 (2.9, 7.2) |
| 2035 | Female | 193205.2 (97462.1, 288948.4) | 5.0 (2.6, 7.4) |
| 2036 | Female | 197017.5 (88892.6, 305142.5) | 4.9 (2.3, 7.5) |
| 2037 | Female | 200969.0 (79433.0, 322504.9) | 4.9 (2.0, 7.7) |
| 2038 | Female | 205183.6 (69055.8, 341311.5) | 4.8 (1.8, 7.9) |
| 2039 | Female | 209726.6 (57669.0, 361784.3) | 4.8 (1.5, 8.0) |
| 2040 | Female | 214498.7 (45125.4, 383888.3) | 4.7 (1.2, 8.2) |
| 2041 | Female | 219387.6 (31258.9, 407562.6) | 4.6 (0.9, 8.4) |
| 2042 | Female | 224314.0 (15947.7, 432803.0) | 4.6 (0.7, 8.5) |
| 2043 | Female | 229314.9 (0.0, 459807.0) | 4.5 (0.4, 8.7) |
| 2044 | Female | 234527.4 (0.0, 489018.8) | 4.5 (0.1, 8.9) |
| 2045 | Female | 240017.8 (0.0, 520803.9) | 4.4 (-0.2, 9.0) |
| 2022 | Male | 179151.8 (170983.3, 187320.2) | 7.9 (7.7, 8.1) |
| 2023 | Male | 182834.8 (172049.7, 193620.0) | 7.8 (7.5, 8.2) |
| 2024 | Male | 186752.6 (171972.1, 201533.0) | 7.8 (7.2, 8.3) |
| 2025 | Male | 190851.4 (170916.1, 210786.7) | 7.7 (7.0, 8.5) |
| 2026 | Male | 195042.9 (168931.9, 221153.8) | 7.7 (6.7, 8.7) |
| 2027 | Male | 199253.2 (166013.2, 232493.2) | 7.6 (6.4, 8.9) |
| 2028 | Male | 203548.7 (162264.5, 244832.9) | 7.6 (6.1, 9.1) |
| 2029 | Male | 208051.6 (157785.4, 258317.8) | 7.5 (5.8, 9.3) |
| 2030 | Male | 212765.8 (152545.5, 272986.1) | 7.5 (5.4, 9.6) |
| 2031 | Male | 217660.8 (146467.1, 288854.6) | 7.5 (5.1, 9.9) |
| 2032 | Male | 222707.3 (139462.1, 305952.5) | 7.4 (4.7, 10.1) |
| 2033 | Male | 227955.2 (131510.8, 324399.7) | 7.4 (4.3, 10.4) |
| 2034 | Male | 233515.3 (122595.6, 344434.9) | 7.4 (4.0, 10.7) |
| 2035 | Male | 239412.7 (112614.7, 366210.6) | 7.3 (3.6, 11.1) |
| 2036 | Male | 245640.8 (101424.1, 389857.5) | 7.3 (3.2, 11.4) |
| 2037 | Male | 252184.8 (88857.5, 415512.0) | 7.3 (2.8, 11.8) |
| 2038 | Male | 259139.1 (74791.1, 443487.2) | 7.2 (2.4, 12.1) |
| 2039 | Male | 266614.8 (59048.8, 474180.7) | 7.2 (1.9, 12.5) |
| 2040 | Male | 274566.0 (41392.5, 507767.7) | 7.2 (1.5, 12.8) |
| 2041 | Male | 282934.8 (21569.2, 544427.2) | 7.2 (1.1, 13.2) |
| 2042 | Male | 291661.8 (0.0, 584359.6) | 7.1 (0.6, 13.6) |
| 2043 | Male | 300733.6 (0.0, 627855.6) | 7.1 (0.2, 14.0) |
| 2044 | Male | 310322.1 (0.0, 675664.3) | 7.1 (-0.3, 14.4) |
| 2045 | Male | 320546.4 (0.0, 728545.8) | 7.1 (-0.7, 14.8) |
| **DALYs** |  |  |  |
| 2022 | Both | 8243704.1 (7889406.8, 8598001.4) | 165.8 (158.7, 172.9) |
| 2023 | Both | 8392426.8 (7931778.3, 8853075.3) | 164.8 (155.7, 173.8) |
| 2024 | Both | 8544187.6 (7919883.0, 9168492.1) | 163.8 (151.8, 175.8) |
| 2025 | Both | 8698315.1 (7862620.6, 9534009.6) | 162.9 (147.2, 178.5) |
| 2026 | Both | 8855078.7 (7766590.1, 9943567.3) | 162.0 (142.1, 181.9) |
| 2027 | Both | 9014250.6 (7634651.2, 10393850.0) | 161.3 (136.6, 186.0) |
| 2028 | Both | 9175281.7 (7468793.5, 10881769.9) | 160.6 (130.7, 190.5) |
| 2029 | Both | 9339377.5 (7270822.7, 11407932.2) | 160.0 (124.6, 195.5) |
| 2030 | Both | 9506141.0 (7040155.3, 11972126.7) | 159.5 (118.1, 200.9) |
| 2031 | Both | 9676925.7 (6776665.8, 12577185.6) | 159.1 (111.4, 206.8) |
| 2032 | Both | 9853073.2 (6479421.9, 13226724.6) | 158.8 (104.4, 213.3) |
| 2033 | Both | 10034707.9 (6147240.7, 13922175.1) | 158.6 (97.1, 220.2) |
| 2034 | Both | 10223107.0 (5778802.0, 14667411.9) | 158.6 (89.5, 227.6) |
| 2035 | Both | 10418103.2 (5371138.0, 15465068.4) | 158.6 (81.6, 235.5) |
| 2036 | Both | 10621327.7 (4921464.4, 16321191.0) | 158.8 (73.4, 244.1) |
| 2037 | Both | 10834560.6 (4426082.0, 17243039.1) | 159.1 (64.8, 253.4) |
| 2038 | Both | 11059933.5 (3881530.2, 18238336.8) | 159.6 (55.8, 263.4) |
| 2039 | Both | 11298741.2 (3282543.0, 19314939.4) | 160.3 (46.3, 274.2) |
| 2040 | Both | 11548452.1 (2621627.0, 20475277.2) | 161.1 (36.3, 285.9) |
| 2041 | Both | 11808766.6 (1892951.8, 21726318.7) | 162.0 (25.6, 298.5) |
| 2042 | Both | 12079901.3 (1092735.9, 23077071.2) | 163.2 (14.4, 312.3) |
| 2043 | Both | 12361962.0 (444696.0, 24536294.2) | 164.7 (5.7, 327.3) |
| 2044 | Both | 12658646.2 (94304.0, 26122003.0) | 166.3 (1.2, 343.7) |
| 2045 | Both | 12972006.5 (0.0, 27851633.4) | 168.1 (0.0, 361.6) |
| 2022 | Female | 3611739.7 (3466651.7, 3756827.7) | 138.2 (135.0, 141.4) |
| 2023 | Female | 3669721.7 (3480901.6, 3858541.9) | 136.9 (131.5, 142.3) |
| 2024 | Female | 3728408.7 (3472501.4, 3984316.0) | 135.6 (127.5, 143.8) |
| 2025 | Female | 3787571.8 (3445389.4, 4129754.3) | 134.4 (123.1, 145.6) |
| 2026 | Female | 3847768.1 (3402796.5, 4292739.7) | 133.2 (118.5, 147.9) |
| 2027 | Female | 3908754.6 (3345813.3, 4471695.9) | 132.0 (113.6, 150.4) |
| 2028 | Female | 3969982.9 (3275131.7, 4664834.2) | 130.9 (108.5, 153.2) |
| 2029 | Female | 4031655.9 (3191431.7, 4871880.2) | 129.7 (103.3, 156.2) |
| 2030 | Female | 4093460.9 (3094571.5, 5092350.2) | 128.6 (97.8, 159.3) |
| 2031 | Female | 4156303.2 (2984979.3, 5327627.1) | 127.4 (92.2, 162.6) |
| 2032 | Female | 4220580.1 (2862356.2, 5578804.0) | 126.3 (86.5, 166.1) |
| 2033 | Female | 4286246.7 (2726442.7, 5846050.8) | 125.2 (80.7, 169.8) |
| 2034 | Female | 4353578.1 (2576891.2, 6130265.0) | 124.1 (74.7, 173.5) |
| 2035 | Female | 4422411.1 (2412839.6, 6431982.5) | 123.0 (68.7, 177.3) |
| 2036 | Female | 4493746.2 (2233775.4, 6753716.9) | 121.9 (62.5, 181.2) |
| 2037 | Female | 4568102.8 (2038538.5, 7097667.1) | 120.8 (56.3, 185.3) |
| 2038 | Female | 4646401.1 (1826328.8, 7466473.3) | 119.8 (50.1, 189.4) |
| 2039 | Female | 4728776.2 (1595540.1, 7862012.3) | 118.7 (43.8, 193.6) |
| 2040 | Female | 4813842.6 (1343797.3, 8283887.9) | 117.6 (37.4, 197.8) |
| 2041 | Female | 4901459.9 (1068978.1, 8733941.6) | 116.5 (31.0, 202.1) |
| 2042 | Female | 4991187.7 (769766.6, 9213778.8) | 115.5 (24.6, 206.4) |
| 2043 | Female | 5083015.3 (444696.0, 9725495.7) | 114.4 (18.1, 210.7) |
| 2044 | Female | 5178259.1 (94304.0, 10274327.2) | 113.3 (11.6, 215.1) |
| 2045 | Female | 5277864.5 (0.0, 10865575.1) | 112.3 (5.1, 219.5) |
| 2022 | Male | 4631964.4 (4422755.1, 4841173.7) | 195.4 (190.3, 200.4) |
| 2023 | Male | 4722705.1 (4450876.7, 4994533.5) | 194.5 (186.0, 203.1) |
| 2024 | Male | 4815778.9 (4447381.7, 5184176.2) | 193.7 (180.8, 206.6) |
| 2025 | Male | 4910743.3 (4417231.2, 5404255.4) | 193.0 (175.0, 210.9) |
| 2026 | Male | 5007310.6 (4363793.6, 5650827.7) | 192.2 (168.7, 215.7) |
| 2027 | Male | 5105496.0 (4288837.9, 5922154.1) | 191.6 (162.0, 221.2) |
| 2028 | Male | 5205298.7 (4193661.8, 6216935.7) | 191.0 (154.9, 227.1) |
| 2029 | Male | 5307721.6 (4079391.1, 6536052.1) | 190.4 (147.3, 233.4) |
| 2030 | Male | 5412680.1 (3945583.8, 6879776.5) | 189.8 (139.5, 240.1) |
| 2031 | Male | 5520622.5 (3791686.5, 7249558.5) | 189.2 (131.3, 247.1) |
| 2032 | Male | 5632493.1 (3617065.6, 7647920.6) | 188.6 (122.8, 254.5) |
| 2033 | Male | 5748461.1 (3420798.0, 8076124.2) | 188.1 (114.0, 262.3) |
| 2034 | Male | 5869528.9 (3201910.9, 8537147.0) | 187.6 (104.9, 270.3) |
| 2035 | Male | 5995692.1 (2958298.4, 9033085.8) | 187.1 (95.6, 278.7) |
| 2036 | Male | 6127581.5 (2687688.9, 9567474.1) | 186.6 (86.0, 287.3) |
| 2037 | Male | 6266457.8 (2387543.5, 10145372.0) | 186.2 (76.2, 296.2) |
| 2038 | Male | 6413532.4 (2055201.4, 10771863.5) | 185.7 (66.1, 305.4) |
| 2039 | Male | 6569965.0 (1687002.9, 11452927.2) | 185.3 (55.8, 314.8) |
| 2040 | Male | 6734609.5 (1277829.7, 12191389.3) | 184.8 (45.3, 324.4) |
| 2041 | Male | 6907306.7 (823973.6, 12992377.1) | 184.4 (34.5, 334.2) |
| 2042 | Male | 7088713.6 (322969.4, 13863292.4) | 184.0 (23.6, 344.3) |
| 2043 | Male | 7278946.7 (0.0, 14810798.5) | 183.5 (12.4, 354.6) |
| 2044 | Male | 7480387.1 (0.0, 15847675.8) | 183.1 (1.1, 365.1) |
| 2045 | Male | 7694142.0 (0.0, 16986058.3) | 182.6 (-10.4, 375.7) |

**Notes:** ASR: age-standardized rates; DALYs: disability-adjusted life years.

**Table S10. Mortality, and DALYs of future forecasts in kidney dysfunction exposure-associated stroke using nordpred model.**

| **Year** | **Sex** | **Number** | **ASR** |
| --- | --- | --- | --- |
| **Deaths** |  |  |  |
| 2022 | Both | 665799 | 13.8 |
| 2023 | Both | 674399 | 13.5 |
| 2024 | Both | 683660 | 13.3 |
| 2025 | Both | 697631 | 13.2 |
| 2026 | Both | 711486 | 13.0 |
| 2027 | Both | 724719 | 12.9 |
| 2028 | Both | 737751 | 12.8 |
| 2029 | Both | 751160 | 12.6 |
| 2030 | Both | 768972 | 12.6 |
| 2031 | Both | 786883 | 12.5 |
| 2032 | Both | 804602 | 12.4 |
| 2033 | Both | 822574 | 12.3 |
| 2034 | Both | 841179 | 12.3 |
| 2035 | Both | 864562 | 12.3 |
| 2036 | Both | 888058 | 12.3 |
| 2037 | Both | 911282 | 12.3 |
| 2038 | Both | 934961 | 12.3 |
| 2039 | Both | 959493 | 12.3 |
| 2040 | Both | 985625 | 12.3 |
| 2041 | Both | 1011219 | 12.3 |
| 2042 | Both | 1035634 | 12.3 |
| 2043 | Both | 1059189 | 12.3 |
| 2044 | Both | 1082597 | 12.3 |
| 2045 | Both | 1105823 | 12.3 |
| 2022 | Female | 325303 | 12.1 |
| 2023 | Female | 329470 | 12.0 |
| 2024 | Female | 333958 | 11.8 |
| 2025 | Female | 340723 | 11.6 |
| 2026 | Female | 347497 | 11.5 |
| 2027 | Female | 354038 | 11.4 |
| 2028 | Female | 360511 | 11.3 |
| 2029 | Female | 367177 | 11.2 |
| 2030 | Female | 376003 | 11.1 |
| 2031 | Female | 384930 | 11.0 |
| 2032 | Female | 393824 | 11.0 |
| 2033 | Female | 402881 | 10.9 |
| 2034 | Female | 412267 | 10.8 |
| 2035 | Female | 423987 | 10.8 |
| 2036 | Female | 435792 | 10.8 |
| 2037 | Female | 447492 | 10.8 |
| 2038 | Female | 459457 | 10.8 |
| 2039 | Female | 471864 | 10.8 |
| 2040 | Female | 485168 | 10.8 |
| 2041 | Female | 498159 | 10.8 |
| 2042 | Female | 510491 | 10.8 |
| 2043 | Female | 522319 | 10.9 |
| 2044 | Female | 534023 | 10.9 |
| 2045 | Female | 545603 | 10.9 |
| 2022 | Male | 340496 | 15.6 |
| 2023 | Male | 344929 | 15.4 |
| 2024 | Male | 349702 | 15.1 |
| 2025 | Male | 356908 | 14.9 |
| 2026 | Male | 363990 | 14.8 |
| 2027 | Male | 370680 | 14.6 |
| 2028 | Male | 377240 | 14.5 |
| 2029 | Male | 383983 | 14.3 |
| 2030 | Male | 392969 | 14.2 |
| 2031 | Male | 401953 | 14.1 |
| 2032 | Male | 410778 | 14.1 |
| 2033 | Male | 419693 | 14.0 |
| 2034 | Male | 428912 | 13.9 |
| 2035 | Male | 440575 | 13.9 |
| 2036 | Male | 452266 | 13.9 |
| 2037 | Male | 463789 | 13.9 |
| 2038 | Male | 475504 | 13.9 |
| 2039 | Male | 487629 | 13.9 |
| 2040 | Male | 500457 | 13.9 |
| 2041 | Male | 513060 | 13.9 |
| 2042 | Male | 525143 | 14.0 |
| 2043 | Male | 536870 | 14.0 |
| 2044 | Male | 548574 | 14.0 |
| 2045 | Male | 560220 | 14.0 |
| **DALYs** |  |  |  |
| 2022 | Both | 14999396 | 304.1 |
| 2023 | Both | 15167735 | 299.5 |
| 2024 | Both | 15338608 | 294.9 |
| 2025 | Both | 15601946 | 292.1 |
| 2026 | Both | 15859785 | 289.3 |
| 2027 | Both | 16105300 | 286.6 |
| 2028 | Both | 16342357 | 283.8 |
| 2029 | Both | 16577601 | 281.0 |
| 2030 | Both | 16894820 | 279.7 |
| 2031 | Both | 17209077 | 278.3 |
| 2032 | Both | 17517217 | 277.0 |
| 2033 | Both | 17823953 | 275.6 |
| 2034 | Both | 18133410 | 274.3 |
| 2035 | Both | 18528532 | 274.2 |
| 2036 | Both | 18921648 | 274.2 |
| 2037 | Both | 19308223 | 274.1 |
| 2038 | Both | 19695745 | 274.0 |
| 2039 | Both | 20088805 | 274.0 |
| 2040 | Both | 20498837 | 274.1 |
| 2041 | Both | 20899817 | 274.2 |
| 2042 | Both | 21284774 | 274.4 |
| 2043 | Both | 21656268 | 274.5 |
| 2044 | Both | 22021988 | 274.7 |
| 2045 | Both | 22382000 | 274.8 |
| 2022 | Female | 6927939 | 262.7 |
| 2023 | Female | 7012819 | 258.9 |
| 2024 | Female | 7099494 | 255.2 |
| 2025 | Female | 7228080 | 252.9 |
| 2026 | Female | 7354894 | 250.6 |
| 2027 | Female | 7476684 | 248.2 |
| 2028 | Female | 7594909 | 245.9 |
| 2029 | Female | 7712590 | 243.6 |
| 2030 | Female | 7867607 | 242.5 |
| 2031 | Female | 8021749 | 241.4 |
| 2032 | Female | 8173595 | 240.3 |
| 2033 | Female | 8325219 | 239.2 |
| 2034 | Female | 8478412 | 238.1 |
| 2035 | Female | 8670059 | 238.0 |
| 2036 | Female | 8860890 | 237.9 |
| 2037 | Female | 9048759 | 237.8 |
| 2038 | Female | 9237427 | 237.7 |
| 2039 | Female | 9428976 | 237.6 |
| 2040 | Female | 9629412 | 237.7 |
| 2041 | Female | 9824903 | 237.8 |
| 2042 | Female | 10011823 | 237.9 |
| 2043 | Female | 10191367 | 238.0 |
| 2044 | Female | 10367502 | 238.1 |
| 2045 | Female | 10540487 | 238.2 |
| 2022 | Male | 8071457 | 349.4 |
| 2023 | Male | 8154916 | 343.9 |
| 2024 | Male | 8239114 | 338.4 |
| 2025 | Male | 8373866 | 335.1 |
| 2026 | Male | 8504891 | 331.8 |
| 2027 | Male | 8628617 | 328.4 |
| 2028 | Male | 8747448 | 325.1 |
| 2029 | Male | 8865011 | 321.8 |
| 2030 | Male | 9027213 | 320.2 |
| 2031 | Male | 9187328 | 318.6 |
| 2032 | Male | 9343621 | 316.9 |
| 2033 | Male | 9498734 | 315.3 |
| 2034 | Male | 9654999 | 313.7 |
| 2035 | Male | 9858473 | 313.6 |
| 2036 | Male | 10060757 | 313.6 |
| 2037 | Male | 10259464 | 313.5 |
| 2038 | Male | 10458318 | 313.4 |
| 2039 | Male | 10659829 | 313.4 |
| 2040 | Male | 10869424 | 313.5 |
| 2041 | Male | 11074914 | 313.7 |
| 2042 | Male | 11272951 | 313.8 |
| 2043 | Male | 11464901 | 314.0 |
| 2044 | Male | 11654486 | 314.2 |
| 2045 | Male | 11841513 | 314.3 |

**Notes:** ASR: age-standardized rates; DALYs: disability-adjusted life years.

**Table S11. Mortality, and DALYs of future forecasts in kidney dysfunction exposure-associated ischemic stroke using nordpred model.**

| **Year** | **Sex** | **Number** | **ASR** |
| --- | --- | --- | --- |
| **Deaths** |  |  |  |
| 2022 | Both | 338092 | 7.1 |
| 2023 | Both | 343724 | 7.0 |
| 2024 | Both | 349950 | 6.9 |
| 2025 | Both | 358535 | 6.8 |
| 2026 | Both | 367165 | 6.8 |
| 2027 | Both | 375503 | 6.7 |
| 2028 | Both | 383844 | 6.6 |
| 2029 | Both | 392586 | 6.6 |
| 2030 | Both | 403595 | 6.5 |
| 2031 | Both | 414803 | 6.5 |
| 2032 | Both | 426020 | 6.5 |
| 2033 | Both | 437583 | 6.5 |
| 2034 | Both | 449755 | 6.4 |
| 2035 | Both | 464563 | 6.4 |
| 2036 | Both | 479534 | 6.4 |
| 2037 | Both | 494371 | 6.4 |
| 2038 | Both | 509571 | 6.5 |
| 2039 | Both | 525430 | 6.5 |
| 2040 | Both | 542482 | 6.5 |
| 2041 | Both | 559258 | 6.5 |
| 2042 | Both | 575326 | 6.5 |
| 2043 | Both | 590939 | 6.5 |
| 2044 | Both | 606595 | 6.5 |
| 2045 | Both | 622239 | 6.6 |
| 2022 | Female | 172297 | 6.4 |
| 2023 | Female | 174942 | 6.3 |
| 2024 | Female | 177867 | 6.2 |
| 2025 | Female | 182032 | 6.1 |
| 2026 | Female | 186263 | 6.1 |
| 2027 | Female | 190398 | 6.0 |
| 2028 | Female | 194548 | 6.0 |
| 2029 | Female | 198900 | 5.9 |
| 2030 | Female | 204465 | 5.9 |
| 2031 | Female | 210174 | 5.9 |
| 2032 | Female | 215940 | 5.8 |
| 2033 | Female | 221916 | 5.8 |
| 2034 | Female | 228219 | 5.8 |
| 2035 | Female | 235923 | 5.8 |
| 2036 | Female | 243743 | 5.8 |
| 2037 | Female | 251524 | 5.8 |
| 2038 | Female | 259523 | 5.8 |
| 2039 | Female | 267876 | 5.8 |
| 2040 | Female | 276946 | 5.8 |
| 2041 | Female | 285850 | 5.8 |
| 2042 | Female | 294344 | 5.9 |
| 2043 | Female | 302553 | 5.9 |
| 2044 | Female | 310747 | 5.9 |
| 2045 | Female | 318915 | 5.9 |
| 2022 | Male | 165796 | 7.9 |
| 2023 | Male | 168782 | 7.8 |
| 2024 | Male | 172084 | 7.7 |
| 2025 | Male | 176502 | 7.6 |
| 2026 | Male | 180902 | 7.6 |
| 2027 | Male | 185106 | 7.5 |
| 2028 | Male | 189295 | 7.4 |
| 2029 | Male | 193686 | 7.4 |
| 2030 | Male | 199130 | 7.3 |
| 2031 | Male | 204629 | 7.3 |
| 2032 | Male | 210080 | 7.3 |
| 2033 | Male | 215667 | 7.2 |
| 2034 | Male | 221536 | 7.2 |
| 2035 | Male | 228639 | 7.2 |
| 2036 | Male | 235792 | 7.2 |
| 2037 | Male | 242847 | 7.2 |
| 2038 | Male | 250048 | 7.2 |
| 2039 | Male | 257554 | 7.2 |
| 2040 | Male | 265536 | 7.2 |
| 2041 | Male | 273408 | 7.3 |
| 2042 | Male | 280981 | 7.3 |
| 2043 | Male | 288386 | 7.3 |
| 2044 | Male | 295848 | 7.3 |
| 2045 | Male | 303323 | 7.3 |
| **DALYs** |  |  |  |
| 2022 | Both | 6953320 | 142.3 |
| 2023 | Both | 7074104 | 140.6 |
| 2024 | Both | 7201353 | 138.9 |
| 2025 | Both | 7371178 | 138.0 |
| 2026 | Both | 7540696 | 137.1 |
| 2027 | Both | 7705096 | 136.2 |
| 2028 | Both | 7867077 | 135.3 |
| 2029 | Both | 8031419 | 134.4 |
| 2030 | Both | 8233052 | 134.1 |
| 2031 | Both | 8435806 | 133.8 |
| 2032 | Both | 8637638 | 133.5 |
| 2033 | Both | 8842425 | 133.2 |
| 2034 | Both | 9053033 | 132.9 |
| 2035 | Both | 9305156 | 133.1 |
| 2036 | Both | 9557964 | 133.4 |
| 2037 | Both | 9807713 | 133.7 |
| 2038 | Both | 10059489 | 133.9 |
| 2039 | Both | 10316885 | 134.2 |
| 2040 | Both | 10589165 | 134.6 |
| 2041 | Both | 10857521 | 135.0 |
| 2042 | Both | 11117325 | 135.4 |
| 2043 | Both | 11370961 | 135.8 |
| 2044 | Both | 11623665 | 136.3 |
| 2045 | Both | 11874951 | 136.7 |
| 2022 | Female | 3361249 | 126.2 |
| 2023 | Female | 3420610 | 124.8 |
| 2024 | Female | 3483210 | 123.3 |
| 2025 | Female | 3567358 | 122.6 |
| 2026 | Female | 3651945 | 121.9 |
| 2027 | Female | 3734657 | 121.2 |
| 2028 | Female | 3816431 | 120.4 |
| 2029 | Female | 3899446 | 119.7 |
| 2030 | Female | 4001537 | 119.5 |
| 2031 | Female | 4104758 | 119.4 |
| 2032 | Female | 4208236 | 119.2 |
| 2033 | Female | 4313725 | 119.0 |
| 2034 | Female | 4422441 | 118.8 |
| 2035 | Female | 4551757 | 119.2 |
| 2036 | Female | 4681793 | 119.5 |
| 2037 | Female | 4810667 | 119.8 |
| 2038 | Female | 4940945 | 120.1 |
| 2039 | Female | 5074295 | 120.4 |
| 2040 | Female | 5215880 | 120.8 |
| 2041 | Female | 5355313 | 121.3 |
| 2042 | Female | 5490063 | 121.7 |
| 2043 | Female | 5621187 | 122.2 |
| 2044 | Female | 5751472 | 122.7 |
| 2045 | Female | 5880885 | 123.1 |
| 2022 | Male | 3592071 | 160.5 |
| 2023 | Male | 3653494 | 158.5 |
| 2024 | Male | 3718143 | 156.6 |
| 2025 | Male | 3803820 | 155.4 |
| 2026 | Male | 3888750 | 154.3 |
| 2027 | Male | 3970439 | 153.2 |
| 2028 | Male | 4050646 | 152.1 |
| 2029 | Male | 4131974 | 150.9 |
| 2030 | Male | 4231515 | 150.5 |
| 2031 | Male | 4331049 | 150.0 |
| 2032 | Male | 4429402 | 149.6 |
| 2033 | Male | 4528699 | 149.1 |
| 2034 | Male | 4630592 | 148.7 |
| 2035 | Male | 4753399 | 148.9 |
| 2036 | Male | 4876170 | 149.1 |
| 2037 | Male | 4997046 | 149.3 |
| 2038 | Male | 5118544 | 149.5 |
| 2039 | Male | 5242590 | 149.7 |
| 2040 | Male | 5373285 | 150.0 |
| 2041 | Male | 5502208 | 150.4 |
| 2042 | Male | 5627262 | 150.8 |
| 2043 | Male | 5749774 | 151.1 |
| 2044 | Male | 5872193 | 151.5 |
| 2045 | Male | 5994067 | 151.9 |

**Notes:**ASR: age-standardized rates; DALYs: disability-adjusted life years.

**Table S12. Mortality, and DALYs of future forecasts in kidney dysfunction exposure-associated intracerebral hemorrhage using nordpred model.**

| **Year** | **Sex** | **Number** | **ASR** |
| --- | --- | --- | --- |
| **Deaths** |  |  |  |
| 2022 | Both | 328461 | 6.7 |
| 2023 | Both | 331720 | 6.6 |
| 2024 | Both | 335068 | 6.4 |
| 2025 | Both | 340766 | 6.4 |
| 2026 | Both | 346328 | 6.3 |
| 2027 | Both | 351579 | 6.2 |
| 2028 | Both | 356649 | 6.2 |
| 2029 | Both | 361715 | 6.1 |
| 2030 | Both | 368910 | 6.1 |
| 2031 | Both | 376032 | 6.0 |
| 2032 | Both | 382980 | 6.0 |
| 2033 | Both | 389864 | 6.0 |
| 2034 | Both | 396801 | 5.9 |
| 2035 | Both | 405919 | 5.9 |
| 2036 | Both | 415014 | 5.9 |
| 2037 | Both | 423993 | 5.9 |
| 2038 | Both | 433084 | 5.9 |
| 2039 | Both | 442395 | 5.9 |
| 2040 | Both | 452255 | 5.9 |
| 2041 | Both | 461885 | 5.9 |
| 2042 | Both | 471072 | 5.9 |
| 2043 | Both | 479881 | 5.9 |
| 2044 | Both | 488532 | 5.9 |
| 2045 | Both | 497043 | 5.9 |
| 2022 | Female | 153352 | 5.8 |
| 2023 | Female | 155007 | 5.7 |
| 2024 | Female | 156714 | 5.6 |
| 2025 | Female | 159453 | 5.5 |
| 2026 | Female | 162148 | 5.5 |
| 2027 | Female | 164717 | 5.4 |
| 2028 | Female | 167209 | 5.3 |
| 2029 | Female | 169703 | 5.3 |
| 2030 | Female | 173133 | 5.2 |
| 2031 | Female | 176534 | 5.2 |
| 2032 | Female | 179858 | 5.2 |
| 2033 | Female | 183148 | 5.2 |
| 2034 | Female | 186453 | 5.1 |
| 2035 | Female | 190717 | 5.1 |
| 2036 | Female | 194967 | 5.1 |
| 2037 | Female | 199162 | 5.1 |
| 2038 | Female | 203414 | 5.1 |
| 2039 | Female | 207766 | 5.1 |
| 2040 | Female | 212387 | 5.1 |
| 2041 | Female | 216881 | 5.1 |
| 2042 | Female | 221142 | 5.1 |
| 2043 | Female | 225201 | 5.1 |
| 2044 | Female | 229165 | 5.1 |
| 2045 | Female | 233048 | 5.1 |
| 2022 | Male | 175108 | 7.7 |
| 2023 | Male | 176712 | 7.6 |
| 2024 | Male | 178354 | 7.4 |
| 2025 | Male | 181313 | 7.3 |
| 2026 | Male | 184180 | 7.3 |
| 2027 | Male | 186863 | 7.2 |
| 2028 | Male | 189439 | 7.1 |
| 2029 | Male | 192012 | 7.0 |
| 2030 | Male | 195777 | 7.0 |
| 2031 | Male | 199498 | 6.9 |
| 2032 | Male | 203122 | 6.9 |
| 2033 | Male | 206716 | 6.9 |
| 2034 | Male | 210348 | 6.8 |
| 2035 | Male | 215202 | 6.8 |
| 2036 | Male | 220047 | 6.8 |
| 2037 | Male | 224830 | 6.8 |
| 2038 | Male | 229670 | 6.8 |
| 2039 | Male | 234630 | 6.8 |
| 2040 | Male | 239868 | 6.8 |
| 2041 | Male | 245004 | 6.8 |
| 2042 | Male | 249929 | 6.8 |
| 2043 | Male | 254680 | 6.8 |
| 2044 | Male | 259367 | 6.9 |
| 2045 | Male | 263995 | 6.9 |
| **DALYs** |  |  |  |
| 2022 | Both | 8064776 | 162.2 |
| 2023 | Both | 8119550 | 159.4 |
| 2024 | Both | 8170920 | 156.6 |
| 2025 | Both | 8274989 | 154.9 |
| 2026 | Both | 8374551 | 153.2 |
| 2027 | Both | 8467523 | 151.5 |
| 2028 | Both | 8555000 | 149.8 |
| 2029 | Both | 8638872 | 148.1 |
| 2030 | Both | 8769383 | 147.2 |
| 2031 | Both | 8896604 | 146.4 |
| 2032 | Both | 9019427 | 145.6 |
| 2033 | Both | 9138728 | 144.7 |
| 2034 | Both | 9255766 | 143.9 |
| 2035 | Both | 9419133 | 143.7 |
| 2036 | Both | 9580599 | 143.6 |
| 2037 | Both | 9739221 | 143.5 |
| 2038 | Both | 9897364 | 143.4 |
| 2039 | Both | 10056098 | 143.3 |
| 2040 | Both | 10220069 | 143.3 |
| 2041 | Both | 10379768 | 143.2 |
| 2042 | Both | 10532778 | 143.2 |
| 2043 | Both | 10679297 | 143.2 |
| 2044 | Both | 10821808 | 143.1 |
| 2045 | Both | 10960799 | 143.1 |
| 2022 | Female | 3575289 | 136.8 |
| 2023 | Female | 3604132 | 134.6 |
| 2024 | Female | 3631758 | 132.3 |
| 2025 | Female | 3681677 | 130.9 |
| 2026 | Female | 3729756 | 129.5 |
| 2027 | Female | 3775019 | 128.1 |
| 2028 | Female | 3817928 | 126.7 |
| 2029 | Female | 3859323 | 125.3 |
| 2030 | Female | 3920137 | 124.5 |
| 2031 | Female | 3979403 | 123.8 |
| 2032 | Female | 4036581 | 123.0 |
| 2033 | Female | 4091993 | 122.3 |
| 2034 | Female | 4146198 | 121.5 |
| 2035 | Female | 4219686 | 121.3 |
| 2036 | Female | 4292145 | 121.2 |
| 2037 | Female | 4363228 | 121.0 |
| 2038 | Female | 4434071 | 120.8 |
| 2039 | Female | 4505114 | 120.6 |
| 2040 | Female | 4578424 | 120.5 |
| 2041 | Female | 4649487 | 120.4 |
| 2042 | Female | 4717163 | 120.3 |
| 2043 | Female | 4781557 | 120.2 |
| 2044 | Female | 4843841 | 120.0 |
| 2045 | Female | 4904290 | 119.9 |
| 2022 | Male | 4489487 | 189.4 |
| 2023 | Male | 4515418 | 186.0 |
| 2024 | Male | 4539163 | 182.7 |
| 2025 | Male | 4593311 | 180.6 |
| 2026 | Male | 4644795 | 178.6 |
| 2027 | Male | 4692504 | 176.6 |
| 2028 | Male | 4737071 | 174.5 |
| 2029 | Male | 4779549 | 172.5 |
| 2030 | Male | 4849246 | 171.5 |
| 2031 | Male | 4917202 | 170.6 |
| 2032 | Male | 4982846 | 169.6 |
| 2033 | Male | 5046735 | 168.6 |
| 2034 | Male | 5109568 | 167.7 |
| 2035 | Male | 5199446 | 167.6 |
| 2036 | Male | 5288454 | 167.5 |
| 2037 | Male | 5375994 | 167.5 |
| 2038 | Male | 5463293 | 167.4 |
| 2039 | Male | 5550984 | 167.4 |
| 2040 | Male | 5641645 | 167.4 |
| 2041 | Male | 5730281 | 167.4 |
| 2042 | Male | 5815615 | 167.5 |
| 2043 | Male | 5897741 | 167.5 |
| 2044 | Male | 5977968 | 167.5 |
| 2045 | Male | 6056509 | 167.6 |

**Notes:** ASR: age-standardized rates; DALYs: disability-adjusted life years.
